# The Arylation of α,β-Unsaturated Carbonyl Compounds by Arenediazonium Salts—A Synthetic Route to Precursors of Substituted 2-Mercaptothiazoles

**DOI:** 10.3390/molecules31162908

**Published:** 2026-08-20

**Authors:** Mary Y. Ostapiuk, Oksana V. Barabash, Mykola D. Obushak, Mykola Kravets, Andreas Schmidt, Yurii V. Ostapiuk

**Affiliations:** 1 Department of Organic Chemistry, Ivan Franko National University of Lviv, Kyryla i Mefodiya Str. 6, 79005 Lviv, Ukraine; mariiasofiia.ostapiuk@lnu.edu.ua (M.Y.O.); oksana.barabash@lnu.edu.ua (O.V.B.); mykola.obushak@lnu.edu.ua (M.D.O.); 2 Institute of Physical Chemistry, Polish Academy of Sciences, Kasprzaka 44/52, 01-224 Warsaw, Poland; mkravets@ichf.edu.pl; 3 Institute of Organic Chemistry, Clausthal University of Technology, Leibnizstrasse 6, D-38678 Clausthal-Zellerfeld, Germany

**Keywords:** arenediazonium salts, Meerwein reaction, thiazole-2-thiols, α-thiocyanatocarbonyl compounds

## Abstract

This paper presents the first examples of the use of arylation products of α,β-unsaturated carbonyl compounds under Meerwein reaction conditions for the synthesis of substituted 2-mercaptothiazoles. The method described here yields α-thiocyanatocarbonyl building blocks, which subsequently enable cyclization reactions with thiourea or mercaptoacetamides to synthesize the title compounds. In this way, 5-arylmethyl-2-mercaptothiazoles substituted at the 4-position and their S-substituted derivatives can be prepared very efficiently.

## 1. Introduction

**Figure 1:**
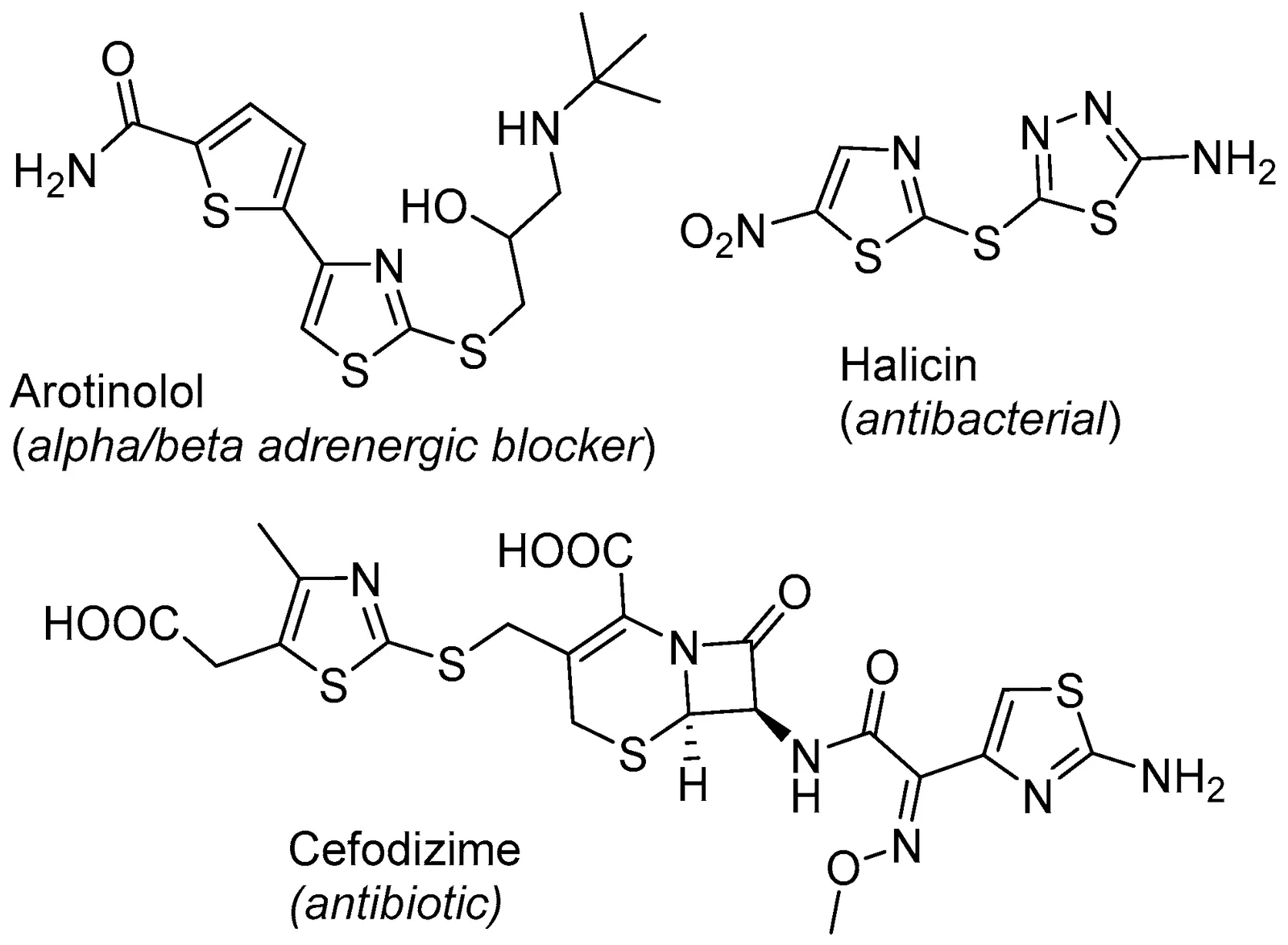
Biologically active 2-mercaptothiazoles.

Substituted thiazoles constitute an important class of heterocyclic compounds. Among these, 2-mercaptothiazole derivatives stand out in particular due to their versatile applications. For example, the 2-mercaptothiazole substructure is a significant pharmacophore in a range of drugs and bioactive compounds, spanning the spectrum from antitumor [1,2], antibacterial [3,4,5,6,7,8], and antifungal [8] agents, all the way to β-adrenoreceptor blockers [9], α-glucosidase inhibitors (for the treatment of type 2 diabetes) [10], fructose-1,6-bisphosphatase [11], and 5-HT7 ligands [12]. As examples, Figure 1 shows several structures of clinical drugs that contain the 2-mercaptothiazole pharmacophore. Furthermore, 2-mercaptothiazoles are useful building blocks for organic syntheses. For instance, the mercapto group can be easily modified by hetarylation and arylation [2,4], alkylation [5,6], the formation of S–S bonds [7,11], acylation [10], glycosylation, or oxidative sulfonation of hydrazines [13,14]. 2-Mercaptothiazoles are also used as surface modifiers for cathode materials and for the organocatalysis of C–H borylation [15,16].

**Figure 2:**
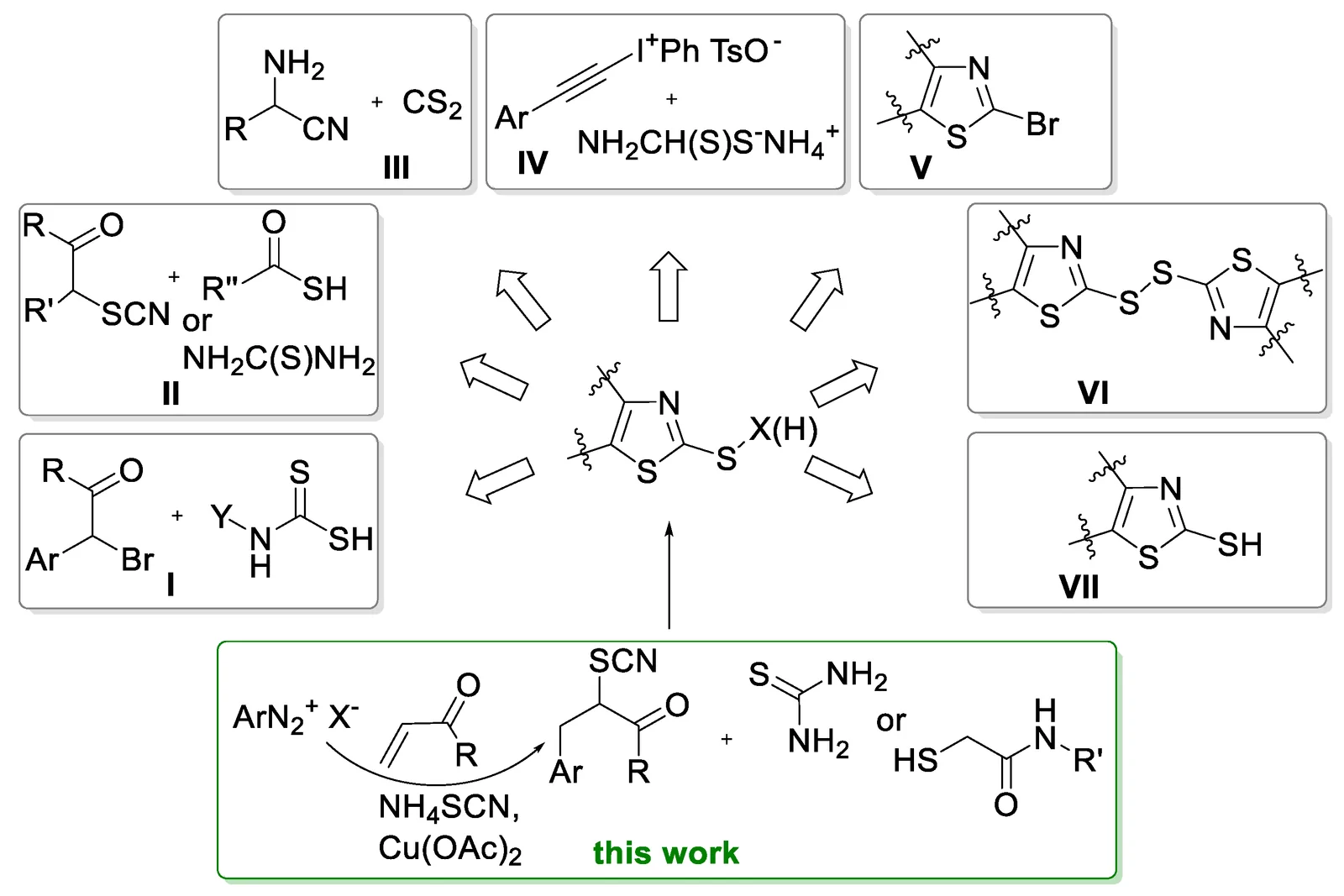
Synthetic approaches to substituted 2-mercaptothiazoles (**above**). Overview and current work (**below**).

Substituted 2-mercaptothiazoles have mostly been prepared by the reaction of α-halocarbonyl compounds **I** with carbamodithioic acid or its derivatives (Figure 2) [12,17,18,19]. Several approaches are based on the reaction of α-thiocyanatoketones **II** with alkylthiocarboxylic acids [20], or the earlier developed and unfairly forgotten reaction with thiourea [21,22]. Few approaches start from α-aminonitriles **III** [23,24], alkynyl(phenyl)iodonium salts **IV** [25], 2-halothiazoles **V** [26,27,28], 1,2-bis(thiazol-2-yl)disulfanes **VI** [29], or by modifying the 2-mercaptothiazole ring **VII** [30]. It should be noted that S-substituted 2-mercaptothiazoles are mostly obtained by modifying of the SH-group of the thiazole ring [2,4,5,6,7,8,10,11].

These methods, however, suffer from severe drawbacks. First, they are restricted to suitable and accessible precursors. Second, these often involve the use of unstable carbamodithioic acid or smelly reagents such as ammonium salts or alkylthiocarboxylic acids, respectively. Nevertheless, α-thiocyanatoketones are one of the most promising precursors for the syntheses of substituted mercaptothiazoles.

## 2. Results and Discussion

In our previous studies, we demonstrated the significant synthetic potential of Meerwein reaction products as acyclic building blocks for the construction of heterocyclic systems [31,32,33,34,35]. In continuation of our studies, we report here on preparations of derivatives of 2-mercaptothiazoles from the products of the arylation of α,β-unsaturated carbonyl compounds by aryldiazonium salts.

**Scheme 1:**
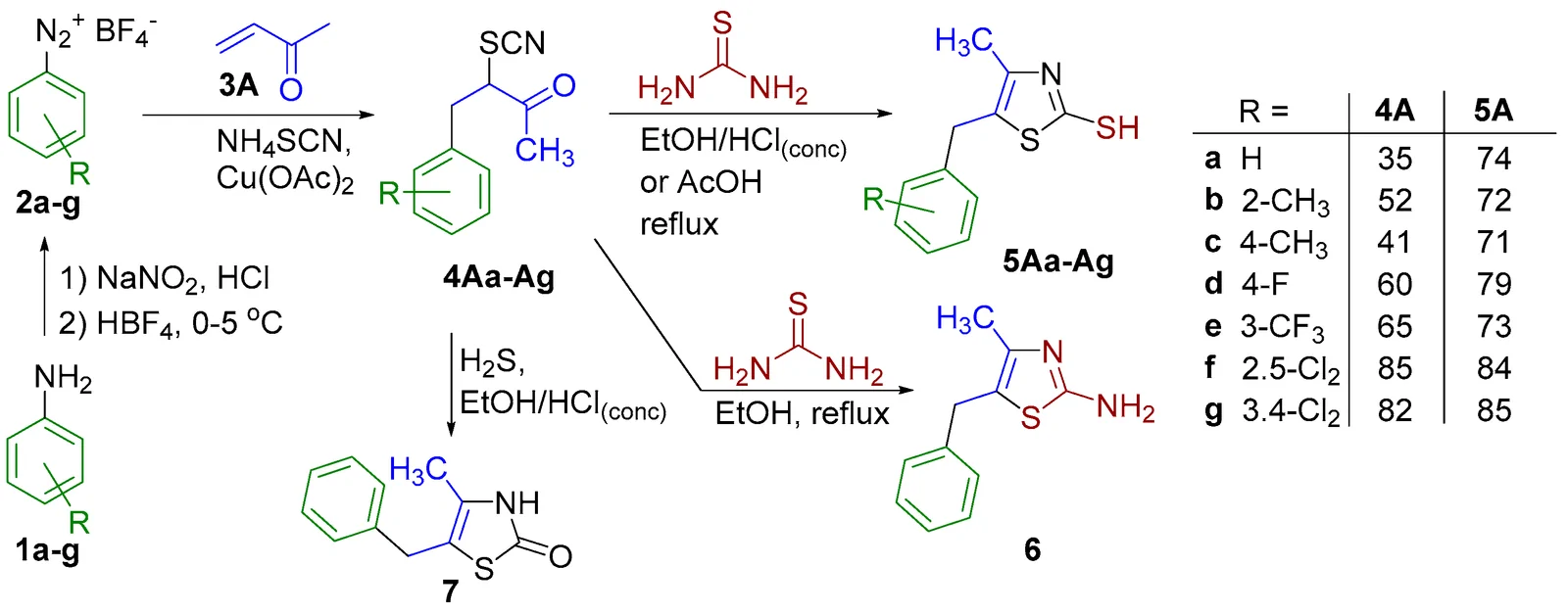
Gram-scale synthesis of 5-arylmethyl-4-methyl-2-mercaptothiazoles **5A**. Yields of **4A** and **5A** are given in %.

We started our investigation by using 4-aryl-3-thiocyanatobutan-2-ones **4Aa**–**g** to form the 2-mercaptothiazole moiety (Scheme 1). α-Thiocyanatoketones **4** were synthesized by three-component reactions of arenediazonium tetrafluoroborates **2** (obtained from appropriate anilines **1**) with methyl vinyl ketone **3A** and ammonium thiocyanate in the presence of copper(II) acetate as described by us previously [31]. It is worth noting that we obtained most of the compounds **4** in high yields as products of Meerwein-type reactions.

It has been established that thiocyanatoketones **4A** react with thiourea to form a thiazole ring. A distinctive feature of the use of reagents **4A** in this cyclization is the introduction of a benzyl substituent at the 5-position of the thiazole ring, which would be difficult to install by other methods.

As summarized in Table 1, the desired 5-benzyl-4-methyl-2-mercaptothiazole **5Aa** was obtained in different yields (entry 2–6). Best results were achieved when the reaction of thiocyanatoketone **4Aa** with thiourea was carried out in a mixture of concentrated hydrochloric acid and ethanol under reflux (Table 1, entry 2). On the other hand, the reaction of **4Aa** with thiourea in ethanol in the absence of acid led to the formation of 5-benzyl-4-methyl-2-aminothiazole **6** only in 40% yield (Table 1, entry 1).

At the same time, attempts to cyclize ketone **4Aa** with hydrogen sulfide were unsuccessful. When **4Aa** was heated in ethanol saturated with H_2_S (Table 1, entry 7), no reaction occurred at all (only the starting thiocyanatoketone **4Aa** was isolated from the reaction mixture), whereas when heated in H_2_S/ethanol/hydrochloric acid, the only product isolated was thiazol-2-one **7** in 74% yield (Table 1, entry 8). Thiazol-2-one **7** is formed by a water molecule addition to the C≡N bond of the SCN group, followed by intramolecular cyclization.

**Scheme 2:**
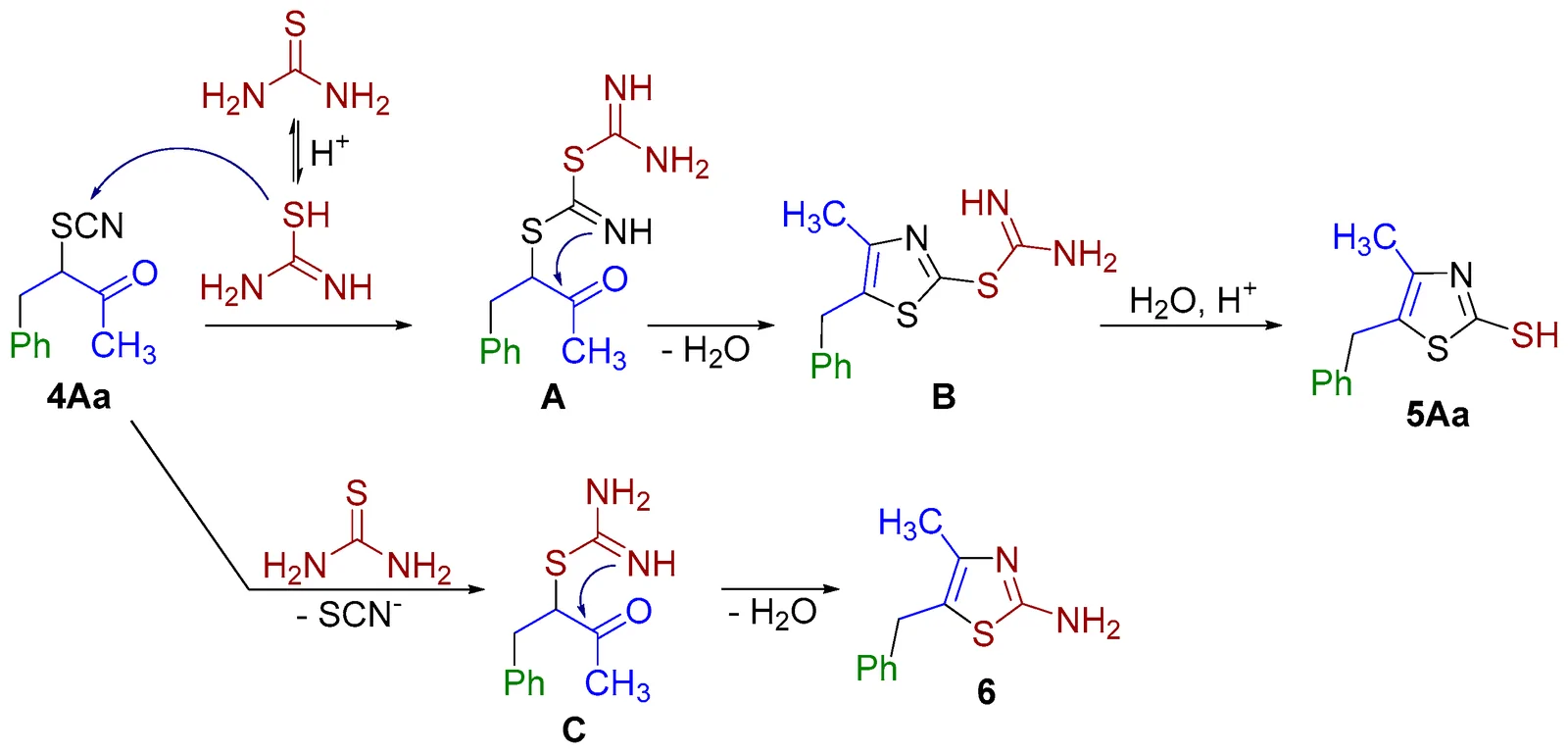
Plausible mechanism for the synthesis of **5Aa** and **6**.

We propose that the formation of the 2-mercaptothiazole fragment begins with the addition of a sulfur atom from thiourea to the C≡N bond of the SCN group of the thiocyanatoketone, resulting in the formation of intermediate **A** (Scheme 2). This is followed by an intramolecular nucleophilic addition of NH to a carbonyl group, resulting in the formation of the thiazole ring (compound **B**, Scheme 2). Subsequent hydrolysis of isothiourea **B** under acidic conditions affords the target mercaptothiazole **5Aa**. However, the formation of aminothiazole **6** is apparently the result of nucleophilic substitution of the thiocyanate group by the atom of sulfur of thiourea (compound **C**, Scheme 2) and subsequent cyclization of the formed intermediate.

We also attempted to obtain α-thiocyanatoaldehydes by the thiocyanatoarylation of acrolein, which can be considered as precursors of 4-H-2-mercapto-thiazoles. The attempt to carry out three-component reactions of aryl diazonium tetrafluoroborates **2** with acrolein **3B** and ammonium thiocyanate was unsuccessful, and only resinous substances were obtained.

**Scheme 3:**
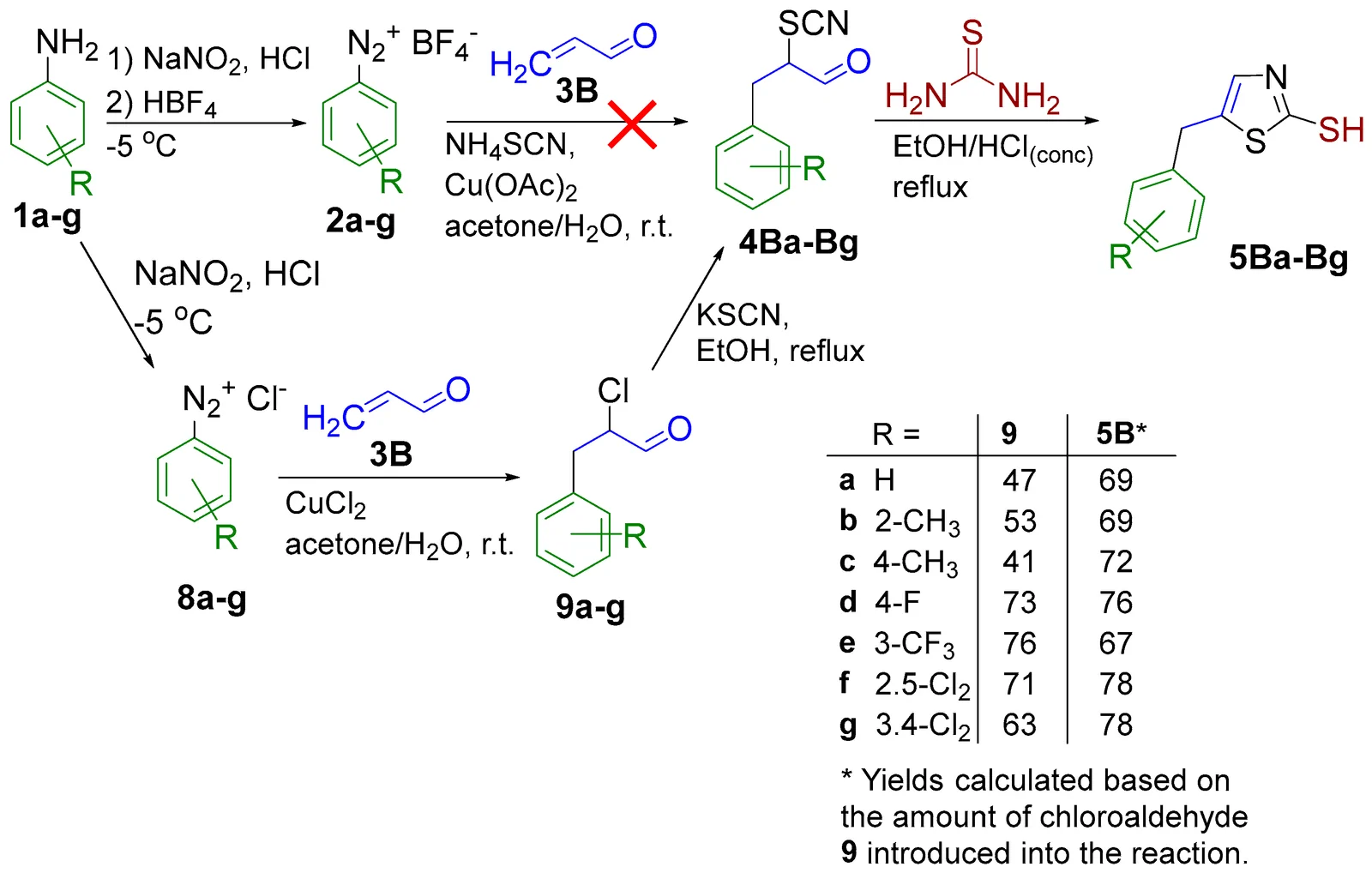
Gram-scale synthesis of 5-arylmethyl-2-mercaptothiazoles **5B**.

However, we obtained the targeted 3-aryl-2-thiocyanatopropanals **4B** from the products of chloroarylation of acrolein (**8a**–**g** → **9a**–**g**, Scheme 3). α-Chloroaldehydes **9a**–**g** were extracted with dichloromethane from the reaction mixtures, distilled under reduced pressure (1–2 torr) [36], and immediately reacted with potassium thiocyanate in ethanol. It should be noted that the α-thiocyanatopropanals **4B** obtained in this way are unstable and cannot be isolated in an individual state, so we used them in situ for the synthesis of 5-arylmethyl-2-mercaptothiazoles **5B** under the optimized conditions described above. Obviously, the instability of compounds **4B** complicated their synthesis by direct thiocyanatoarylation (**2** → **4B**).

It should be noted that the obtained 2-mercaptothiazoles can exist in either the thiol (enethiol) or thione (thioamide) form. According to NMR spectroscopy data, the majority of the compounds **5** obtained are in the thione form (see Table 2), at least for solutions in DMSO or in CDCl_3_ (see ^1^H NMR **5Bd**). This was confirmed by the NH group’s proton signals at 12.93–13.05 ppm in ^1^H NMR and the signals of carbon of the C=S group at 186.3–188.3 ppm in ^13^C NMR. Only the compounds **5Ba** and **5Be** were exclusively in the thiol form under the NMR measurement conditions applied (SH signal 9.5–10.9 ppm and absence of the C=S signal in the ^13^C NMR spectra). For compounds **5Ab**, **5Ac**, **5Bd**, and **5Bf**, a small amount (6–10%) of the thiol form was observed in the equilibrium of mixtures of tautomers according to the NMR spectra. The IR spectra show signals from both the thiol (SH) and thione (NH and C=S) forms of compounds **5**. However, although the thione form predominates, we have depicted compound **5** in the thiol form, as it is this form that undergoes the transformations we have investigated.

**Scheme 4:**
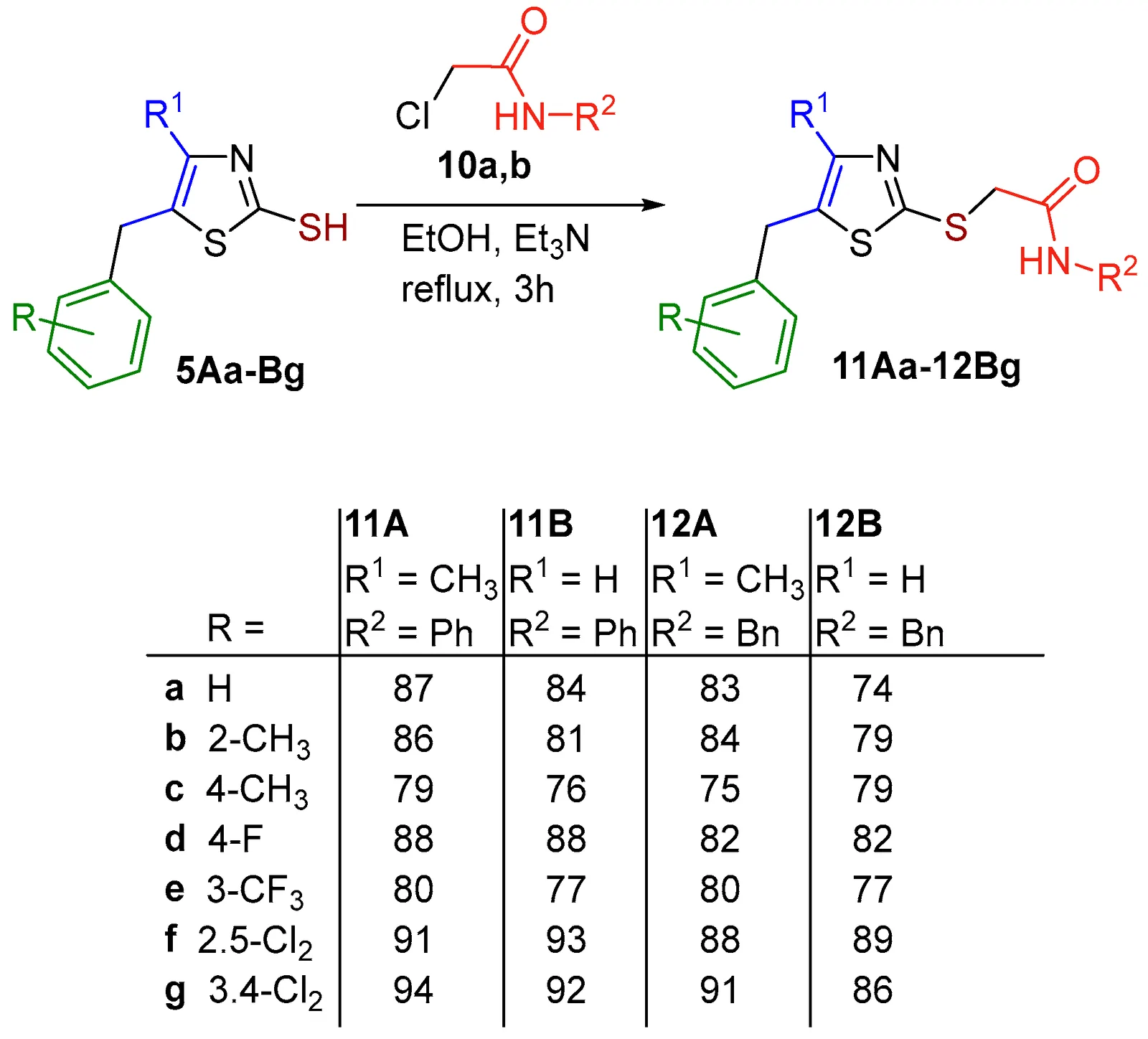
Gram-scale synthesis of *N*-substituted 2-((5-(*R*-benzyl)-4-*R*^1^-thiazol-2-yl)thio)acetamides **11Aa–12Bg** (Method A). Yields are given in %.

We investigated the possibility of synthesizing 2-*S*-substituted derivatives of 5-arylmethyl-4-*R*-2-mercaptothiazoles first by modification of the sulfur atom in position 2 of the thiazole ring of the mercaptothiazoles **5A**,**B**. It was found that the applied base had a significant effect on the yields of the S-alkylation products of the mercaptothiazoles. We investigated the reaction in the presence of potassium hydroxide in ethanol, sodium ethanolate in anhydrous alcohol, and triethylamine as the base. In the case of potassium hydroxide or sodium ethanolate, the target products were obtained in moderate yields (see Table 3, entry 1 and 2). When these bases are used, significant resinification is observed due to side reactions, in particular, the possible opening of the thiazole ring in the thione form. The reaction of **5** with chloroacetamides **10a**,**b** in the presence of triethylamine as a base yielded the amides **11**–**12** in the very good yields (Scheme 4; Table 3, entry 3). It should be noted that the use of higher-boiling solvents (dioxane or propanol) results in a lower yield of product **11Aa** (Table 3, entry 5 and 6), whilst the use of a low-boiling solvent (methanol) prolongs the reaction time (Table 3, entry 4).

**Scheme 5:**
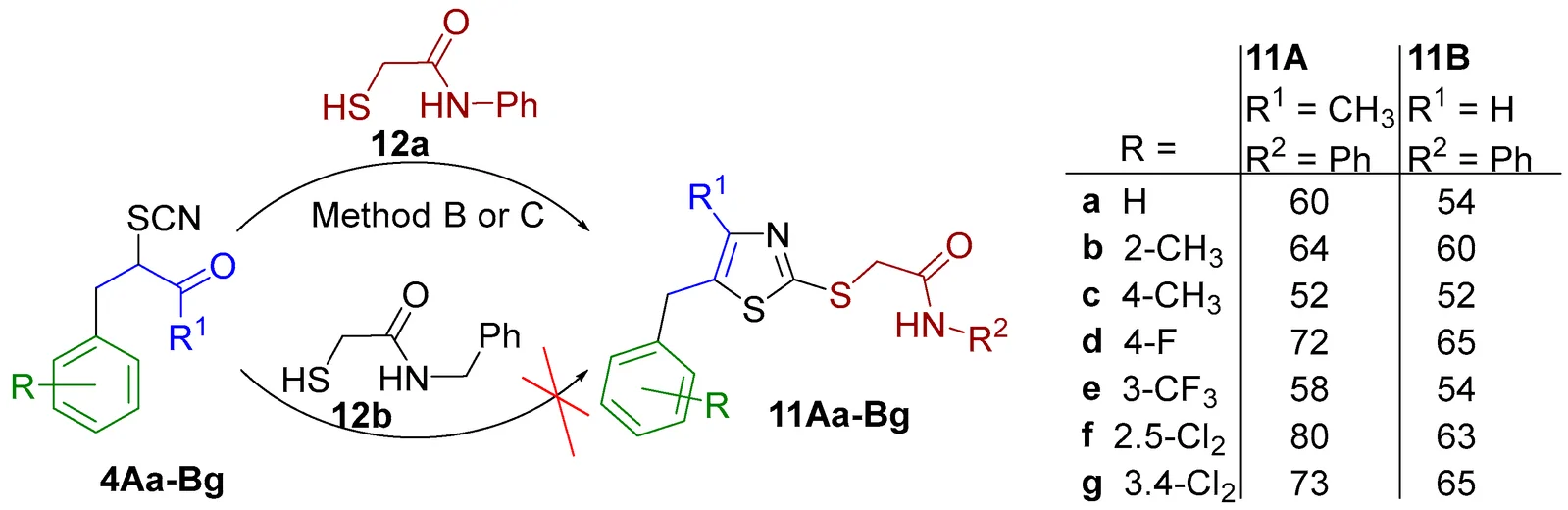
Synthesis of *N*-phenyl-2-((5-(*R*-benzyl)-4-*R*^1^-thiazol-2-yl)thio)acetamides **11** (Methods B and C). Yields are given in %.

Secondly, we subjected α-thiocyanatocarbonyl compounds **4** to a reaction with mercaptoacetamides **12a,b** (Scheme 5). We found that the reaction of thiocyanatoketones **4A** with mercaptoacetanilide **12a** in glacial acetic acid under reflux proceeded to form amides **11A** in good yields. The reaction of thiocyanate aldehydes **4b** proceeded similarly, but the amides **11B** were formed in lower yields. Unfortunately, the reaction of thiocyanatocarbonyl compounds **4** with mercaptoacetamide **12b** did not occur.

It can therefore be concluded that although the formation of a 2-mercaptothiazole fragment from α-thiocyanatocarbonyl compounds is possible upon interaction with various SH-containing compounds, the structure of the thiols has a decisive influence on the result of such a reaction.

In conclusion, we have developed a cyclization of α-thiocyanatocarbonyl compounds obtained from arenediazonium salts by reaction with SH-containing compounds to access a wide range of 4,5-substituted thiazole-2-thioles in good yields. The possibility of an effective modification of the SH group of such compounds is also demonstrated. A wide range of available starting materials, including both anilines and α,β-unsaturated carbonyl compounds, allowed us to achieve a structural variety at positions 4 and 5 of the thiazole core, which are difficult to prepare by other routes. Thus, our method represents a facile and mild procedure toward substituted 5-arylmethyl-4-*R*-1,3-thiazole-2-thioles.

## 3. Materials and Methods

Commercially available reagents and solvents were obtained from TCI (TCI, Haven 1063, 2070 Zwijndrecht, Belgium), J&K (J&K, Marbach, Germany), and Aldrich (Merck KGaA, Darmstadt, Germany) and used without further purification unless otherwise specified.

Reported yields are isolated and have not been optimized. Melting points were determined using a Büchi apparatus (Büchi, Uster, Switzerland) and are reported uncorrected.

Thin-layer chromatography (TLC) was carried out on Merck (Merck KGaA, Darmstadt, Germany) silica gel 60 F_254_ aluminum plates, with visualization under UV light.

^1^H and ^13^C NMR spectra were obtained on a Bruker 170 Avance 500 spectrometer (at 500 MHz for ^1^H, 126 MHz for ^13^C), and a Bruker Avance 400 MHz spectrometer (at 400 MHz for ^1^H, 101 MHz for ^13^C) (Bruker, Bremen, Germany). Chemical shifts (δ) are reported in parts per million (ppm) relative to the internal standard or solvent signal. Signal multiplicities are denoted as follows: s = singlet, br.s = broad singlet, d = doublet, dd = doublet of doublets, t = triplet, q = quartet, m = multiplet. Signal orientations in DEPT experiments are indicated as: o = no signal; + = positive (CH, CH_3_); − = negative (CH_2_).

Electrospray ionization mass spectra (ESI-MS) were recorded on a Bruker Impact II mass spectrometer using MeCN as the solvent.

ATR-IR spectra were recorded on a Shimadzu IRSpirit-T spectrometer (Shimadzu, Kyoto, Japan) over the range of 400–4000 cm^−1^.

**General Procedure for the Preparation of the 4-Aryl-3-thiocyanatobutan-2-ones (4A)**

The corresponding aniline **1** (70 mmol, 1.0 equiv.) was added to 37% aqueous HCl (13.1 mL, 154 mmol, 2.2 equiv.). The mixture was cooled to −5 °C, and 5.3 g (77 mmol, 1.1 equiv.) of a saturated solution of sodium nitrite was added dropwise at such a rate that the temperature did not exceed 5 °C. After that, 40% solution of HBF_4_ (14.3 mL, 84 mmol, 1.2 equiv.) at 0 °C was added to the mixture. The precipitate was filtered off, washed with EtOH (2 × 5 mL), then Et_2_O (2 × 5 mL). The aryldiazonium tetrafluoroborates were processed without further purification. Freshly-prepared diazonium tetrafluoroborate **2** (50 mmol, 1.0 equiv.) was added portionwise under vigorous stirring to a mixture of Cu(AcO)_2_ × H_2_O (50 mg, 0.25 mmol, 0.5 mol%), NH_4_SCN (3.8 g, 50 mmol, 1.0 equiv.), methyl vinyl ketone (5.2 mL, 50 mmol, 1.0 equiv.), water (30 mL), and acetone (30 mL). The rate of the addition was selected so that nitrogen evolved at a rate of 2–3 bubbles per second (addition time 0.5–1 h). Then, the mixture was stirred until nitrogen no longer evolved; 100 mL of water was added, and the organic layer was separated and triturated with hexane. Crystals were filtered off and recrystallized from benzene-hexane. If the organic layer did not crystallize after trituration with hexane, the aqueous layer was extracted with dichloromethane (3 × 15 mL). The combined extract was dried over MgSO_4_, evaporated, and the residue was distilled under reduced pressure.

Caution. Aryldiazonium tetrafluoroborates pose severe explosion hazards from thermal decomposition and friction in a dry state. However, they are stable when stored at room temperature. Ammonium thiocyanate is harmful if swallowed, inhaled, or absorbed through the skin, and causes severe eye damage or irritation.

**4-Phenyl-3-thiocyanatobutan-2-one (4Aa)**

Yield 3.591 g (35%), cream-colored crystalline solid, bp 130–135 °C/0.5 torr, m.p. 75–76 °C. ^1^H NMR (500 MHz, CDCl_3_): δ = 7.37–7.27 (m, 3H, C_6_H_5_), 7.22 (d, *J* = 7.1 Hz, 2H, C_6_H_5_), 4.03 (t, *J* = 7.4 Hz, 1H, CH), 3.39 (dd, *J* = 14.4, 7.3 Hz, 1H, CH_2_), 3.18 (dd, *J* = 14.4, 7.4 Hz, 1H, CH_2_), 2.28 (s, 3H, CH_3_). ^13^C NMR (101 MHz, CDCl_3_): δ = 201.1, 135.7, 129.3, 129.1, 127.8, 109.9, 57.1, 37.1, 28.6. Spectroscopic data are in agreement with those reported in the literature [31].

**3-Thiocyanato-4-(*o*-tolyl)butan-2-one (4Ab)**

Yield 5.703 g (52%), white crystalline solid, m.p. 60–61 °C.

^1^H NMR (400 MHz, [D_6_]DMSO): δ = 7.25–7.10 (m, 4H, C_6_H_4_), 4.57 (dd, *J* = 9.2, 6.0 Hz, 1H, CH), 3.35 (dd, *J* = 14.8, 6.0 Hz, 1H, CH_2_), 2.97 (dd, *J* = 14.9, 9.2 Hz, 1H, CH_2_), 2.33 (s, 3H, CH_3_), 2.31 (s, 3H, CH_3_).

^13^C NMR (101 MHz, [D_6_]DMSO): δ = 201.7, 136.4, 134.9, 130.3, 129.7, 127.0, 125.9, 110.8, 53.8, 32.5, 27.9, 19.1.

MS (ESI): m/z = 220.1 [M+H]^+^.

IR (ATR) ν_max_ = 2152 (SCN), 1717 (C=O) cm^−1^.

Anal. Calcd. for C_12_H_13_NOS: C, 65.72; H, 5.98; N, 6.39. Found: C, 65.67; H, 5.87; N, 6.31.

**4-(4-Methylphenyl)-3-thiocyanatobutan-2-one (4Ac)**

Yield 4.490 g (41%), white crystalline solid, m.p. 64–65 °C. ^1^H NMR (400 MHz, [D_6_]DMSO): δ = 7.20–7.04 (m, 4H, C_6_H_4_), 4.59 (dd, *J* = 8.8, 5.9 Hz, 1H, CH), 3.29 (dd, *J* = 14.7, 5.8 Hz, 1H, CH_2_), 2.96 (dd, *J* = 14.7, 8.8 Hz, 1H, CH_2_), 2.31 (s, 3H, CH_3_), 2.28 (s, 3H, CH_3_). ^13^C NMR (101 MHz, [D_6_]DMSO): δ = 201.9 (o), 136.1 (o), 133.5 (o), 129.0 (+), 110.9 (o), 55.6 (+), 34.7 (-), 27.8 (+), 20.7 (+). Spectroscopic data are in agreement with those reported in the literature [31].

**4-(4-Fluorophenyl)-3-thiocyanatobutan-2-one (4Ad)**

Yield 6.707 g (60%), off-white crystalline solid, m.p. 73–74 °C. ^1^H NMR (400 MHz, [D_6_]DMSO): δ = 7.31 (dd, *J* = 8.7, 5.6 Hz, 2H, C_6_H_4_), 7.16 (t, *J* = 8.9 Hz, 2H, C_6_H_4_), 4.59 (dd, *J* = 9.1, 5.6 Hz, 1H, CH), 3.39–3.28 (m, 1H, CH_2_), 2.98 (dd, *J* = 14.7, 9.1 Hz, 1H, CH_2_), 2.33 (s, 3H, CH_3_). ^13^C NMR (101 MHz, [D_6_]DMSO): δ = 201.8 (o), 161.3 (o, d, *J* = 242.8 Hz), 132.8 (o, d, *J* = 3.0 Hz), 131.1 (+, d, *J* = 8.1 Hz), 115.2 (+, d, *J* = 21.2 Hz), 110.7 (o), 55.5 (+), 34.2 (-), 27.8 (+). Spectroscopic data are in agreement with those reported in the literature [31].

**3-Thiocyanato-4-(3-(trifluoromethyl)phenyl)butan-2-one (4Ae)**

Yield 8.880 g (65%), white crystalline solid, m.p. 45–46 °C. ^1^H NMR (400 MHz, [D_6_]DMSO): δ = 7.68 (s, 1H, C_6_H_4_), 7.65–7.62 (m, 1H, C_6_H_4_), 7.61–7.56 (m, 2H, C_6_H_4_), 4.66 (dd, *J* = 9.7, 5.3 Hz, 1H, CH), 3.46 (dd, *J* = 14.7, 5.3 Hz, 1H, CH_2_), 3.05 (dd, *J* = 14.7, 9.7 Hz, 1H, CH_2_), 2.35 (s, 3H, CH_3_). ^13^C NMR (101 MHz, [D_6_]DMSO): δ = 201.6 (o), 138.3 (o), 133.5 (+), 129.4 (+), 129.18 (o, q, *J* = 31.5 Hz), 125.74 (+, q, *J* = 3.6 Hz), 124.22 (o, q, *J* = 272.2 Hz), 123.74 (+, q, *J* = 3.7 Hz), 110.6 (o), 54.9 (+), 34.5 (-), 27.7 (+). Spectroscopic data are in agreement with those reported in the literature [31].

**4-(2,5-Dichlorophenyl)-3-thiocyanatobutan-2-one (4Af**)

Yield 11.651 g (85%), cream-colored crystalline solid, m.p. 99–100 °C.

^1^H NMR (400 MHz, [D_6_]DMSO): δ = 7.52 (d, *J* = 2.7 Hz, 1H, C_6_H_3_), 7.51 (d, *J* = 8.6 Hz, 1H, C_6_H_3_), 7.39 (dd, *J* = 8.6, 2.6 Hz, 1H, C_6_H_3_), 4.64 (dd, *J* = 10.1, 4.9 Hz, 1H, CH), 3.41 (dd, *J* = 15.1, 4.9 Hz, 1H, CH_2_), 3.13 (dd, *J* = 15.1, 10.1 Hz, 1H, CH_2_), 2.36 (s, 3H, CH_3_).

^13^C NMR (101 MHz, [D_6_]DMSO): δ = 201.2 (o), 136.6 (o), 132.4 (o), 131.7 (o), 131.1 (+), 131.0 (+), 128.8 (+), 110.6 (o), 53.7 (+), 32.4 (-), 27.6 (+).

MS (ESI): m/z = 274.0 [M+H]^+^.

IR (ATR) ν_max_ = 2155 (SCN), 1712 (C=O) cm^−1^.

Anal. Calcd. for C_11_H_9_Cl_2_NOS: C, 48.19; H, 3.31; N, 5.11. Found: C, 48.12; H, 3.22; N, 5.01.

**4-(3,4-Dichlorophenyl)-3-thiocyanatobutan-2-one (4Ag)**

Yield 11.235 g (82%), white crystalline solid, m.p. 87–88 °C.

^1^H NMR (400 MHz, [D_6_]DMSO): δ = 7.73–7.52 (m, 2H, C_6_H_3_), 7.28 (dd, *J* = 8.2, 2.0 Hz, 1H, C_6_H_3_), 4.62 (dd, *J* = 9.6, 5.3 Hz, 1H, CH), 3.37 (dd, *J* = 14.8, 5.3 Hz, 1H, CH_2_), 2.96 (dd, *J* = 14.8, 9.6 Hz, 1H, CH_2_), 2.34 (s, 1H, CH_3_).

^13^C NMR (101 MHz, [D_6_]DMSO): δ = 201.5, 138.1, 131.2, 130.9, 130.5, 129.7, 129.6, 110.6, 54.7, 33.9, 27.6.

MS (ESI): m/z = 274.0 [M+H]^+^.

IR (ATR) ν_max_ = 2152 (SCN), 1708 (C=O) cm^−1^.

Anal. Calcd. for C_11_H_9_Cl_2_NOS: C, 48.19; H, 3.31; N, 5.11. Found: C, 48.09; H, 3.26; N, 5.02.

**General Procedure for the Preparation of the 5-Arylmethyl-4-methyl-2-mercaptothiazoles (** **5A)**

Mixture of the appropriate 4-aryl-3-thiocyanatobutan-2-one **4A** (30 mmol, 1.0 equiv.), thiourea (4.8 g, 63 mmol, 2.1 equiv.), 37% aqueous HCl (10 mL, 117 mmol, 3.9 equiv.), ethanol (30 mL), and water (20 mL) was refluxed under vigorous stirring on the hot plate magnetic stirrer for 6 h. After that, 30 mL water was added to the mixture. The resulting solidified product **5A** was filtered off and recrystallized from toluene or a mixture of toluene-hexane.

Caution. Thiourea is harmful if swallowed, suspected of causing cancer, teratogenic.

**5-Benzyl-4-methylthiazole-2-thiol (5Aa)**

Yield 4.915 g (74%), off-white crystalline solid, m.p. 143–144 °C.

^1^H NMR (400 MHz, [D_6_]DMSO): δ = 12.95 (s, 1H, NH), 7.31 (t, *J* = 7.3 Hz, 2H, C_6_H_5_), 7.25–7.18 (m, 3H, C_6_H_5_), 3.84 (s, 2H, CH_2_), 2.14 (s, 3H, CH_3_).

^13^C NMR (101 MHz, [D_6_]DMSO): δ = 186.4, 139.0, 134.0, 128.6, 128.2, 126.6, 122.9, 31.0, 11.2.

MS (ESI): m/z = 222.0 [M+H]^+^.

IR (ATR) ν_max_ = 3024 (NH), 2866 (SH), 1624 (C=S) cm^−1^.

Anal. Calcd. for C_11_H_11_NS_2_: C, 59.69; H, 5.01; N, 6.33. Found: C, 59.61; H, 4.95; N, 6.27.

**4-Methyl-5-(2-methylbenzyl)thiazole-2-thiol (5Ab)**

Yield 5.085 g (72%), off-white crystalline solid, m.p. 197–198 °C.

^1^H NMR (400 MHz, [D_6_]DMSO): δ = 12.94 (s, 0.93H, NH), 10.86 (s, 0.07H, SH), 7.17–7.12 (m, 3H, C_6_H_4_), 7.14–7.05 (m, 1H, C_6_H_4_), 3.82 (s, 1.86H, CH_2_), 3.73 (s, 0.14H, CH_2_), 2.23 (s, 3H, CH_3_), 2.12 (s, 2.79H, CH_3_), 2.01 (s, 0.21H, CH_3_).

^13^C NMR (101 MHz, [D_6_]DMSO): δ = 186.3, 137.1, 135.9, 133.9, 130.3, 128.6, 126.9, 126.2, 122.5, 29.1, 18.9, 11.3.

MS (ESI): m/z = 236.1 [M+H]^+^.

IR (ATR) ν_max_ = 3297 (NH), 2928 (SH), 1621 (C=S) cm^−1^.

Anal. Calcd. for C_12_H_13_NS_2_: C, 61.24; H, 5.57; N, 5.95. Found: C, 61.16; H, 5.46; N, 5.90.

**4-Methyl-5-(4-methylbenzyl)thiazole-2-thiol (5Ac)**

Yield 5.020 g (71%), cream-colored crystalline solid, m.p. 141–142 °C.

^1^H NMR (400 MHz, [D_6_]DMSO): δ = ^1^H NMR (400 MHz, DMSO-*d*_6_) δ 12.93 (s, 0.93H, NH), 10.85 (s, 0.07H, SH), 7.11 (d, *J* = 8.2 Hz, 1H, C_6_H_4_), 7.07 (d, *J* = 8.2 Hz, 1H, C_6_H_4_), 3.78 (s, 1.86H, CH_2_), 3.69 (s, 0.14H, CH_2_), 2.25 (s, 3H, CH_3_), 2.13 (s, 2.79H, CH_3_), 2.02 (s, 0.21H, CH_3_).

^13^C NMR (101 MHz, [D_6_]DMSO): δ = 186.3, 135.9, 135.7, 133.8, 129.2, 128.1, 123.3, 30.7, 20.6, 11.2.

MS (ESI): m/z = 236.1 [M+H]^+^.

IR (ATR) ν_max_ = 3032 (NH), 2865 (SH), 1625(C=S) cm^−1^.

Anal. Calcd. for C_12_H_13_NS_2_: C, 61.24; H, 5.57; N, 5.95. Found: C, 61.18; H, 5.49; N, 5.84.

**5-(4-Fluorobenzyl)-4-methylthiazole-2-thiol (5Ad)**

Yield 5.672 g (79%), beige crystalline solid, m.p. 158–159 °C.

^1^H NMR (400 MHz, [D_6_]DMSO): δ = 12.95 (s, 1H, NH), 7.24 (dd, *J* = 8.6, 5.6 Hz, 2H, C_6_H_4_), 7.12 (t, *J* = 8.9 Hz, 2H, C_6_H_4_), 3.84 (s, 2H, CH_2_), 2.13 (s, 3H, CH_3_).

^13^C NMR (101 MHz, [D_6_]DMSO): δ = 186.4, 161.0 (d, *J* = 242.6 Hz), 135.2 (d, *J* = 3.0 Hz), 134.1, 130.1 (d, *J* = 8.0 Hz), 122.8, 115.3 (d, *J* = 21.3 Hz), 30.1, 11.2.

MS (ESI): m/z = 240.0 [M+H]^+^.

IR (ATR) ν_max_ = 3030 (NH), 2869 (SH), 1618 (C=S) cm^−1^.

Anal. Calcd. for C_11_H_10_FNS_2_: C, 55.21; H, 4.21; N, 5.85. Found: C, 55.10; H, 4.13; N, 5.76.

**4-Methyl-5-(3-(trifluoromethyl)benzyl)thiazole-2-thiol (5Ae)**

Yield 6.340 g (73%), white crystalline solid, m.p. 151–152 °C.

^1^H NMR (400 MHz, [D_6_]DMSO): δ = 12.99 (s, 1H, NH), 7.63–7.56 (m, 2H, C_6_H_4_), 7.55–7.50 (m, 2H, C_6_H_4_), 3.98 (s, 2H, CH_2_), 2.15 (s, 3H, CH_3_).

^13^C NMR (101 MHz, [D_6_]DMSO): δ = 186.6, 140.6, 134.7, 132.4 (q, *J* = 1.1 Hz), 129.7, 129.30 (q, *J* = 31.6 Hz), 124.7 (q, *J* = 3.8 Hz), 124.15 (q, *J* = 272.4 Hz), 123.4 (q, *J* = 3.9 Hz), 121.9, 30.5, 11.3.

MS (ESI): m/z = 290.0 [M+H]^+^.

IR (ATR) ν_max_ = 3044 (NH), 2876 (SH), 1624 (C=S) cm^−1^.

Anal. Calcd. for C_12_H_10_F_3_NS_2_: C, 49.82; H, 3.48; N, 4.84. Found: C, 49.75; H, 3.40; N, 4.78.

**5-(2,5-Dichlorobenzyl)-4-methylthiazole-2-thiol (5Af)**

Yield 7.310 g (84%), light-yellow crystalline solid, m.p. 200–201 °C.

^1^H NMR (400 MHz, [D_6_]DMSO): δ = 12.99 (s, 1H, NH), 7.48 (d, *J* = 8.5 Hz, 1H, C_6_H_3_), 7.44 (d, *J* = 2.3 Hz, 1H, C_6_H_3_), 7.37 (dd, *J* = 8.5, 2.4 Hz, 1H, C_6_H_3_), 3.94 (s, 2H, CH_2_), 2.15 (s, 3H, CH_3_).

^13^C NMR (101 MHz, [D_6_]DMSO): δ = 186.5, 138.5, 135.0, 132.0, 131.5, 131.1, 130.2, 128.7, 120.2, 29.1, 11.4.

MS (ESI): m/z = 290.0 [M+H]^+^.

IR (ATR) ν_max_ = 3030 (NH), 2869 (SH), 1618 (C=S) cm^−1^.

Anal. Calcd. for C_11_H_9_Cl_2_NS_2_: C, 45.52; H, 3.13; N, 4.83. Found: C, 45.41; H, 3.05; N, 4.72.

**5-(3,4-Dichlorobenzyl)-4-methylthiazole-2-thiol (5Ag)**

Yield 7.411 g (85%), light-yellow crystalline solid, m.p. 201–202 °C.

^1^H NMR (400 MHz, [D_6_]DMSO): δ = 12.98 (s, 1H, NH), 7.56 (d, *J* = 8.3 Hz, 1H, C_6_H_3_), 7.49 (d, *J* = 1.8 Hz, 1H, C_6_H_3_), 7.20 (dd, *J* = 8.2, 1.9 Hz, 1H, C_6_H_3_), 3.88 (s, 2H, CH_2_), 2.13 (s, 3H, CH_3_).

^13^C NMR (101 MHz, [D_6_]DMSO): δ = 186.6, 140.3, 134.8, 131.1, 130.7, 130.2, 129.3, 128.6, 121.5, 29.9, 11.3.

MS (ESI): m/z = 290.0 [M+H]^+^.

IR (ATR) ν_max_ = 3034 (NH), 2885 (SH), 1621 (C=S) cm^−1^.

Anal. Calcd. for C_11_H_9_Cl_2_NS_2_: C, 45.52; H, 3.13; N, 4.83. Found: C, 45.40; H, 3.04; N, 4.75.

**General Procedure for the Preparation of the 5-Arylmethyl-2-mercaptothiazoles (** **5B)**

The corresponding aniline **1** (70 mmol, 1.0 equiv.) was added to 37% aqueous HCl (13.1 mL, 154 mmol, 2.2 equiv.). The mixture was cooled to −5 °C, and 5.3 g (77 mmol, 1.1 equiv.) of a saturated solution of sodium nitrite was added dropwise at such a rate that the temperature did not exceed 5 °C. A freshly-prepared solution of diazonium chloride **8** was added dropwise under vigorous stirring to a mixture of CuCl_2_ × 2H_2_O (3.4 g, 20 mmol, 28.6 mol%), acrolein (6.1 mL, 91 mmol, 1.3 equiv.), and acetone (70 mL). The rate of the addition was selected so that nitrogen evolved at a rate of 2–3 bubbles per second (addition time 0.2–0.5 h). Then, the mixture was stirred until nitrogen no longer evolved; 200 mL of water was added, the organic layer was separated, and the aqueous layer was extracted with dichloromethane (4 × 15 mL). The combined extract was dried over MgSO_4_, evaporated, and the residue was distilled under reduced pressure: 2-chloro-3-phenylpropanal **9a**, yield 5.570 g (47%), bp 80–85 °C/2 torr; 2-chloro-3-(*o*-tolyl)propanal **9b**, yield 6.810 g (53%), bp 94–98 °C/2 torr; 2-chloro-3-(*p*-tolyl)propanal **9c**, yield 5.302 g (41%), bp 99–103 °C/2 torr; 2-chloro-3-(4-fluorophenyl)propanal **9d**, yield 9.600 g (73%), bp 91–95 °C/2 torr; 2-chloro-3-(3-(trifluoromethyl)phenyl)propanal **9e**, yield 12.700 g (76%), bp 94–96 °C/2 torr; 2-chloro-3-(2,5-dichlorophenyl)propanal **9f**, yield 11.853 g (71%), bp 124–130 °C/0.5 torr; 2-chloro-3-(3,4-dichlorophenyl)propanal **9g**, yield 10.520 g (63%), bp 132–136 °C/0.5 torr.

Potassium thiocyanate (4.85 g, 50 mmol, 1.0 equiv.) was added to a solution of the freshly-prepared appropriate 3-aryl-2-chloropropanal **9** (50 mmol, 1.0 equiv.) in ethanol (50 mL), and the mixture was refluxed on the hot plate magnetic stirrer for 1 h. After that, thiourea (8.0 g, 105 mmol, 2.1 equiv.), 37% aqueous HCl (16.7 mL, 195 mmol, 3.9 equiv.), and water (30 mL) were added, and the reaction mixture was refluxed under vigorous stirring for a further 6 h. After cooling, 50 mL of water was added to the reaction mixture and the solidified product **5B** was filtered off and recrystallized from toluene or a mixture of toluene-hexane.

Caution. Aryldiazonium chlorides should be used in situ; in their pure form, they are explosive.

**5-Benzylthiazole-2-thiol (5Ba)**

Yield 7.150 g (69%), white crystalline solid, m.p. 118–119 °C.

^1^H NMR (400 MHz, [D_6_]DMSO): δ = 10.90 (s, 1H, SH), 7.35–7.27 (m, 2H, C_6_H_4_), 7.27–7.19 (m, 3H, C_6_H_4_), 6.65 (s, 1H, H-thiazole), 3.78 (s, 2H, CH_2_).

^13^C NMR (101 MHz, [D_6_]DMSO): δ = 172.7, 138.9, 128.5, 128.3, 126.6, 118.7, 117.9, 34.0.

MS (ESI): m/z = 208.0 [M+H]^+^.

IR (ATR) ν_max_ = 3034 (NH), 2869 (SH), 1618 (C=S) cm^−1^.

Anal. Calcd. for C_10_H_9_NS_2_: C, 57.94; H, 4.38; N, 6.76. Found: C, 57.88; H, 4.29; N, 6.65.

**5-(2-Methylbenzyl)thiazole-2-thiol (5Bb)**

Yield 7.630 g (69%), white crystalline solid, m.p. 179–180 °C.

^1^H NMR (400 MHz, [D_6_]DMSO): δ = 12.98 (s, 1H, NH), 7.15 (s, 4H, C_6_H_4_), 7.00 (s, 1H, H-thiazole), 3.87 (d, *J* = 1.2 Hz, 2H, CH_2_), 2.24 (s, 3H, CH_3_).

^13^C NMR (101 MHz, [D_6_]DMSO): δ = 188.0, 136.7, 135.9, 130.3, 130.1, 128.9, 127.0, 126.2, 125.5, 30.4, 18.9.

MS (ESI): m/z = 222.0 [M+H]^+^.

IR (ATR) ν_max_ = 3037 (NH), 2880 (SH), 1598 (C=S) cm^−1^.

Anal. Calcd. for C_11_H_11_NS_2_: C, 59.69; H, 5.01; N, 6.33. Found: C, 59.59; H, 4.93; N, 6.26.

**5-(4-Methylbenzyl)thiazole-2-thiol (5Bc)**

Yield 7.965 g (72%), light-yellow crystalline solid, m.p. 143–144 °C.

^1^H NMR (400 MHz, [D_6_]DMSO): δ = 12.96 (s, 1H, NH), 7.11 (s, 4H, C_6_H_4_), 7.09 (s, 1H, H-thiazole), 3.83 (s, 2H, CH_2_), 2.26 (s, 3H, CH_3_).

^13^C NMR (101 MHz, [D_6_]DMSO): δ = 188.1, 135.8, 135.7, 131.0, 129.2, 128.2, 125.4, 32.2, 20.6.

MS (ESI): m/z = 222.0 [M+H]^+^.

IR (ATR) ν_max_ = 3036 (NH), 2890 (SH), 1593 (C=S) cm^−1^.

Anal. Calcd. for C_11_H_11_NS_2_: C, 59.69; H, 5.01; N, 6.33. Found: C, 59.62; H, 4.92; N, 6.27.

**5-(4-Fluorobenzyl)thiazole-2-thiol (5Bd)**

Yield 8.563 g (76%), light yellow crystalline solid, m.p. 138–139 °C.

^1^H NMR (400 MHz, [D_6_]DMSO): δ = 12.99 (s, 0.9H, NH), 10.91 (s, 0.1H, SH), 7.28 (dd, *J* = 8.3, 5.7 Hz, 2H, C_6_H_4_), 7.19–7.11 (m, 2H, C_6_H_4_), 7.11 (s, 0.9H, H-thiazole), 6.65 (s, 0.1H, H-thiazole), 3.89 (s, 0.8H, CH_2_), 3.77 (s, 0.2H, CH_2_). ^1^H NMR (400 MHz, CDCl_3_): δ = 11.82 (s, 0.9H, NH), 10.06 (s, 0.1H, SH), 7.16 (dd, *J* = 8.4, 5.3 Hz, 2H, C_6_H_4_), 7.02 (t, *J* = 8.6 Hz, 2H, C_6_H_4_), 6.67 (s, 0.9H, H-thiazole), 6.31 (s, 0.1H, H-thiazole), 3.84 (s, 1.8H, CH_2_), 3.77 (s, 0.2H, CH_2_).

^13^C NMR (101 MHz, [D_6_]DMSO): δ = 188.2, 161.1 (d, *J* = 242.6 Hz), 134.9 (d, *J* = 3.1 Hz), 130.6 (d, *J* = 1.0 Hz), 130.2 (d, *J* = 8.1 Hz), 125.7, 115.4 (d, *J* = 21.4 Hz), 31.6. ^13^C NMR (101 MHz, CDCl_3_): δ = 189.2, 162.2 (d, *J* = 246.3 Hz), 132.97 (d, *J* = 3.3 Hz), 132.81 (d, *J* = 1.0 Hz),130.2 (d, *J* = 8.1 Hz), 123.9, 115.9 (d, *J* = 21.5 Hz), 33.1.

MS (ESI): m/z = 226.0 [M+H]^+^.

IR (ATR) ν_max_ = 3086 (NH), 2899 (SH), 1641 (C=S) cm^−1^.

Anal. Calcd. for C_10_H_8_FNS_2_: C, 53.31; H, 3.58; N, 6.22. Found: C, 53.24; H, 3.52; N, 6.11.

**5-(3-(Trifluoromethyl)benzyl)thiazole-2-thiol (5Be)**

Yield 9.220 g (67%), white crystalline solid, m.p. 203–204 °C.

^1^H NMR (400 MHz, [D_6_]DMSO): δ = 9.54 (s, 1H, SH), 7.68 (s, 1H, C_6_H_4_), 7.64–7.48 (m, 3H, C_6_H_4_), 7.20 (s, 1H, H-thiazole), 4.10 (s, 1H, CH_2_).

^13^C NMR (101 MHz, [D_6_]DMSO): δ = 169.3, 140.2, 132.6 (q, *J* = 1.4 Hz), 129.8, 129.34 (q, *J* = 31.4 Hz), 125.0 (q, *J* = 3.8 Hz), 124.16 (q, *J* = 272.4 Hz), 123.7 (q, *J* = 3.8 Hz), 123.5, 123.2, 31.28.

MS (ESI): m/z = 276.0 [M+H]^+^.

IR (ATR) ν_max_ = 3059 (NH), 2816 (SH), 1624 (C=S) cm^−1^.

Anal. Calcd. for C_11_H_8_F_3_NS_2_: C, 47.99; H, 2.93; N, 5.09. Found: C, 47.88; H, 2.60; N, 4.98.

**5-(2,5-Dichlorobenzyl)thiazole-2-thiol (5Bf)**

Yield 10.774 g (78%), cream-colored crystalline solid, m.p. 199–200 °C.

^1^H NMR (400 MHz, [D_6_]DMSO): δ = 13.05 (s, 0.94H, NH), 10.98 (s, 0.06H, SH), 7.52 (d, *J* = 2.6 Hz, 1H, C_6_H_3_), 7.48 (d, *J* = 8.5 Hz, 1H, C_6_H_3_), 7.37 (dd, *J* = 8.6, 2.6 Hz, 1H, C_6_H_3_), 7.11 (s, 0.94H, H-thiazole), 6.66 (s, 0.06H, H-thiazole), 3.99 (s, 1.88H, CH_2_), 3.88 (s, 0.12H, CH_2_).

^13^C NMR (101 MHz, [D_6_]DMSO): δ = 188.2, 138.3, 132.0, 131.5, 131.1, 130.4, 128.8, 127.6, 126.6, 30.3.

MS (ESI): m/z = 275.9 [M+H]^+^.

IR (ATR) ν_max_ = 3084 (NH), 2893 (SH), 1630 (C=S) cm^−1^.

Anal. Calcd. for C_10_H_7_Cl_2_NS_2_: C, 43.49; H, 2.55; N, 5.07. Found: C, 43.40; H, 2.45; N, 5.00.

**5-(3,4-Dichlorobenzyl)thiazole-2-thiol (5Bg)**

Yield 10.763 g (78%), off-white crystalline solid, m.p. 163–164 °C.

^1^H NMR (400 MHz, [D_6_]DMSO): δ = 13.02 (s, 1H, NH), 7.56 (d, *J* = 8.3 Hz, 1H, C_6_H_3_), 7.54–7.52 (m, 1H, C_6_H_3_), 7.24 (dd, *J* = 8.2, 1.7 Hz, 1H, C_6_H_3_), 7.15 (s, 1H, H-thiazole), 3.91 (s, 2H, CH_2_).

^13^C NMR (101 MHz, [D_6_]DMSO): δ = 188.3, 139.9, 131.1, 130.7, 130.4, 129.5, 129.2, 128.8, 126.3, 31.4.

MS (ESI): m/z = 275.9 [M+H]^+^.

IR (ATR) ν_max_ = 3069 (NH), 2878 (SH), 1597 (C=S) cm^−1^.

Anal. Calcd. for C_10_H_7_Cl_2_NS_2_: C, 43.49; H, 2.55; N, 5.07. Found: C, 43.38; H, 2.48; N, 5.02.

**Procedure for the Preparation of 5-Benzyl-4-methylthiazol-2-amine (6)**

A mixture of the corresponding 4-phenyl-3-thiocyanatobutan-2-one **4A** (10 mmol, 1.0 equiv.), thiourea (0.76 g, 10 mmol, 1.0 equiv.), and 5 mL of ethanol was stirred under reflux temperature over a period of 2 h. Next, 50 mL of hot water was added to the mixture, then the aqueous layer was separated and alkalified by a 20% solution of NH_3_ to pH ≈ 11. The mixture was allowed to cool to 20 °C whereupon a precipitate formed, which was filtered off, dried in air, and recrystallized from hexane-toluene.

**5-Benzyl-4-methylthiazol-2-amine (6)**

Yield 0.765 g, 40%, off-white crystals, m.p. 112–113 °C.

^1^H NMR (500 MHz, [D_6_]DMSO): δ = 7.29–7.26 (m, 2H, C_6_H_5_), 7.19–7.16 (m, 3H, C_6_H_5_), 6.58 (s, 2H, NH_2_), 3.84 (s, 2H, CH_2_), 2.06 (s, 3H, CH_3_) ppm.

^13^C NMR (126 MHz, [D_6_]DMSO): δ = 165.3, 142.6, 140.9, 128.4, 128.0, 126.1, 116.6, 31.4, 14.6 ppm.

MS (ESI): *m/z* = 205.1 [M+H]^+^.

Spectroscopic data are in agreement with those reported in the literature [32].

**Procedure for the Preparation of 5-Benzyl-4-methylthiazol-2(3*H*)-one (** **7)**

A solution of 4-phenyl-3-thiocyanatobutan-2-one **3** (2 mmol, 1.0 equiv.) in 96% ethanol (20 mL) was saturated with anhydrous H_2_S at ambient temperature, then 37% aqueous HCl (0.67 mL, 7.8 mmol, 3.9 equiv.) was added. The resulting mixture was stirred under reflux on a hot plate magnetic stirrer for 3 h. Then, the solvent was removed under reduced pressure, water (10 mL) was added to the residue, and the resulting solidified product **7** was filtered off. Recrystallization from toluene.

**5-Benzyl-4-methylthiazol-2(3*H*)-one** (**7)**

Yield 0.305 g (74%), white crystalline solid, m.p. 124–125 °C.

^1^H NMR (400 MHz, DMSO): δ = 10.87 (s, 1H, NH), 7.33–7.27 (m, 2H, C_6_H_5_), 7.24–7.17 (m, 3H, C_6_H_5_), 3.75 (s, 2H, CH_2_), 2.03 (s, 3H, CH_3_).

^13^C NMR (101 MHz, DMSO): δ = 171.4, 139.5, 128.5, 128.1, 126.4, 126.0, 110.9, 31.7, 11.2.

MS (ESI): m/z = 206.0 [M+H]^+^.

Spectroscopic data are in accordance with those reported in the literature [31].

**General Procedure for the Preparation of the *N*-substituted 2-((5-(*R*-Benzyl)-4-*R*^1^-thiazol-2-yl)thio)acetamides (11Aa–12Bg) (Method A)**

A mixture of the corresponding 5-arylmethyl-4-*R*-2-mercaptothiazole **5** (3 mmol, 1.0 equiv.), appropriate cloroacetamide **10** (3 mmol, 1.0 equiv.), triethylamine (0.46 mL, 3.3 mmol, 1.1 equiv.), and 10 mL of ethanol was stirred under reflux temperature over a period of 2 h. After cooling, 50 mL of cold water was added to the reaction mixture, whereupon a precipitate formed, which was filtered off and recrystallized from ethanol or a mixture of ethanol-DMF.

**General Procedure for the Preparation of the *N*-Phenyl-2-((5-(*R*-benzyl)-4-methylthiazol-2-yl)thio)acetamides (11Aa–g) (Method B)**

A mixture of the corresponding 4-aryl-3-thiocyanatobutan-2-one **4A** (3 mmol, 1.0 equiv.), 2-mercapto-*N*-phenylacetamide **12a** (0.50 g, 3 mmol, 1.0 equiv.), and 5 mL of glacial acetic acid was stirred under reflux temperature over a period of 6 h. After cooling, 50 mL of cold water was added to the reaction mixture, whereupon a precipitate formed, which was filtered off and recrystallized from ethanol or a mixture of ethanol-DMF.

**General Procedure for the Preparation of the *N*-Phenyl-2-((5-(*R*-benzyl)thiazol-2-yl)thio)acetamides (11Ba–g) (Method C)**

Potassium thiocyanate (0.294 g, 3 mmol, 1.0 equiv.) was added to a solution of the freshly-prepared appropriate 3-aryl-2-chloropropanal **9** (3 mmol, 1.0 equiv.) in ethanol (5 mL), and the mixture was refluxed on a hot plate magnetic stirrer for 1 h. After that, 5 mL of glacial acetic acid was added, and ethanol was evaporated under reduced pressure. To the obtained solution of 3-aryl-2-thiocyanatopropanal **4B** 2-mercapto-*N*-phenylacetamide **12a** (0.50 g, 3 mmol, 1.0 equiv.) was added, then the mixture was stirred under reflux temperature over a period of 6 h. After cooling, 50 mL of cold water was added to the reaction mixture, whereupon a precipitate formed, which was filtered off and recrystallized from ethanol or a mixture of ethanol-DMF.

**2-((5-Benzyl-4-methylthiazol-2-yl)thio)-*N*-phenylacetamide (11Aa)**

Yield 0.930 g (87%), off-white crystalline solid, m.p. 104–105 °C.

^1^H NMR (400 MHz, [D_6_]DMSO): δ = 10.28 (s, 1H, NH), 7.64–7.51 (m, 2H, C_6_H_5_), 7.38–7.26 (m, 4H, C_6_H_5_), 7.26–7.18 (m, 3H, C_6_H_5_), 7.06 (t, *J* = 7.4 Hz, 1H, C_6_H_5_), 4.11 (s, 2H, CH_2_), 4.05 (s, 2H, CH_2_).

^13^C NMR (101 MHz, [D_6_]DMSO): δ = 165.5, 158.9, 147.9, 139.9, 138.7, 131.6, 128.8, 128.6, 128.2, 126.5, 123.6, 119.2, 38.3, 31.3, 14.8.

MS (ESI): m/z = 355.1 [M+H]^+^.

IR (ATR) ν_max_ = 3298 (NH), 1685 (C=O) cm^−1^.

Anal. Calcd. for C_19_H_18_N_2_OS_2_: C, 64.38; H, 5.12; N, 7.90. Found: C, 64.27; H, 5.05; N, 7.80.

**2-((4-Methyl-5-(2-methylbenzyl)thiazol-2-yl)thio)-*N*-phenylacetamide (11Ab)**

Yield 0.950 g (86%), white crystalline solid, m.p. 97–98 °C.

^1^H NMR (400 MHz, [D_6_]DMSO): δ = 10.27 (s, 1H, NH), 7.53 (d, *J* = 7.7 Hz, 2H, C_6_H_5_), 7.31 (t, *J* = 7.9 Hz, 2H, C_6_H_5_), 7.17–7.11 (m, 3H, Ar), 7.10–7.03 (m, 2H, Ar), 4.09 (s, 2H, CH_2_), 4.01 (s, 2H, CH_2_), 2.30 (s, 3H, CH_3_), 2.21 (s, 3H, CH_3_).

^13^C NMR (101 MHz, [D_6_]DMSO): δ = 165.5, 158.8, 147.7, 138.7, 138.1, 135.8, 131.4, 130.3, 128.8, 128.6, 126.9, 126.2, 123.6, 119.2, 38.4, 29.5, 18.9, 14.8.

MS (ESI): m/z = 369.1 [M+H]^+^.

IR (ATR) ν_max_ = 3297 (NH), 1680 (C=O) cm^−1^.

Anal. Calcd. for C_20_H_20_N_2_OS_2_: C, 65.19; H, 5.47; N, 7.60. Found: C, 65.11; H, 5.38; N, 7.49.

**2-((4-Methyl-5-(4-methylbenzyl)thiazol-2-yl)thio)-*N*-phenylacetamide (11Ac)**

Yield 0.878 g (79%), off-white crystalline solid, m.p. 89–90 °C.

^1^H NMR (400 MHz, [D_6_]DMSO): δ = 10.28 (s, 1H, NH), 7.56 (d, *J* = 7.8 Hz, 2H, C_6_H_5_), 7.31 (t, *J* = 7.9 Hz, 2H, C_6_H_5_), 7.12–7.03 (m, 5H, C_6_H_4_, C_6_H_5_), 4.11 (s, 2H, CH_2_), 3.98 (s, 2H, CH_2_), 2.28 (s, 3H, CH_3_), 2.25 (s, 3H, CH_3_).

^13^C NMR (101 MHz, [D_6_]DMSO): δ = 165.5, 158.8, 147.7, 138.7, 136.8, 135.5, 131.9, 129.2, 128.8, 128.1, 123.5, 119.2, 38.3, 30.9, 20.6, 14.8.

MS (ESI): m/z = 369.1 [M+H]^+^.

IR (ATR) ν_max_ = 3315 (NH), 1680 (C=O) cm^−1^.

Anal. Calcd. for C_20_H_20_N_2_OS_2_: C, 65.19; H, 5.47; N, 7.60. Found: C, 65.09; H, 5.42; N, 7.50.

**2-((5-(4-Fluorobenzyl)-4-methylthiazol-2-yl)thio)-*N*-phenylacetamide (11Ad)**

Yield 0.984 g (88%), cream-colored crystalline solid, m.p. 105–106 °C.

^1^H NMR (400 MHz, [D_6_]DMSO): δ = 10.28 (s, 1H, NH), 7.55 (d, *J* = 7.6 Hz, 2H, C_6_H_5_), 7.31 (t, *J* = 7.9 Hz, 2H, C_6_H_5_), 7.23 (dd, *J* = 8.6, 5.6 Hz, 2H, C_6_H_4_), 7.11 (t, *J* = 8.9 Hz, 2H, C_6_H_3_), 7.06 (t, *J* = 7.4 Hz, 1H, C_6_H_5_), 4.11 (s, 2H, CH_2_), 4.04 (s, 2H, CH_2_), 2.29 (s, 3H, CH_3_).

^13^C NMR (101 MHz, [D_6_]DMSO): δ = 165.5, 160.9 (d, *J* = 242.2 Hz), 159.0, 147.9, 138.7, 136.1 (d, *J* = 3.1 Hz), 131.5, 130.1 (d, *J* = 8.1 Hz), 128.8, 123.6, 119.2, 115.3 (d, *J* = 21.3 Hz), 38.3, 30.5, 14.8.

MS (ESI): m/z = 373.1 [M+H]^+^.

IR (ATR) ν_max_ = 3260 (NH), 1685 (C=O) cm^−1^.

Anal. Calcd. for C_19_H_17_FN_2_OS_2_: C, 61.27; H, 4.60; N, 7.52. Found: C, 61.21; H, 4.52; N, 7.41.

**2-((4-Methyl-5-(3-(trifluoromethyl)benzyl)thiazol-2-yl)thio)-*N*-phenylacetamide (11Ae)**

Yield 1.021 g (80%), white crystalline solid, m.p. 115–116 °C.

^1^H NMR (400 MHz, [D_6_]DMSO): δ = 10.29 (s, 1H, NH), 7.60 (s, 1H, Ar), 7.59–7.52 (m, 4H, Ar), 7.50 (t, *J* = 6.1 Hz, 1H, Ar), 7.34–7.27 (m, 2H, Ar), 7.09–7.02 (m, 1H, Ar), 4.19 (s, 2H, CH_2_), 4.13 (s, 2H, CH_2_), 2.30 (s, 3H, CH_3_).

^13^C NMR (101 MHz, [D_6_]DMSO): δ = 165.5, 159.5, 148.3, 141.4, 138.7, 132.4 (q, *J* = 1.5 Hz), 130.5, 129.7, 129.3 (q, *J* = 31.4 Hz), 128.8, 124.7 (q, *J* = 3.8 Hz), 124.2 (q, *J* = 272.4 Hz), 123.5, 123.3 (q, *J* = 3.8 Hz), 119.2, 38.3, 30.8, 14.8.

MS (ESI): m/z = 423.1 [M+H]^+^.

IR (ATR) ν_max_ = 3266 (NH), 1661 (C=O) cm^−1^.

Anal. Calcd. for C_20_H_17_F_3_N_2_OS_2_: C, 56.86; H, 4.06; N, 6.63. Found: C, 56.77; H, 4.00; N, 6.54.

**2-((5-(2,5-Dichlorobenzyl)-4-methylthiazol-2-yl)thio)-*N*-phenylacetamide (11Af)**

Yield 1.160 g (91%), white crystalline solid, m.p. 114–115 °C.

^1^H NMR (400 MHz, [D_6_]DMSO): δ = 10.28 (s, 1H, NH), 7.54 (d, *J* = 7.7 Hz, 2H, C_6_H_5_), 7.48 (d, *J* = 8.5 Hz, 1H, C_6_H_3_), 7.43 (d, *J* = 2.5 Hz, 1H, C_6_H_3_), 7.36 (dd, *J* = 8.5, 2.6 Hz, 1H, C_6_H_3_), 7.30 (t, *J* = 7.9 Hz, 2H, C_6_H_5_), 7.06 (t, *J* = 7.4 Hz, 1H, C_6_H_5_), 4.14 (s, 2H, CH_2_), 4.12 (s, 2H, CH_2_), 2.31 (s, 3H, CH_3_).

^13^C NMR (101 MHz, [D_6_]DMSO): δ = 165.4, 159.7, 148.6, 139.5, 138.7, 132.0, 131.5, 131.2, 130.2, 128.9, 128.8, 128.6, 123.6, 119.2, 38.3, 29.5, 14.9.

MS (ESI): m/z = 423.0 [M+H]^+^.

IR (ATR) ν_max_ = 3317 (NH), 1670 (C=O) cm^−1^.

Anal. Calcd. for C_19_H_16_Cl_2_N_2_OS_2_: C, 53.90; H, 3.81; N, 6.62. Found: C, 53.85; H, 3.72; N, 6.55.

**2-((5-(3,4-Dichlorobenzyl)-4-methylthiazol-2-yl)thio)-*N*-phenylacetamide (11Ag)**

Yield 1.203 g (94%), cream-colored crystalline solid, m.p. 105–106 °C.

^1^H NMR (400 MHz, [D_6_]DMSO): δ = 10.28 (s, 1H, NH), 7.60–7.52 (m, 3H, Ar), 7.49 (d, *J* = 1.9 Hz, 1H, C_6_H_3_), 7.30 (t, *J* = 7.9 Hz, 2H, C_6_H_5_), 7.18 (dd, *J* = 8.3, 1.9 Hz, 1H, C_6_H_3_), 7.06 (t, *J* = 7.4 Hz, 1H, C_6_H_5_), 4.12 (s, 2H, CH_2_), 4.08 (s, 2H, CH_2_), 2.28 (s, 3H, CH_3_).

^13^C NMR (101 MHz, [D_6_]DMSO): δ = 165.5, 159.5, 148.4, 141.1, 138.7, 131.1, 130.7, 130.2, 129.2, 128.8, 128.6, 123.6, 119.2, 38.3, 30.2, 14.8.

MS (ESI): m/z = 423.0 [M+H]^+^.

IR (ATR) ν_max_ = 3266 (NH), 1662 (C=O) cm^−1^.

Anal. Calcd. for C_19_H_16_Cl_2_N_2_OS_2_: C, 53.90; H, 3.81; N, 6.62. Found: C, 53.81; H, 3.74; N, 6.54.

**2-((5-Benzylthiazol-2-yl)thio)-*N*-phenylacetamide (11Ba)**

Yield 0.861 g (84%), cream-colored crystalline solid, m.p. 96–97 °C.

^1^H NMR (400 MHz, [D_6_]DMSO): δ = 10.27 (s, 1H, NH), 7.58–7.49 (m, 3H, C_6_H_5_), 7.34–7.27 (m, 4H, C_6_H_5_), 7.26–7.20 (m, 3H, C_6_H_5_), 7.06 (t, *J* = 7.4 Hz, 1H, C_6_H_5_), 4.13 (s, 4H, 2CH_2_).

^13^C NMR (101 MHz, [D_6_]DMSO): δ = 165.5, 161.8, 140.0, 139.7, 139.3, 138.7, 128.8, 128.6, 128.3, 126.6, 123.5, 119.1, 38.2, 32.1.

MS (ESI): m/z = 341.1 [M+H]^+^.

IR (ATR) ν_max_ = 3294 (NH), 1681 (C=O) cm^−1^.

Anal. Calcd. for C_18_H_16_N_2_OS_2_: C, 63.50; H, 4.74; N, 8.23. Found: C, 63.39; H, 4.68; N, 8.12.

**2-((5-(2-Methylbenzyl)thiazol-2-yl)thio)-*N*-phenylacetamide (11Bb)**

Yield 0.862 g (81%), white crystalline solid, m.p. 86–87 °C.

^1^H NMR (400 MHz, [D_6_]DMSO): δ = 10.27 (s, 1H,NH), 7.55 (d, *J* = 7.7 Hz, 2H, Ar), 7.45 (s, 1H, H-thiazole), 7.30 (t, *J* = 7.9 Hz, 2H, Ar), 7.20–7.12 (m, 4H, Ar), 7.06 (t, *J* = 7.4 Hz, 1H, Ar), 4.12 (s, 2H, CH_2_), 4.11 (s, 2H, CH_2_), 2.24 (s, 3H, CH_3_).

^13^C NMR (101 MHz, [D_6_]DMSO): δ = 165.5, 161.4, 139.9, 138.8, 138.7, 137.8, 135.8, 130.3, 128.9, 128.8, 126.9, 126.2, 123.6, 119.2, 38.3, 30.0, 18.9.

MS (ESI): m/z = 355.1 [M+H]^+^.

IR (ATR) ν_max_ = 3295 (NH), 1681 (C=O) cm^−1^.

Anal. Calcd. for C_19_H_18_N_2_OS_2_: C, 64.38; H, 5.12; N, 7.90. Found: C, 64.30; H, 5.06; N, 7.79.

**2-((5-(4-Methylbenzyl)thiazol-2-yl)thio)-*N*-phenylacetamide (11Bc)**

Yield 0.815 g (76%), white crystalline solid, m.p. 84–85 °C.

^1^H NMR (400 MHz, [D_6_]DMSO): δ = 10.28 (s, 1H, NH), 7.55 (d, *J* = 7.8 Hz, 2H, C_6_H_5_), 7.49 (s, 1H, H-thiazole), 7.31 (t, *J* = 7.8 Hz, 2H, C_6_H_5_), 7.12 (d, *J* = 8.4 Hz, 2H, C_6_H_4_), 7.10 (d, *J* = 8.6 Hz, 2H, C_6_H_4_), 7.06 (t, *J* = 7.5 Hz, 1H, C_6_H_5_), 4.13 (s, 2H, CH_2_), 4.07 (s, 2H, CH_2_), 2.25 (s, 3H, CH_3_).

^13^C NMR (101 MHz, [D_6_]DMSO): δ = 165.5, 161.6, 139.9, 139.7, 138.7, 136.7, 135.7, 129.2, 128.8, 128.2, 123.5, 119.1, 38.2, 31.8, 20.6.

MS (ESI): m/z = 355.1 [M+H]^+^.

IR (ATR) ν_max_ = 3323 (NH), 1660 (C=O) cm^−1^.

Anal. Calcd. for C_19_H_18_N_2_OS_2_: C, 64.38; H, 5.12; N, 7.90. Found: 64.28; H, 5.05; N, 7.84.

**2-((5-(4-Fluorobenzyl)thiazol-2-yl)thio)-*N*-phenylacetamide (11Bd)**

Yield 0.950 g (88%), light-yellow crystalline solid, m.p. 100–101 °C.

^1^H NMR (400 MHz, [D_6_]DMSO): δ = 10.28 (s, 1H, NH), 7.55 (d, *J* = 7.8 Hz, 2H, C_6_H_5_), 7.51 (s, 1H, H-thiazole), 7.33–7.25 (m, 4H, Ar), 7.12 (t, *J* = 8.4 Hz, 2H, Ar), 7.06 (t, *J* = 7.2 Hz, 1H, Ar), 4.13 (s, 2H, CH_2_), 4.12 (s, 2H, CH_2_).

^13^C NMR (101 MHz, [D_6_]DMSO): δ = 165.5, 161.9, 161.0 (d, *J* = 242.4 Hz), 140.1, 139.2, 138.7, 135.9 (d, *J* = 3.1 Hz), 130.2 (d, *J* = 8.1 Hz), 128.8, 123.5, 119.1, 115.3 (d, *J* = 21.3 Hz), 38.2, 31.2.

MS (ESI): m/z = 359.1 [M+H]^+^.

IR (ATR) ν_max_ = 3297 (NH), 1644 (C=O) cm^−1^.

Anal. Calcd. for C_18_H_15_FN_2_OS_2_: C, 60.31; H, 4.22; N, 7.82. Found: C, 60.24; H, 4.15; N, 7.75.

***N*-Phenyl-2-((5-(3-(trifluoromethyl)benzyl)thiazol-2-yl)thio)acetamide (11Be)**

Yield 0.945 g (77%), cream-colored crystalline solid, m.p. 194–195 °C.

^1^H NMR (400 MHz, [D_6_]DMSO): δ = 10.28 (s, 1H, NH), 7.65 (s, 1H, Ar), 7.64–7.53 (m, 5H, Ar), 7.32 (t, *J* = 7.9 Hz, 2H, C_6_H_5_), 7.06 (t, *J* = 7.4 Hz, 1H, Ar), 6.88 (s, 1H, Ar), 4.53 (s, 2H, CH_2_), 3.98 (s, 2H, CH_2_).

^13^C NMR (101 MHz, [D_6_]DMSO): δ = 170.8, 165.2, 140.2, 138.6, 132.6 (q, *J* = 1.1 Hz), 129.6, 129.3 (q, *J* = 31.7 Hz), 128.8, 124.9 (q, *J* = 3.7 Hz), 124.2 (q, *J* = 272.5 Hz), 123.7, 123.51 (q, *J* = 4.3 Hz), 123.49, 119.0, 114.9, 47.2, 33.2.

MS (ESI): m/z = 409.1 [M+H]^+^.

IR (ATR) ν_max_ = 3294 (NH), 1643 (C=O) cm^−1^.

Anal. Calcd. for C_19_H_15_F_3_N_2_OS_2_: C, 55.87; H, 3.70; N, 6.86. Found: C, 55.78; H, 3.66; N, 6.76.

**2-((5-(2,5-Dichlorobenzyl)thiazol-2-yl)thio)-*N*-phenylacetamide (11Bf)**

Yield 1.142 g (93%), white crystalline solid, m.p. 73–74 °C.

^1^H NMR (400 MHz, [D_6_]DMSO): δ = 10.28 (s, 1H, NH), 7.58–7.52 (m, 4H, Ar), 7.48 (d, *J* = 8.6 Hz, 1H, Ar), 7.37 (dd, *J* = 8.5, 2.4 Hz, 1H, C_6_H_3_), 7.30 (t, *J* = 7.8 Hz, 2H, Ar), 7.06 (t, *J* = 7.4 Hz, 1H, Ar), 4.23 (s, 2H, CH_2_), 4.14 (s, 2H, CH_2_).

^13^C NMR (101 MHz, [D_6_]DMSO): δ = 165.4, 162.3, 140.8, 139.3, 138.7, 136.3, 132.0, 131.5, 131.2, 130.5, 128.8, 128.7, 123.6, 119.2, 38.2, 29.8.

MS (ESI): m/z = 409.0 [M+H]^+^.

IR (ATR) ν_max_ = 3303 (NH), 1681 (C=O) cm^−1^.

Anal. Calcd. for C_18_H_14_Cl_2_N_2_OS_2_: C, 52.82; H, 3.45; N, 6.84. Found: C, 52.74; H, 3.40; N, 6.73.

**2-((5-(3,4-Dichlorobenzyl)thiazol-2-yl)thio)-*N*-phenylacetamide (11Bg)**

Yield 1.132 g (92%), white crystalline solid, m.p. 108–109 °C.

^1^H NMR (400 MHz, [D_6_]DMSO): δ = 10.28 (s, 1H, NH), 7.58–7.49 (m, 5H, Ar), 7.30 (t, *J* = 7.9 Hz, 2H, C_6_H_5_), 7.24 (dd, *J* = 8.3, 2.0 Hz, 1H, C_6_H_3_), 7.05 (t, *J* = 7.4 Hz, 1H, Ar), 4.15 (s, 2H, CH_2_), 4.14 (s, 2H, CH_2_).

^13^C NMR (101 MHz, [D_6_]DMSO): δ = 165.4, 162.3, 140.9, 140.5, 138.7, 137.9, 131.1, 130.7, 130.3, 129.3, 128.8, 123.5, 119.1, 38.2, 30.9.

MS (ESI): m/z = 409.0 [M+H]^+^.

IR (ATR) ν_max_ = 3308 (NH), 1658 (C=O) cm^−1^.

Anal. Calcd. for C_18_H_14_Cl_2_N_2_OS_2_: C, 52.82; H, 3.45; N, 6.84. Found: C, 52.72; H, 3.38; N, 6.75.

***N*-Benzyl-2-((5-benzyl-4-methylthiazol-2-yl)thio)acetamide (12Aa)**

Yield 0.920 g (83%), cream-colored crystalline solid, m.p. 94–95 °C.

^1^H NMR (400 MHz, [D_6_]DMSO): δ = 8.67 (t, *J* = 6.0 Hz, 1H, NH), 7.36–7.14 (m, 10H, 2C_6_H_5_), 4.29 (d, *J* = 5.9 Hz, 2H, NCH_2_), 4.05 (s, 2H, CH_2_), 3.94 (s, 2H, CH_2_), 2.29 (s, 3H, CH_3_).

^13^C NMR (101 MHz, [D_6_]DMSO): δ = 166.7, 159.1, 147.8, 139.9, 138.9, 131.4, 128.6, 128.2, 128.2, 127.1, 126.8, 126.5, 42.5, 37.0, 31.4, 14.7.

MS (ESI): m/z = 369.1 [M+H]^+^.

IR (ATR) ν_max_ = 3301 (NH), 1643 (C=O) cm^−1^.

Anal. Calcd. for C_20_H_20_N_2_OS_2_: C, 65.19; H, 5.47; N, 7.60. Found: C, 65.08; H, 5.40; N, 7.52.

***N*-Benzyl-2-((4-methyl-5-(2-methylbenzyl)thiazol-2-yl)thio)acetamide (12Ab)**

Yield 0.970 g (84%), cream-colored crystalline solid, m.p. 116–117 °C.

^1^H NMR (400 MHz, [D_6_]DMSO): δ = 8.66 (t, *J* = 5.9 Hz, 1H, NH), 7.33–7.26 (m, 2H, Ar), 7.26–7.19 (m, 3H, Ar), 7.18–7.12 (m, 3H, Ar), 7.14–7.05 (m, 1H, Ar), 4.28 (d, *J* = 5.8 Hz, 2H, NCH_2_), 4.01 (s, 2H, CH_2_), 3.93 (s, 2H, CH_2_), 2.29 (s, 3H, CH_3_), 2.23 (s, 3H, CH_3_).

^13^C NMR (101 MHz, [D_6_]DMSO): δ = 166.7, 158.9, 147.7, 138.9, 138.1, 135.8, 131.1, 130.3, 128.6, 128.2, 127.1, 126.8, 126.8, 126.2, 42.5, 37.0, 29.5, 18.9, 14.8.

MS (ESI): m/z = 383.1 [M+H]^+^.

IR (ATR) ν_max_ = 3377 (NH), 1633 (C=O) cm^−1^.

Anal. Calcd. for C_21_H_22_N_2_OS_2_: C, 65.94; H, 5.80; N, 7.32. Found: C, 65.88; H, 5.70; N, 7.23.

**N-Benzyl-2-((4-methyl-5-(4-methylbenzyl)thiazol-2-yl)thio)acetamide (12Ac)**

Yield 0.871 g (75%), white crystalline solid, m.p. 115–116 °C.

^1^H NMR (400 MHz, [D_6_]DMSO): δ = 8.67 (t, *J* = 5.5 Hz, 1H, NH), 7.31–7.25 (m, 2H, C_6_H_5_), 7.25–7.18 (m, 3H, C_6_H_5_), 7.11 (d, *J* = 8.4 Hz, 2H, C_6_H_4_), 7.08 (d, *J* = 8.4 Hz, 2H, C_6_H_4_), 4.28 (d, *J* = 5.9 Hz, 2H, CH_2_), 3.99 (s, 2H, CH_2_), 3.93 (s, 2H, CH_2_), 2.28 (s, 3H, CH_3_), 2.26 (s, 3H, CH_3_).

^13^C NMR (101 MHz, [D_6_]DMSO): δ = 166.7, 158.9, 147.7, 138.9, 136.9, 135.6, 131.8, 129.2, 128.2, 128.1, 127.1, 126.8, 42.5, 37.1, 31.0, 20.6, 14.7.

MS (ESI): m/z = 383.1 [M+H]^+^.

IR (ATR) ν_max_ = 3271 (NH), 1631 (C=O) cm^−1^.

Anal. Calcd. for C_21_H_22_N_2_OS_2_: C, 65.94; H, 5.80; N, 7.32. Found: C, 65.83; H, 5.72; N, 7.24.

***N*-Benzyl-2-((5-(4-fluorobenzyl)-4-methylthiazol-2-yl)thio)acetamide (12Ad)**

Yield 0.952 g (82%), beige crystalline solid, m.p. 104–105 °C.

^1^H NMR (400 MHz, [D_6_]DMSO): δ = 8.67 (t, *J* = 5.8 Hz, 1H, NH), 7.35–7.17 (m, 7H, Ar), 7.12 (t, *J* = 8.9 Hz, 2H, Ar), 4.28 (d, *J* = 5.9 Hz, 2H, CH_2_), 4.05 (s, 2H, CH_2_), 3.94 (s, 2H, CH_2_), 2.28 (s, 3H, CH_3_).

^13^C NMR (101 MHz, [D_6_]DMSO): δ = 166.7, 160.9 (d, *J* = 242.3 Hz), 159.2, 147.9, 138.9, 136.1 (d, *J* = 3.1 Hz), 131.3, 130.1 (d, *J* = 8.2 Hz), 128.2, 127.1, 126.8, 115.3 (d, *J* = 21.2 Hz), 42.5, 37.0, 30.5, 14.7.

MS (ESI): m/z = 387.1 [M+H]^+^.

IR (ATR) ν_max_ = 3271 (NH), 1631 (C=O) cm^−1^.

Anal. Calcd. for C_20_H_19_FN_2_OS_2_: C, 62.15; H, 4.96; N, 7.25. Found: C, 62.04; H, 4.89; N, 7.16.

***N*-Benzyl-2-((4-methyl-5-(3-(trifluoromethyl)benzyl)thiazol-2-yl)thio)acetamide (12Ae)**

Yield 1.060 g (80%), cream-colored crystalline solid, m.p. 105–106 °C.

^1^H NMR (400 MHz, [D_6_]DMSO): δ = 8.67 (t, *J* = 5.9 Hz, 1H, NH), 7.67–7.47 (m, 4H, Ar), 7.36–7.15 (m, 5H, Ar), 4.28 (d, *J* = 5.8 Hz, 2H, NCH_2_), 4.19 (s, 2H, CH_2_), 3.95 (s, 2H, CH_2_), 2.30 (s, 3H, CH_3_).

^13^C NMR (101 MHz, [D_6_]DMSO): δ = 166.6, 159.6, 148.3, 141.4, 138.9, 132.4, 130.3, 129.7, 129.3 (q, *J* = 31.4 Hz), 128.2, 127.1, 126.8, 124.7 (q, *J* = 3.8 Hz), 124.15 (d, *J* = 272.3 Hz), 123.3 (q, *J* = 4.1 Hz), 42.5, 37.0, 30.8, 14.7.

MS (ESI): m/z = 437.1 [M+H]^+^.

IR (ATR) ν_max_ = 3273 (NH), 1633 (C=O) cm^−1^.

Anal. Calcd. for C_21_H_19_F_3_N_2_OS_2_: C, 57.78; H, 4.39; N, 6.42. Found: C, 57.70; H, 4.29; N, 6.31.

***N*-Benzyl-2-((5-(2,5-dichlorobenzyl)-4-methylthiazol-2-yl)thio)acetamide (12Af)**

Yield 1.162 g (88%), cream-colored crystalline solid, m.p. 104–105 °C.

^1^H NMR (400 MHz, [D_6_]DMSO): δ = 8.68 (t, *J* = 5.6 Hz, 1H, NH), 7.50 (d, *J* = 8.5 Hz, 1H, C_6_H_3_), 7.43 (d, *J* = 2.4 Hz, 1H, C_6_H_3_), 7.38 (dd, *J* = 8.5, 2.5 Hz, 1H, C_6_H_3_), 7.32–7.20 (m, 5H, C_6_H_5_), 4.29 (d, *J* = 5.9 Hz, 2H, CH_2_), 4.15 (s, 2H, CH_2_), 3.95 (s, 2H, CH_2_), 2.31 (s, 3H, CH_3_).

^13^C NMR (101 MHz, [D_6_]DMSO): δ = 166.6, 159.8, 148.5, 139.5, 138.9, 131.9, 131.5, 131.2, 130.2, 128.7, 128.6, 128.2, 127.1, 126.8, 42.5, 36.9, 29.4, 14.9.

MS (ESI): m/z = 437.1 [M+H]^+^.

IR (ATR) ν_max_ = 3254 (NH), 1648 (C=O) cm^−1^.

Anal. Calcd. for C_20_H_18_Cl_2_N_2_OS_2_: C, 54.92; H, 4.15; N, 6.40. Found: C, 54.85; H, 4.06; N, 6.33.

***N*-Benzyl-2-((5-(3,4-dichlorobenzyl)-4-methylthiazol-2-yl)thio)acetamide (12Ag)**

Yield 1.205 g (91%), beige crystalline solid, m.p. 99–100 °C.

^1^H NMR (400 MHz, [D_6_]DMSO): δ = 8.67 (t, *J* = 5.6 Hz, 1H, NH), 7.56 (d, *J* = 8.3 Hz, 1H, 5H-C_6_H_3_), 7.49 (d, *J* = 1.7 Hz, 1H, 2H-C_6_H_3_), 7.33–7.13 (m, 5H, C_6_H_3_, C_6_H_5_), 4.28 (d, *J* = 5.9 Hz, 2H, NCH_2_), 4.09 (s, 2H, CH_2_), 3.95 (s, 2H, CH_2_), 2.28 (s, 3H, CH_3_).

^13^C NMR (101 MHz, [D_6_]DMSO): δ = 166.6, 159.7, 148.4, 141.1, 138.9, 131.1, 130.7, 130.2, 129.9, 129.2, 128.7, 128.2, 127.1, 126.8, 42.5, 37.0, 30.2, 14.7.

MS (ESI): m/z = 437.1 [M+H]^+^.

IR (ATR) ν_max_ = 3280 (NH), 1633 (C=O) cm^−1^.

Anal. Calcd. for C_20_H_18_Cl_2_N_2_OS_2_: C, 54.92; H, 4.15; N, 6.40. Found: C, 54.82; H, 4.08; N, 6.30.

***N*-Benzyl-2-((5-benzylthiazol-2-yl)thio)acetamide (12Ba)**

Yield 0.795 g (74%), white crystalline solid, m.p. 180–181 °C.

^1^H NMR (400 MHz, [D_6_]DMSO): δ = ^1^H NMR (400 MHz, DMSO-*d*_6_) δ 8.64 (t, *J* = 5.7 Hz, 1H, NH), 7.44–7.29 (m, 4H, C_6_H_5_), 7.30–7.19 (m, 6H, C_6_H_5_), 6.78 (s, 1H, H-thiazole), 4.36 (s, 2H, CH_2_), 4.30 (d, *J* = 5.8 Hz, 2H, NCH_2_), 3.82 (s, 2H, CH_2_).

^13^C NMR (101 MHz, [D_6_]DMSO): δ = 170.7, 166.5, 138.9, 138.6, 128.5, 128.4, 128.3, 127.3, 126.9, 126.7, 123.0, 115.8, 46.6, 42.2, 33.7.

MS (ESI): m/z = 355.1 [M+H]^+^.

IR (ATR) ν_max_ = 3284 (NH), 1654 (C=O) cm^−1^.

Anal. Calcd. for C_19_H_18_N_2_OS_2_: C, 64.38; H, 5.12; N, 7.90. Found: C, 64.29; H, 5.08; N, 7.81.

***N*-Benzyl-2-((5-(2-methylbenzyl)thiazol-2-yl)thio)acetamide (12Bb)**

Yield 0.882 g (79%), cream-colored crystalline solid, m.p. 93–94 °C.

^1^H NMR (400 MHz, [D_6_]DMSO): δ = 8.68 (s, 1H, NH), 7.44 (s, 1H, H-thiazole), 7.31–7.13 (m, 9H), 4.28 (d, *J* = 5.5 Hz, 2H, CH_2_), 4.11 (s, 2H, CH_2_), 3.96 (s, 2H, CH_2_), 2.25 (s, 3H, CH_3_).

^13^C NMR (101 MHz, [D_6_]DMSO): δ = 166.7, 161.6, 139.9, 138.9, 138.6, 137.8, 135.8, 130.3, 128.9, 128.2, 127.1, 126.9, 126.8, 126.2, 42.5, 37.1, 30.1, 18.9.

MS (ESI): m/z = 369.1 [M+H]^+^.

IR (ATR) ν_max_ = 3315 (NH), 1662 (C=O) cm^−1^.

Anal. Calcd. for C_20_H_20_N_2_OS_2_: C, 65.19; H, 5.47; N, 7.60. Found: C, 65.12; H, 5.38; N, 7.53.

***N*-Benzyl-2-((5-(4-methylbenzyl)thiazol-2-yl)thio)acetamide (12Bc)**

Yield 0.875g (79%), white crystalline solid, m.p. 100–101 °C.

^1^H NMR (400 MHz, [D_6_]DMSO): δ = 8.68 (t, *J* = 6.0 Hz, 1H, NH), 7.49 (s, 1H, H-thiazole), 7.32–7.26 (m, 2H, C_6_H_5_), 7.27–7.18 (m, 3H, C_6_H_5_), 7.16–7.09 (m, 4H, C_6_H_4_), 4.29 (d, *J* = 5.9 Hz, 2H, NCH_2_), 4.07 (s, 2H, CH_2_), 3.96 (s, 2H, CH_2_), 2.26 (s, 3H, CH_3_).

^13^C NMR (101 MHz, [D_6_]DMSO): δ = 166.7, 161.8, 139.8, 139.5, 138.9, 136.7, 135.7, 129.2, 128.20, 128.20, 127.1, 126.8, 42.5, 37.1, 31.8, 20.6.

MS (ESI): m/z = 369.1 [M+H]^+^.

IR (ATR) ν_max_ = 3323 (NH), 1660 (C=O) cm^−1^.

Anal. Calcd. for C_20_H_20_N_2_OS_2_: C, 65.19; H, 5.47; N, 7.60. Found: C, 65.09; H, 5.42; N, 7.51.

***N*-Benzyl-2-((5-(4-fluorobenzyl)thiazol-2-yl)thio)acetamide (12Bd)**

Yield 0.920 g (82%), white crystalline solid, m.p. 106–107 °C.

^1^H NMR (400 MHz, [D_6_]DMSO): δ = 8.67 (t, *J* = 5.5 Hz, 1H, NH), 7.51 (s, 1H, H-thiazole), 7.33–7.23 (m, 4H, Ar), 7.25–7.19 (m, 3H, Ar), 7.13 (t, *J* = 8.8 Hz, 2H, Ar), 4.28 (d, *J* = 5.9 Hz, 2H, CH_2_), 4.13 (s, 2H, CH_2_), 3.96 (s, 2H, CH_2_).

^13^C NMR (101 MHz, [D_6_]DMSO): δ = 166.7, 162.1, 161.0 (d, *J* = 242.5 Hz), 140.0, 139.1, 138.9, 135.9 (d, *J* = 3.3 Hz), 130.2 (d, *J* = 8.1 Hz), 128.2, 127.2, 126.8, 115.3 (d, *J* = 21.3 Hz), 42.5, 37.0, 31.2.

MS (ESI): m/z = 373.1 [M+H]^+^.

IR (ATR) ν_max_ = 3325 (NH), 1658 (C=O) cm^−1^.

Anal. Calcd. for C_19_H_17_FN_2_OS_2_: C, 61.27; H, 4.60; N, 7.52. Found: C, 61.19; H, 4.52; N, 7.42.

***N*-Benzyl-2-((5-(3-(trifluoromethyl)benzyl)thiazol-2-yl)thio)acetamide (12Be)**

Yield 0.976 g (77%), light-yellow crystalline solid, m.p. 153–154 °C.

^1^H NMR (400 MHz, [D_6_]DMSO): δ = 8.65 (t, *J* = 6.0 Hz, 1H, NH), 7.67–7.59 (m, 2H, Ar), 7.59–7.55 (m, 2H, Ar), 7.37–7.29 (m, 2H, Ar), 7.29–7.20 (m, 3H, Ar), 6.84 (s, 1H, H-thiazole), 4.37 (s, 2H, CH_2_), 4.30 (d, *J* = 5.9 Hz, 2H, NCH_2_), 3.96 (s, 2H, CH_2_).

^13^C NMR (101 MHz, [D_6_]DMSO): δ = 170.7, 166.4, 140.2, 138.9, 132.6, 129.6, 129.2 (q, *J* = 32.1 Hz), 128.3, 127.3, 126.9, 124.9 (q, *J* = 3.6 Hz), 124.2 (q, *J* = 273.4 Hz), 123.6, 123.5 (q, *J* = 4.1 Hz), 114.8, 46.6, 42.2, 33.2.

MS (ESI): m/z = 423.1 [M+H]^+^.

IR (ATR) ν_max_ = 3278 (NH), 1633 (C=O) cm^−1^.

Anal. Calcd. for C_20_H_17_F_3_N_2_OS_2_: C, 56.86; H, 4.06; N, 6.63. Found: C, 56.77; H, 3.99; N, 5.54.

***N*-Benzyl-2-((5-(2,5-dichlorobenzyl)thiazol-2-yl)thio)acetamide (12Bf)**

Yield 1.135 g (89%), white crystalline solid, m.p. 90–91 °C.

^1^H NMR (400 MHz, [D_6_]DMSO): δ = 9.06–8.47 (m, 1H, NH), 7.60–7.46 (m, 3H, Ar), 7.41–7.34 (m, 1H, Ar), 7.33–7.17 (m, 5H, Ar), 4.28 (d, *J* = 5.7 Hz, 2H, NCH_2_), 4.24 (s, 2H, CH_2_), 3.98 (s, 2H, CH_2_).

^13^C NMR (101 MHz, [D_6_]DMSO): δ = 166.6, 162.4, 140.8, 139.3, 138.9, 136.1, 132.0, 131.5, 131.1, 130.5, 128.7, 128.2, 127.1, 126.8, 42.5, 37.0, 29.8.

MS (ESI): m/z = 423.1 [M+H]^+^.

IR (ATR) ν_max_ = 3268 (NH), 1637 (C=O) cm^−1^.

Anal. Calcd. for C_19_H_16_Cl_2_N_2_OS_2_: C, 53.90; H, 3.81; N, 6.62. Found: C, 53.83; H, 3.71; N, 5.54.

***N*-Benzyl-2-((5-(3,4-dichlorobenzyl)thiazol-2-yl)thio)acetamide (12Bg)**

Yield 1.098 g (86%), off-white crystalline solid, m.p. 87–88 °C.

^1^H NMR (400 MHz, [D_6_]DMSO): δ = ^1^H NMR (400 MHz, DMSO-*d*_6_) δ 8.68 (t, *J* = 6.0 Hz, 1H, NH), 7.61–7.49 (m, 3H, Ar), 7.34–7.13 (m, 6H, Ar), 4.29 (d, *J* = 5.9 Hz, 2H, CH_2_), 4.16 (s, 2H, CH_2_), 3.98 (s, 2H, CH_2_).

^13^C NMR (101 MHz, [D_6_]DMSO): δ = 166.6, 162.5, 140.9, 140.5, 138.9, 137.8, 131.1, 130.8, 130.4, 129.3, 128.8, 128.2, 127.1, 126.8, 42.5, 37.0, 30.9.

MS (ESI): m/z = 423.1 [M+H]^+^.

IR (ATR) ν_max_ = 3330 (NH), 1658 (C=O) cm^−1^.

Anal. Calcd. for C_19_H_16_Cl_2_N_2_OS_2_: C, 53.90; H, 3.81; N, 6.62. Found: C, 53.80; H, 3.71; N, 5.53.

## Figures and Tables

**Table 1:** Reaction conditions for the generation of the title compound **5a**.

Entry	Conditions	Yield of 5a (%) ^a^	Yield of 6 (%) ^a^	Yield of 7 (%) ^a^	Reaction Time, h
1	Ethanol, reflux	0	40	-	10
2	Ethanol/HCl(conc.), reflux	78	-	-	6
3	Methanol/HCl(conc.), reflux	76	-	-	10
4	Acetic acid, reflux	24	-	-	10
5	Acetic acid/HCl(conc.), reflux	60	-	-	6
6	Dioxane/HCl(conc.), reflux	65	-	-	6
7	H_2_S/Ethanol, 70 °C	-	-	-	24
8	H_2_S/Ethanol/HCl(conc.), 70 °C	-	-	74	24

^a^ Isolated yields.

**Table 2:** Thiol/thione form ratios for **5A** and **5B** under NMR spectra measurement conditions.

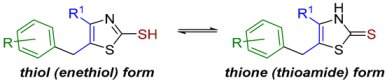			
	R =	Thiol/Thione Form Ratio (%) ^a^	
		5A	5B
**a**	H	0:100	0:100
**b**	2-CH_3_	7:93	0:100
**c**	4-CH_3_	7:93	0:100
**d**	4-F	0:100	10:90
**e**	3-CF_3_	0:100	100:0
**f**	2,5-Cl_2_	0:100	6:94
**g**	3,4-Cl_2_	0:100	4:96

^a^ Based on NMR data.

**Table 3:** Reactions conditions for the generation of the title compound **11Aa**.

Entry	Conditions	Yield of 11Aa (%) ^a^	Reaction Time, h
1	KOH/EtOH, reflux	35	2
2	EtONa/EtOH, reflux	60	2
3	Et_3_N/EtOH, reflux	87	2
4	Et_3_N/MeOH, reflux	86	5
5	Et_3_N/Dioxane, reflux	75	2
6	Et_3_N/PrOH, reflux	74	2

^a^ Isolated yields.

## Data Availability

The original contributions presented in this study are included in the article and Supplementary Materials. Further inquiries can be directed to the corresponding authors.

## Supplementary Materials

The following supporting information can be downloaded at https://www.mdpi.com/article/10.3390/molecules31162908/s1; ^1^H-NMR and ^13^C-NMR spectrum of compounds **4Aa**–**12Bg**.
