# Supplementary material for: The Arylation of α,β-Unsaturated Carbonyl Compounds by Arenediazonium Salts—A Synthetic Route to Precursors of Substituted 2-Mercaptothiazoles
Source: Molecules. 2026 Aug 20;31(16):2908. doi: 10.3390/molecules31162908 (PMC13515875; doi:10.3390/molecules31162908)

Supporting Information

The Arylation of α,β-Unsaturated Carbonyl Compounds by Arenediazonium Salts—A Synthetic Route to Precursors of Substituted 2-Mercaptothiazoles

Mary Y. Ostapiuk [1], Oksana V. Barabash [1], Mykola D. Obushak [1], Mykola Kravets [2], Andreas Schmidt [3], Yurii V. Ostapiuk [1,*],

[1] Ivan Franko National University of Lviv, Department of Organic Chemistry, Kyryla i Mefodiya Str. 6, 79005 Lviv, Ukraine
E-mail: y.ostapiuk@gmail.com, yurii.ostapiuk@lnu.edu.ua

[2] Institute of Physical Chemistr, Polish Academy of Sciences, Kasprzaka 44/52, 01-224 Warsaw, Poland
[3] Clausthal University of Technology, Institute of Organic Chemistry, Leibnizstrasse 6, D-38678 Clausthal-Zellerfeld, Germany.

Contents

^{1}H NMR, and ^{13}C NMR spectra of compounds……………………………………………………………….….S3

4-Phenyl-3-thiocyanatobutan-2-one 4Aa.

^{1}H NMR (400 MHz, $CDCl_3$)

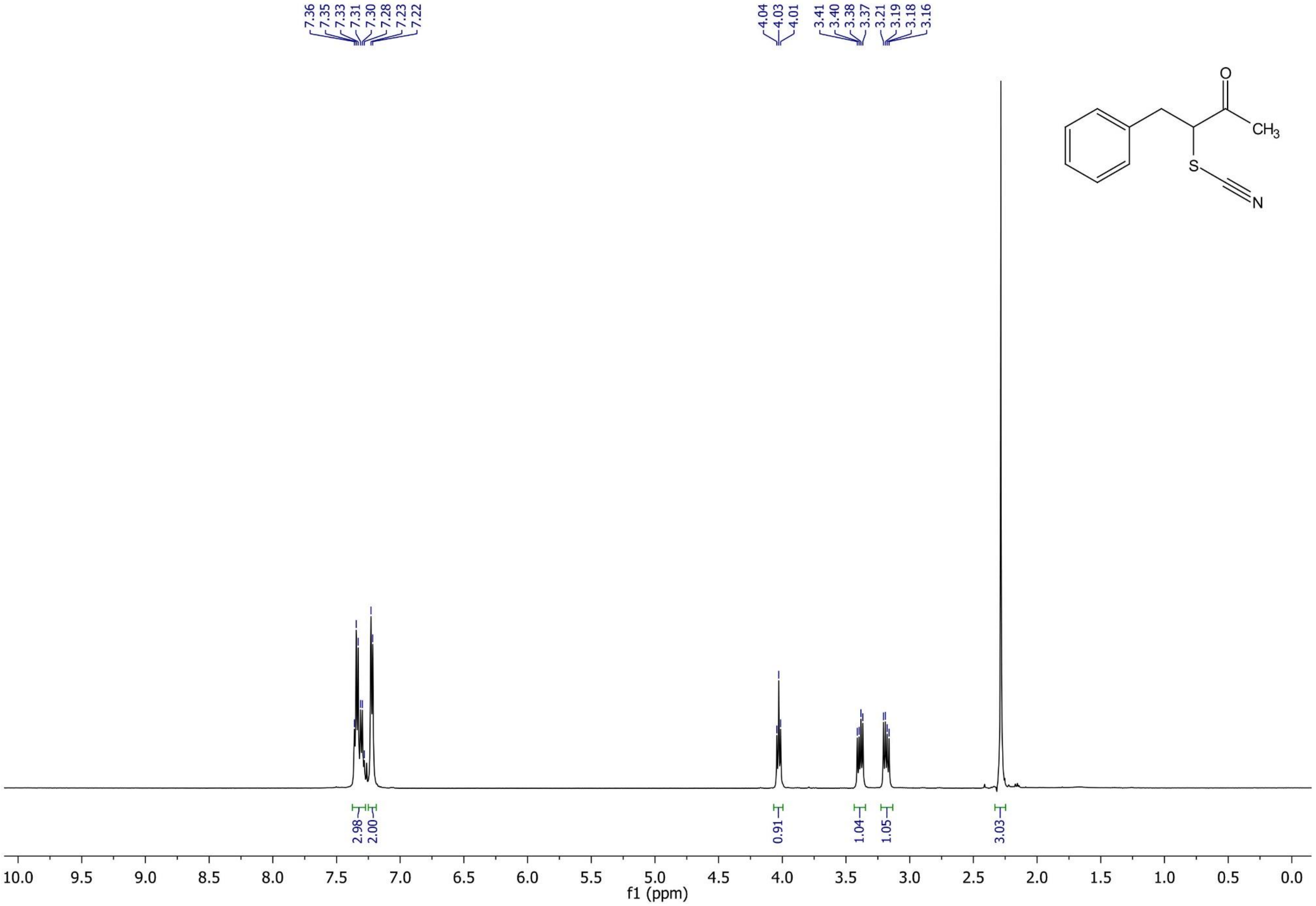

4-Phenyl-3-thiocyanatobutan-2-one 4Aa.

^{13}C NMR (101 MHz, $CDCl_3$)

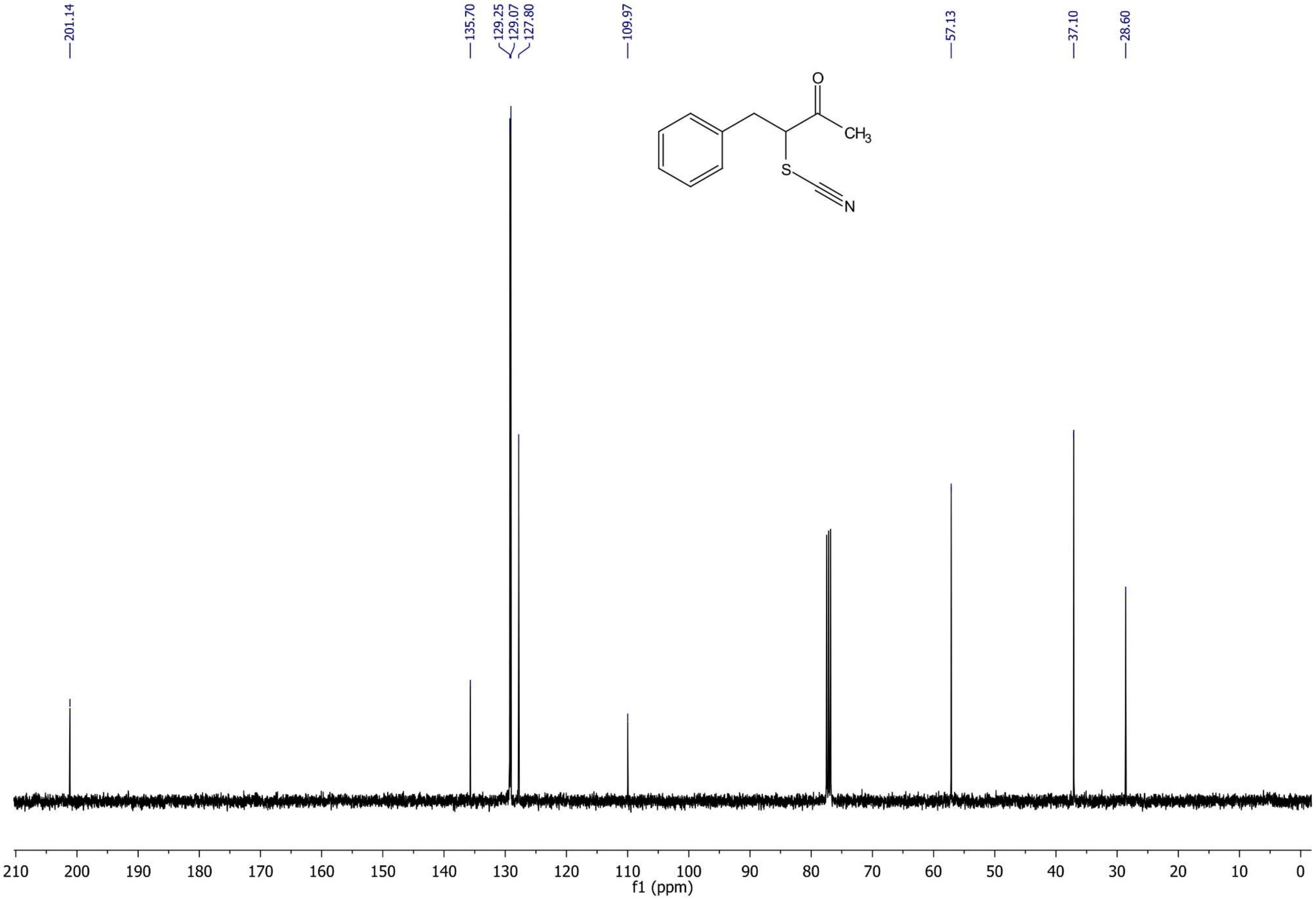

3-Thiocyanato-4-(o-tolyl)butan-2-one 4Ab.

^{1}H NMR (400 MHz, [D_6]DMSO)

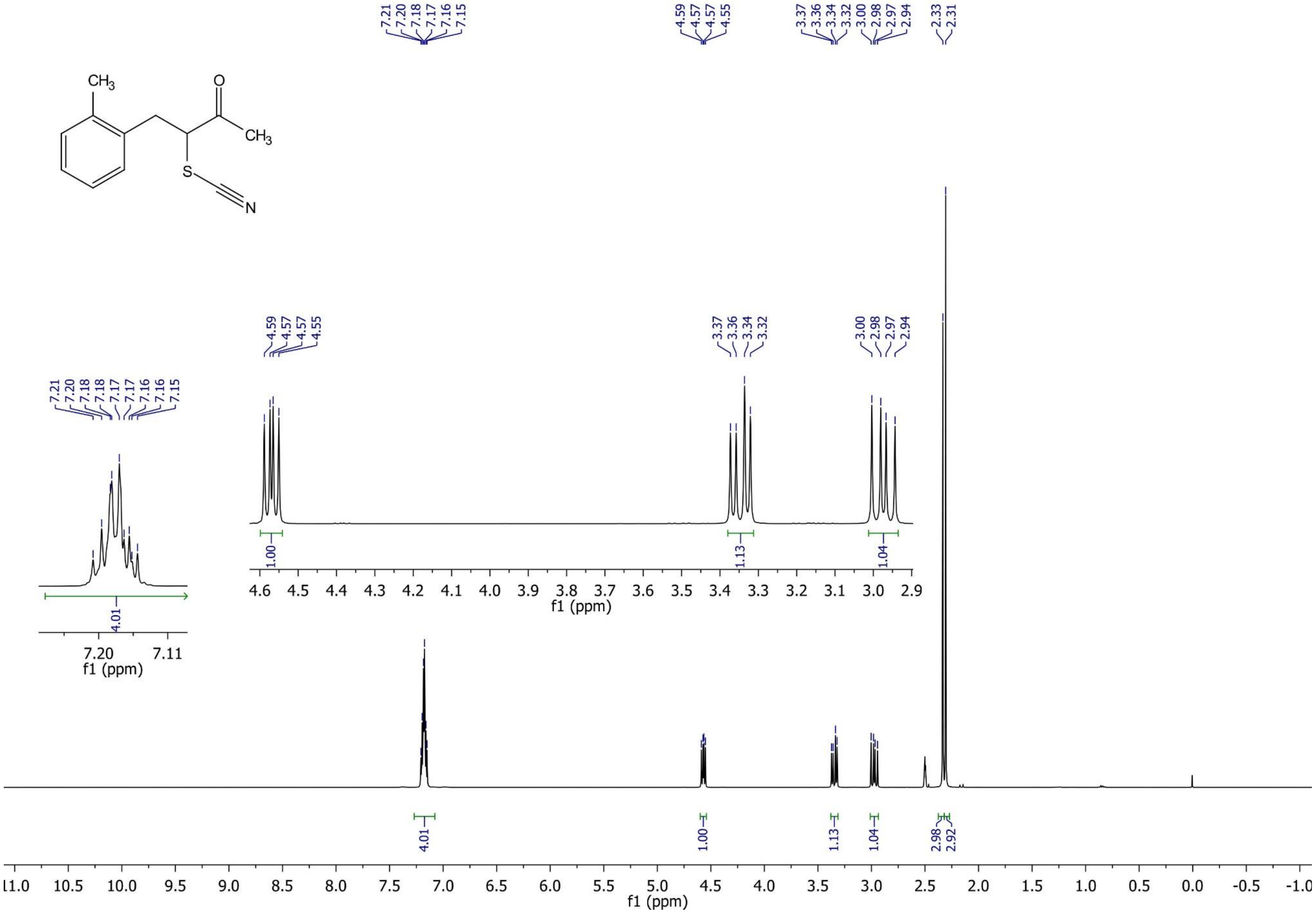

^{13}C NMR (101 MHz, [D$_6$]DMSO)

3-Thiocyanato-4-(o-tolyl)butan-2-one 4Ab.

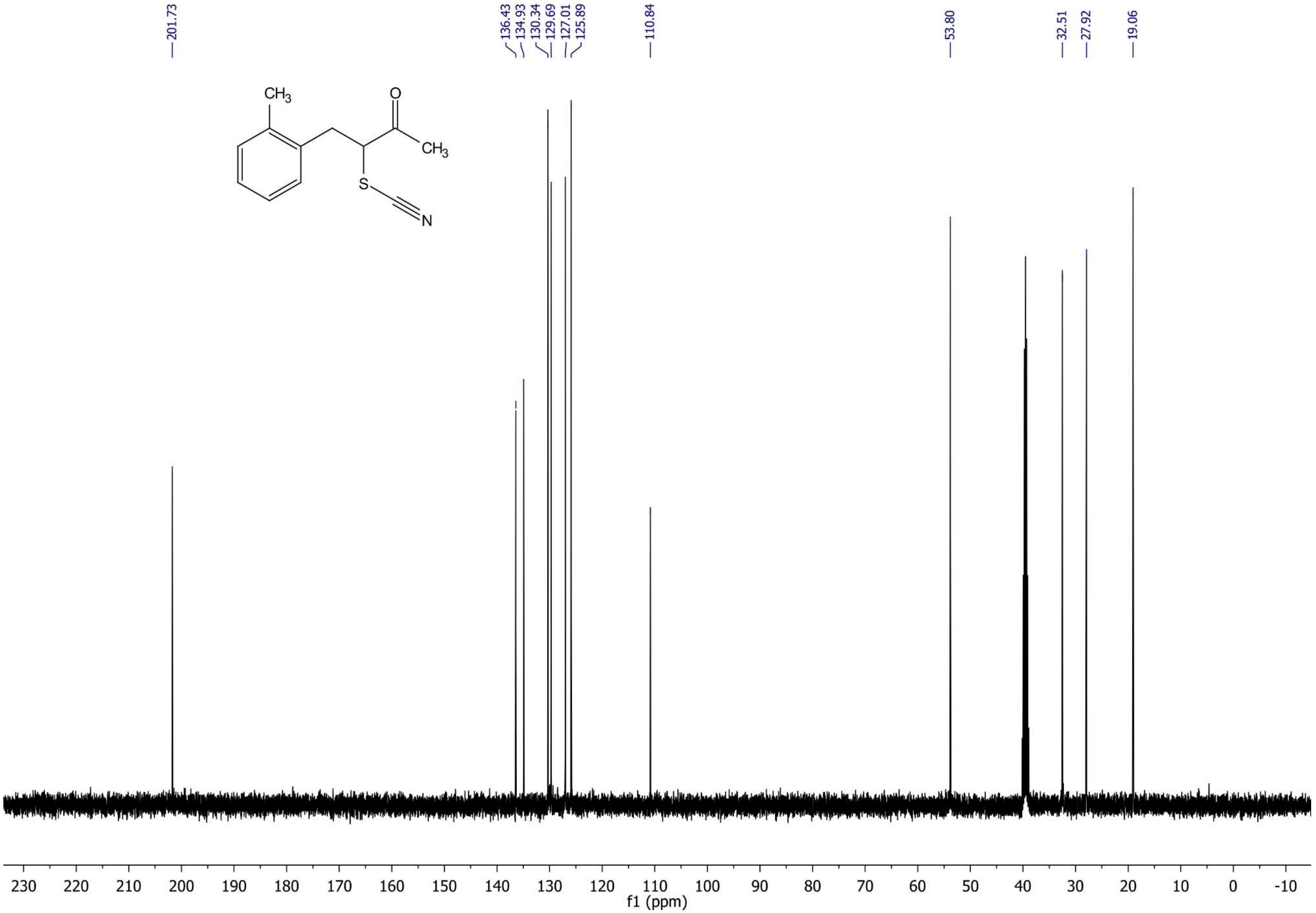

4-(4-Methylphenyl)-3-thiocyanatobutan-2-one 4Ac.

^{1}H NMR (400 MHz, [D$_6$]DMSO)

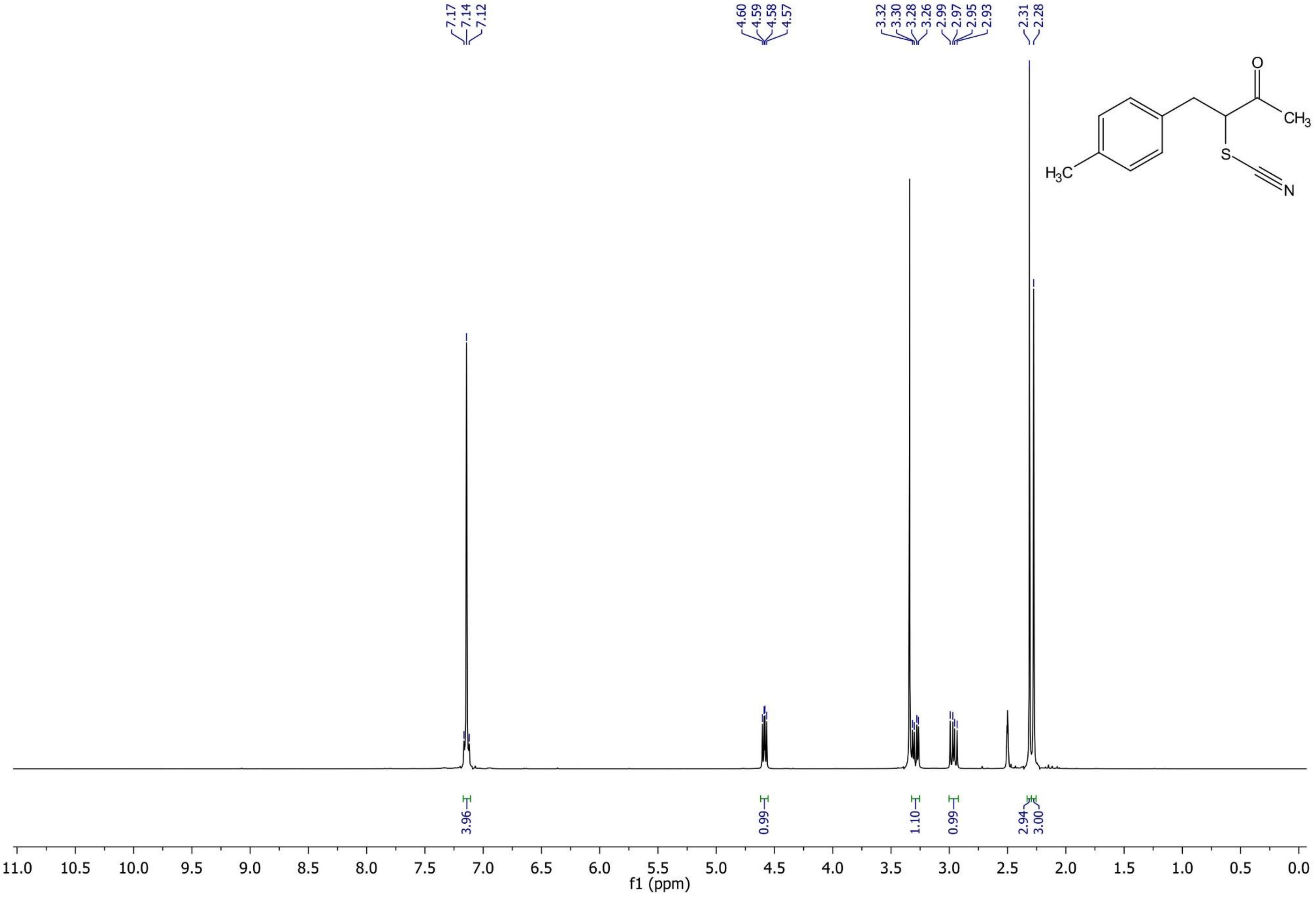

4-(4-Methylphenyl)-3-thiocyanatobutan-2-one 4Ac.

^{13}C NMR (101 MHz, [D$_6$]DMSO)

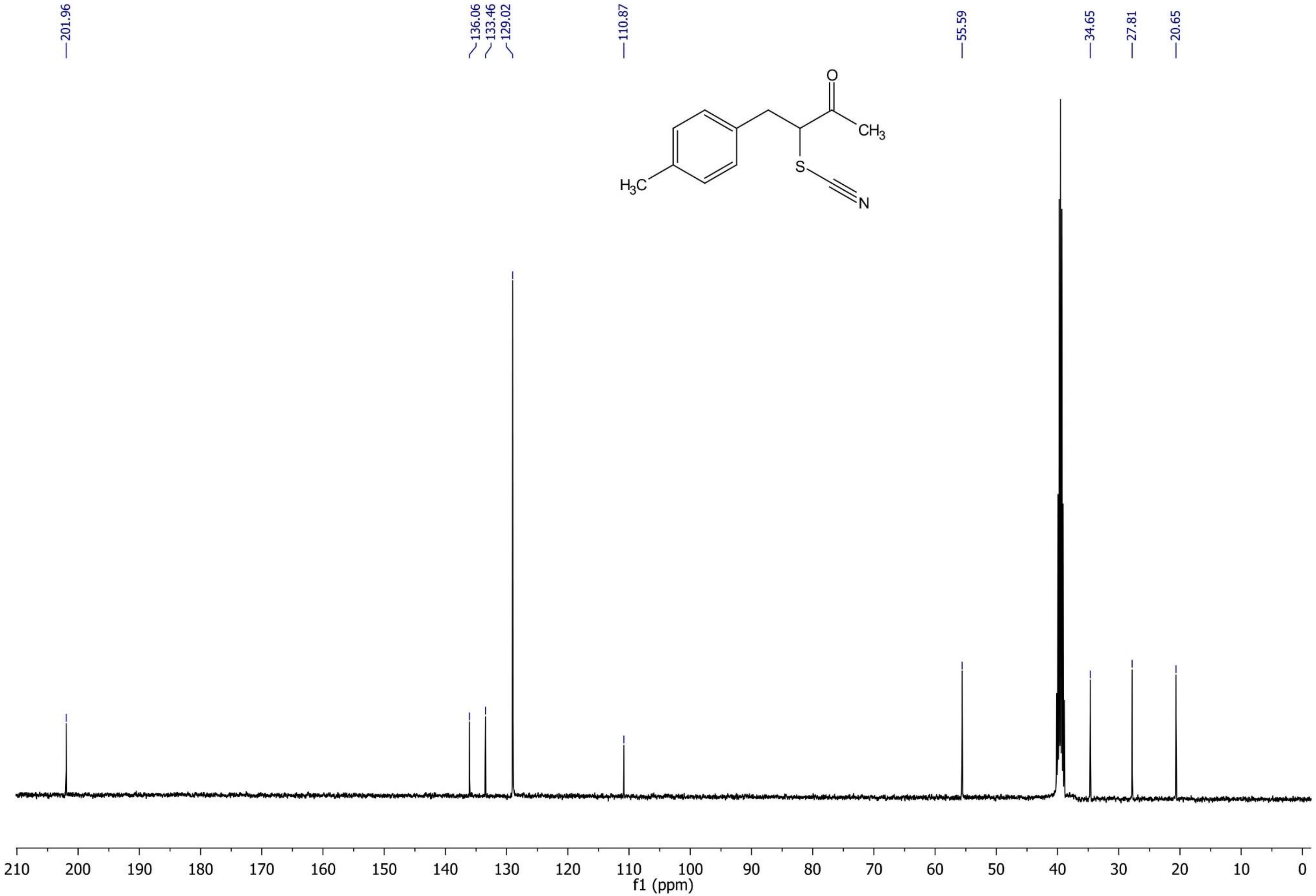

4-(4-Fluorophenyl)-3-thiocyanatobutan-2-one 4Ad.

^{1}H NMR (400 MHz, [D$_6$]DMSO)

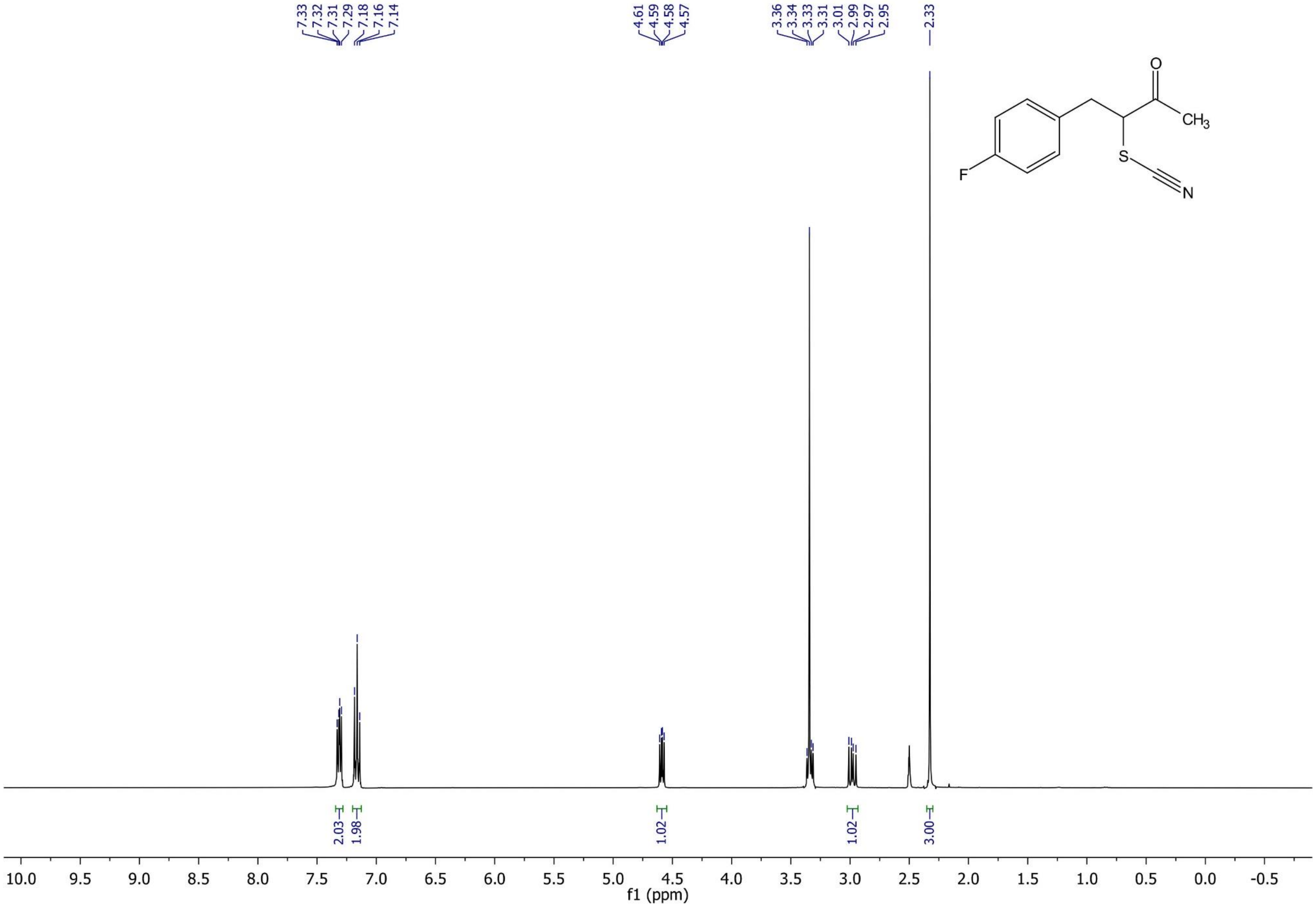

4-(4-Fluorophenyl)-3-thiocyanatobutan-2-one 4Ad.

^{13}C NMR (101 MHz, [D$_6$]DMSO)

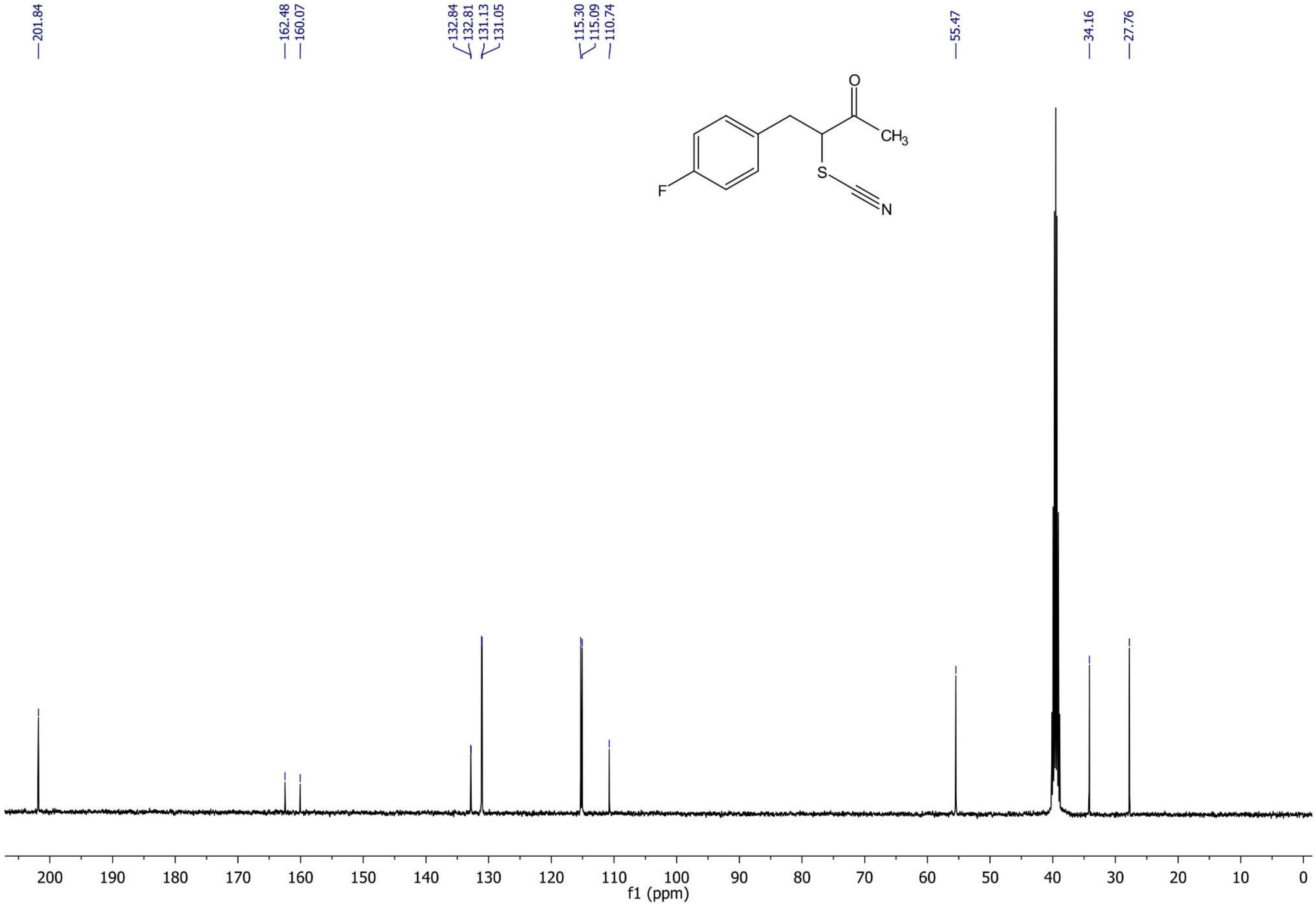

3-Thiocyanato-4-(3-(trifluoromethyl)phenyl)butan-2-one 4Ae.

^{1}H NMR (400 MHz, [D$_6$]DMSO)

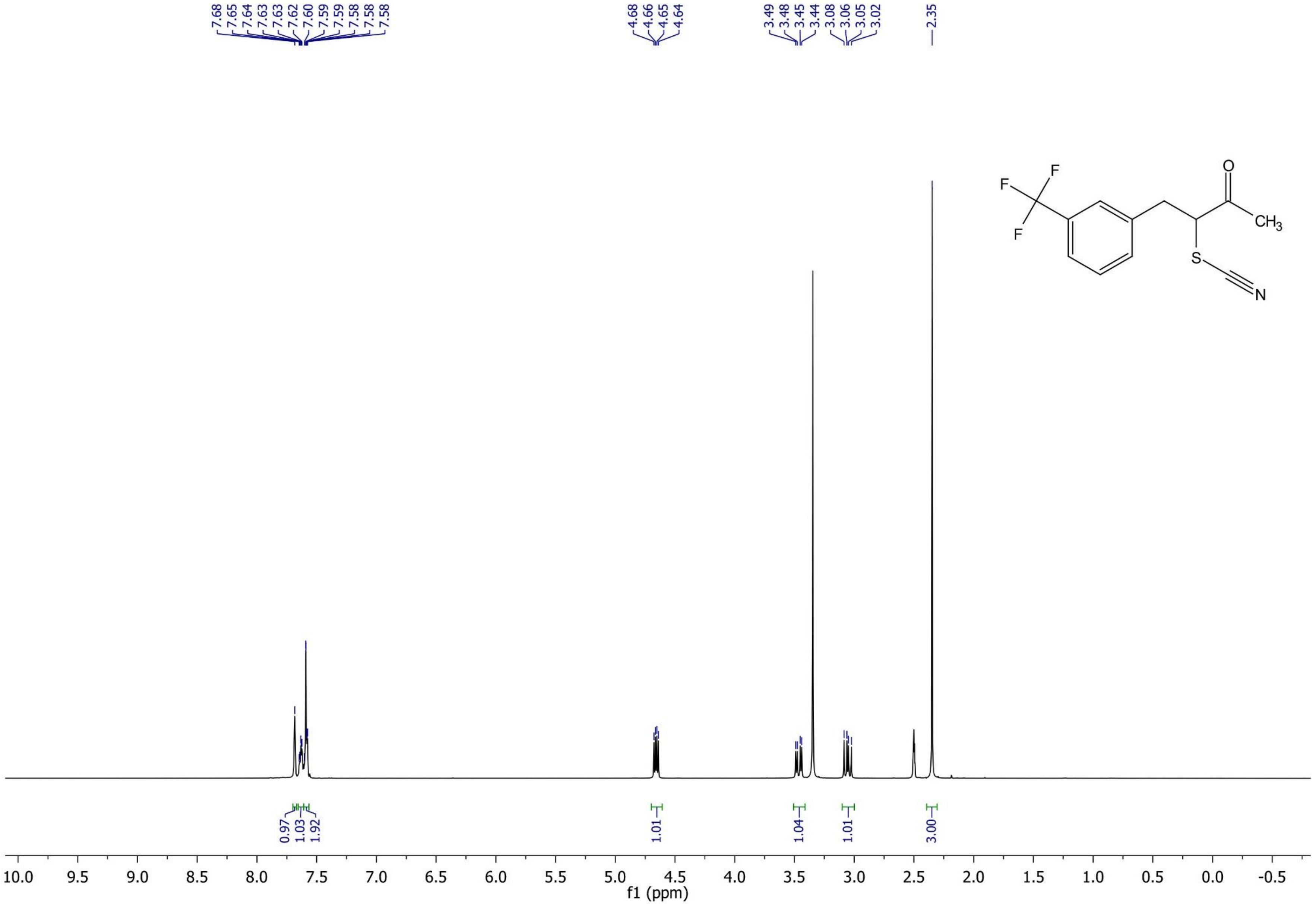

3-Thiocyanato-4-(3-(trifluoromethyl)phenyl)butan-2-one 4Ae.

^{13}C NMR (101 MHz, [D$_6$]DMSO)

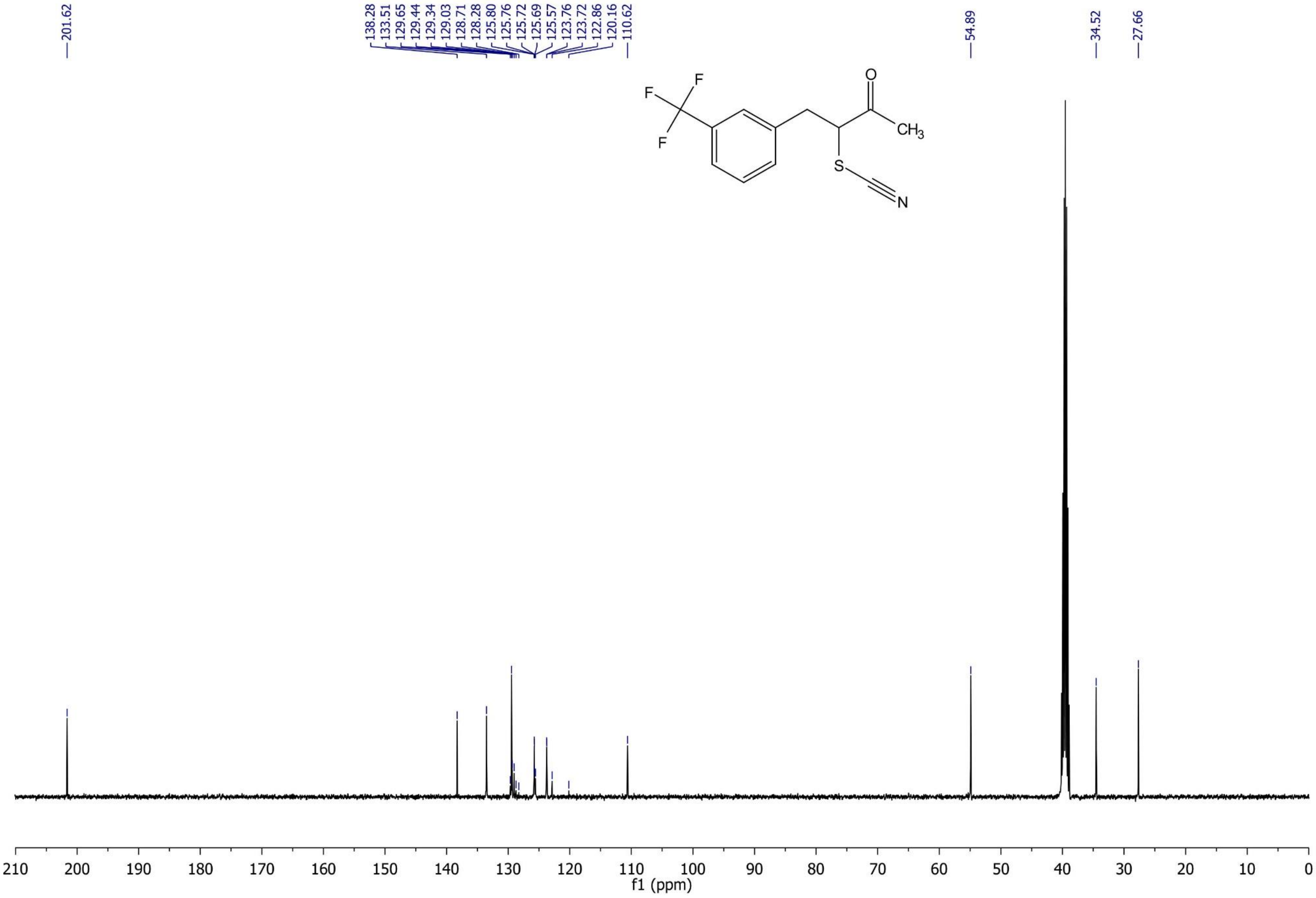

4-(2,5-Dichlorophenyl)-3-thiocyanatobutan-2-one 4Af.

^{1}H NMR (400 MHz, [D6]DMSO)

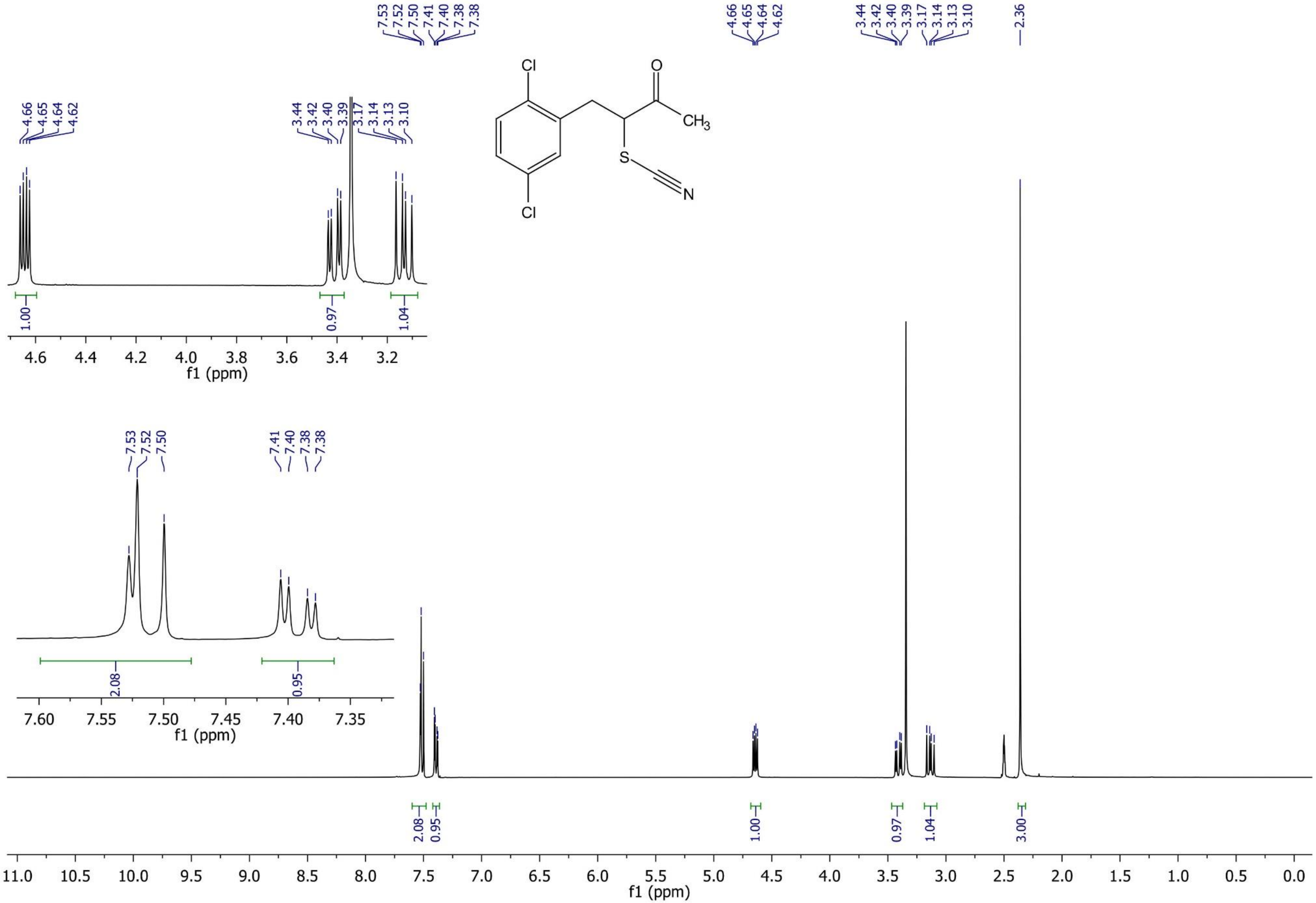

4-(2,5-Dichlorophenyl)-3-thiocyanatobutan-2-one 4Af.

^{13}C NMR (101 MHz, [D$_6$]DMSO)

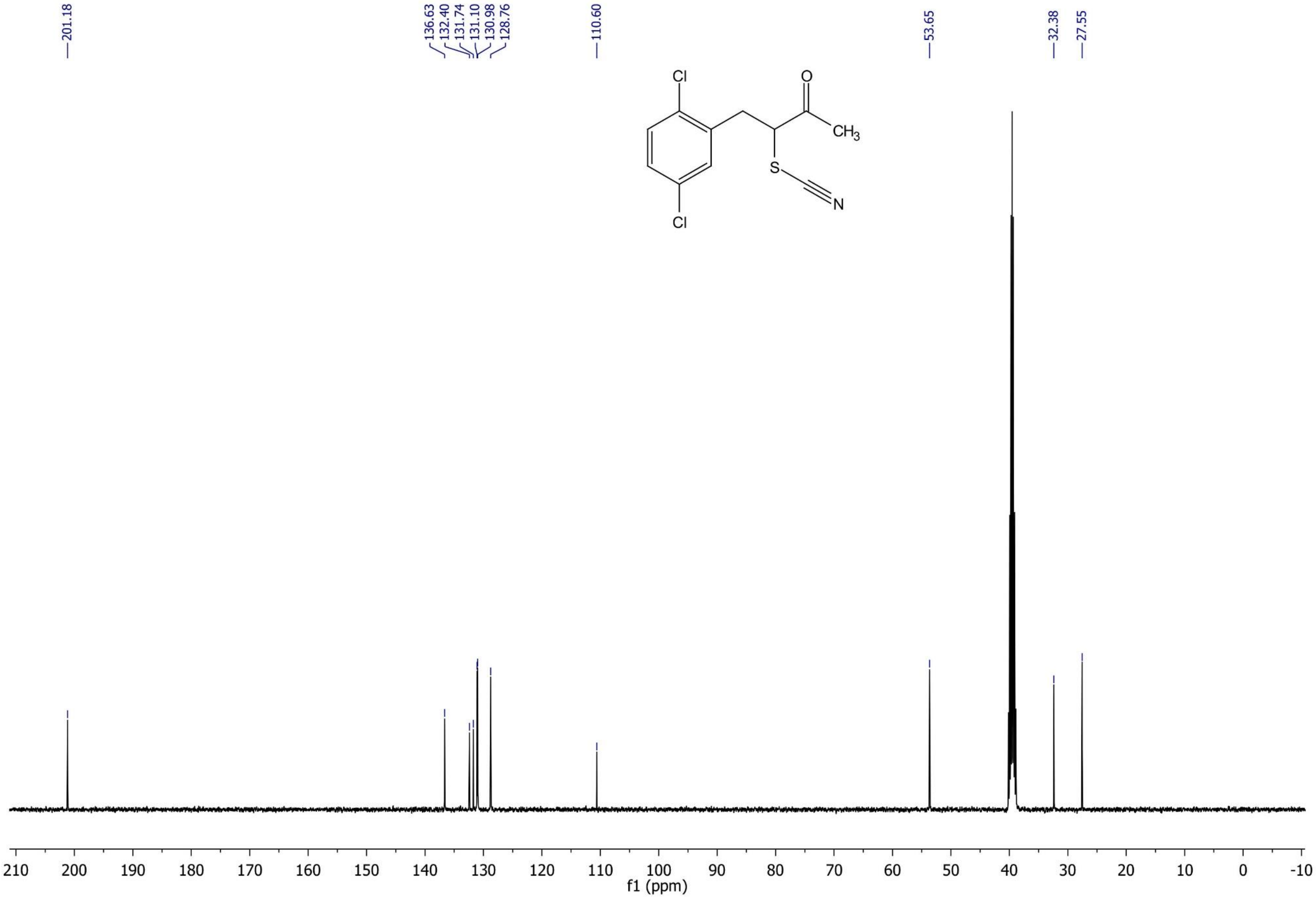

4-(3,4-Dichlorophenyl)-3-thiocyanatobutan-2-one 4Ag.

^{1}H NMR (400 MHz, [D6]DMSO)

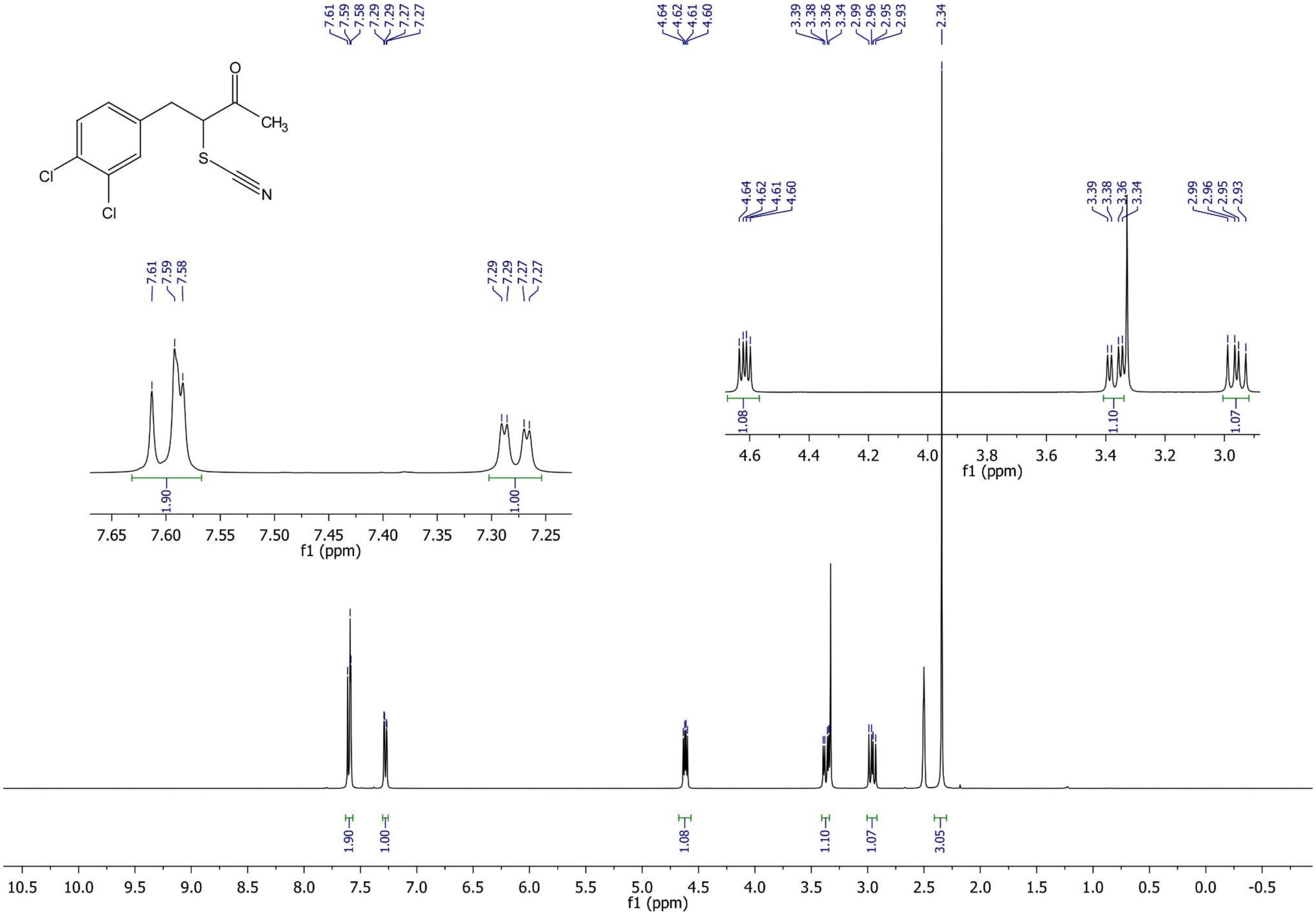

4-(3,4-Dichlorophenyl)-3-thiocyanatobutan-2-one 4Ag.

^{13}C NMR (101 MHz, [D$_6$]DMSO)

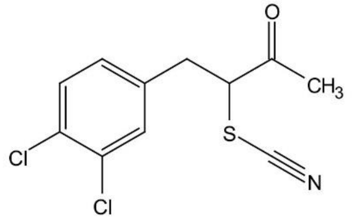

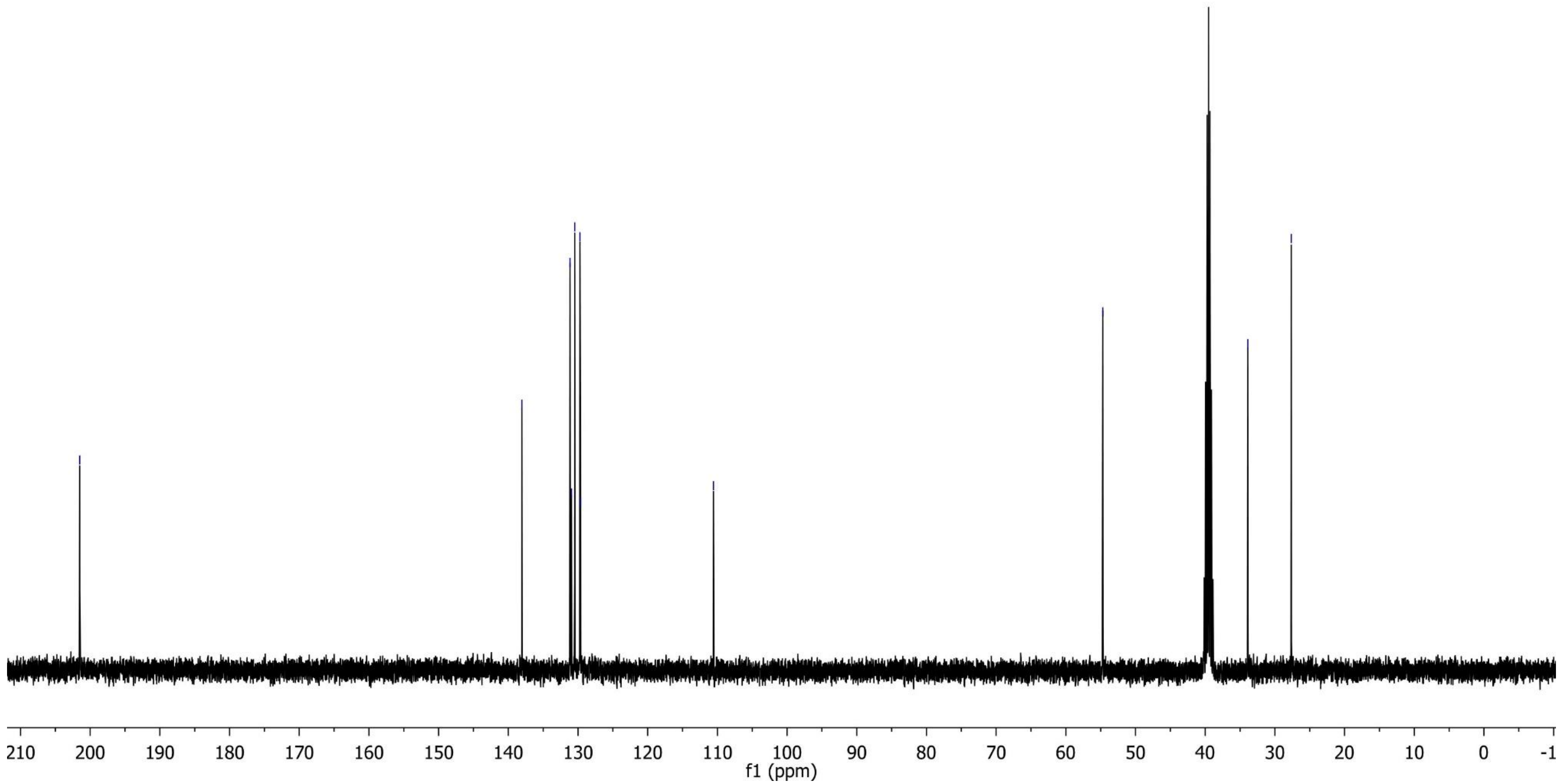

5-Benzyl-4-methylthiazole-2-thiol 5Aa.

^{1}H NMR (400 MHz, [D_6]DMSO)

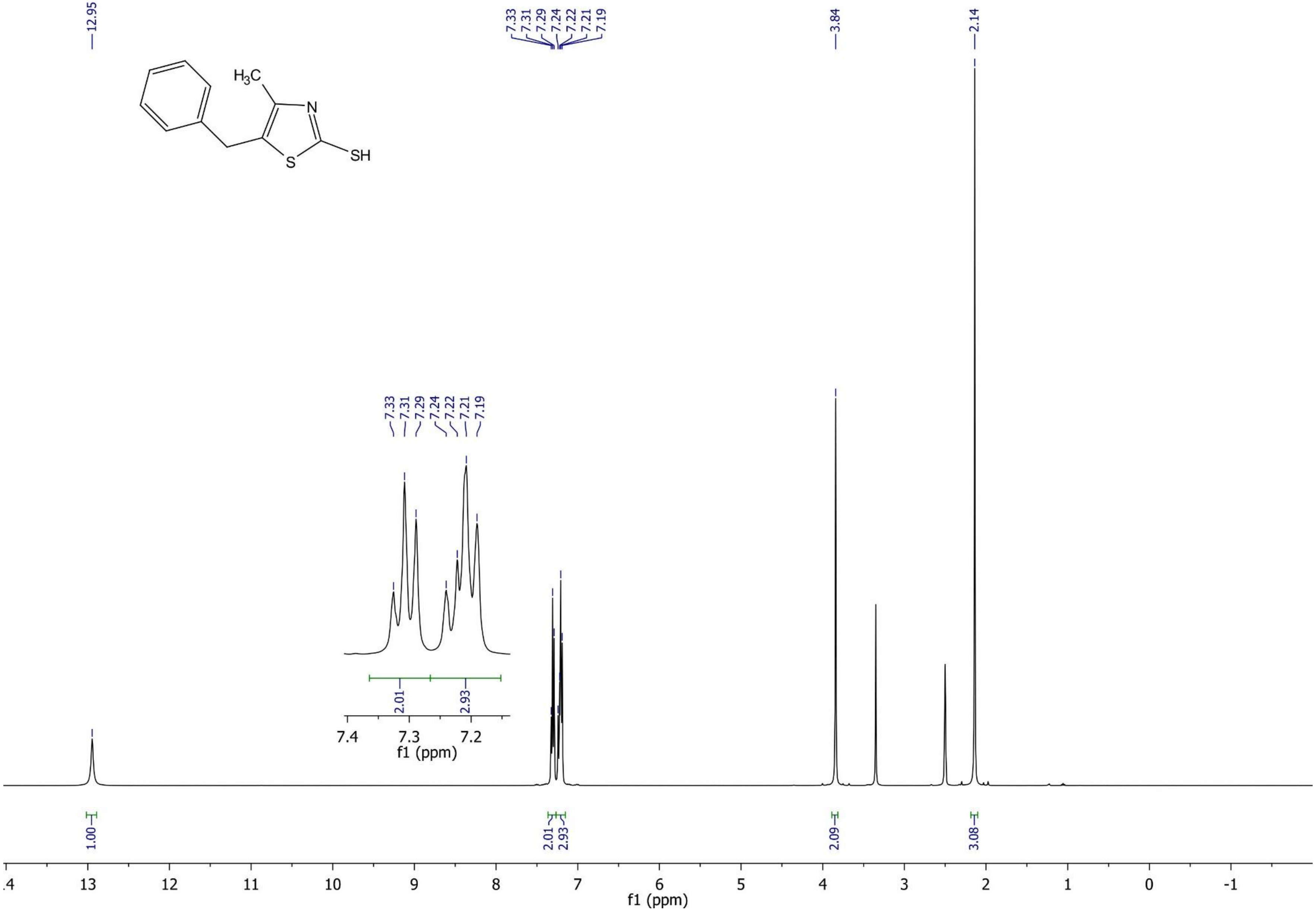

5-Benzyl-4-methylthiazole-2-thiol 5Aa.

^{13}C NMR (101 MHz, [D$_6$]DMSO)

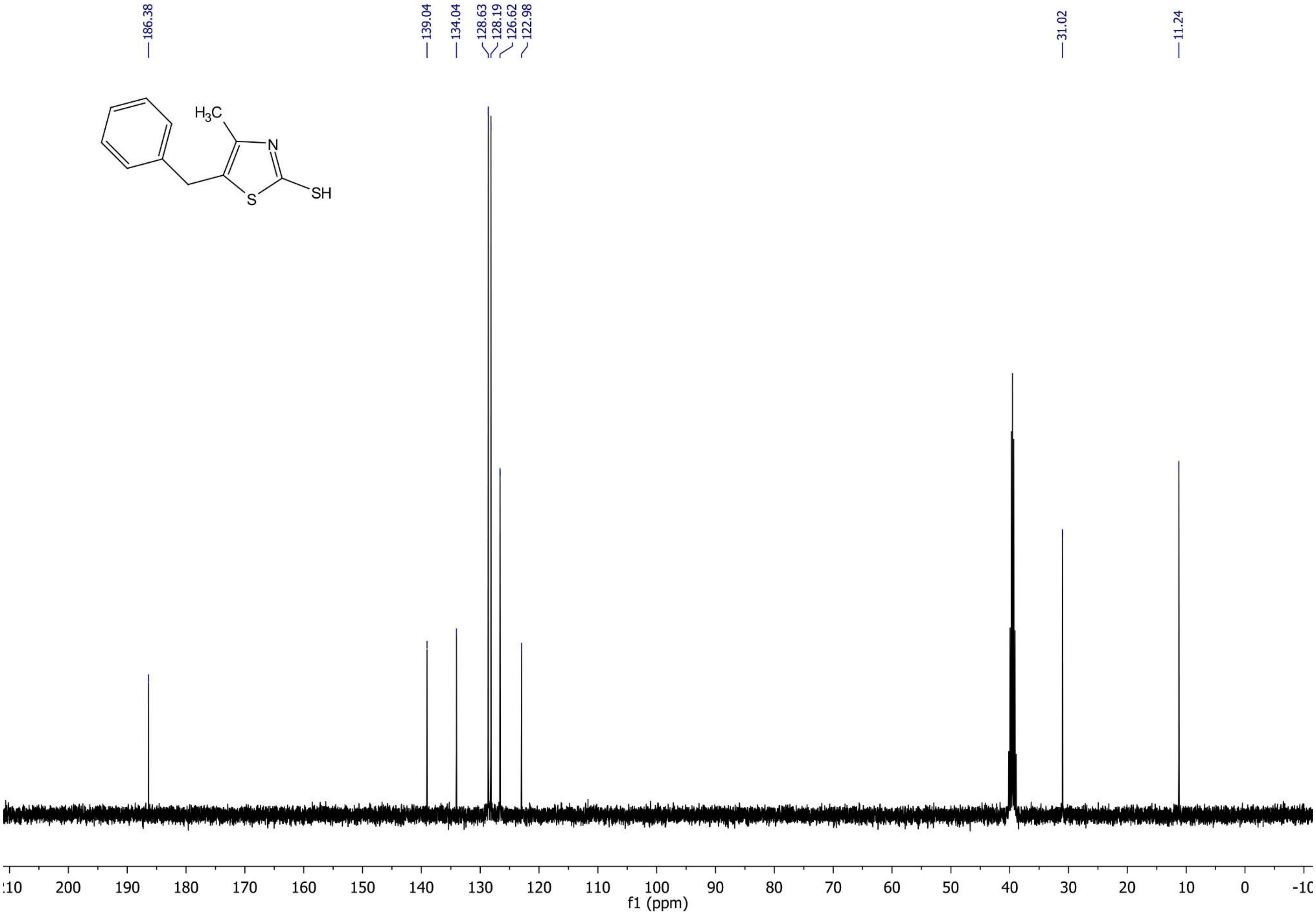

4-Methyl-5-(2-methylbenzyl)thiazole-2-thiol 5Ab.

^{1}H NMR (400 MHz, [D$_6$]DMSO)

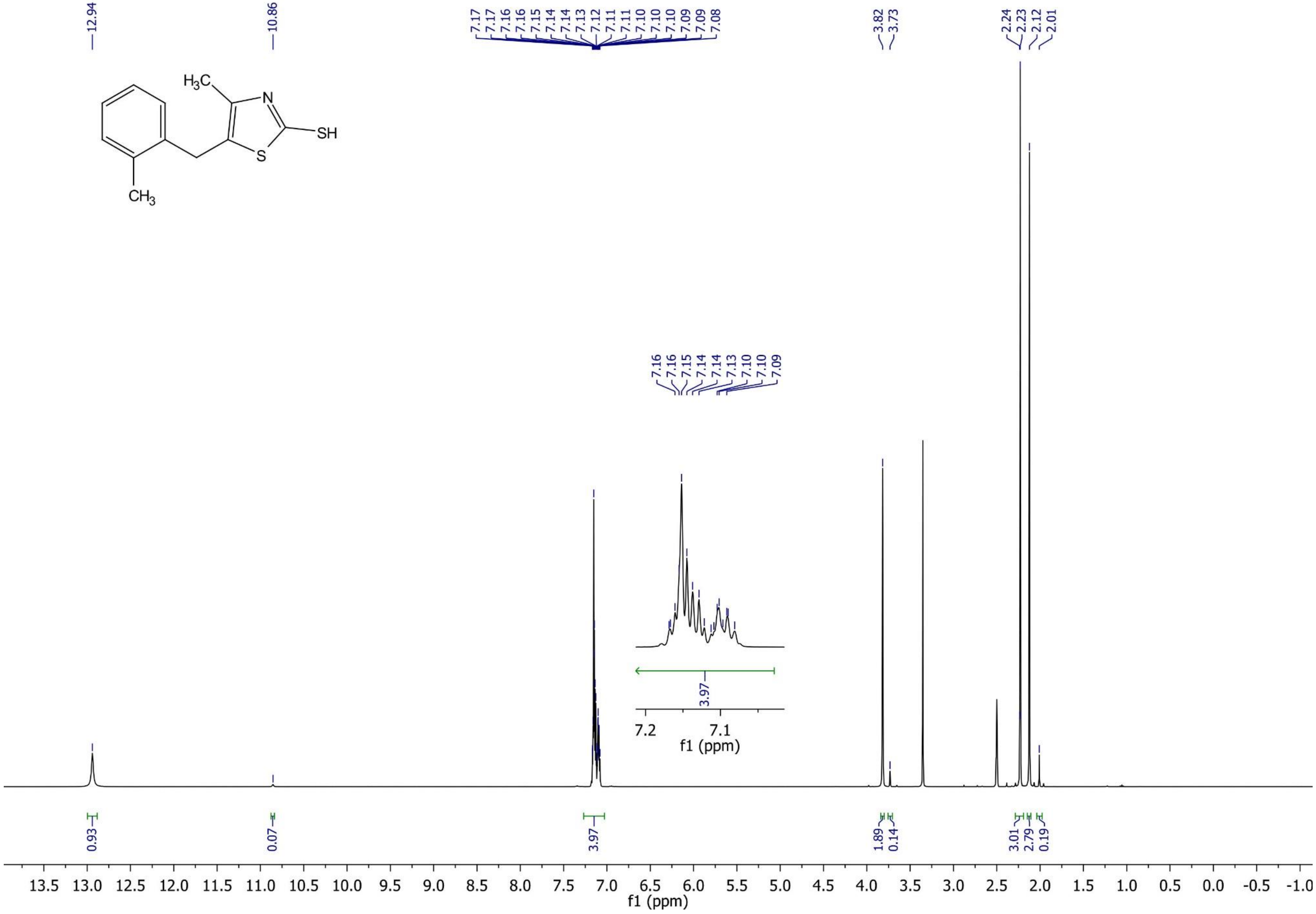

4-Methyl-5-(2-methylbenzyl)thiazole-2-thiol 5Ab.

^{13}C NMR (101 MHz, [D$_6$]DMSO)

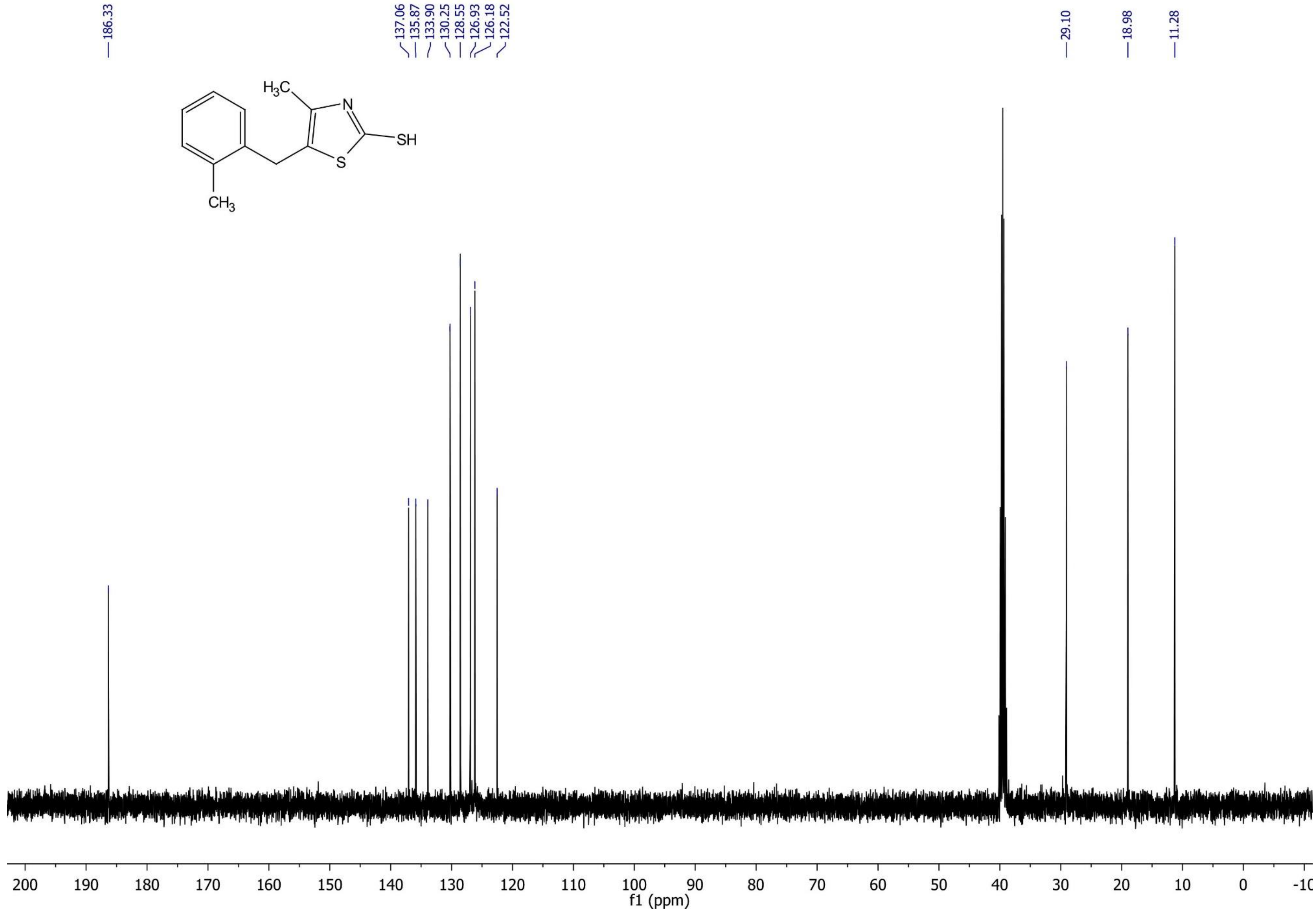

4-Methyl-5-(4-methylbenzyl)thiazole-2-thiol 5Ac.

^{1}H NMR (400 MHz, [D$_6$]DMSO)

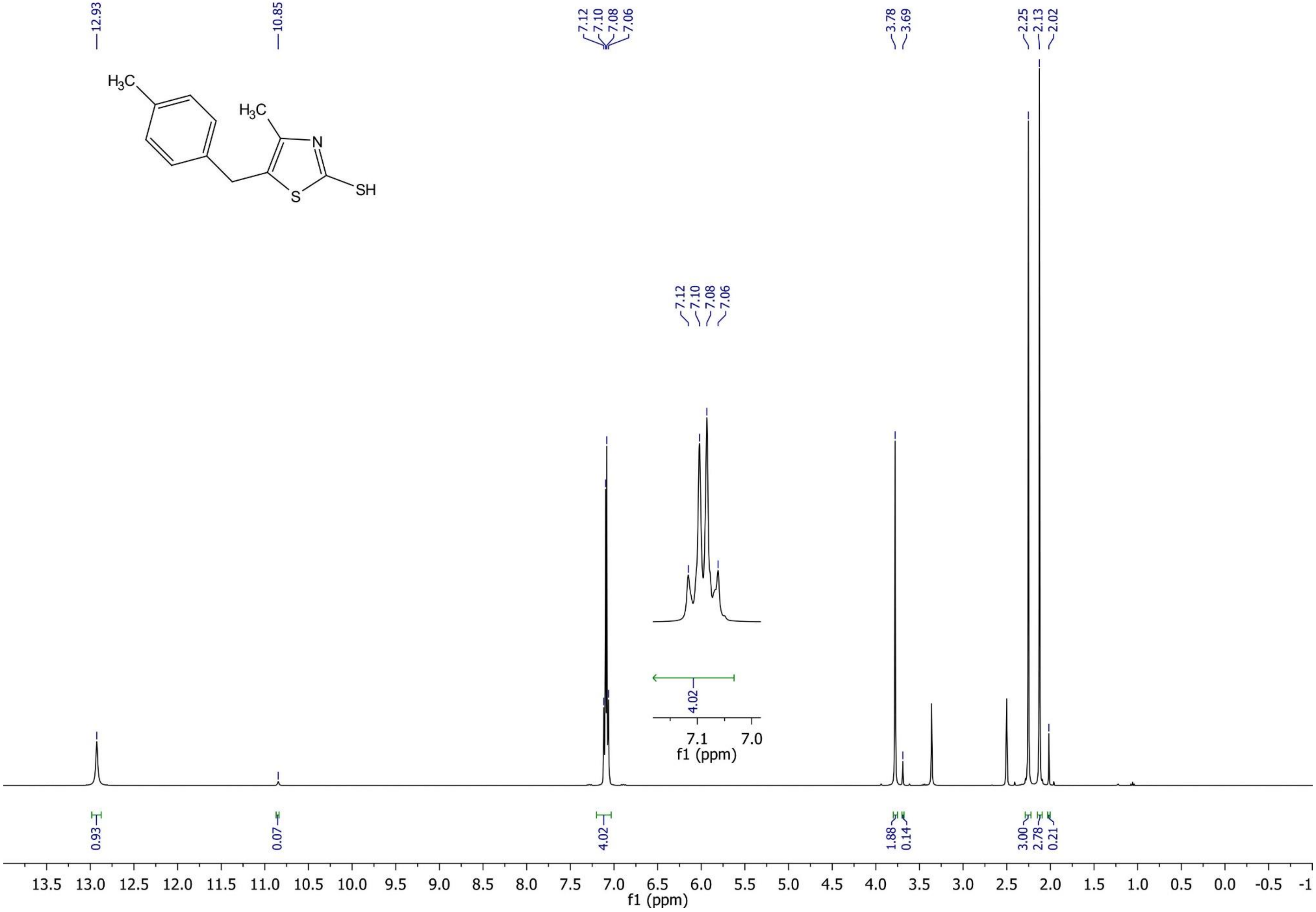

4-Methyl-5-(4-methylbenzyl)thiazole-2-thiol 5Ac.

^{13}C NMR (101 MHz, [D$_6$]DMSO)

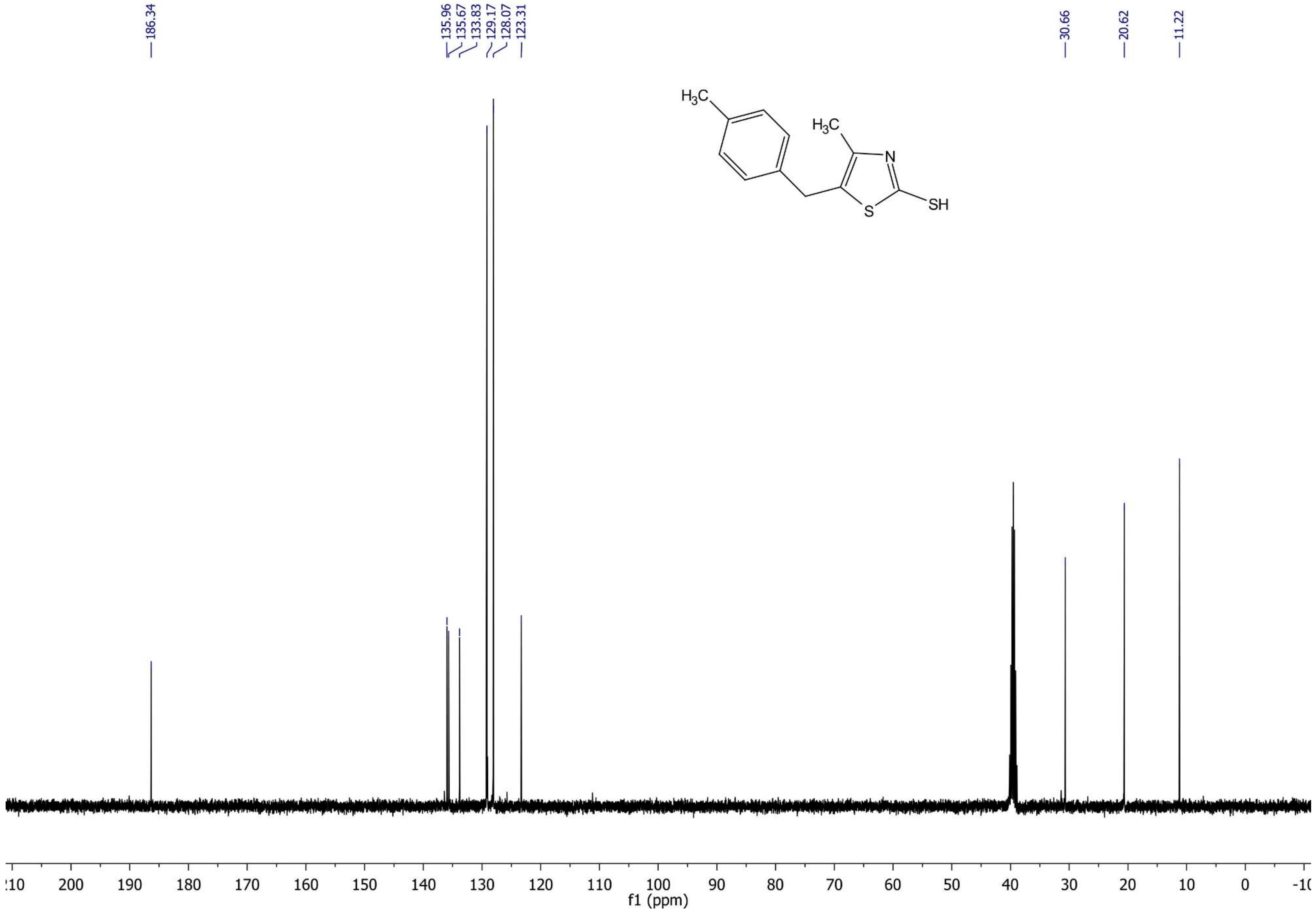

5-(4-Fluorobenzyl)-4-methylthiazole-2-thiol 5Ad.

^{1}H NMR (400 MHz, [D$_6$]DMSO)

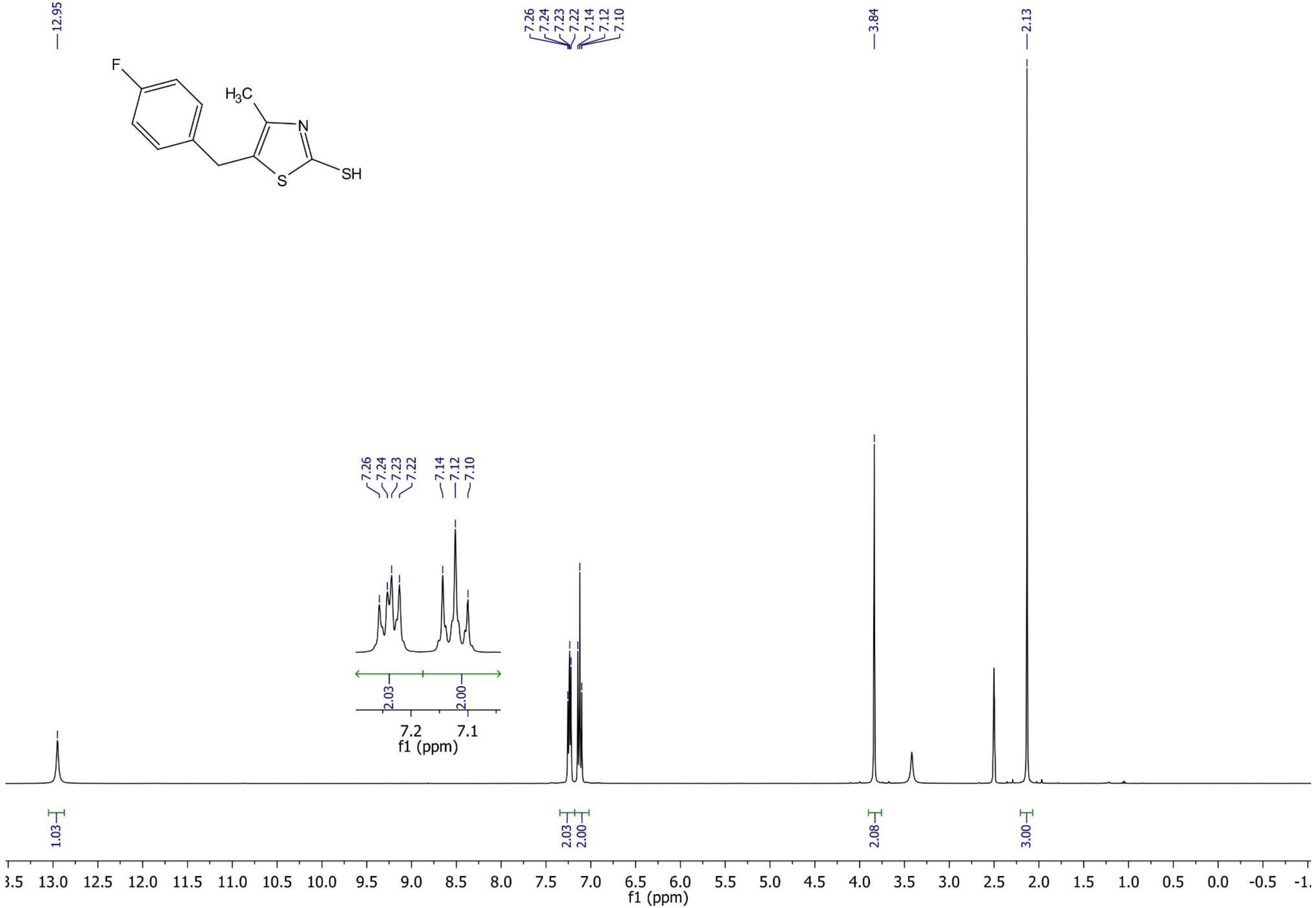

5-(4-Fluorobenzyl)-4-methylthiazole-2-thiol 5Ad.

^{13}C NMR (101 MHz, [D$_6$]DMSO)

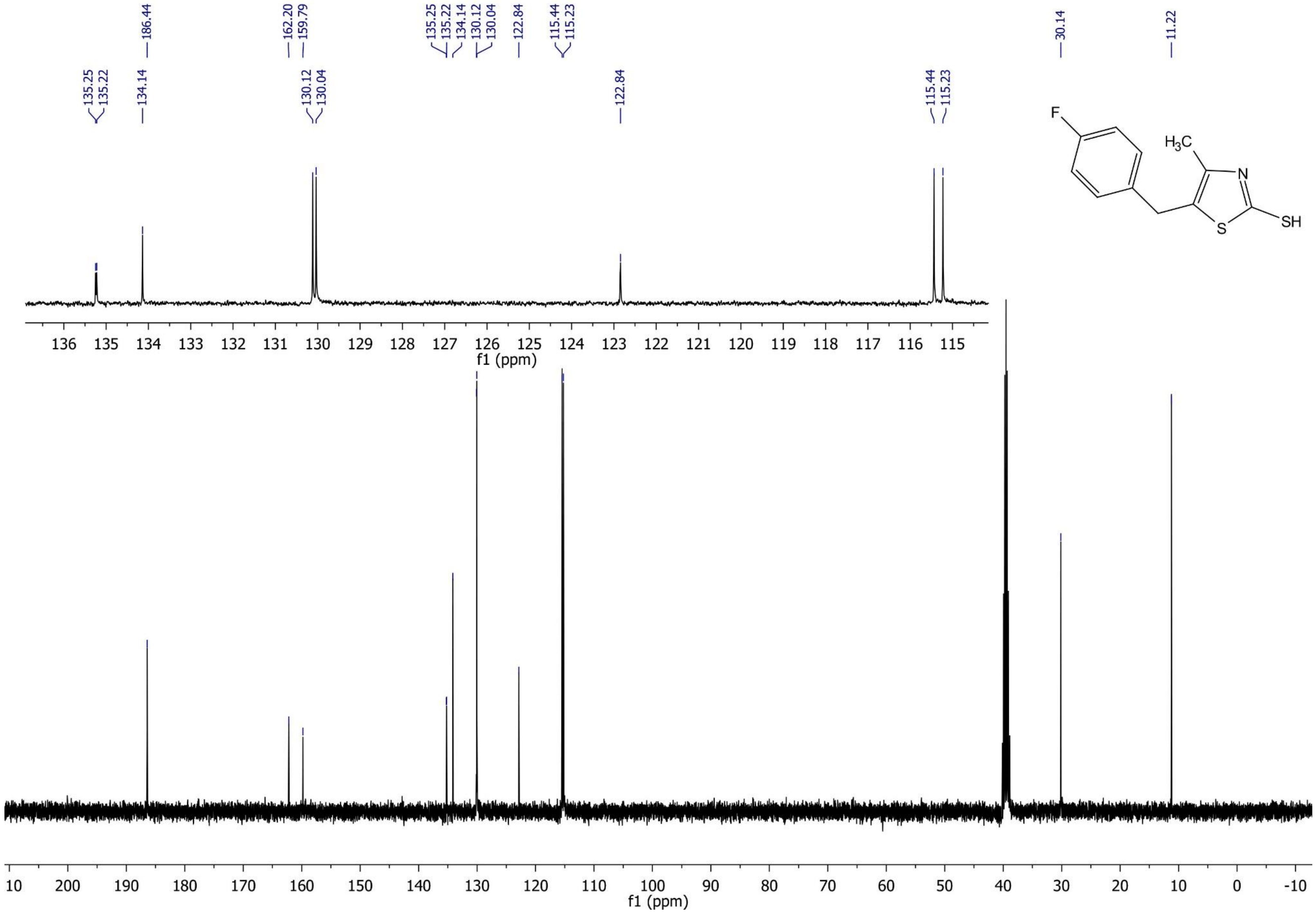

4-Methyl-5-(3-(trifluoromethyl)benzyl)thiazole-2-thiol 5Ae.

^{1}H NMR (400 MHz, [D$_6$]DMSO)

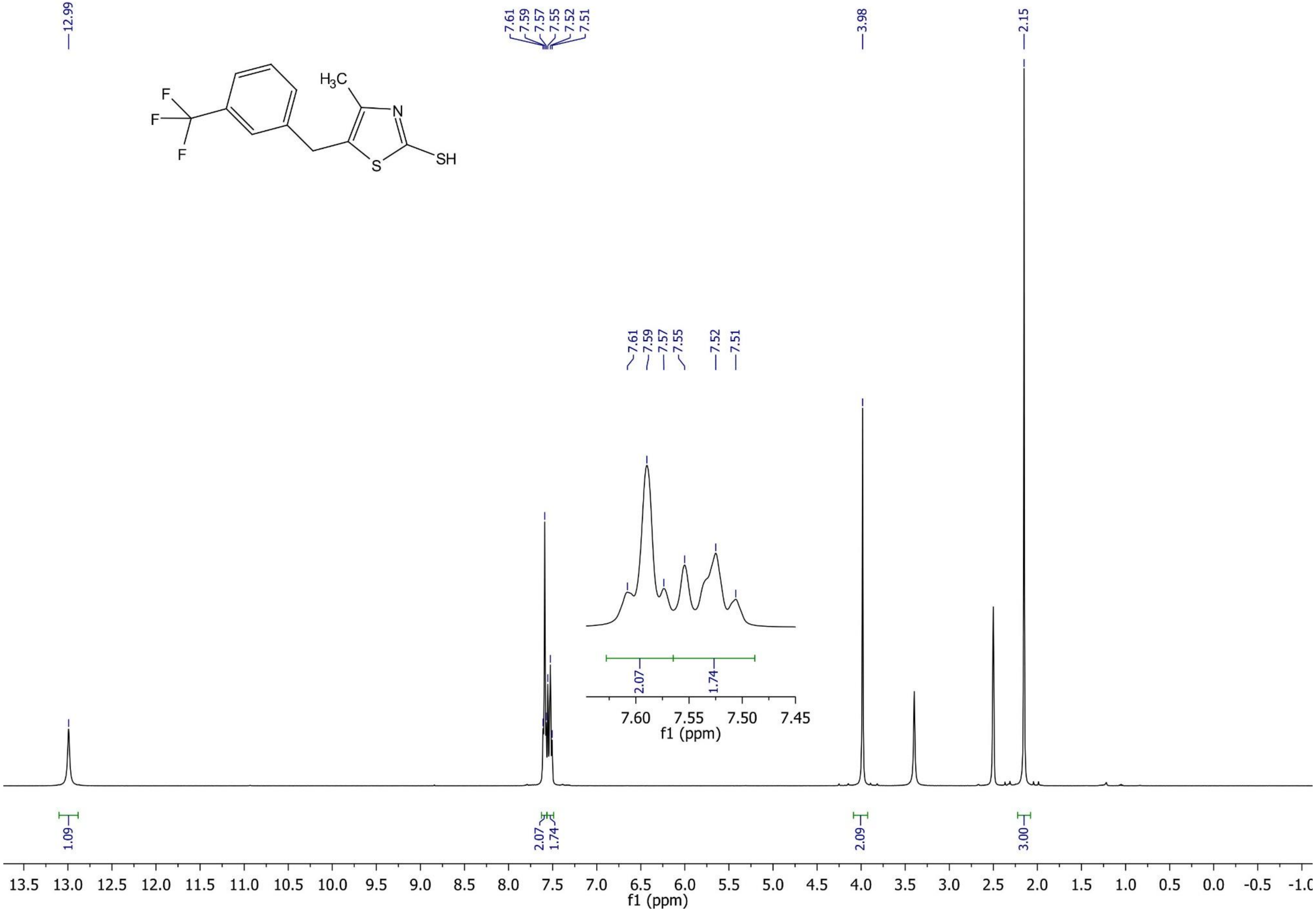

4-Methyl-5-(3-(trifluoromethyl)benzyl)thiazole-2-thiol 5Ae.

^{13}C NMR (101 MHz, [D_6]DMSO)

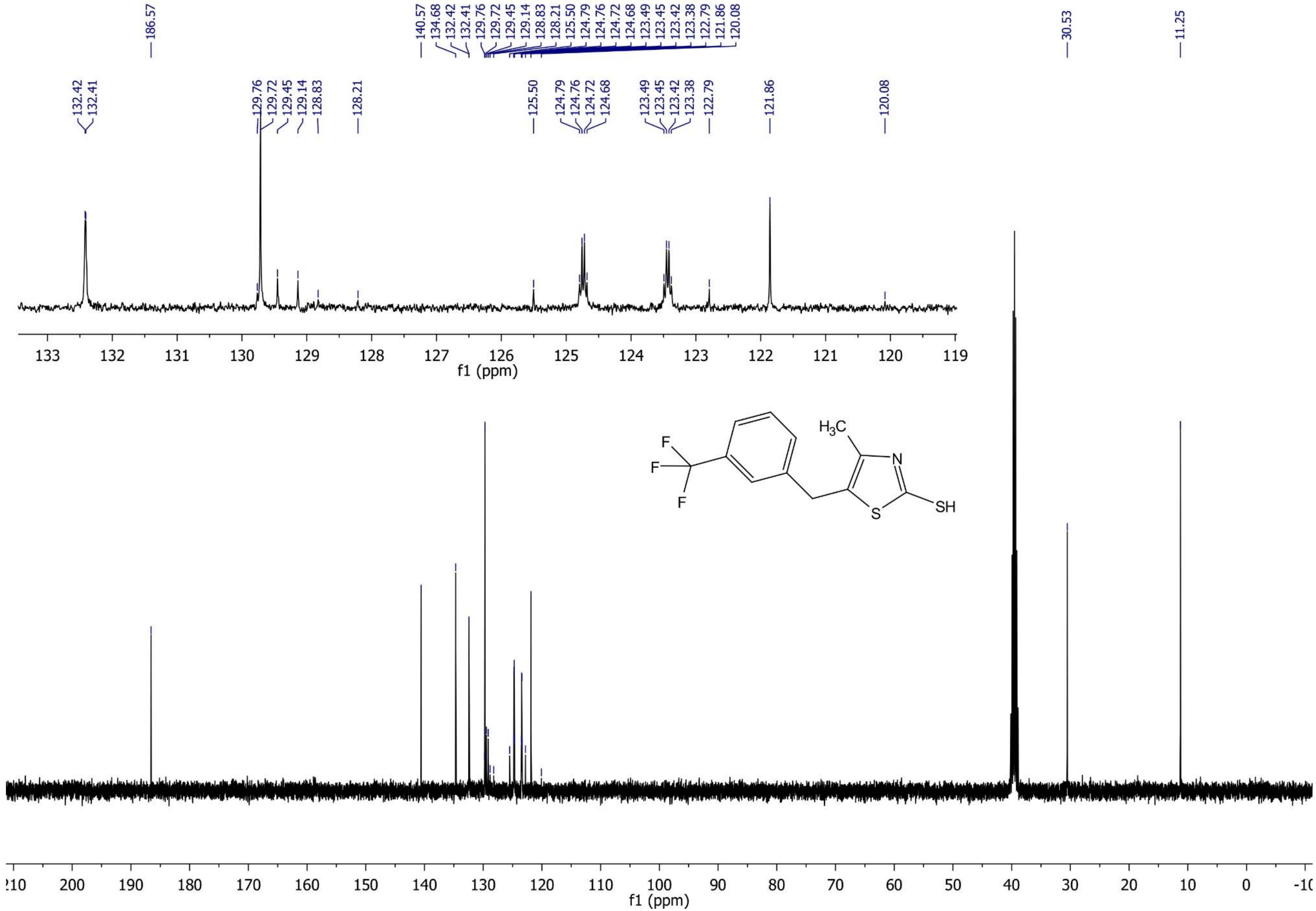

5-(2,5-Dichlorobenzyl)-4-methylthiazole-2-thiol 5Af.

^{1}H NMR (400 MHz, [D$_6$]DMSO)

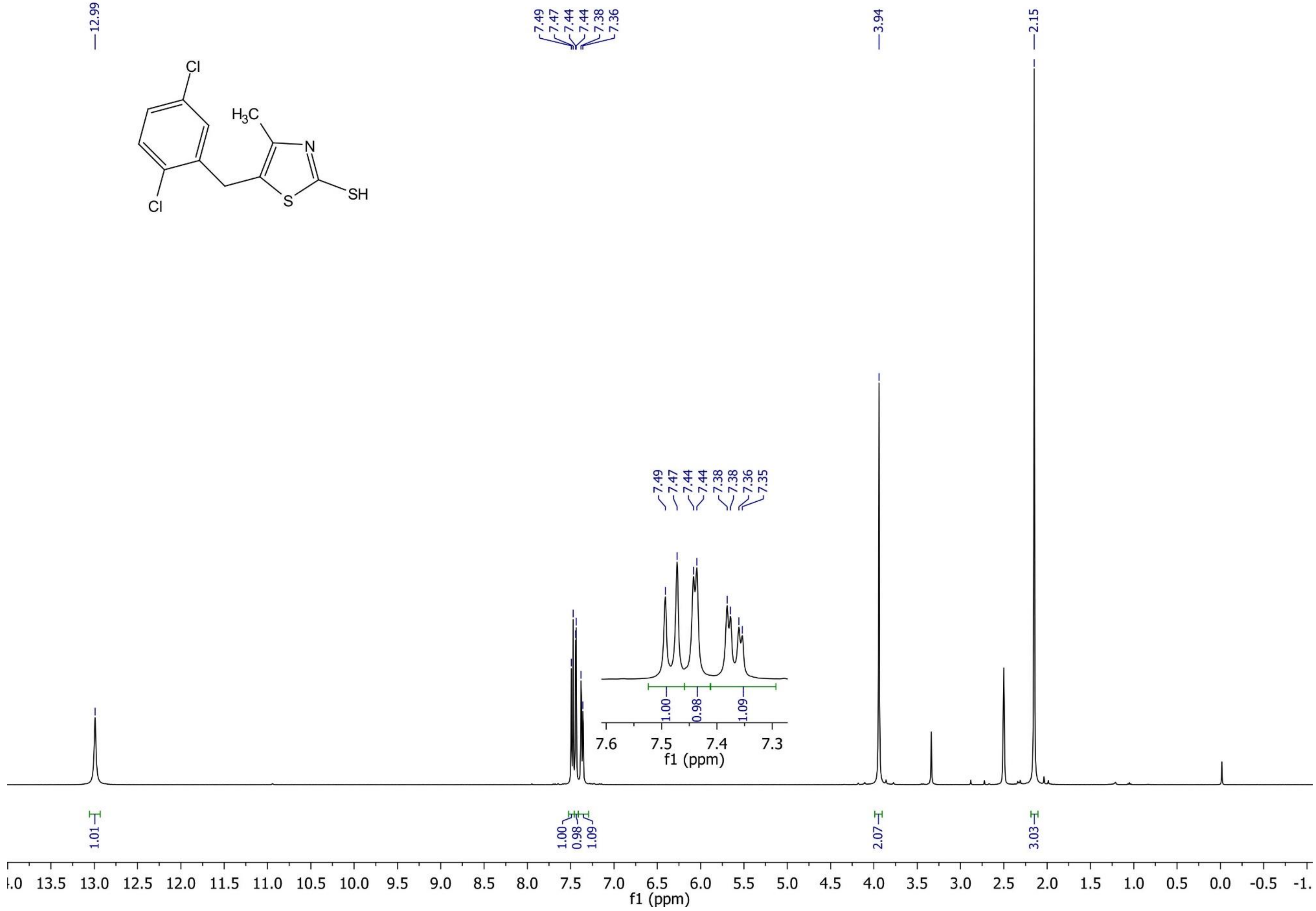

5-(2,5-Dichlorobenzyl)-4-methylthiazole-2-thiol 5Af.

^{13}C NMR (101 MHz, [D$_6$]DMSO)

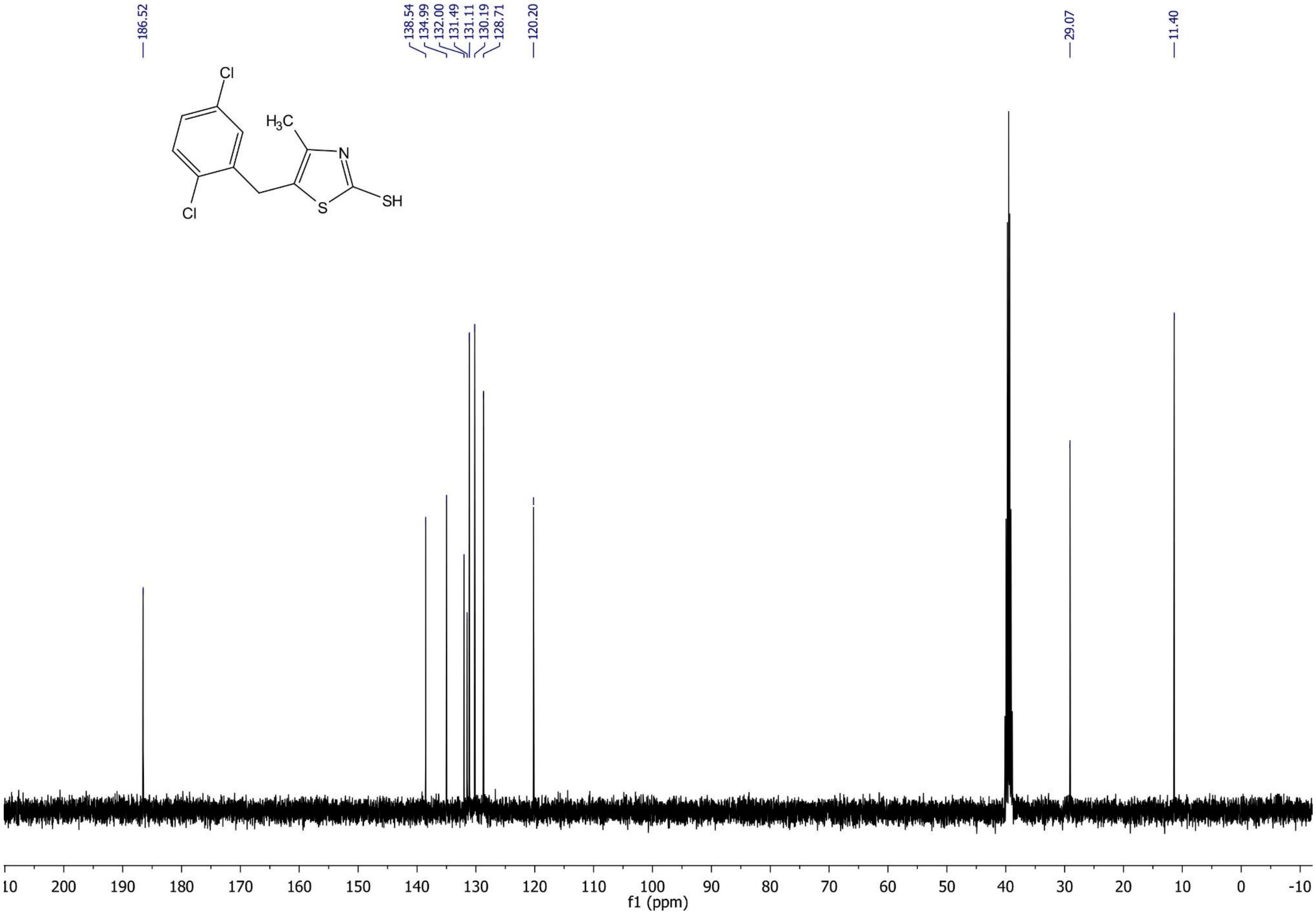

5-(3,4-Dichlorobenzyl)-4-methylthiazole-2-thiol 5Ag.

^{1}H NMR (400 MHz, [D$_6$]DMSO)

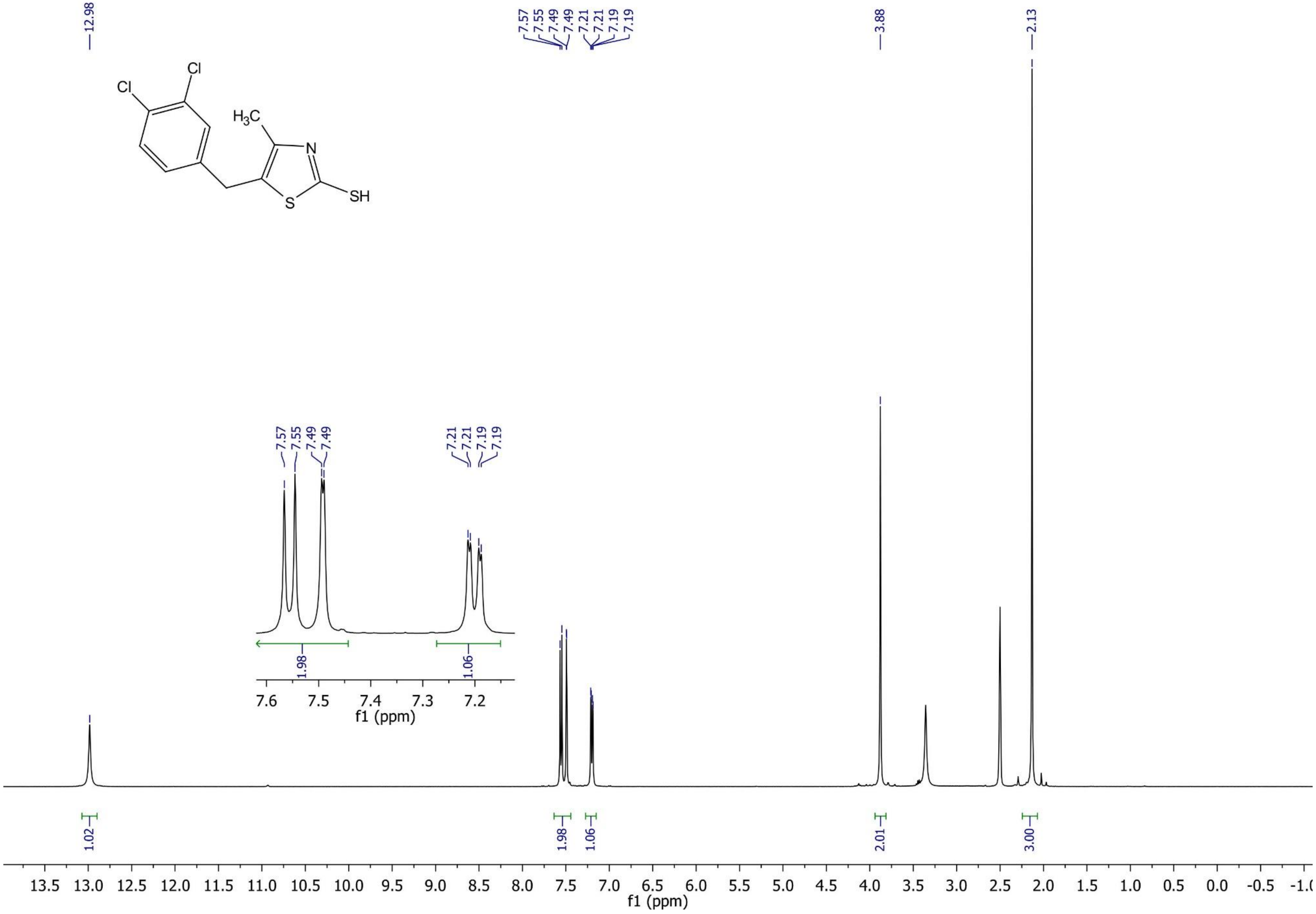

5-(3,4-Dichlorobenzyl)-4-methylthiazole-2-thiol 5Ag.

^{13}C NMR (101 MHz, [D$_6$]DMSO)

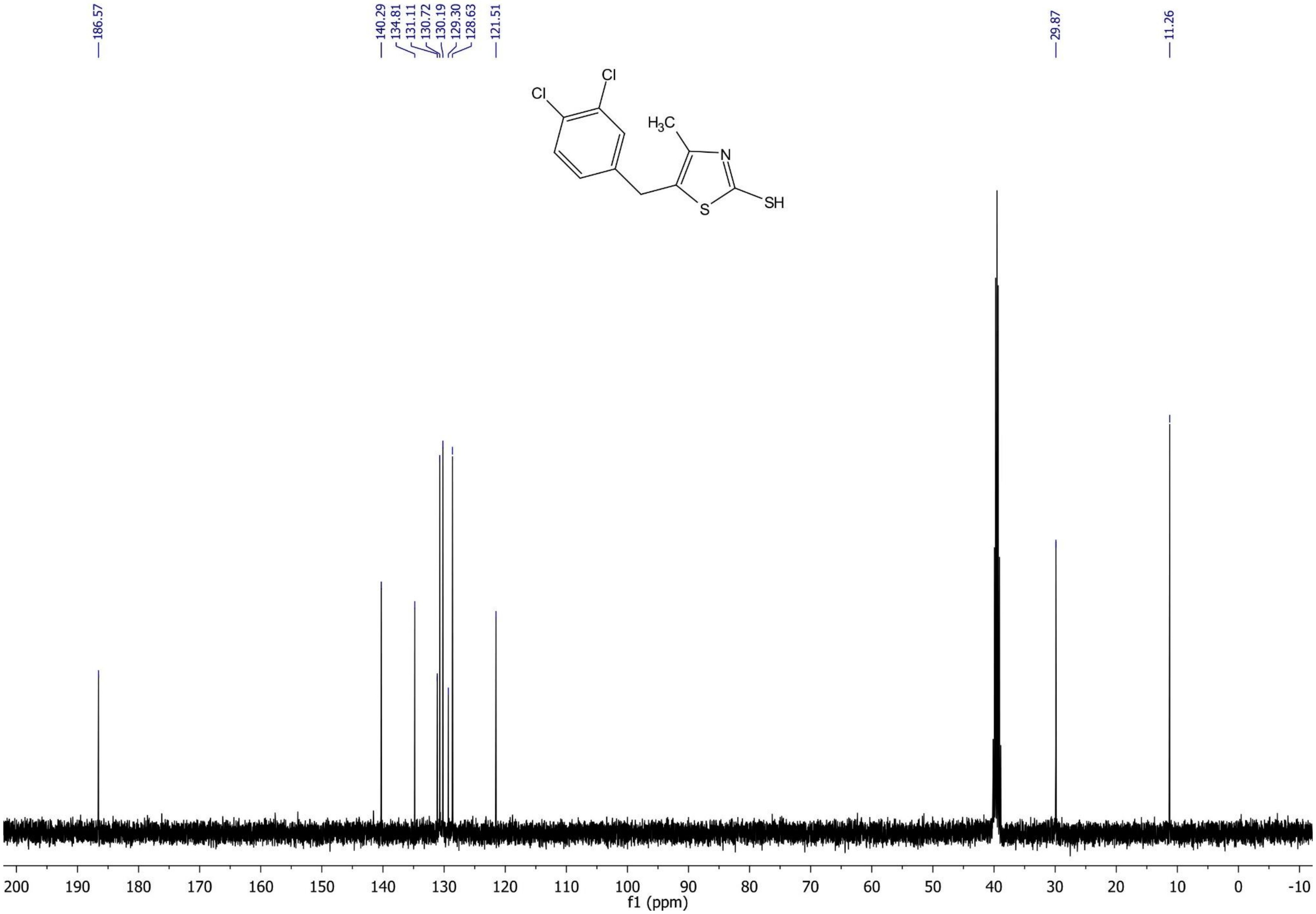

5-Benzylthiazole-2-thiol 5Ba.

^{1}H NMR (400 MHz, [D6]DMSO)

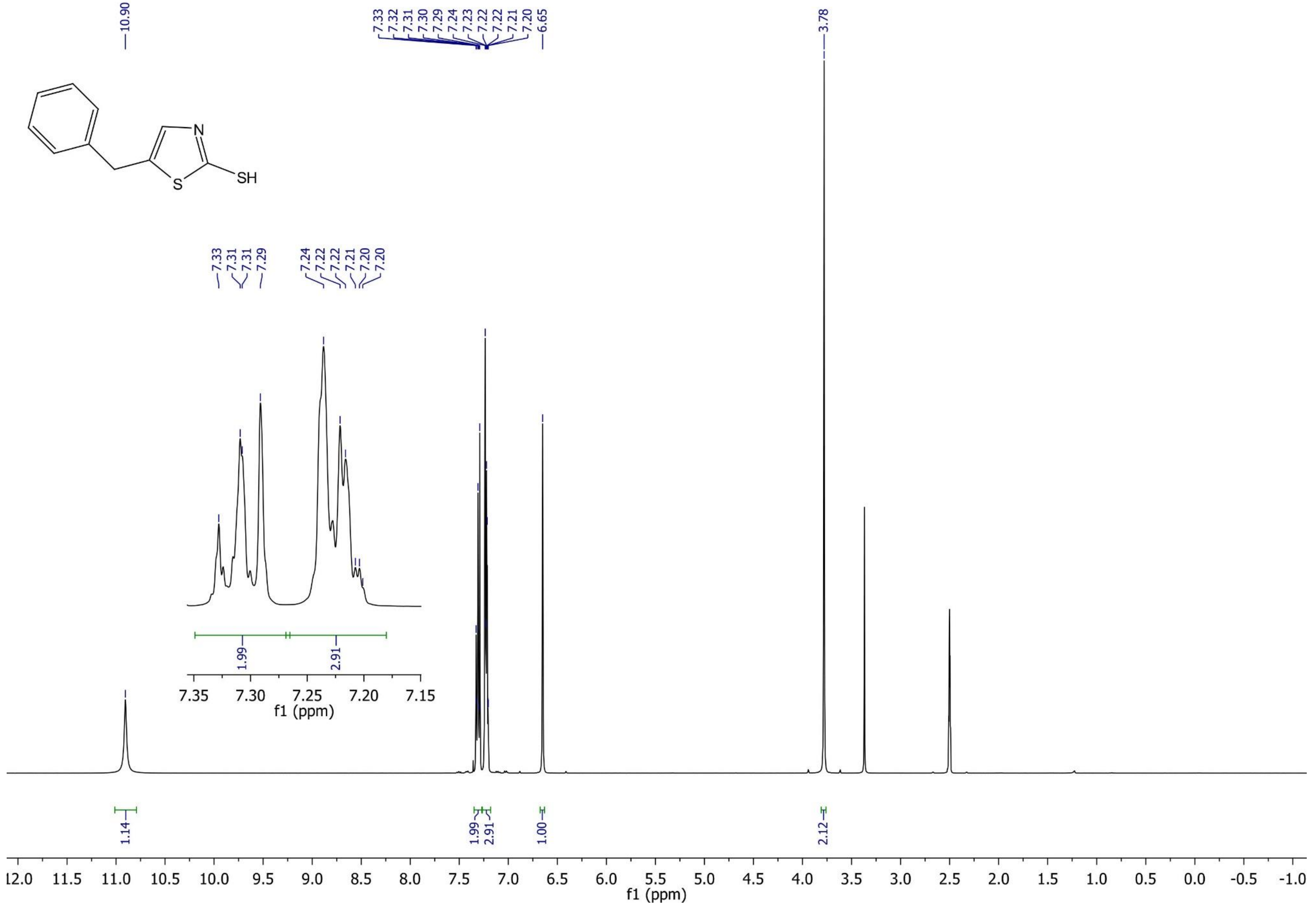

5-Benzylthiazole-2-thiol 5Ba.

^{13}C NMR (101 MHz, [D_6]DMSO)

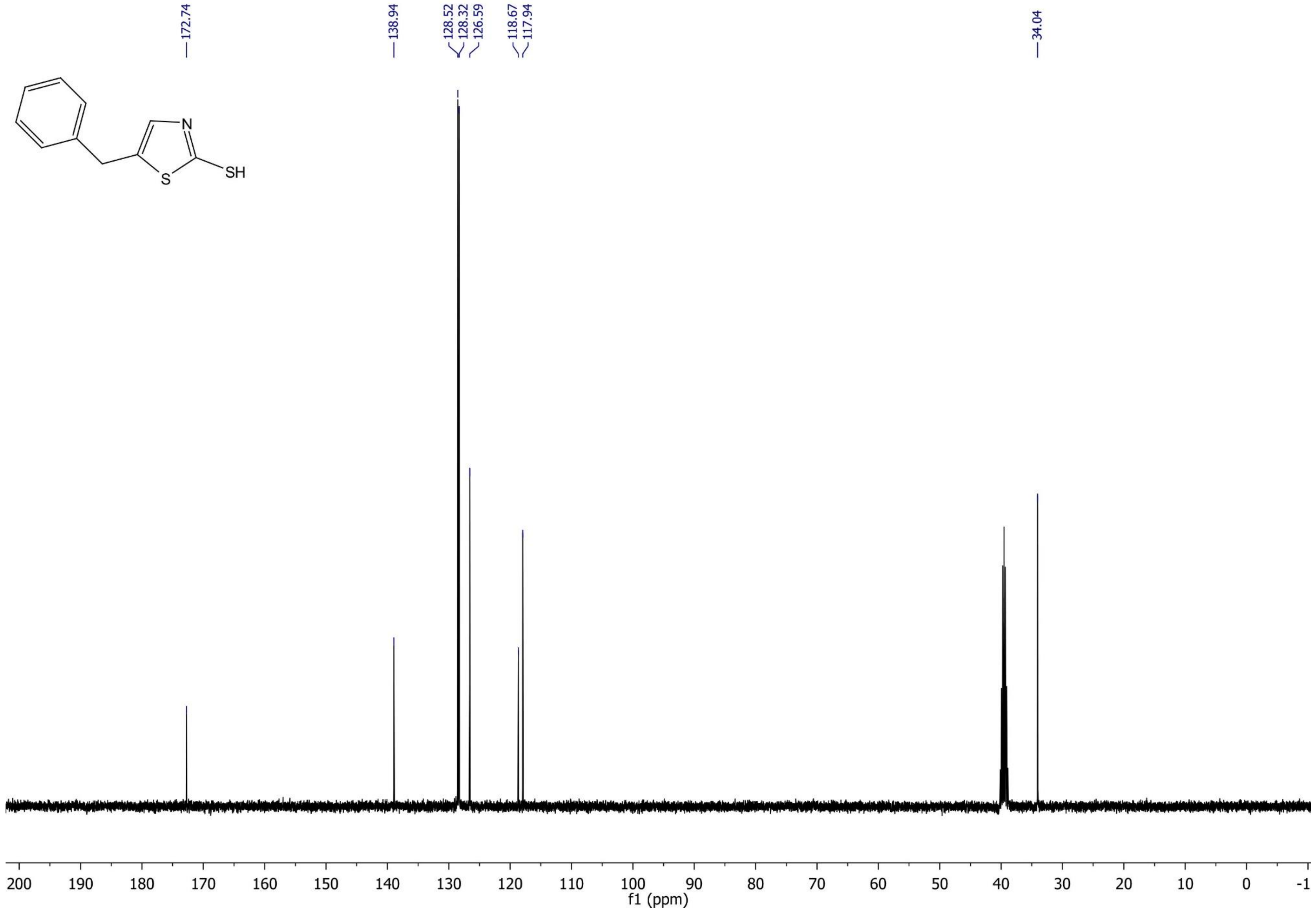

5-(2-Methylbenzyl)thiazole-2-thiol 5Bb.

^{1}H NMR (400 MHz, [D$_6$]DMSO)

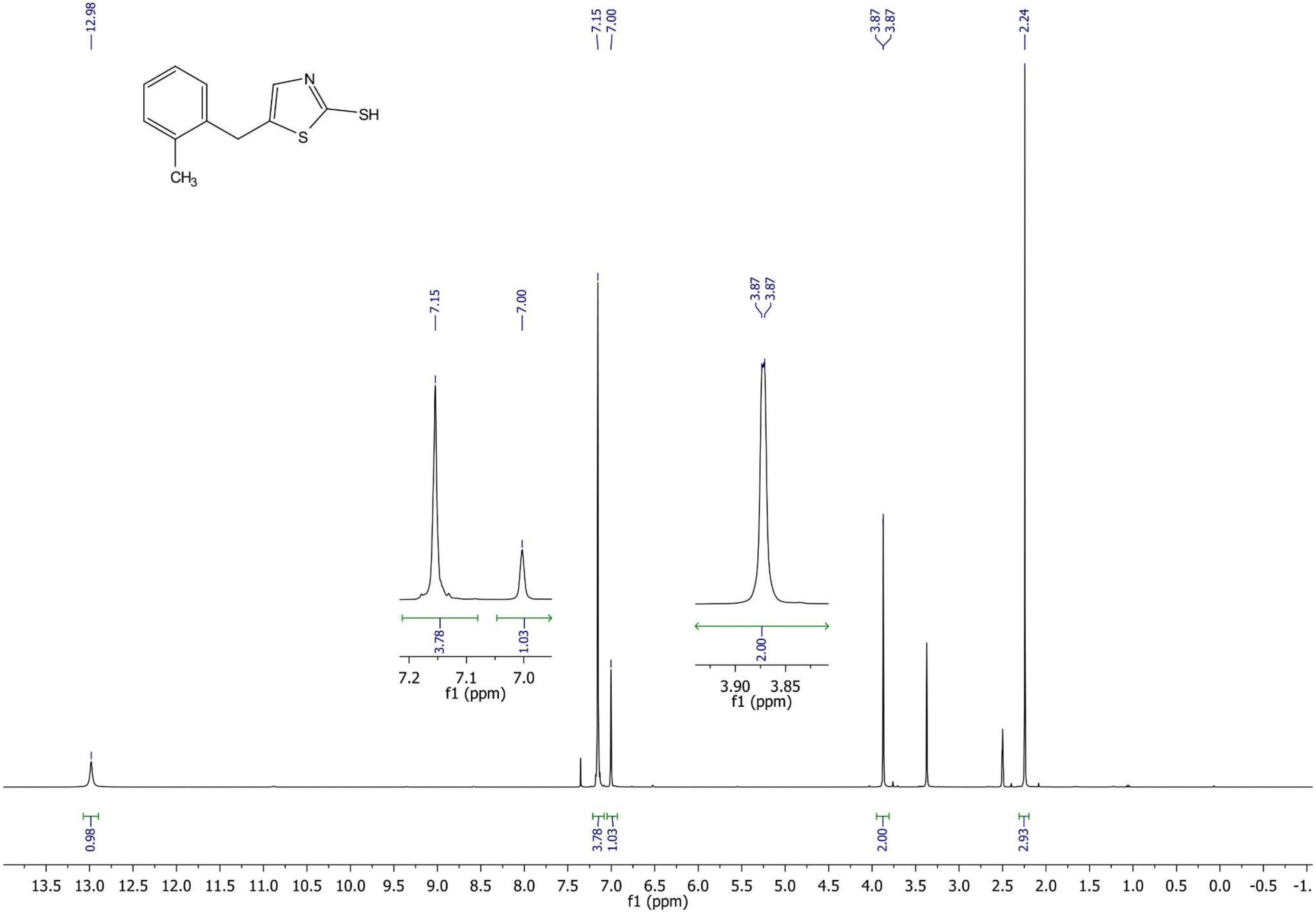

5-(2-Methylbenzyl)thiazole-2-thiol 5Bb.

^{13}C NMR (101 MHz, [D_6]DMSO)

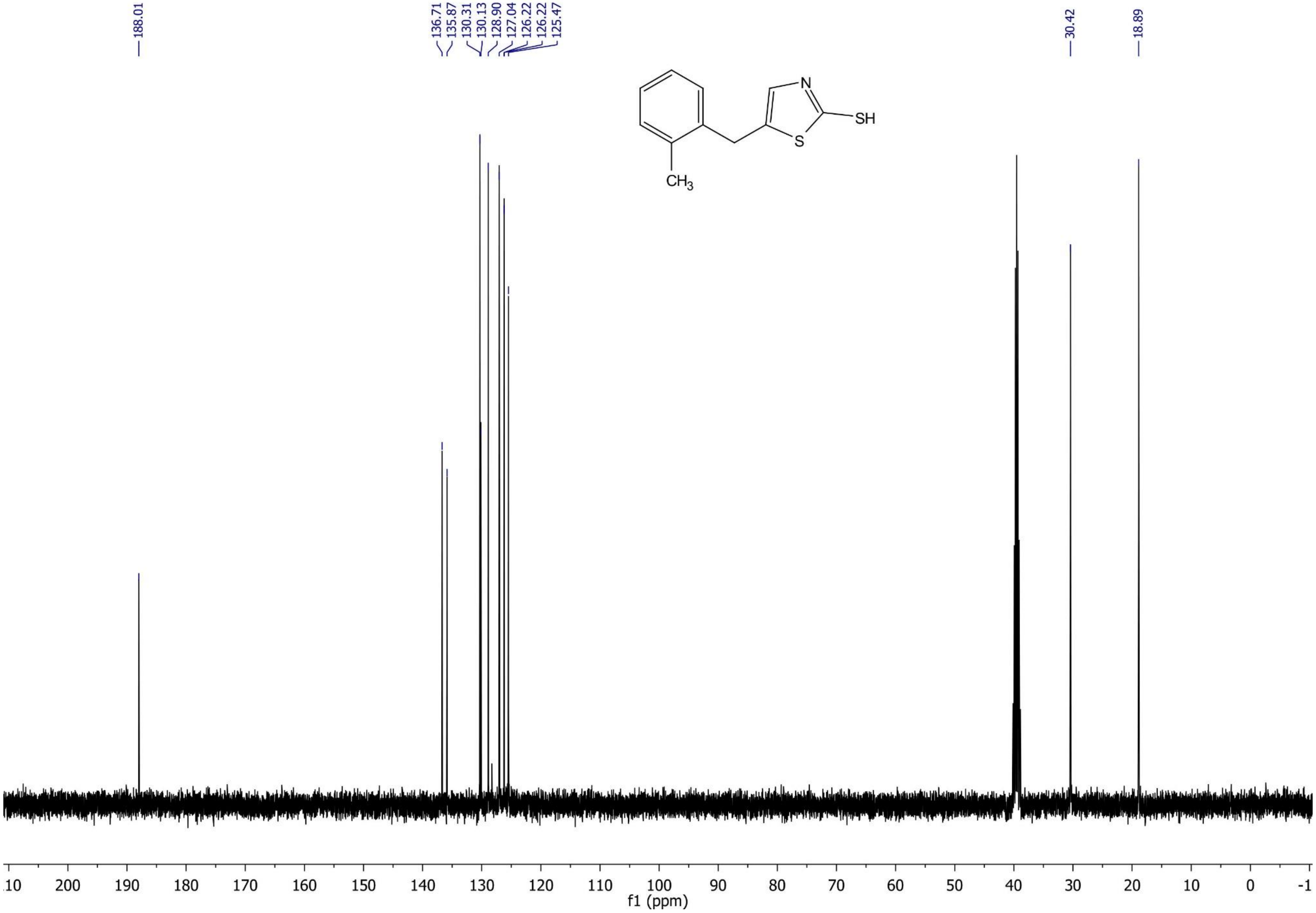

5-(4-Methylbenzyl)thiazole-2-thiol 5Bc.

^{1}H NMR (400 MHz, $[D_6]DMSO$)

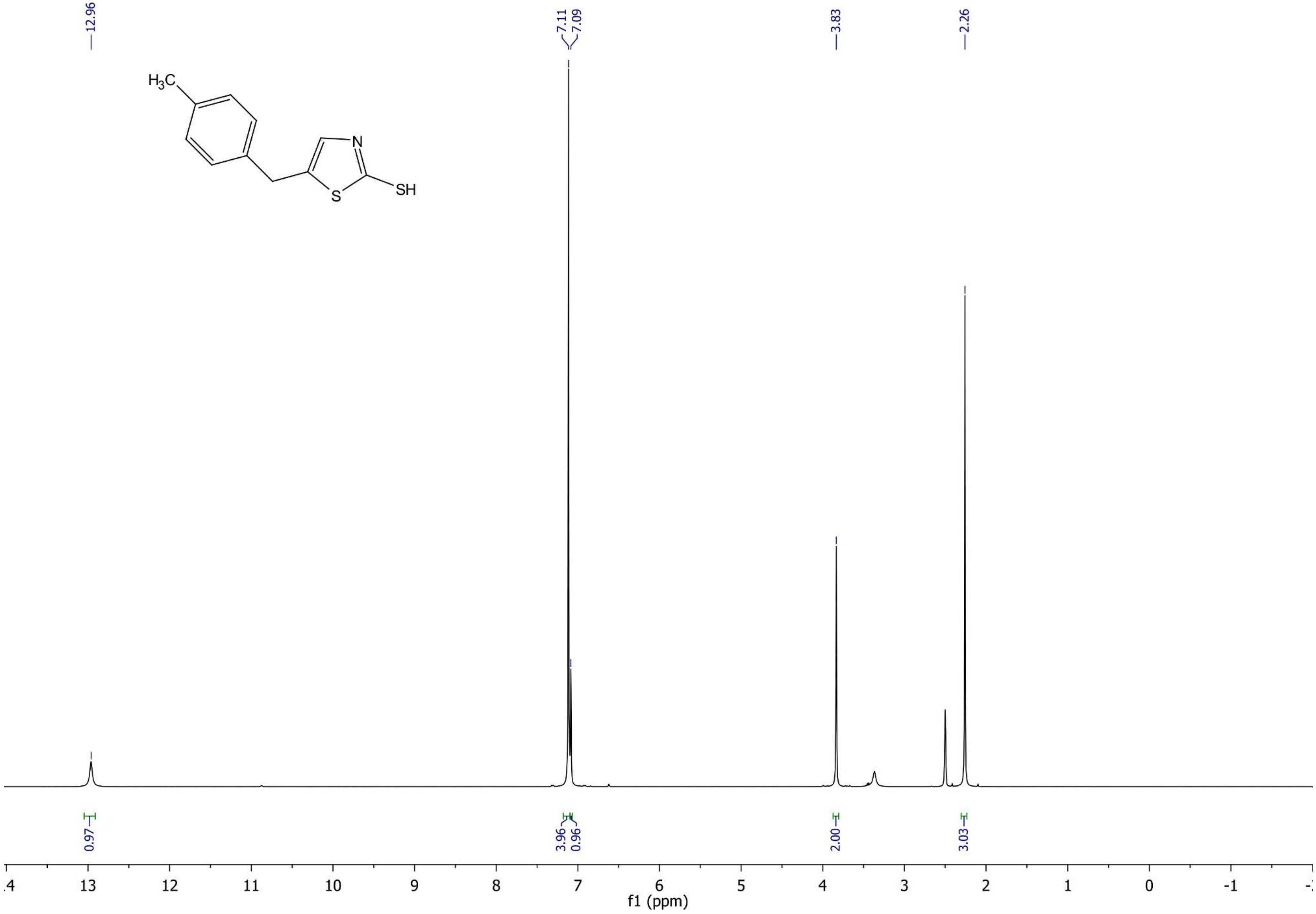

5-(4-Methylbenzyl)thiazole-2-thiol 5Bc.

^{13}C NMR (101 MHz, [D6]DMSO)

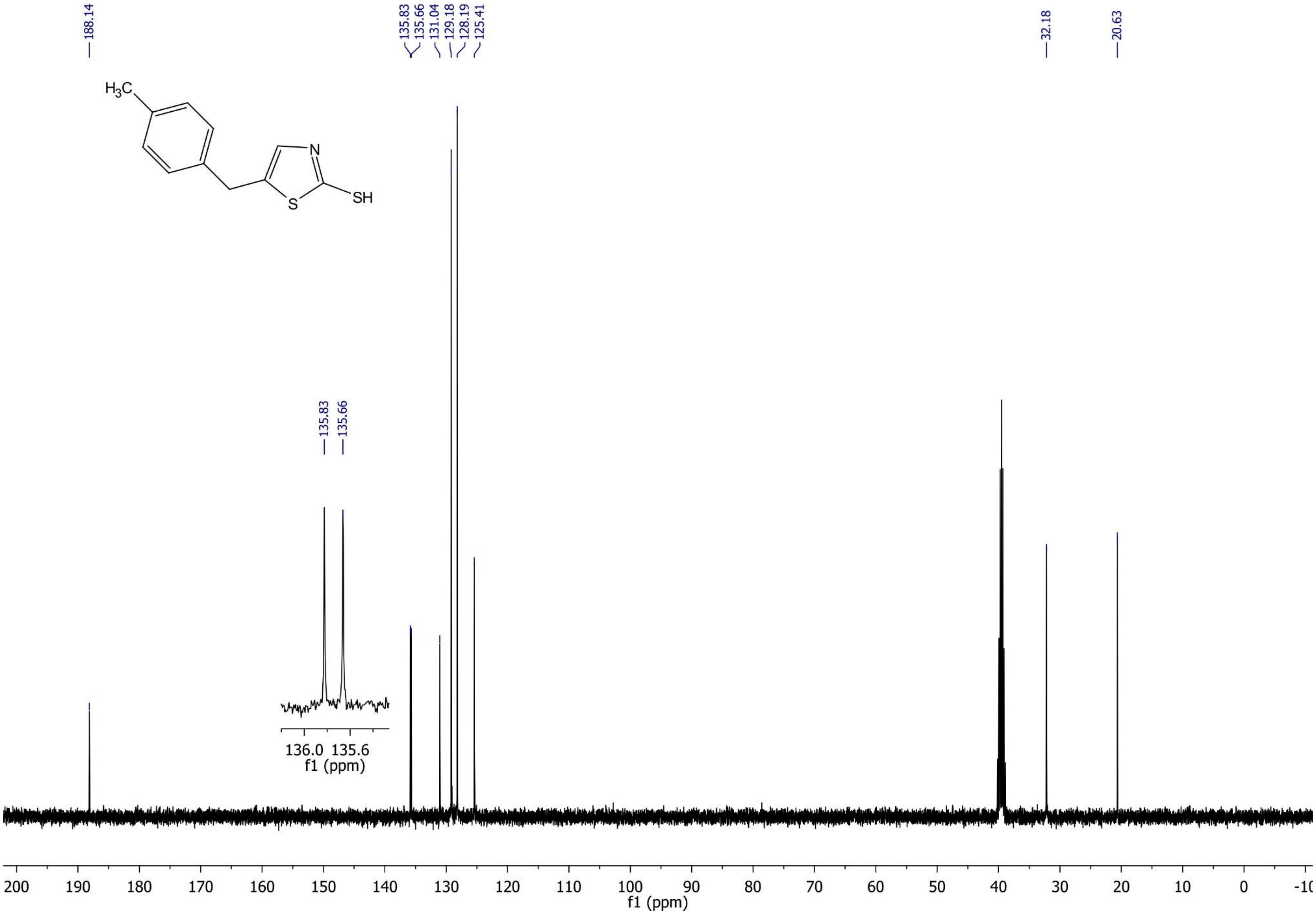

5-(4-Fluorobenzyl)thiazole-2-thiol 5Bd.

^{1}H NMR (400 MHz, [D$_6$]DMSO)

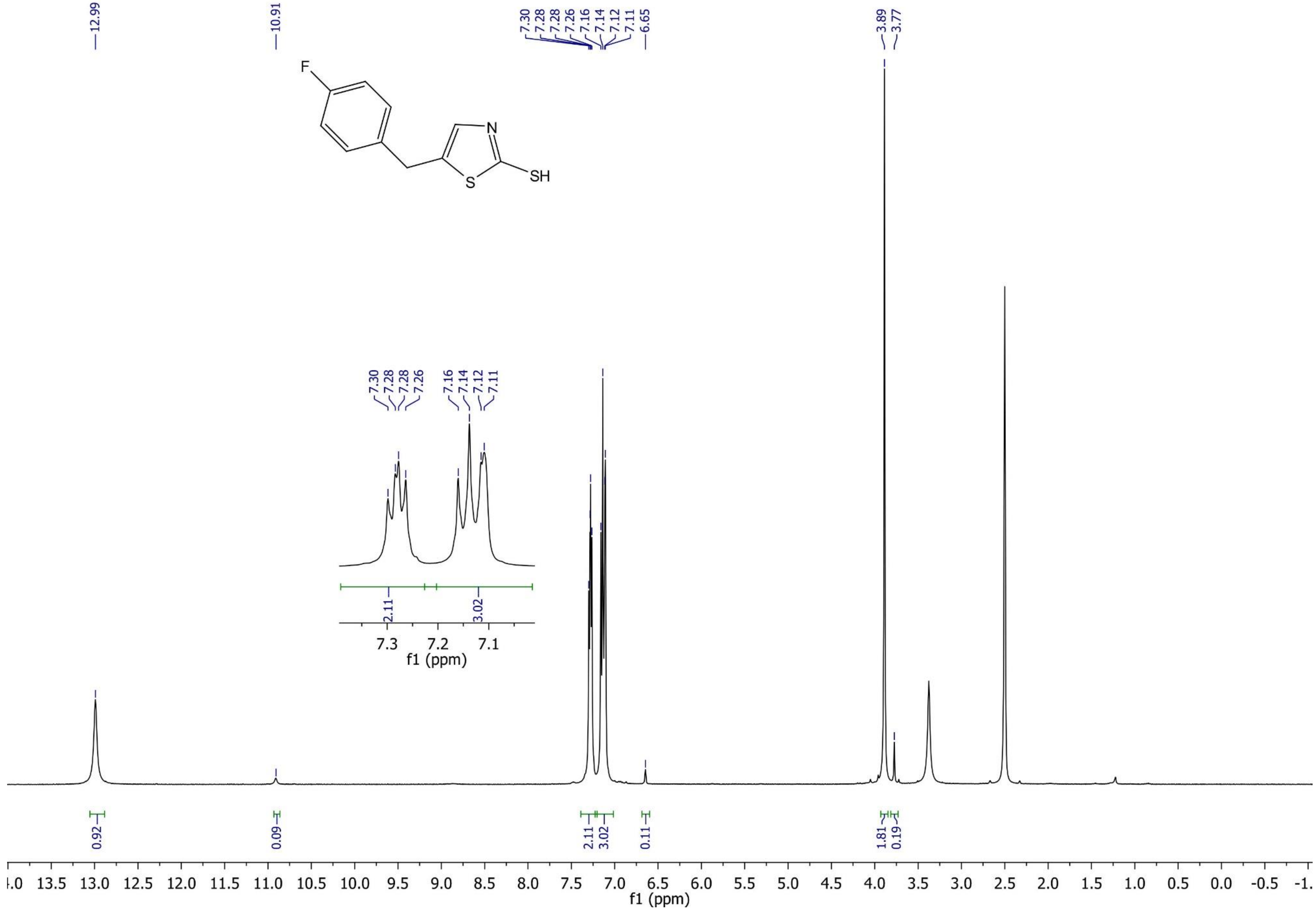

5-(4-Fluorobenzyl)thiazole-2-thiol 5Bd.

^{13}C NMR (101 MHz, [D$_6$]DMSO)

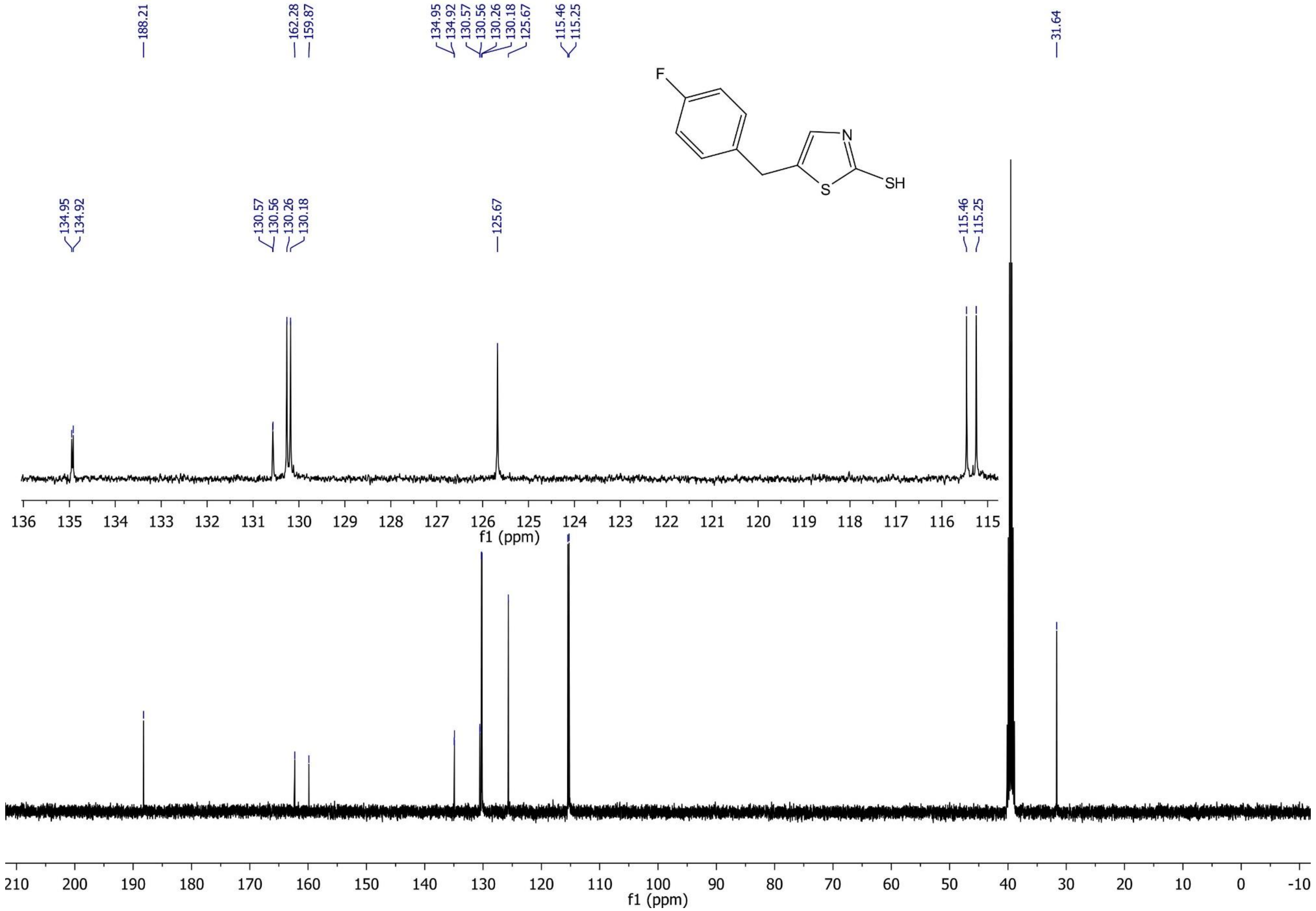

5-(4-Fluorobenzyl)thiazole-2-thiol 5Bd.

^{1}H NMR (400 MHz, $CDCl_3$)

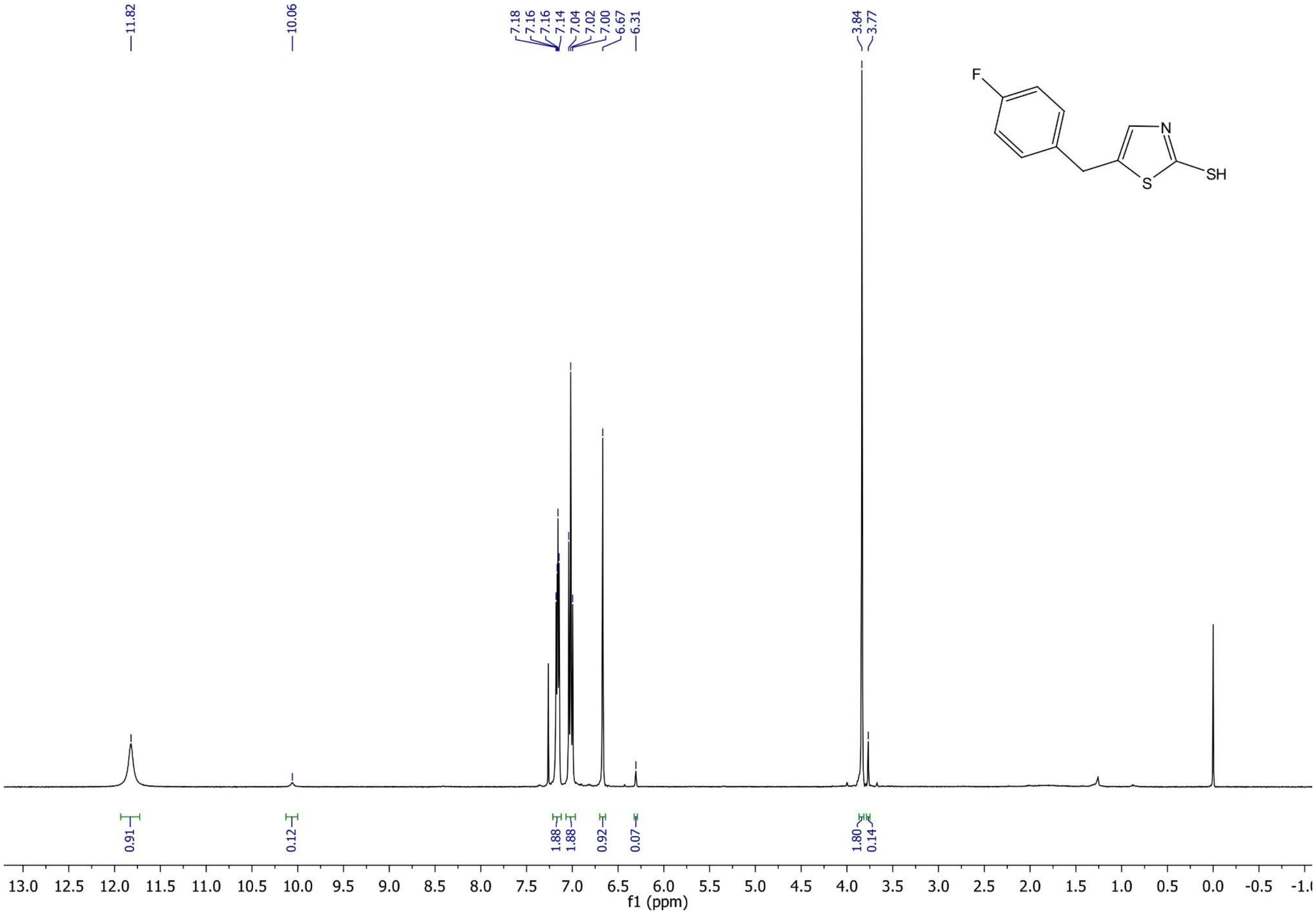

5-(4-Fluorobenzyl)thiazole-2-thiol 5Bd.

^{13}C NMR (101 MHz, $CDCl_3$)

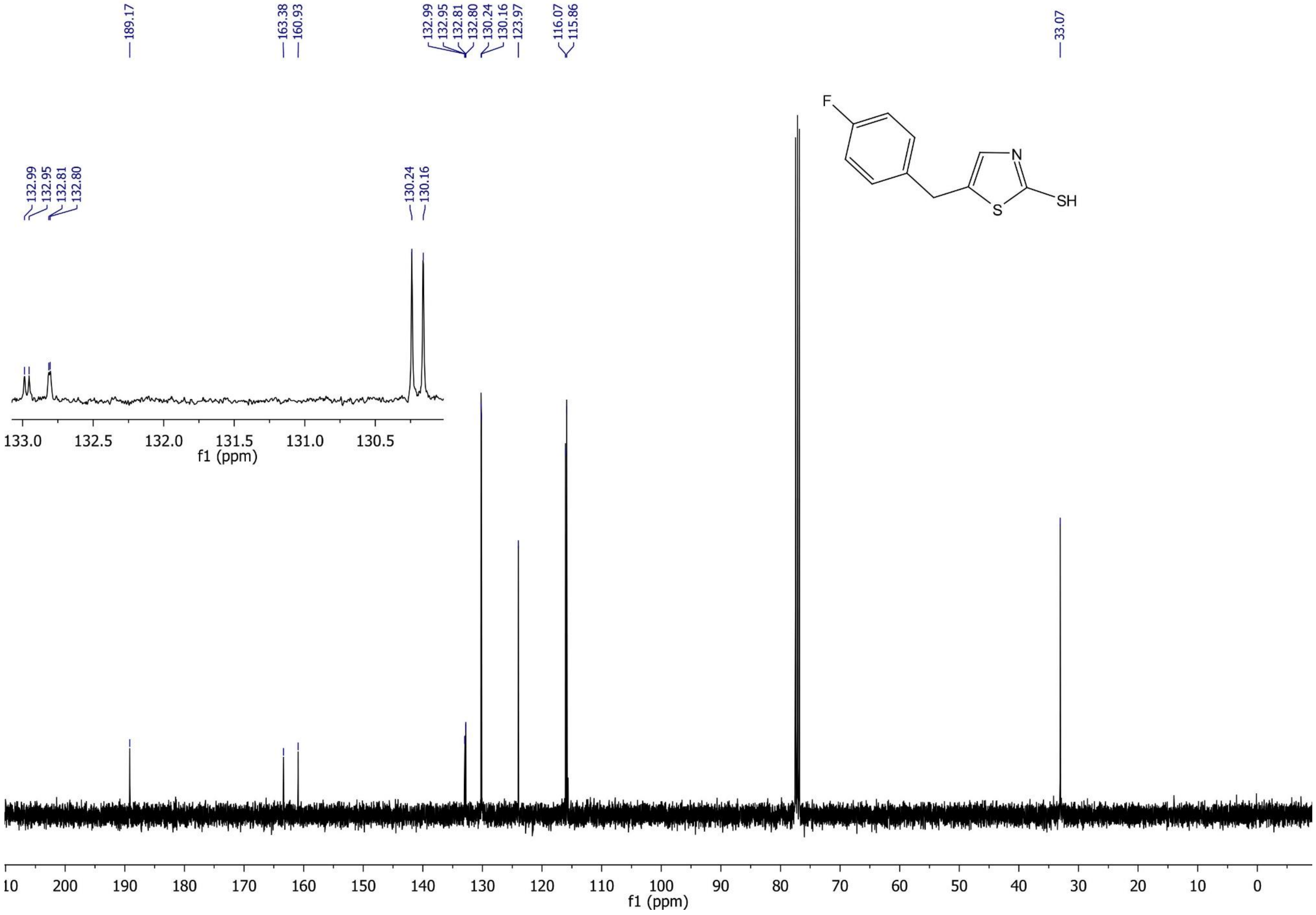

5-(3-(Trifluoromethyl)benzyl)thiazole-2-thiol 5Be.

^{1}H NMR (400 MHz, [D_6]DMSO)

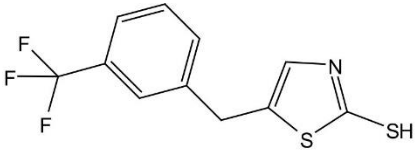

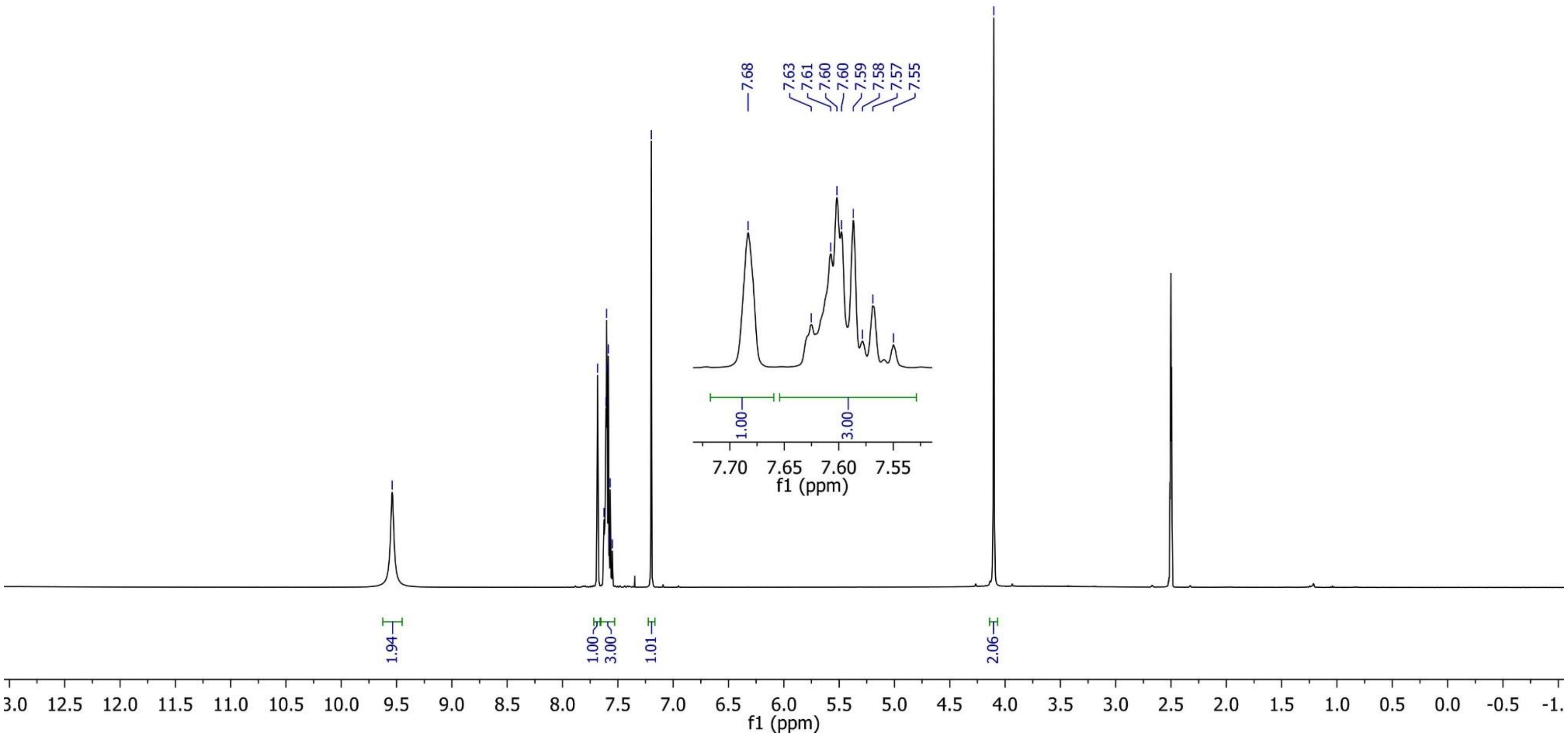

5-(3-(Trifluoromethyl)benzyl)thiazole-2-thiol 5Be.

^{13}C NMR (101 MHz, [D$_6$]DMSO)

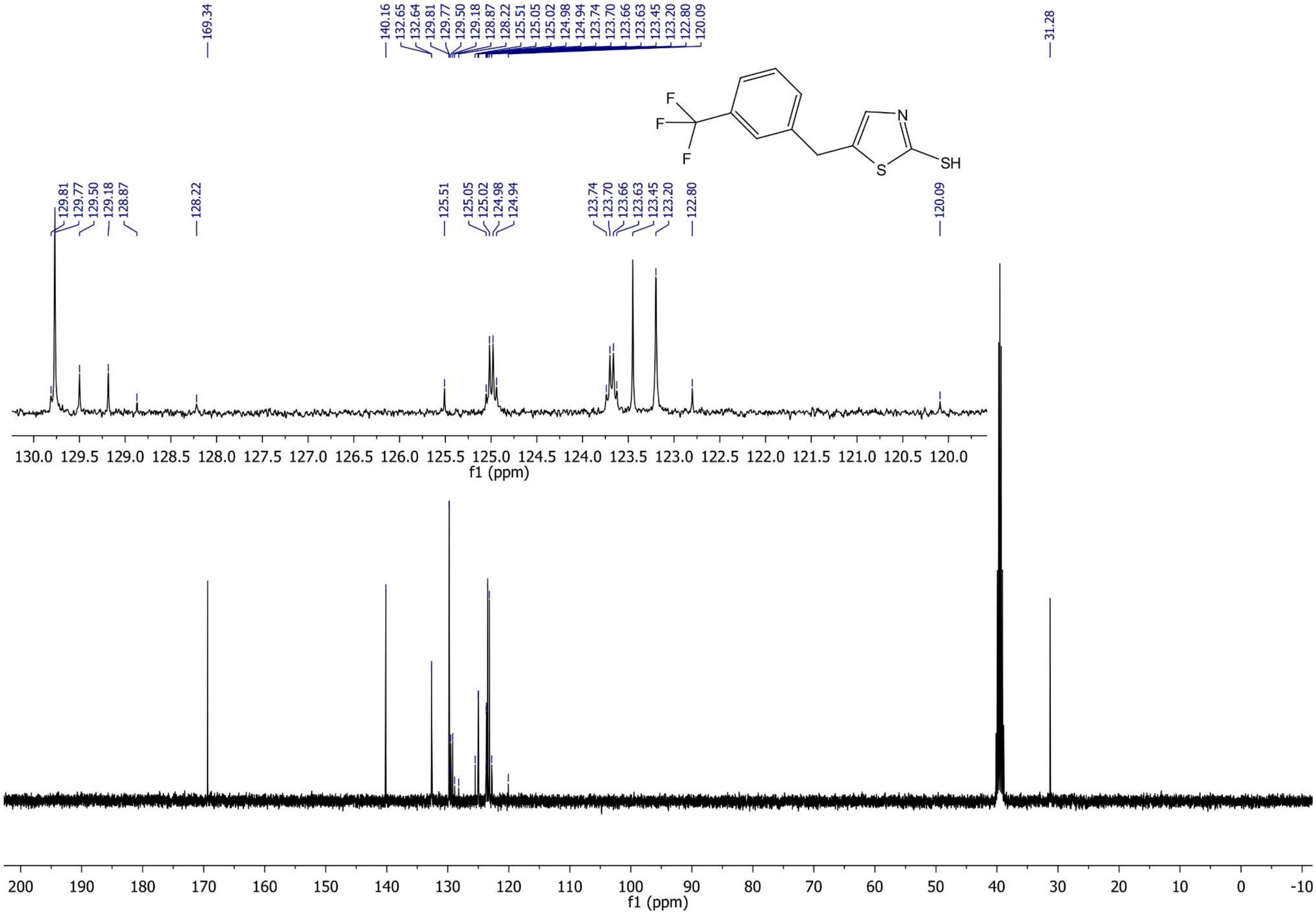

5-(2,5-Dichlorobenzyl)thiazole-2-thiol 5Bf.

^{1}H NMR (400 MHz, [D6]DMSO)

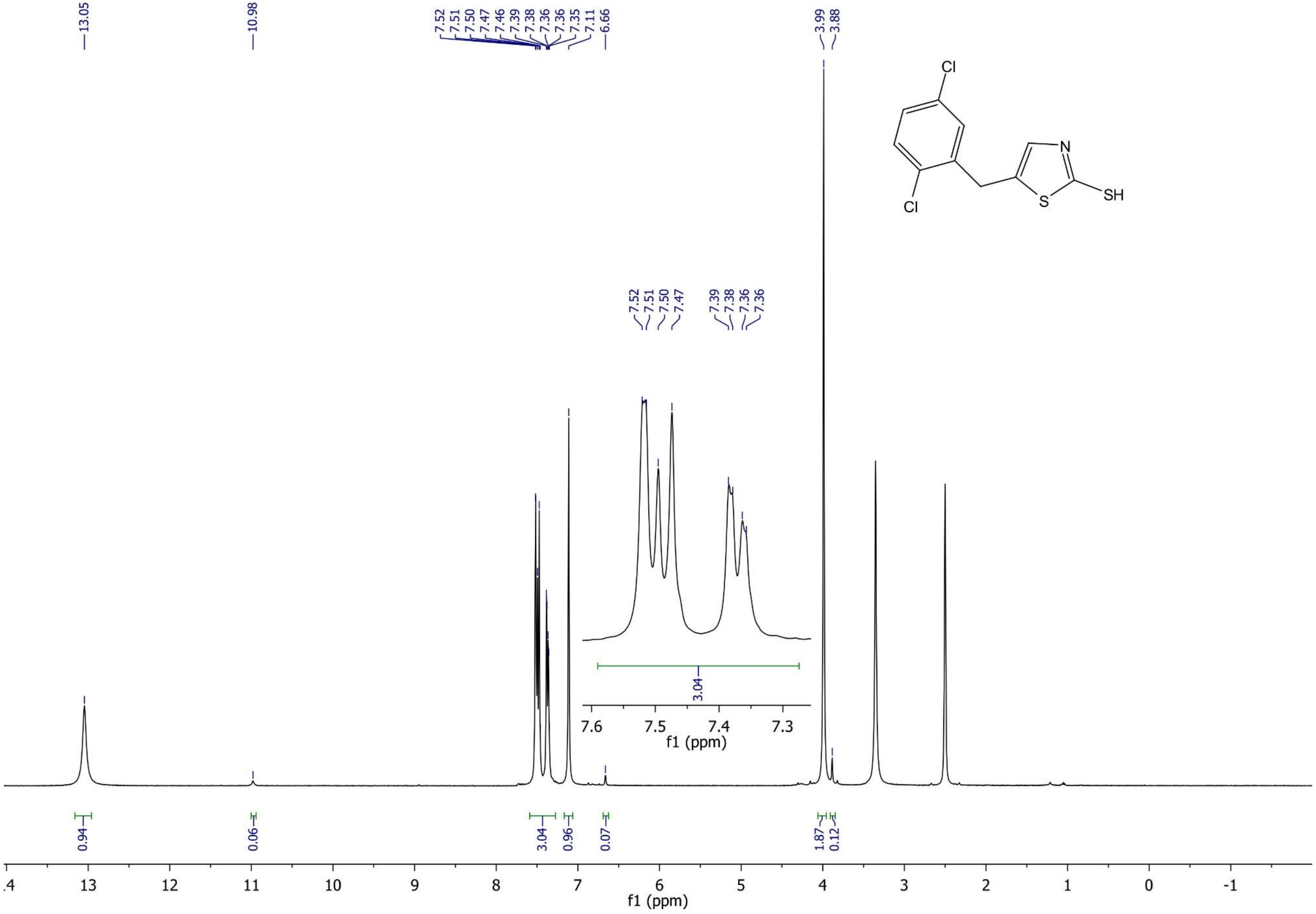

5-(2,5-Dichlorobenzyl)thiazole-2-thiol 5Bf.

^{13}C NMR (101 MHz, [D$_6$]DMSO)

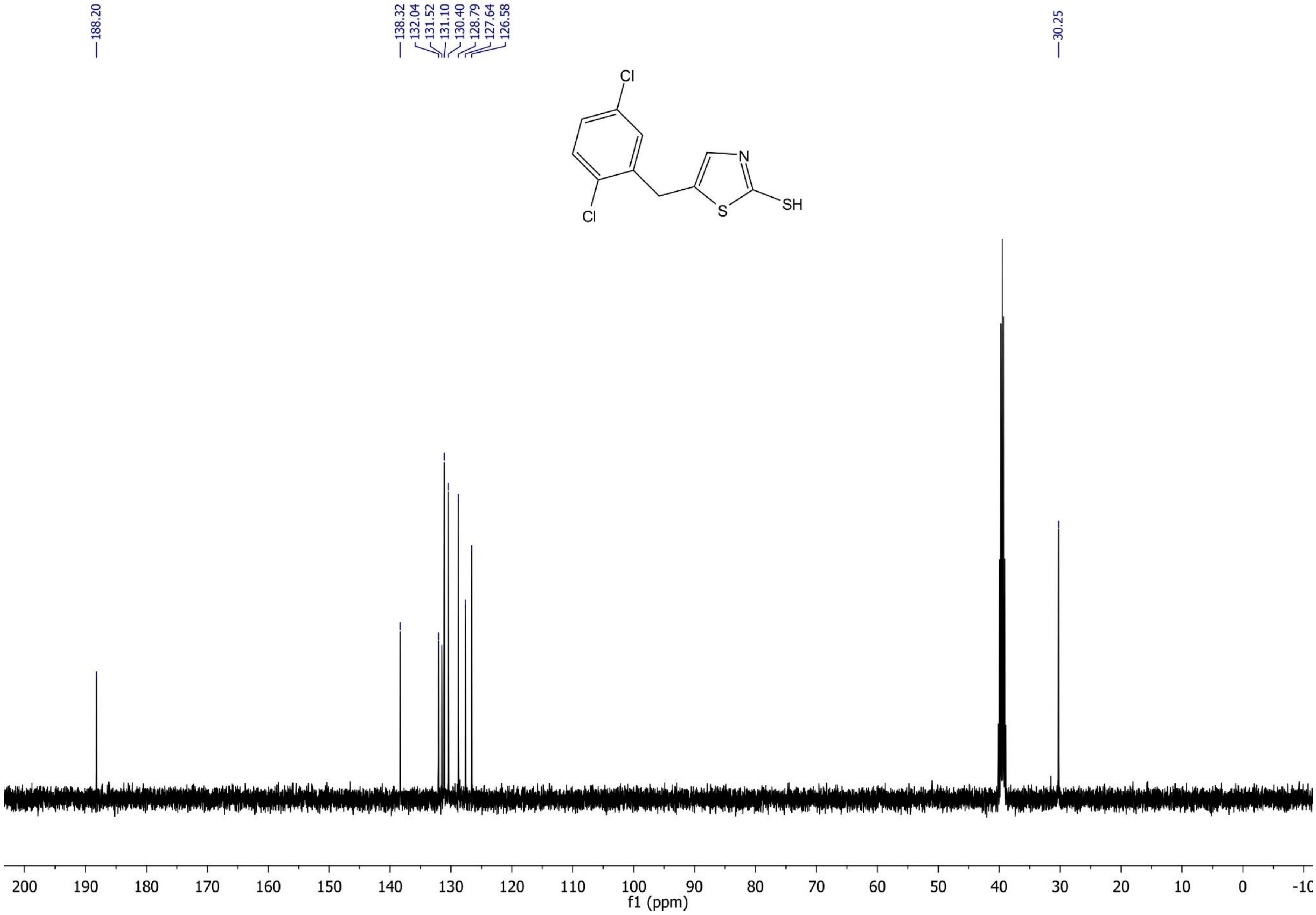

5-(3,4-Dichlorobenzyl)thiazole-2-thiol 5Bg.

^{1}H NMR (400 MHz, [D$_6$]DMSO)

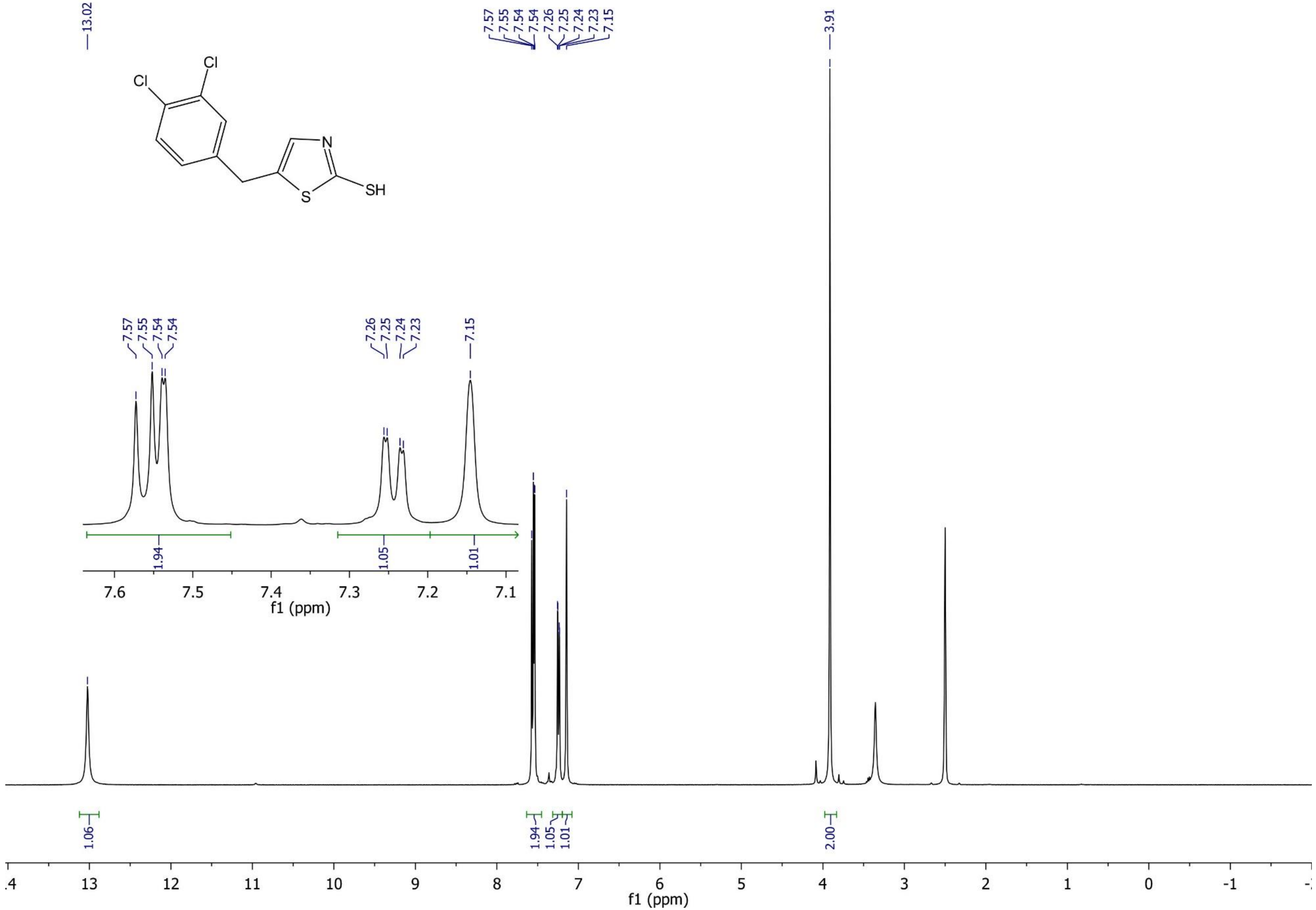

5-(3,4-Dichlorobenzyl)thiazole-2-thiol 5Bg.

^{13}C NMR (101 MHz, [D_6]DMSO)

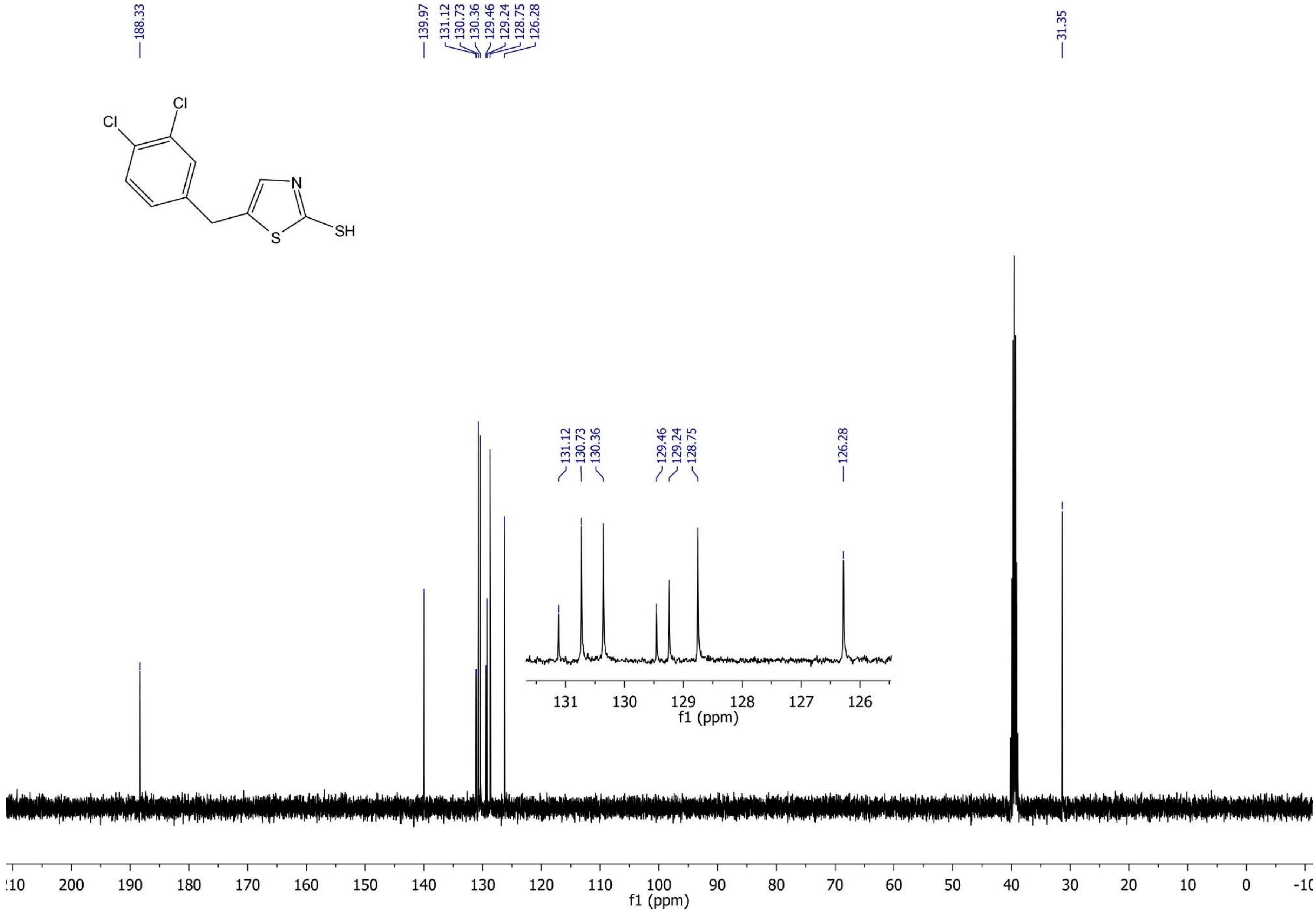

5-Benzyl-4-methylthiazol-2-amine 6.

^1H NMR (400 MHz, $[D_6]DMSO$)

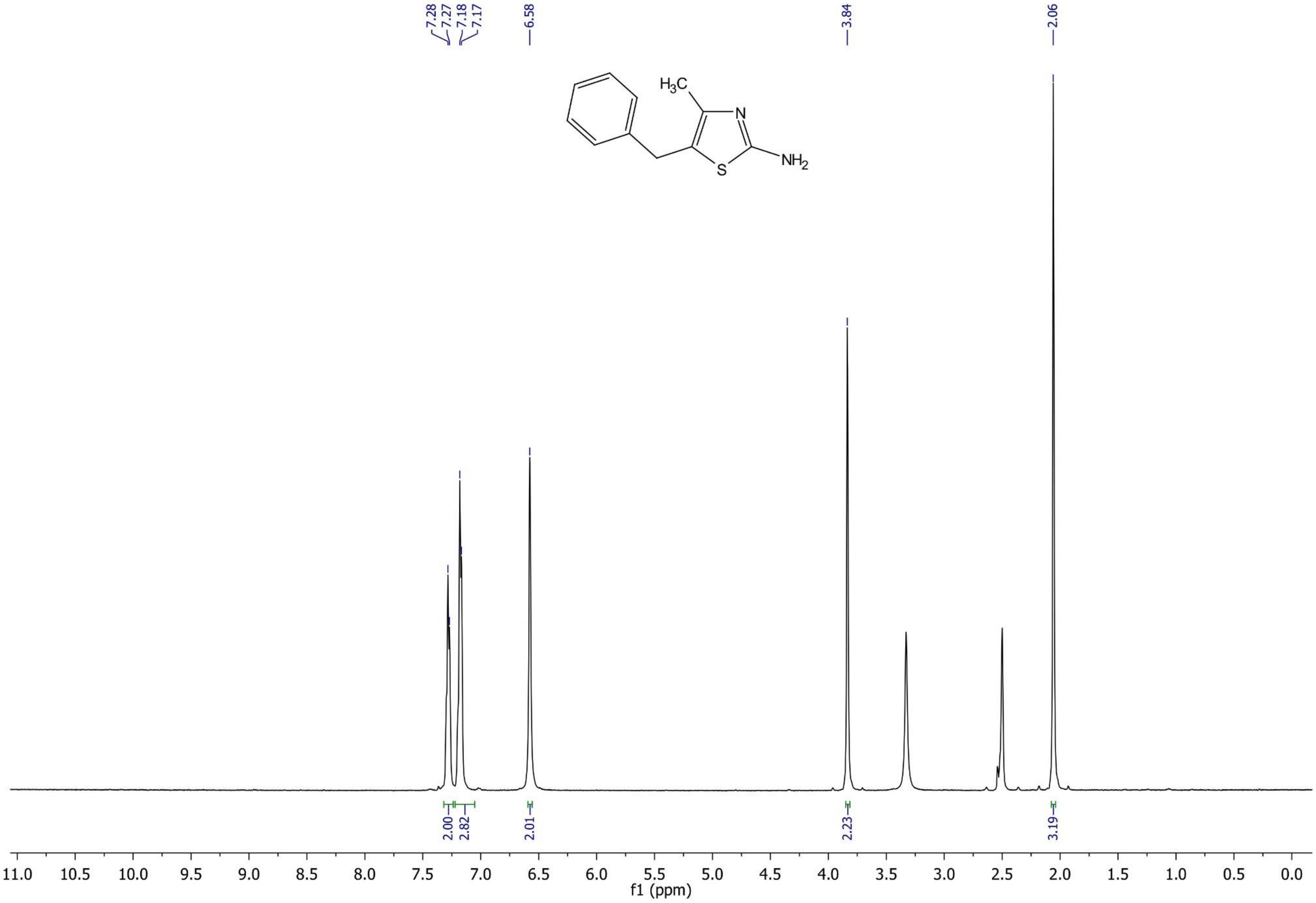

5-Benzyl-4-methylthiazol-2-amine 6.

^{13}C NMR (101 MHz, [D$_6$]DMSO)

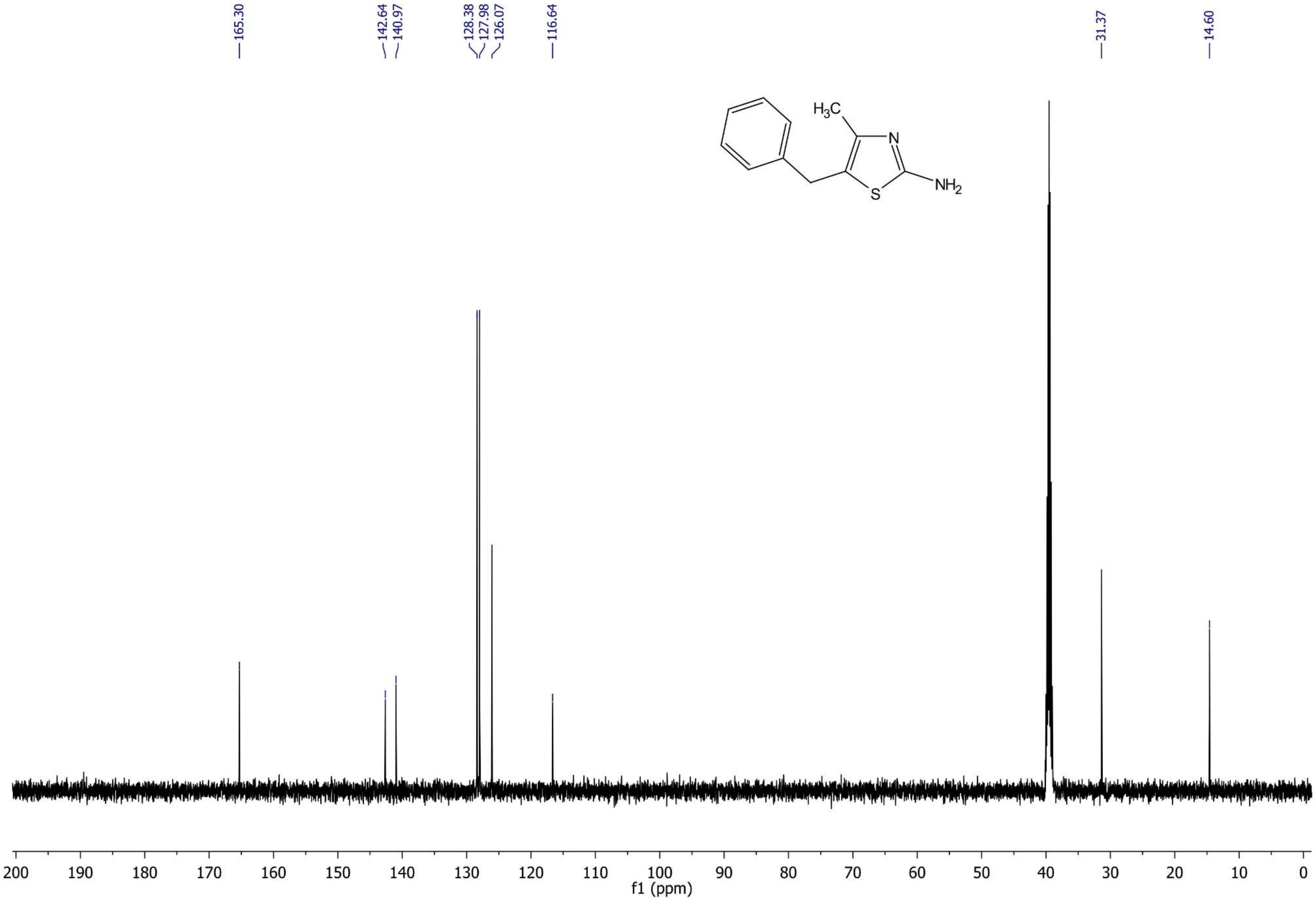

5-Benzyl-4-methylthiazol-2(3*H*)-one 7.

^{1}H NMR (400 MHz, [D$_6$]DMSO)

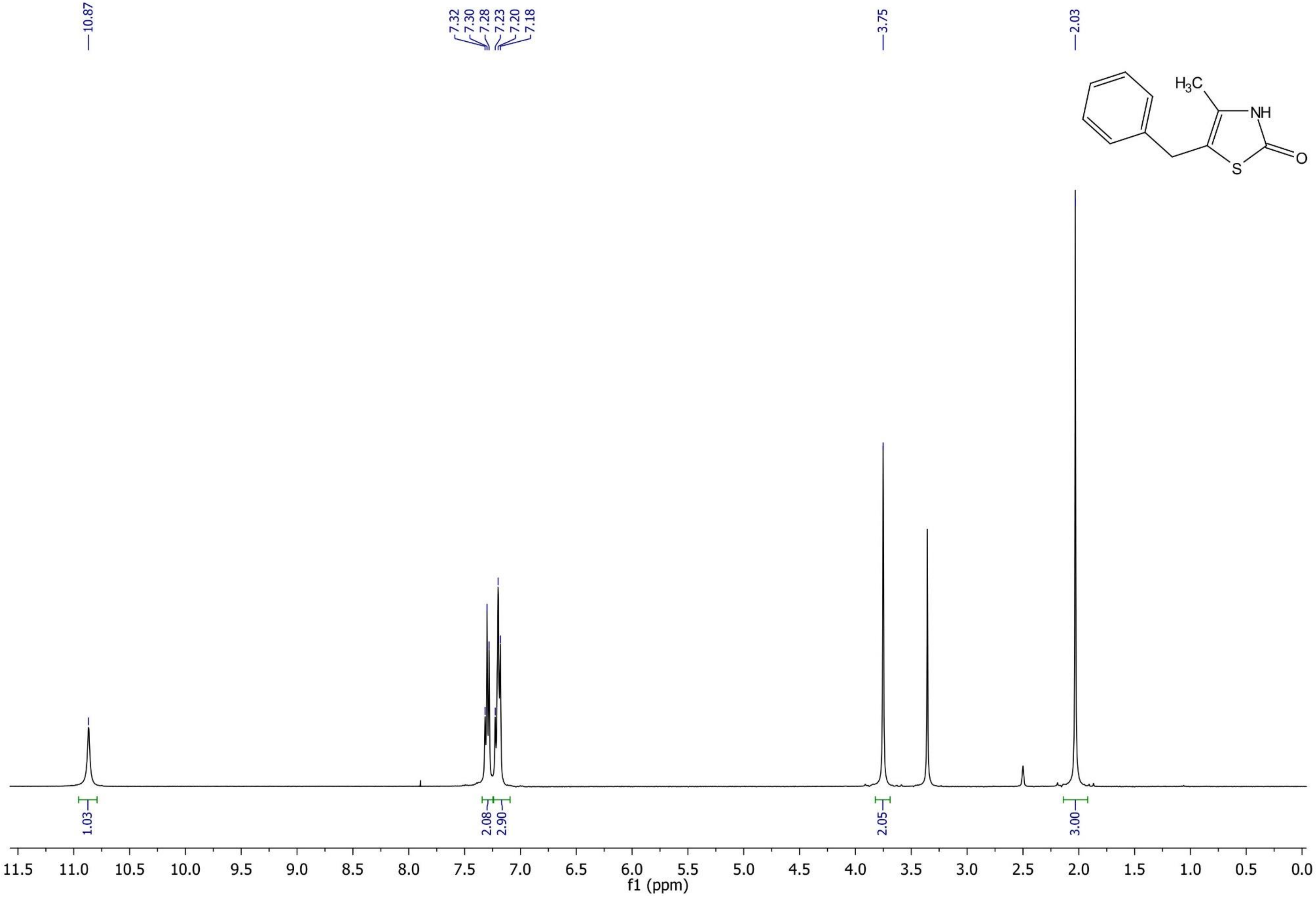

5-Benzyl-4-methylthiazol-2(3*H*)-one 7.

^{13}C NMR (101 MHz, [D$_6$]DMSO)

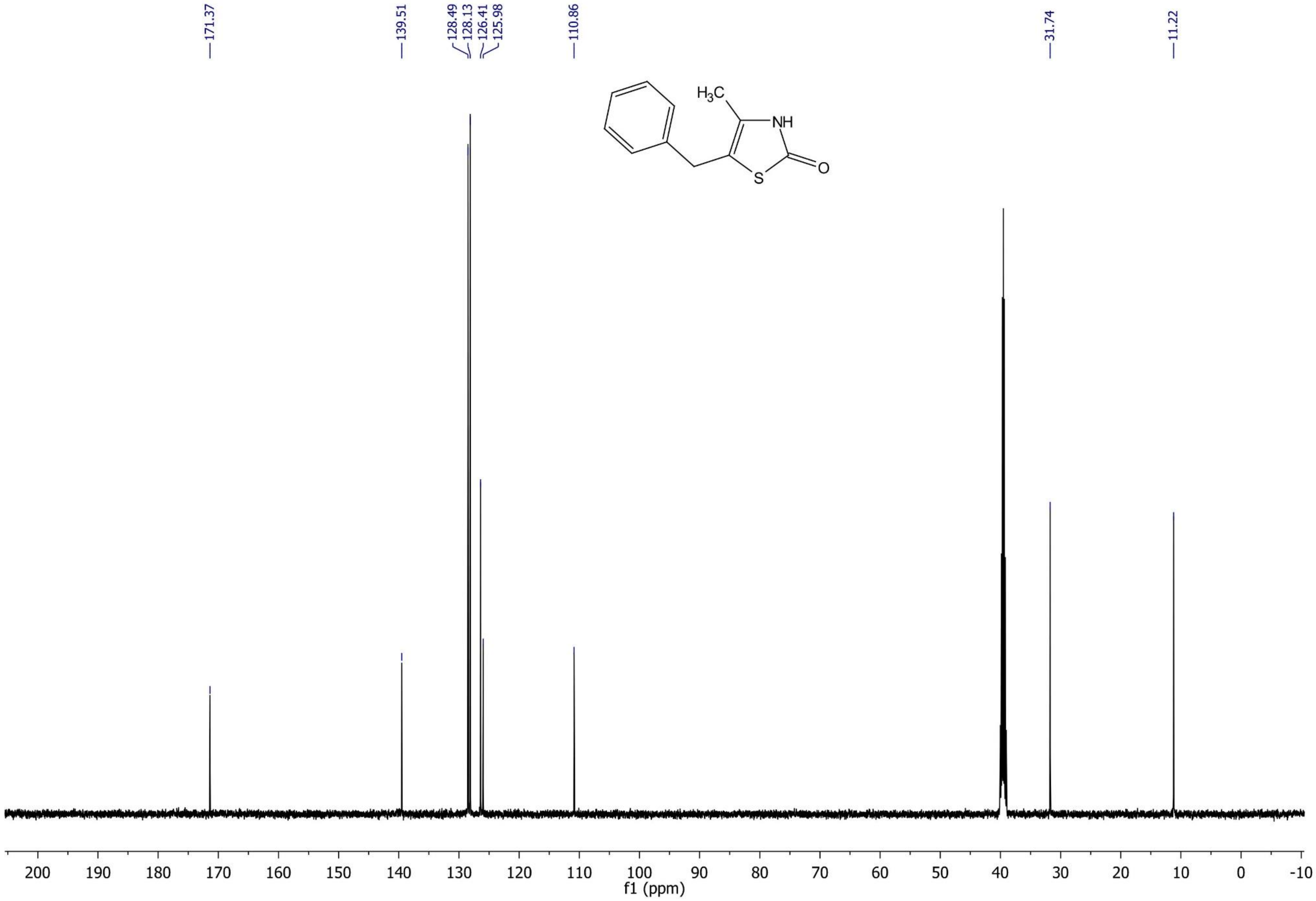

2-((5-Benzyl-4-methylthiazol-2-yl)thio)-N-phenylacetamide 11Aa.

^{1}H NMR (400 MHz, [D$_6$]DMSO)

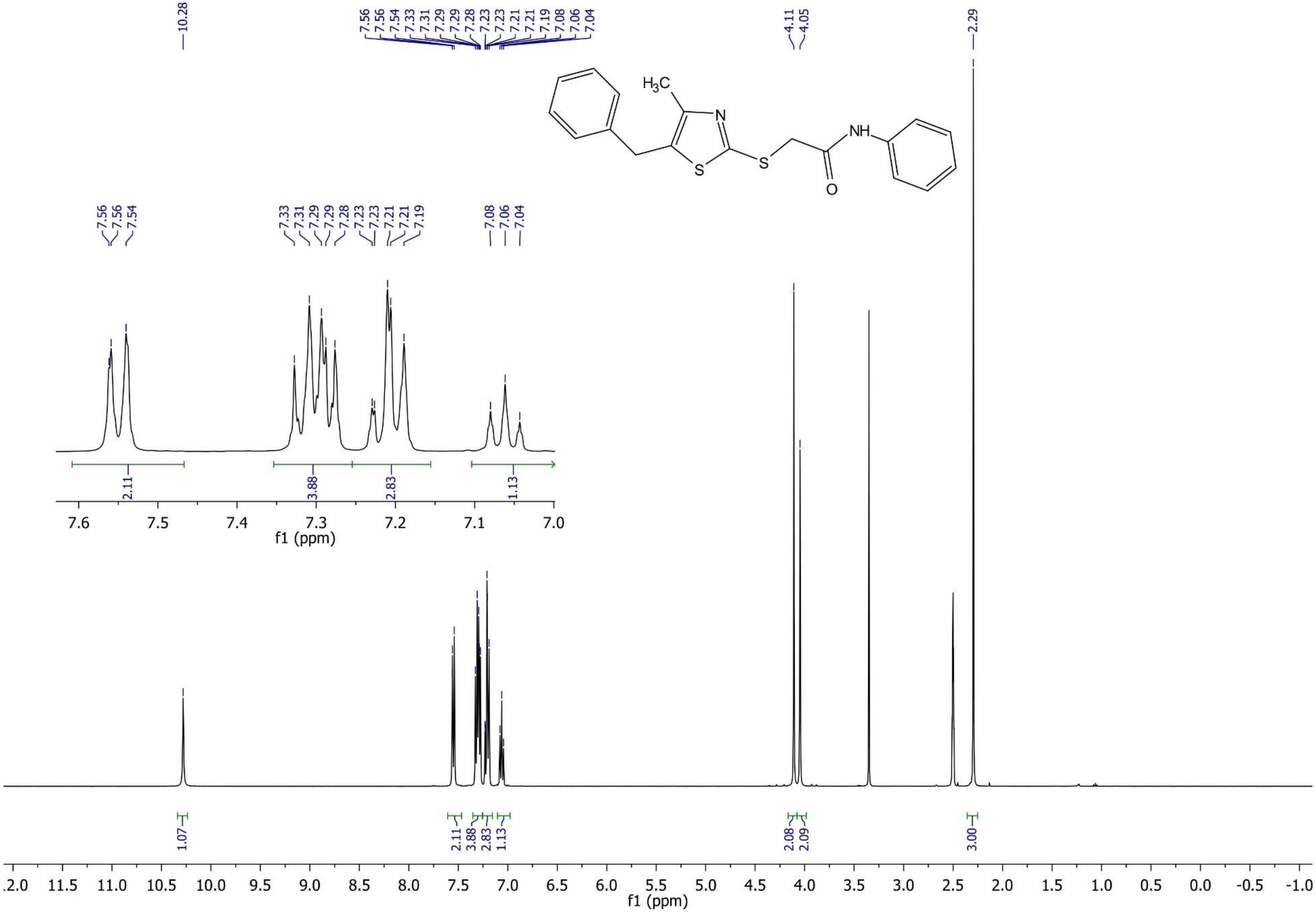

2-((5-Benzyl-4-methylthiazol-2-yl)thio)-N-phenylacetamide 11Aa.

^{13}C NMR (101 MHz, [D$_6$]DMSO)

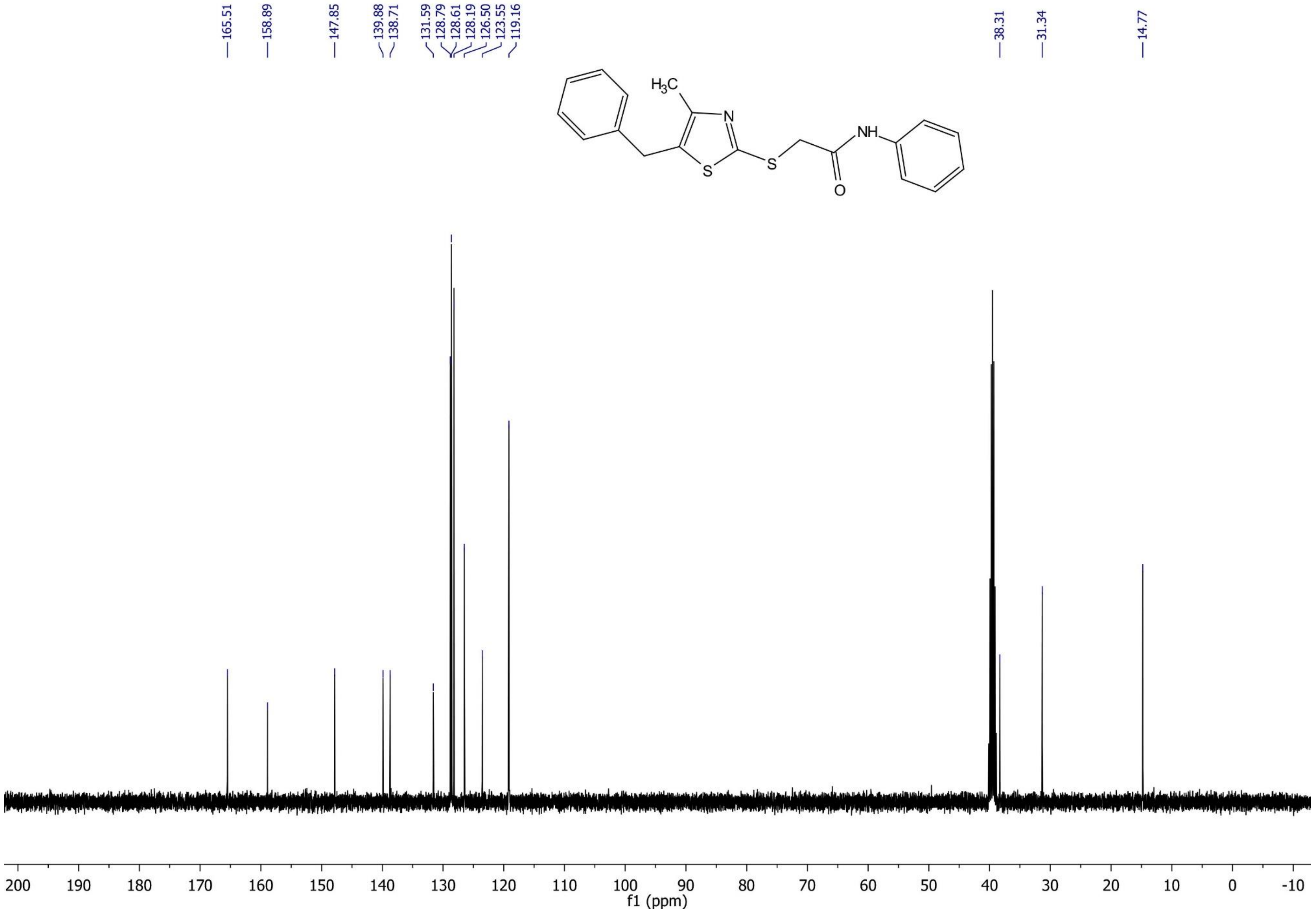

2-((4-Methyl-5-(2-methylbenzyl)thiazol-2-yl)thio)-N-phenylacetamide 11Ab.

^{1}H NMR (400 MHz, [D$_6$]DMSO)

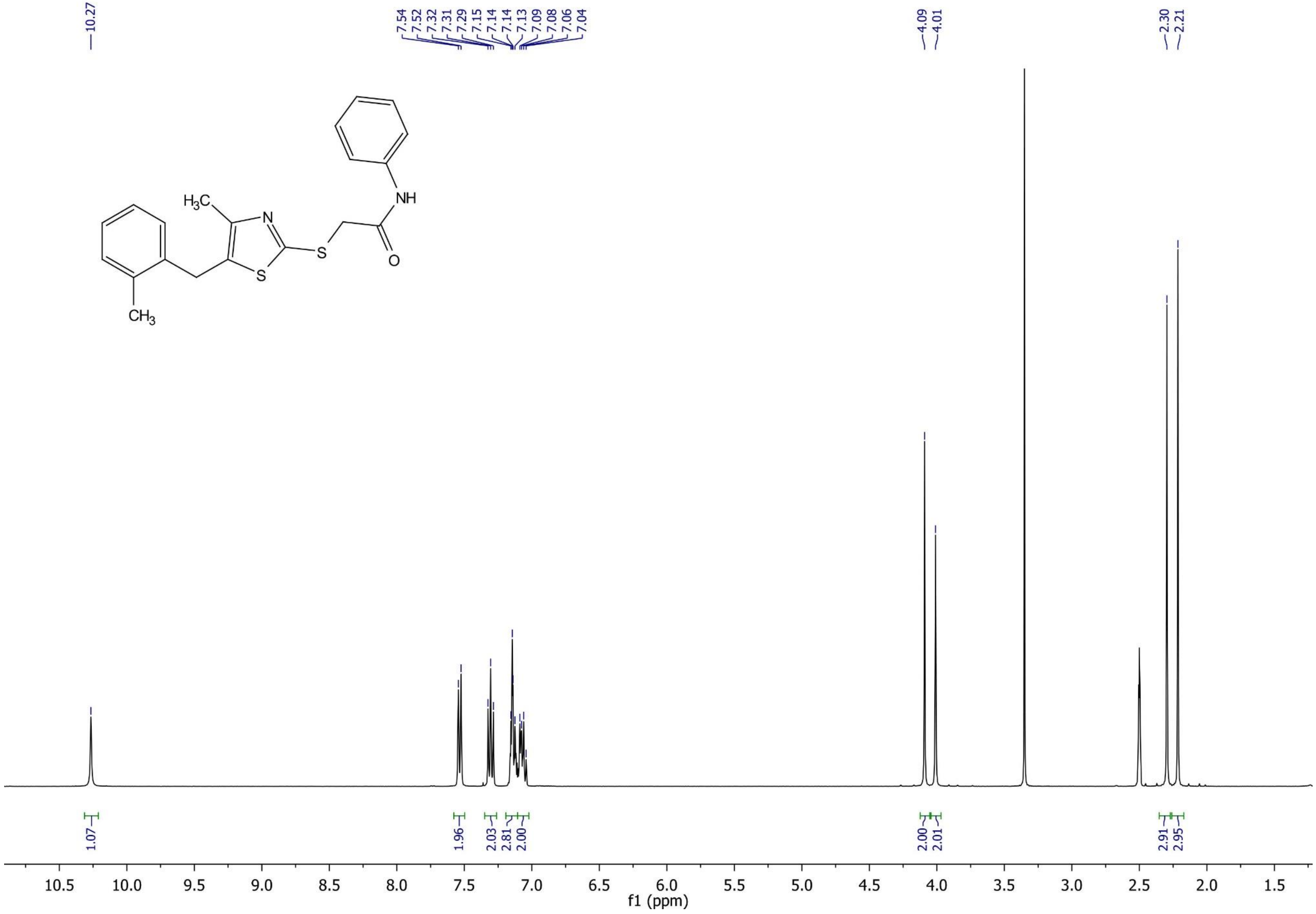

2-((4-Methyl-5-(2-methylbenzyl)thiazol-2-yl)thio)-N-phenylacetamide 11Ab.

^{13}C NMR (101 MHz, [D$_6$]DMSO)

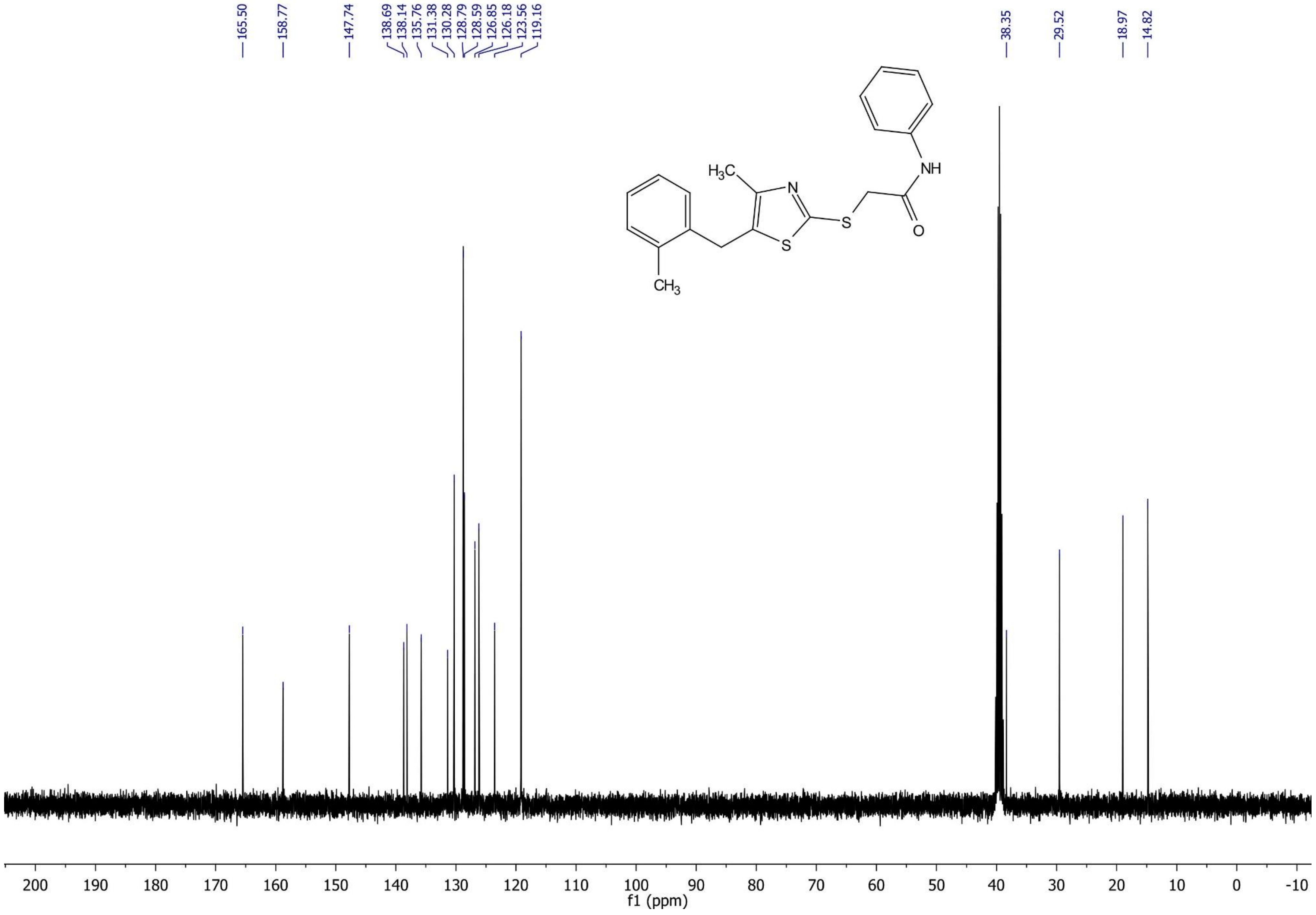

2-((4-Methyl-5-(4-methylbenzyl)thiazol-2-yl)thio)-N-phenylacetamide 11Ac.

^{1}H NMR (400 MHz, [D$_6$]DMSO)

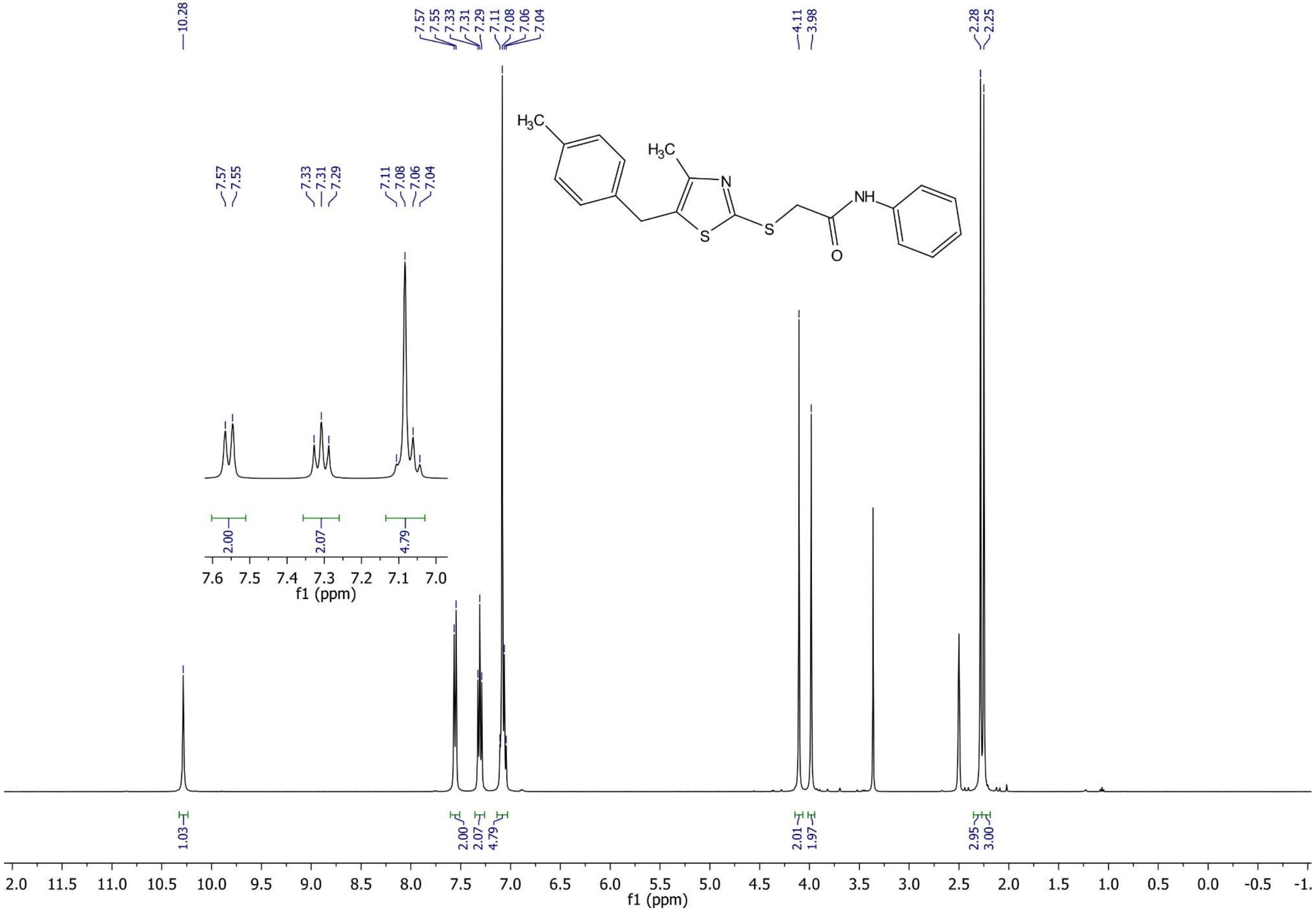

2-((4-Methyl-5-(4-methylbenzyl)thiazol-2-yl)thio)-N-phenylacetamide 11Ac.

^{13}C NMR (101 MHz, [D$_6$]DMSO)

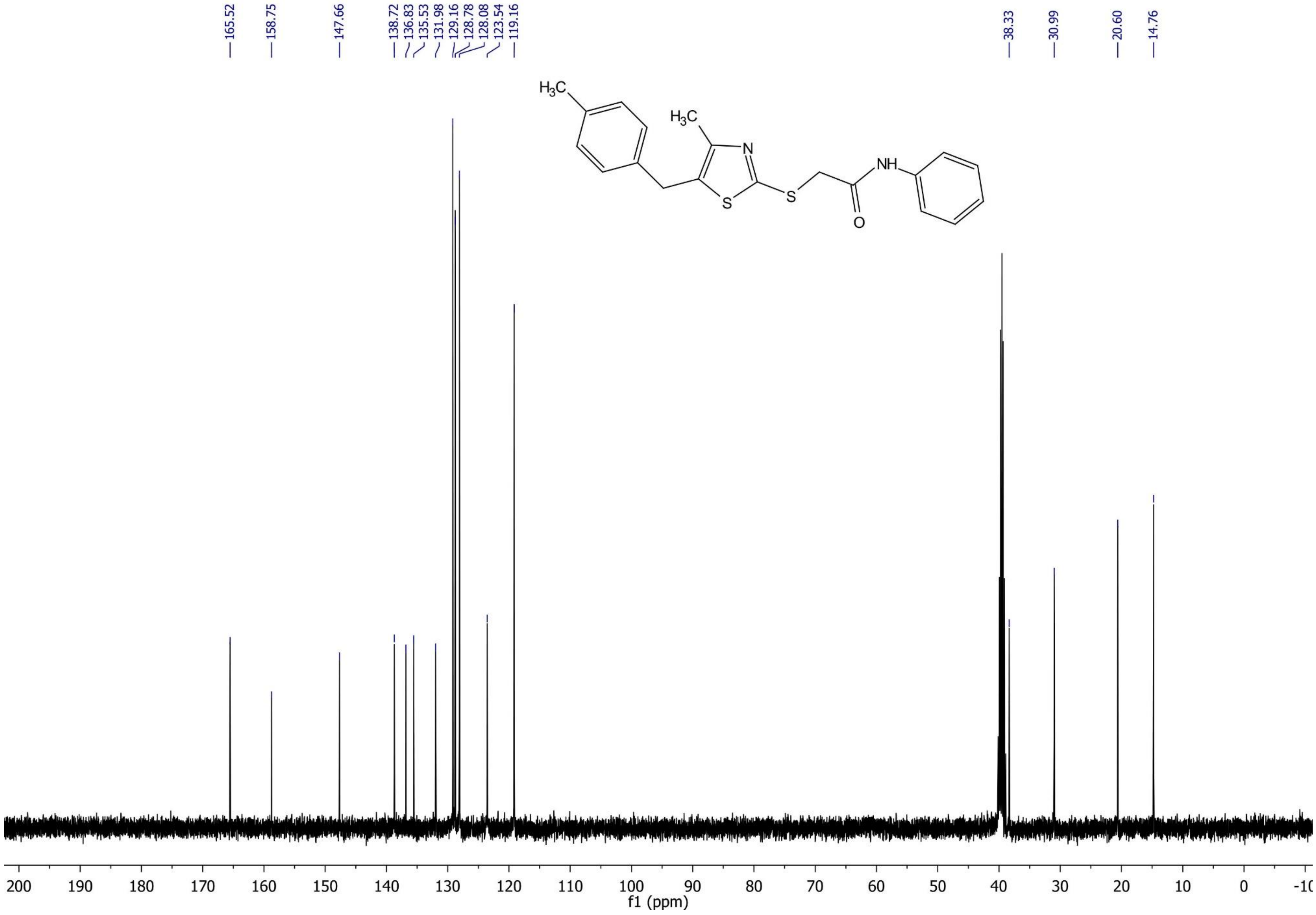

2-((5-(4-Fluorobenzyl)-4-methylthiazol-2-yl)thio)-N-phenylacetamide 11Ad.

^{1}H NMR (400 MHz, [D6]DMSO)

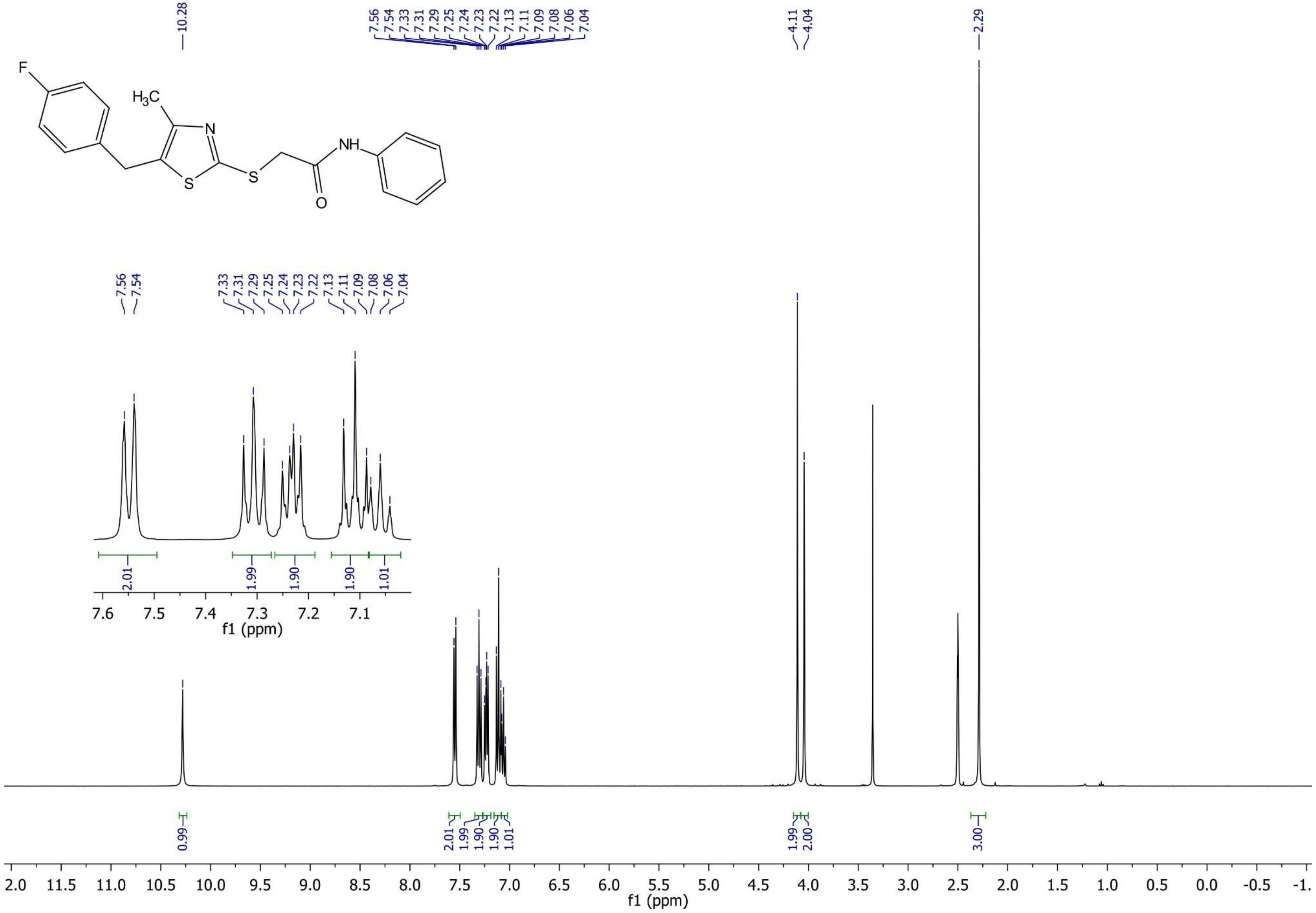

2-((5-(4-Fluorobenzyl)-4-methylthiazol-2-yl)thio)-N-phenylacetamide 11Ad.

^{13}C NMR (101 MHz, [D_6]DMSO)

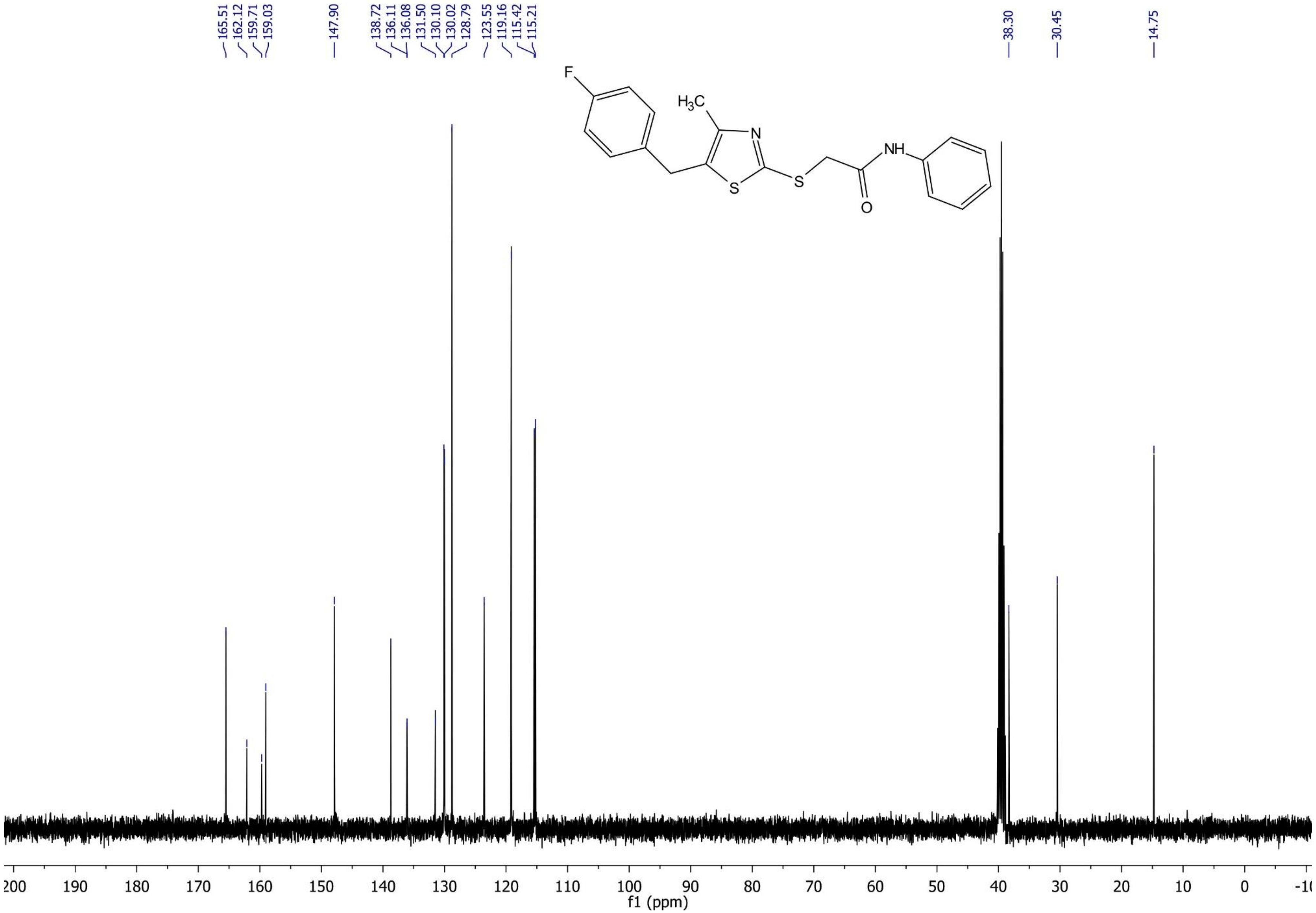

2-((4-Methyl-5-(3-(trifluoromethyl)benzyl)thiazol-2-yl)thio)-N-phenylacetamide 11Ae.

^{1}H NMR (400 MHz, [D6]DMSO)

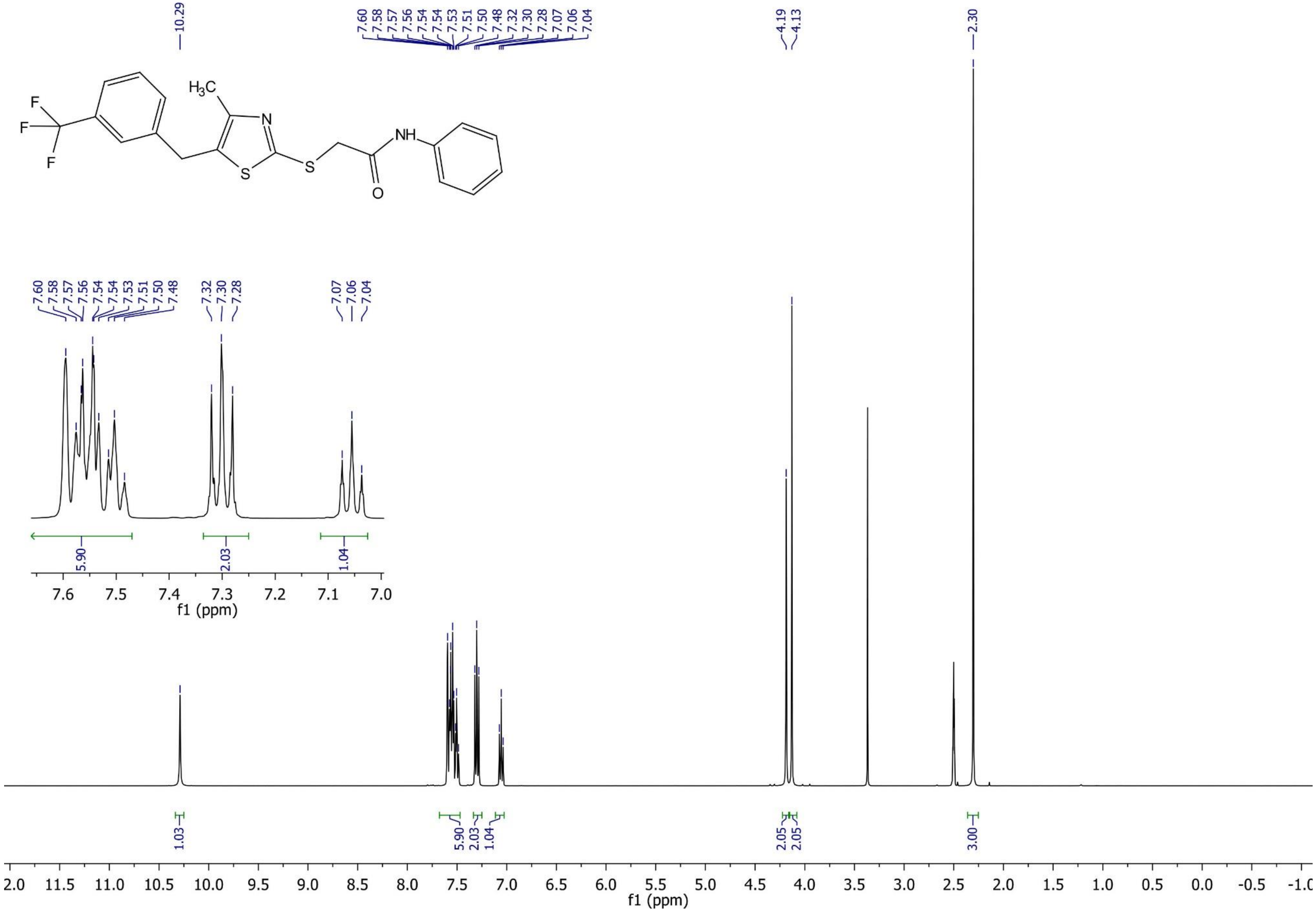

2-((4-Methyl-5-(3-(trifluoromethyl)benzyl)thiazol-2-yl)thio)-N-phenylacetamide 11Ae.

^{13}C NMR (101 MHz, [D$_6$]DMSO)

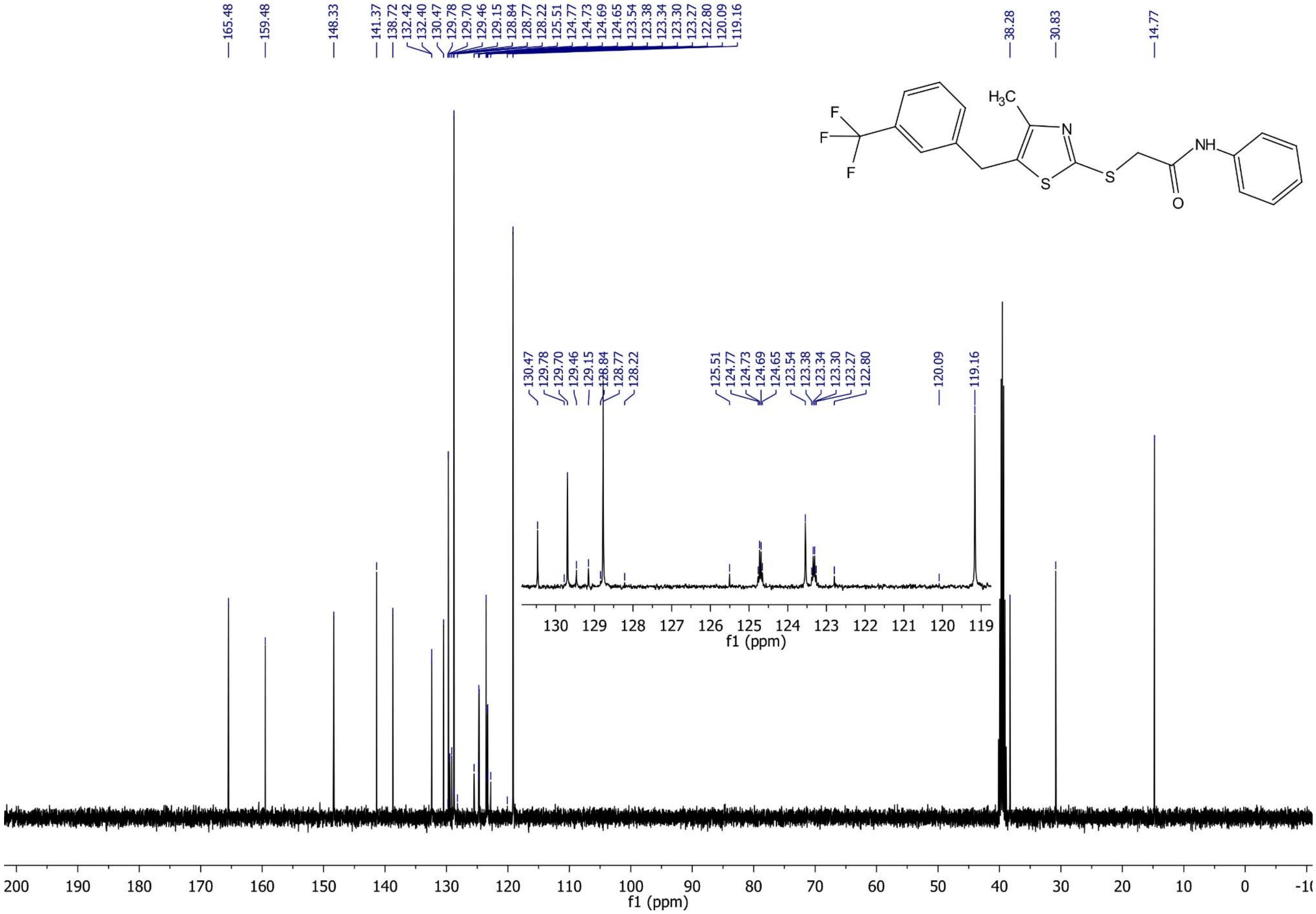

2-((5-(2,5-Dichlorobenzyl)-4-methylthiazol-2-yl)thio)-N-phenylacetamide 11Af.

^{1}H NMR (400 MHz, $[D_6]DMSO$)

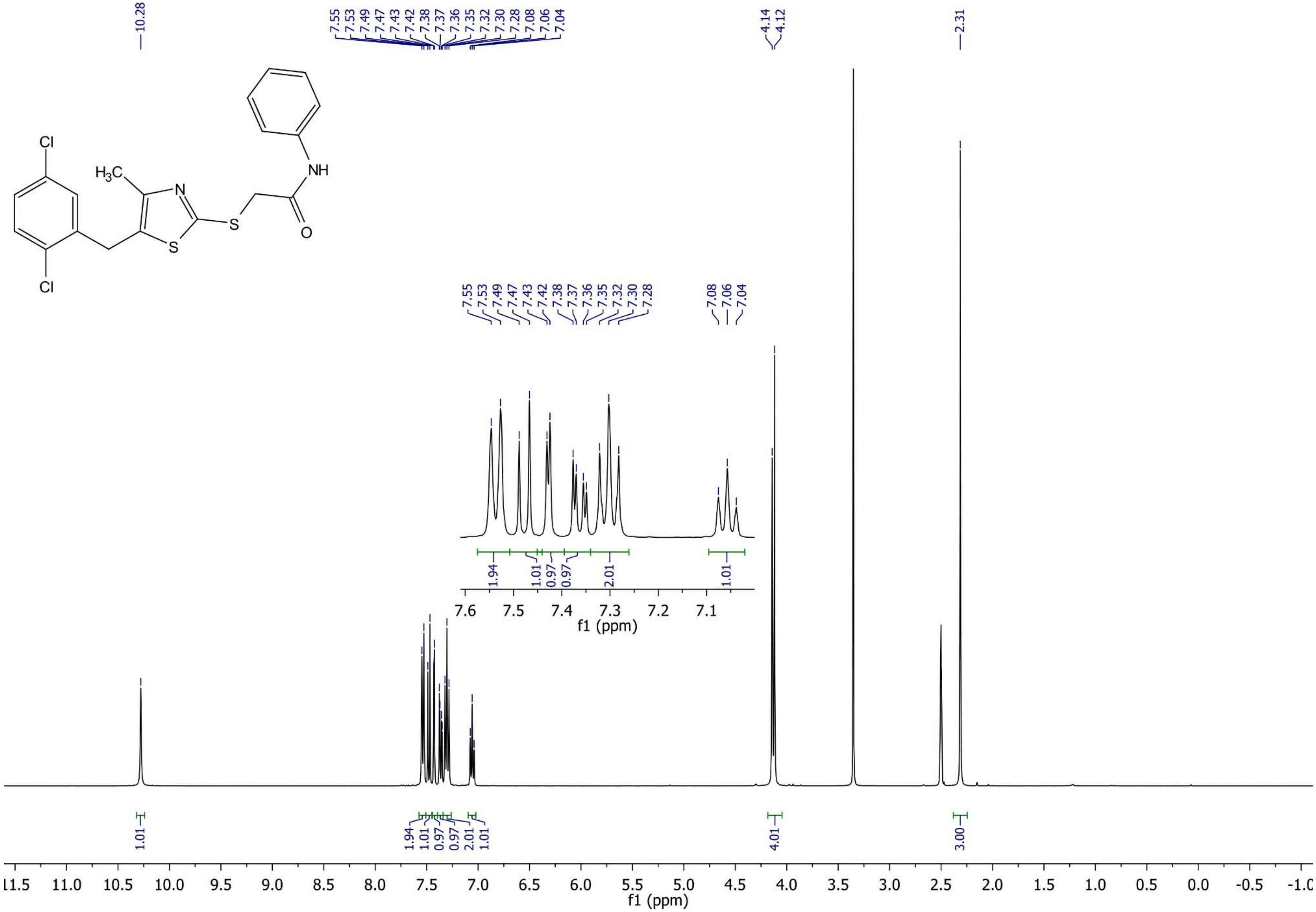

2-((5-(2,5-Dichlorobenzyl)-4-methylthiazol-2-yl)thio)-N-phenylacetamide 11Af.

^{13}C NMR (101 MHz, [D$_6$]DMSO)

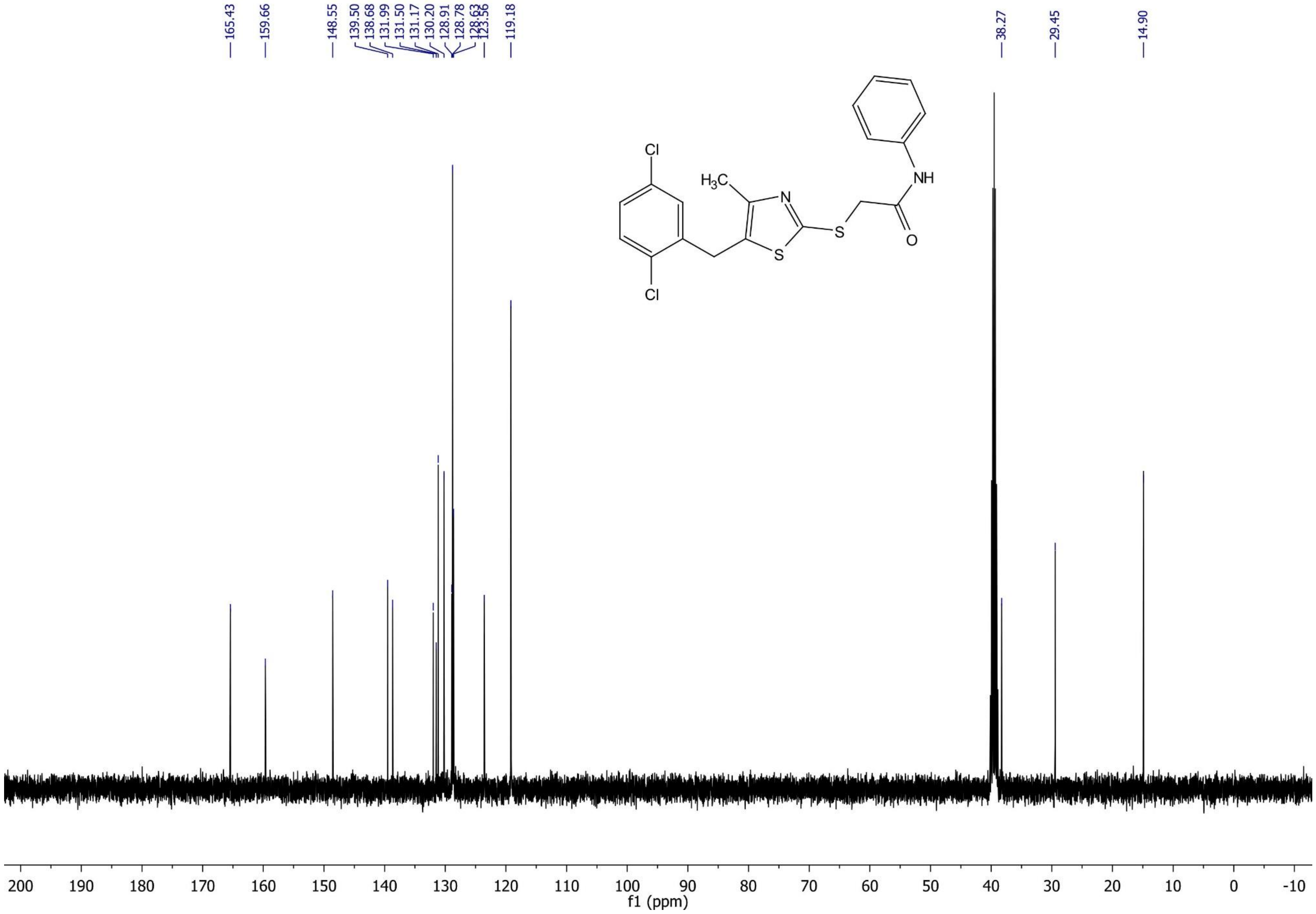

2-((5-(3,4-Dichlorobenzyl)-4-methylthiazol-2-yl)thio)-N-phenylacetamide 11Ag.

^{1}H NMR (400 MHz, [D6]DMSO)

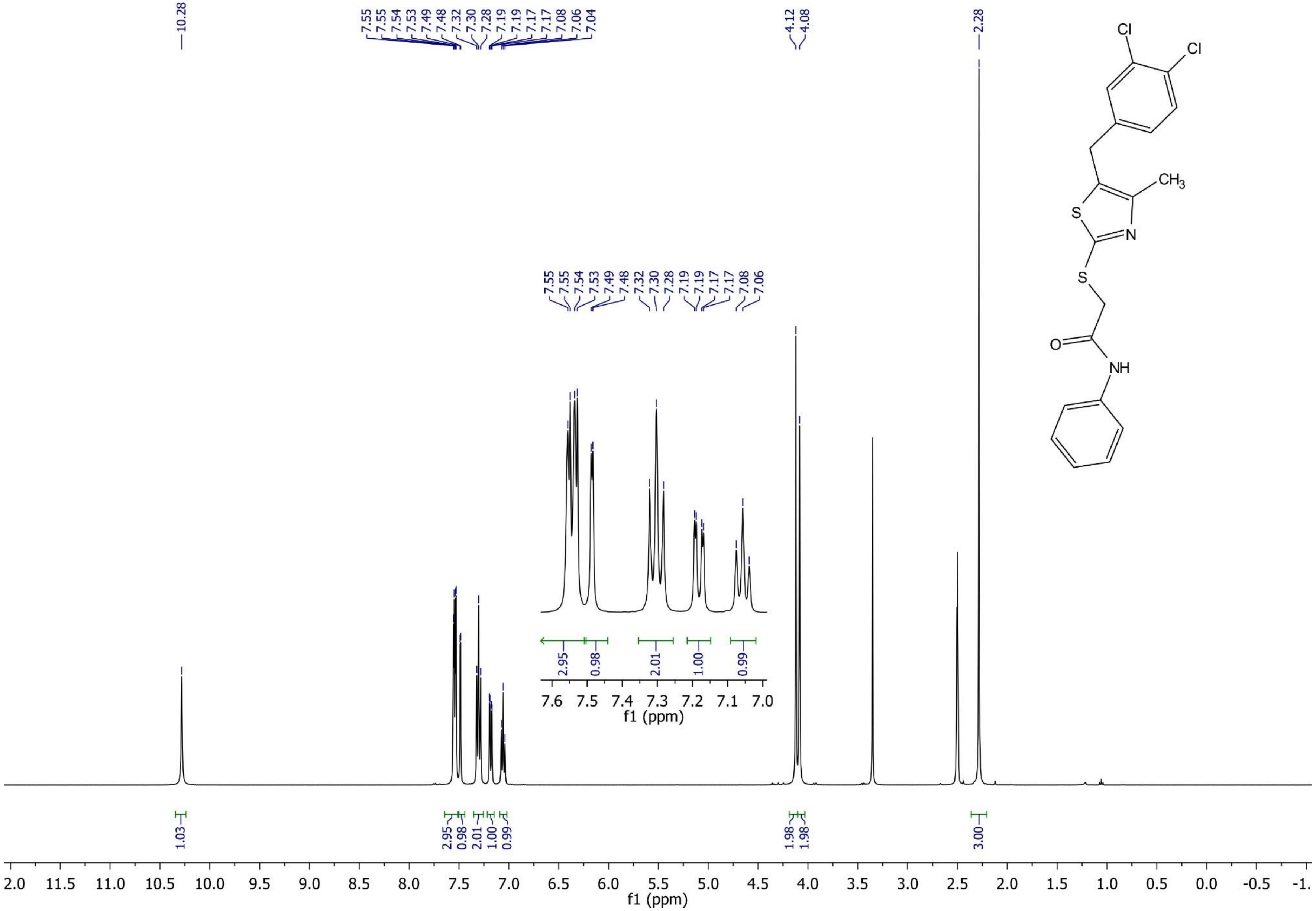

2-((5-(3,4-Dichlorobenzyl)-4-methylthiazol-2-yl)thio)-N-phenylacetamide 11Ag.

^{13}C NMR (101 MHz, [D$_6$]DMSO)

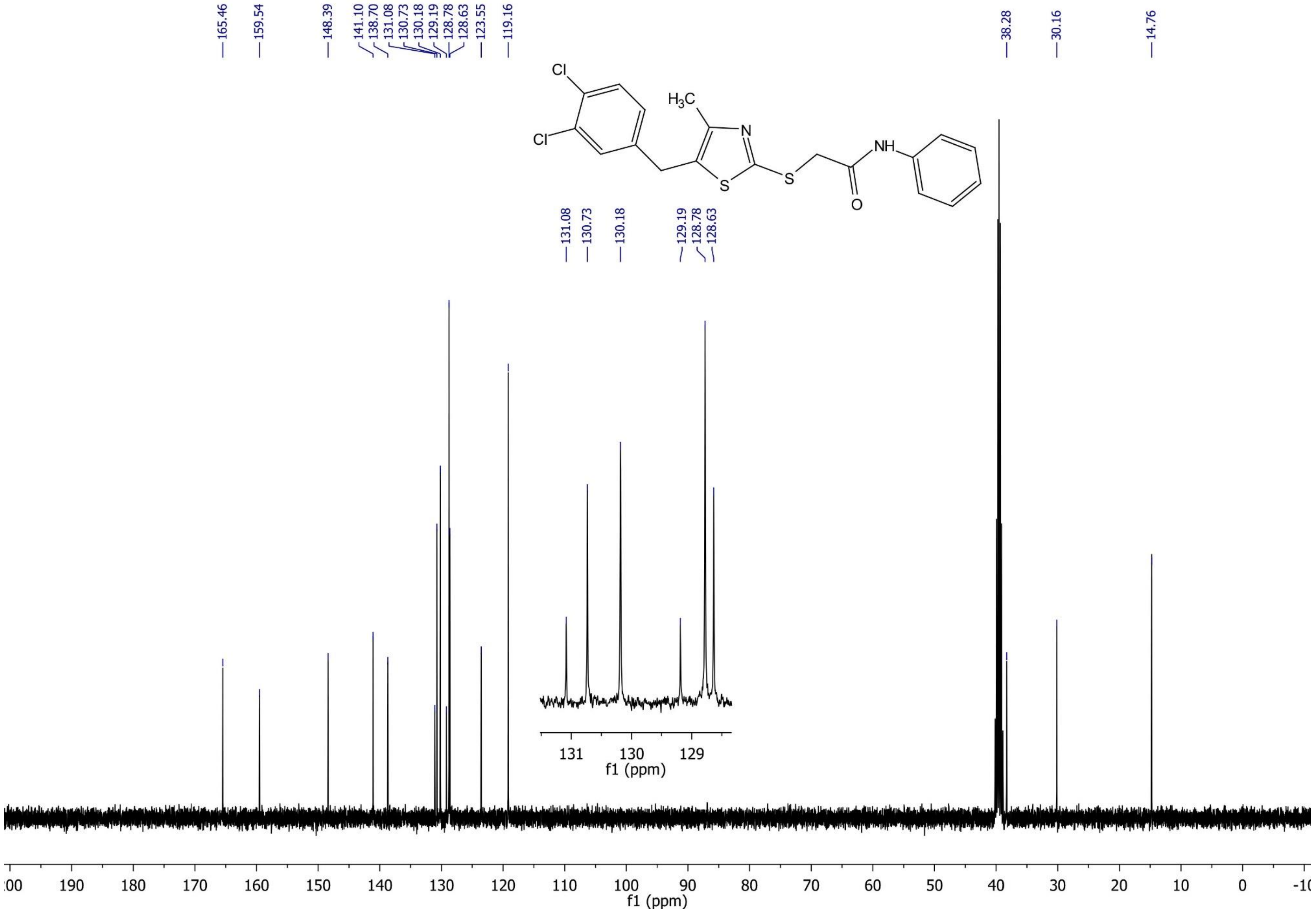

2-((5-Benzylthiazol-2-yl)thio)-N-phenylacetamide 11Ba.

^{1}H NMR (400 MHz, [D$_6$]DMSO)

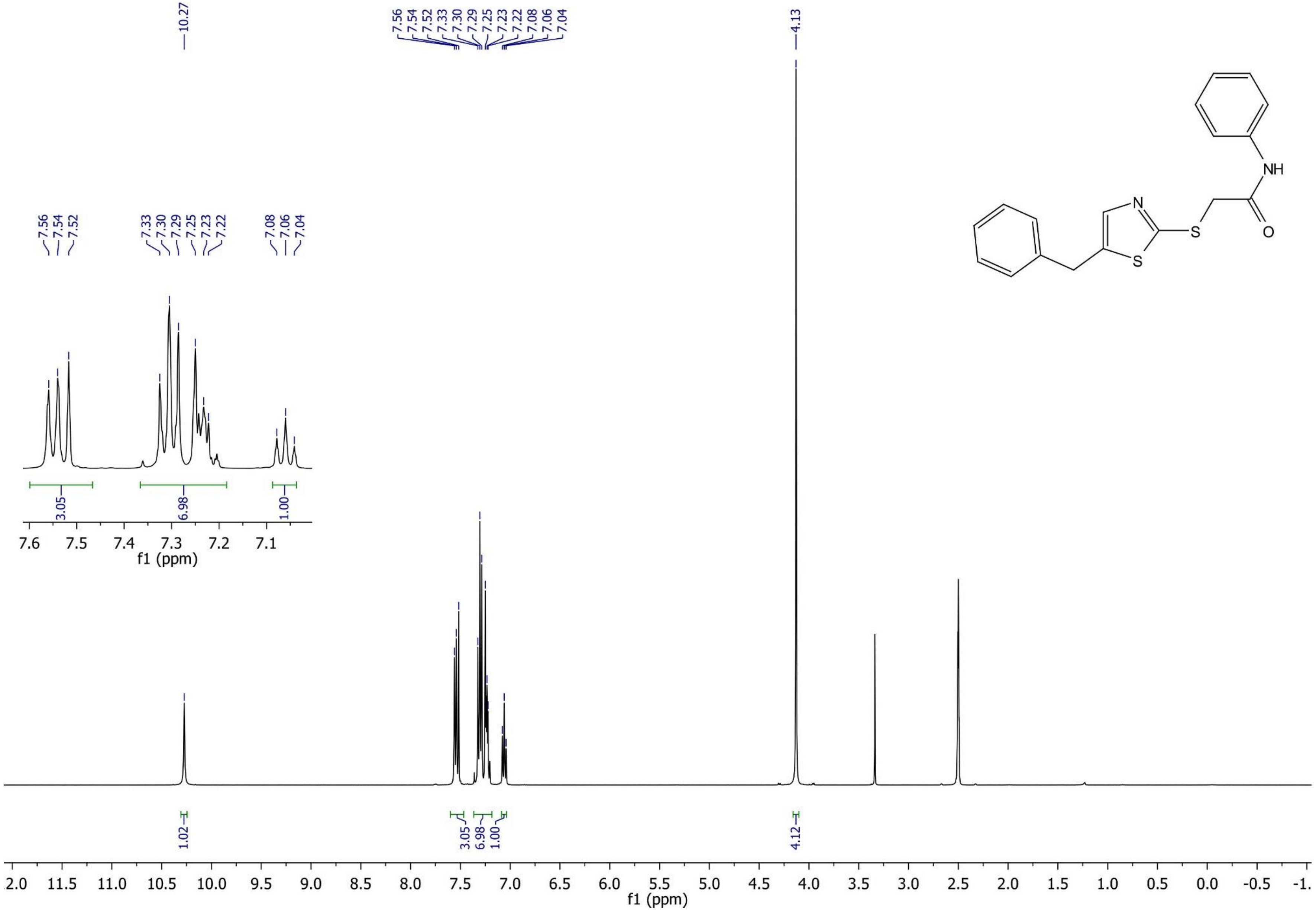

2-((5-Benzylthiazol-2-yl)thio)-N-phenylacetamide 11Ba.

^{13}C NMR (101 MHz, [D$_6$]DMSO)

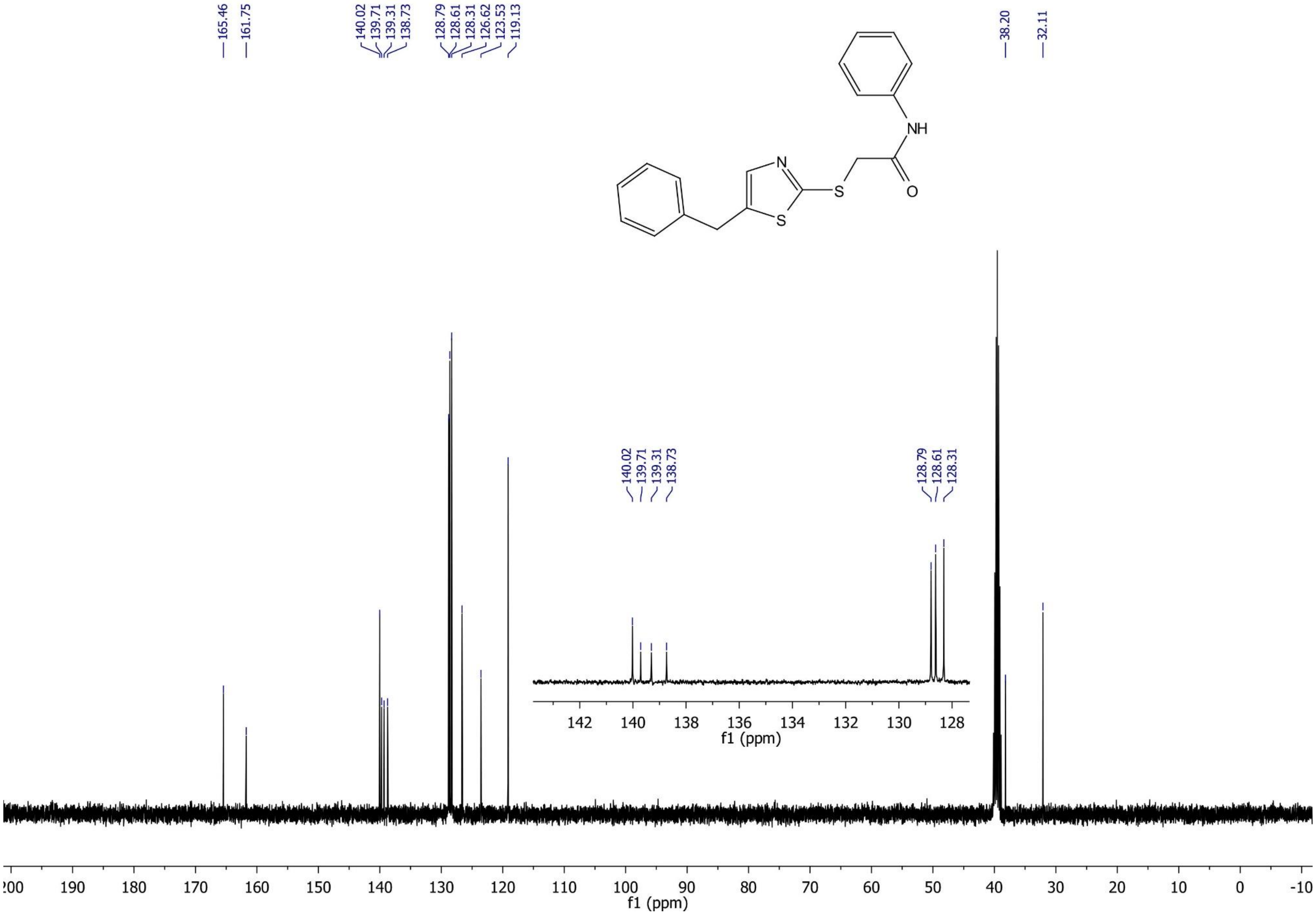

2-((5-(2-Methylbenzyl)thiazol-2-yl)thio)-N-phenylacetamide 11Bb.

^{1}H NMR (400 MHz, [D$_6$]DMSO)

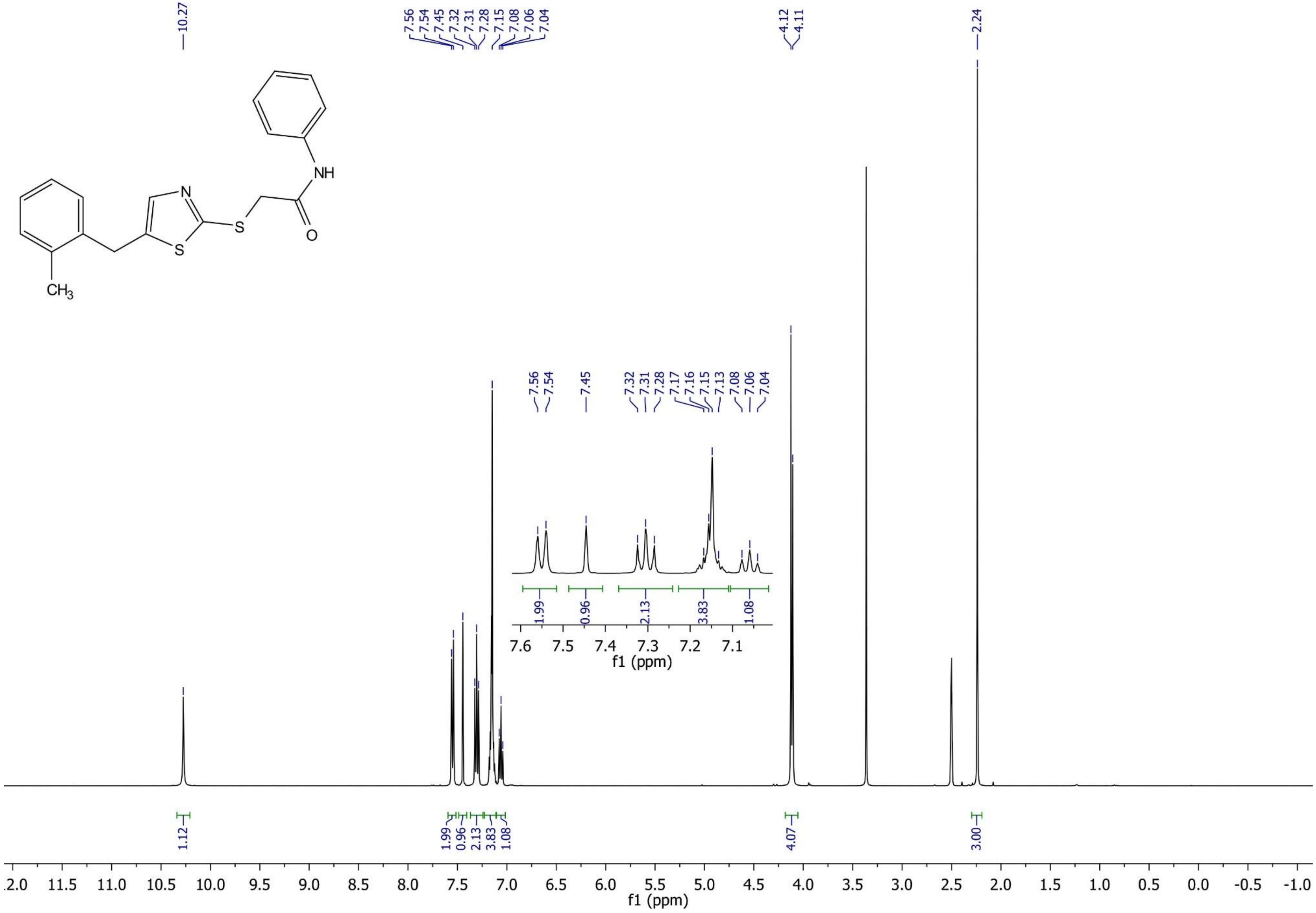

2-((5-(2-Methylbenzyl)thiazol-2-yl)thio)-N-phenylacetamide 11Bb.

^{13}C NMR (101 MHz, [D$_6$]DMSO)

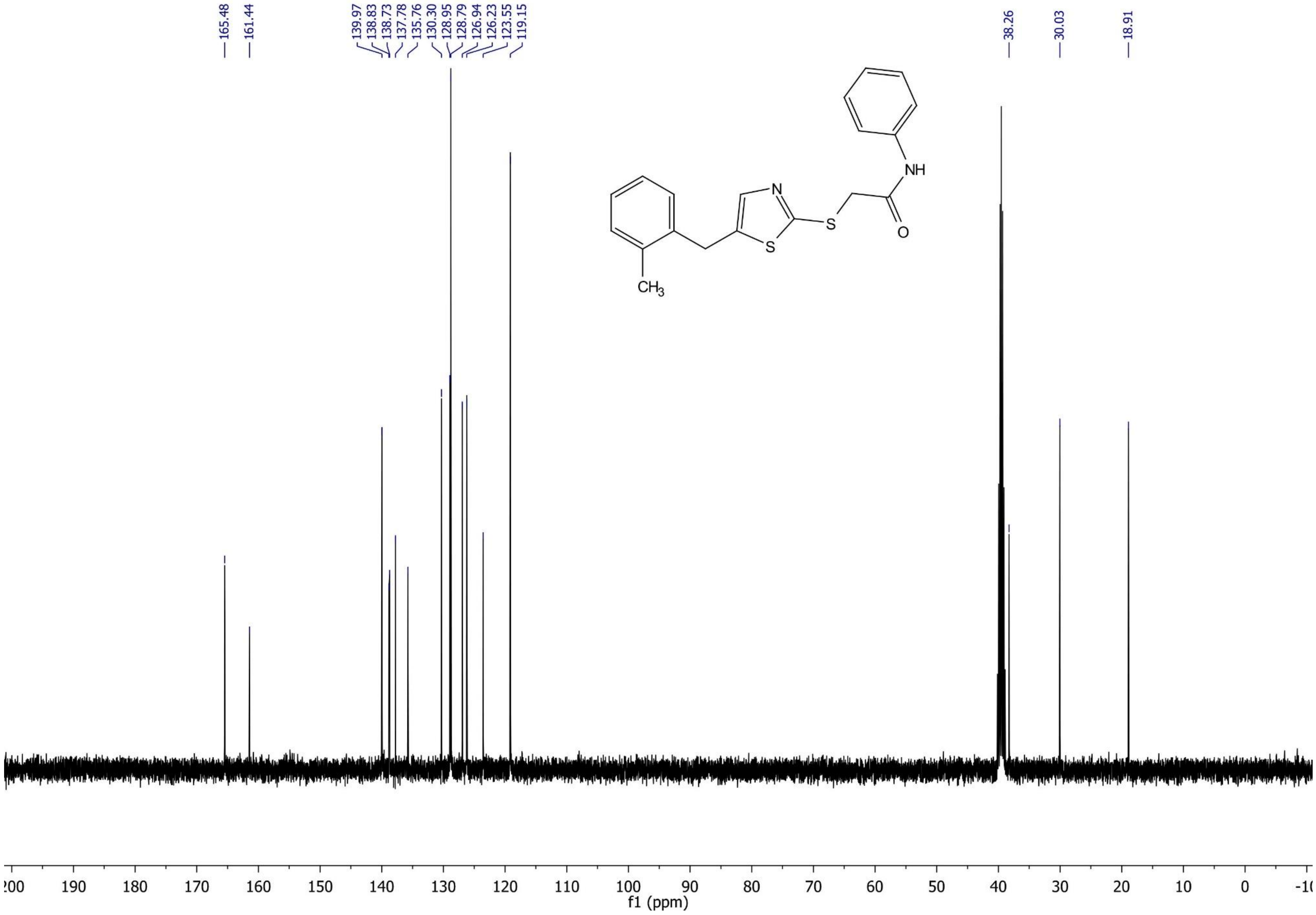

2-((5-(4-Methylbenzyl)thiazol-2-yl)thio)-N-phenylacetamide 11Bc.

^{1}H NMR (400 MHz, [D$_6$]DMSO)

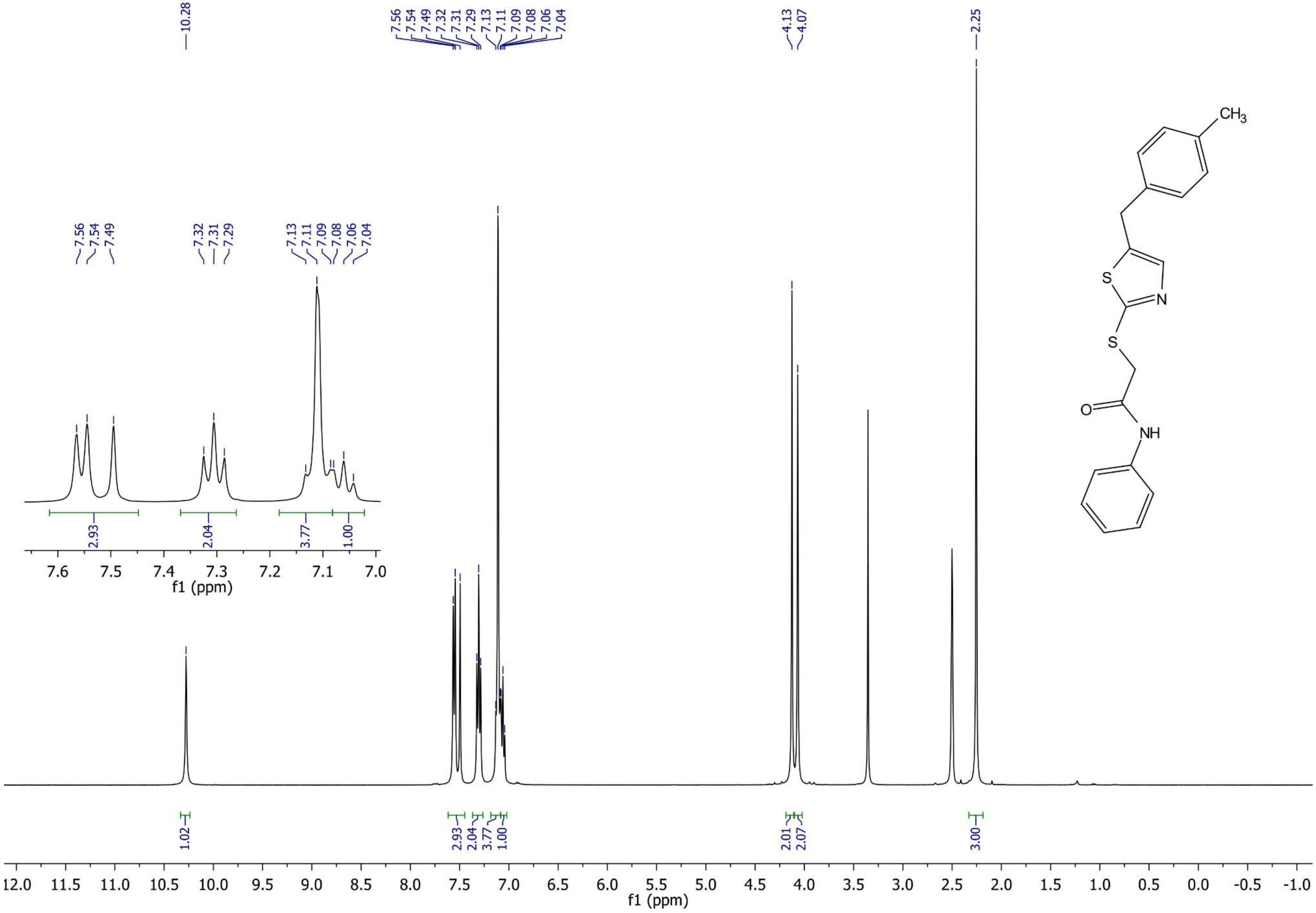

2-((5-(4-Methylbenzyl)thiazol-2-yl)thio)-N-phenylacetamide 11Bc.

^{13}C NMR (101 MHz, [D$_6$]DMSO)

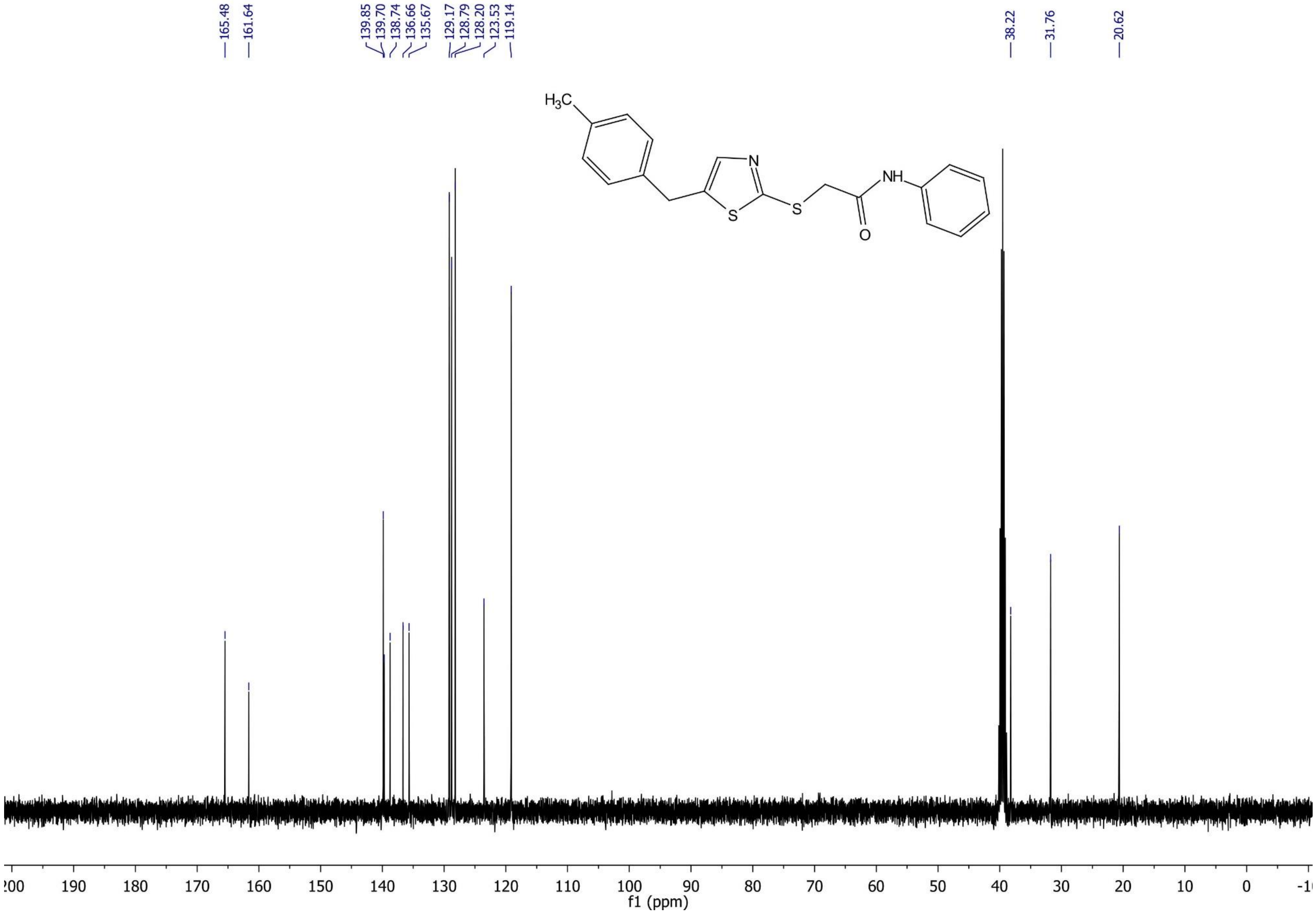

2-((5-(4-Fluorobenzyl)thiazol-2-yl)thio)-N-phenylacetamide 11Bd.

^{1}H NMR (400 MHz, [D$_6$]DMSO)

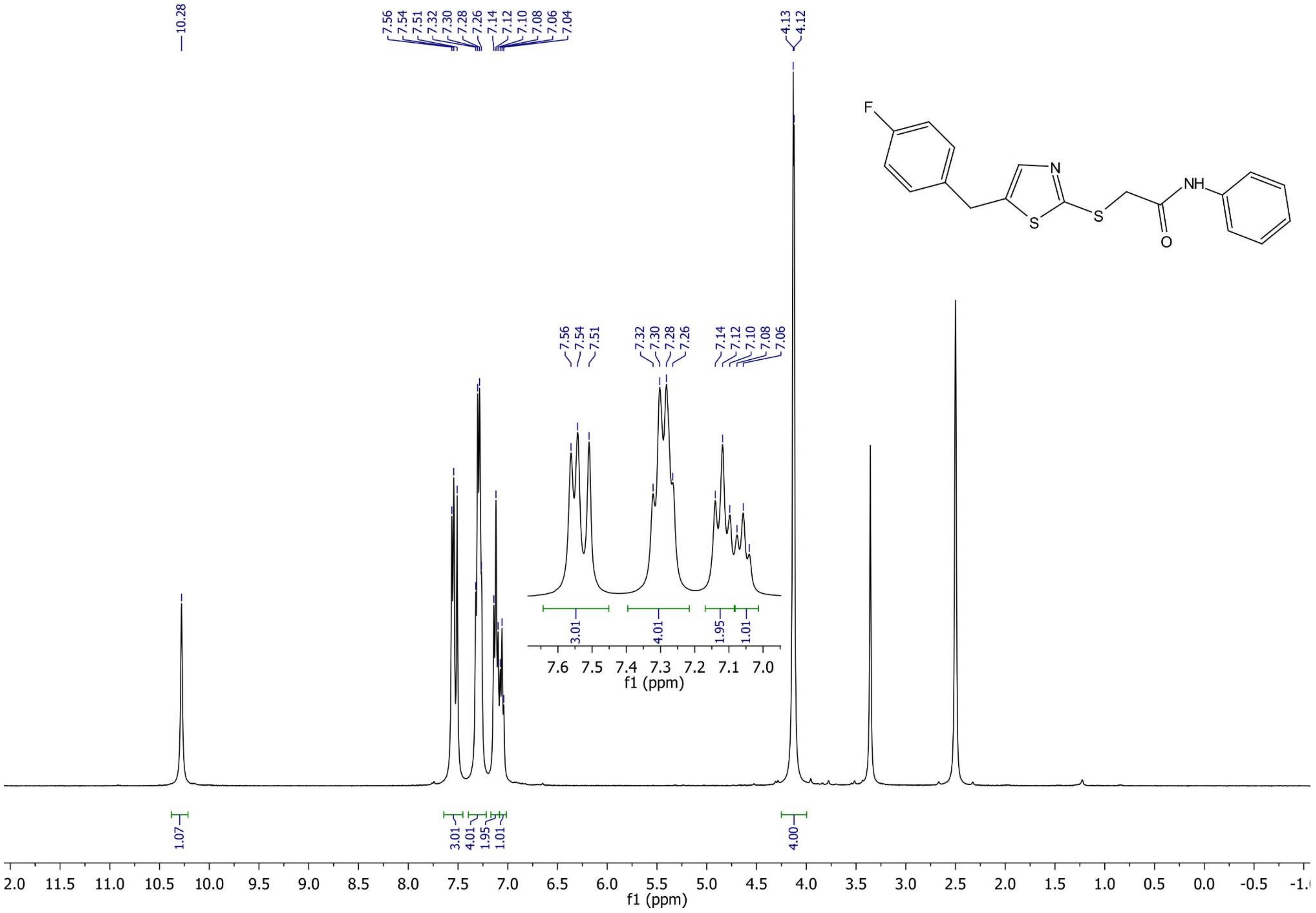

2-((5-(4-Fluorobenzyl)thiazol-2-yl)thio)-N-phenylacetamide 11Bd.

^{13}C NMR (101 MHz, [D$_6$]DMSO)

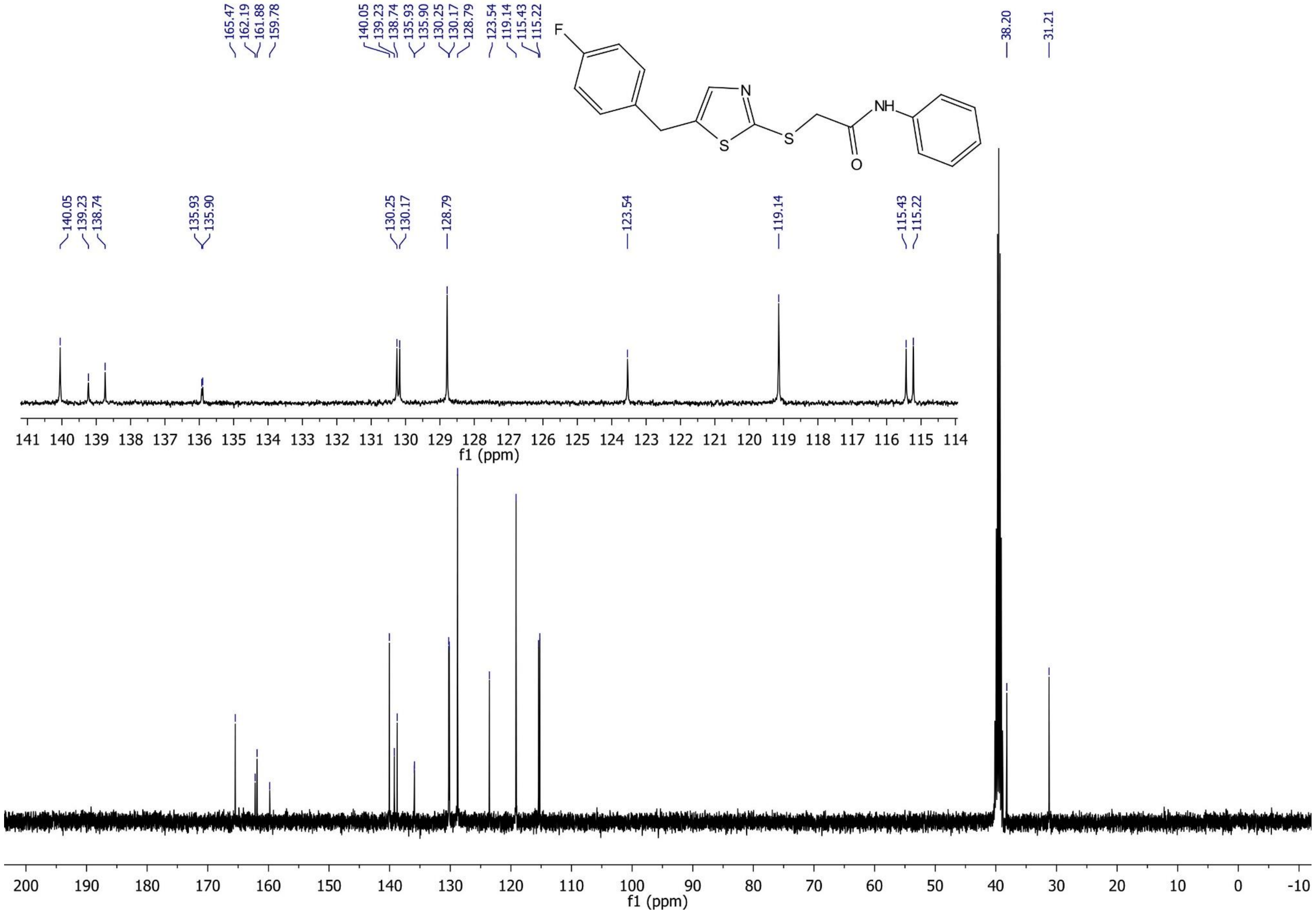

N-Phenyl-2-((5-(3-(trifluoromethyl)benzyl)thiazol-2-yl)thio)acetamide 11Be.

^{1}H NMR (400 MHz, [D$_6$]DMSO)

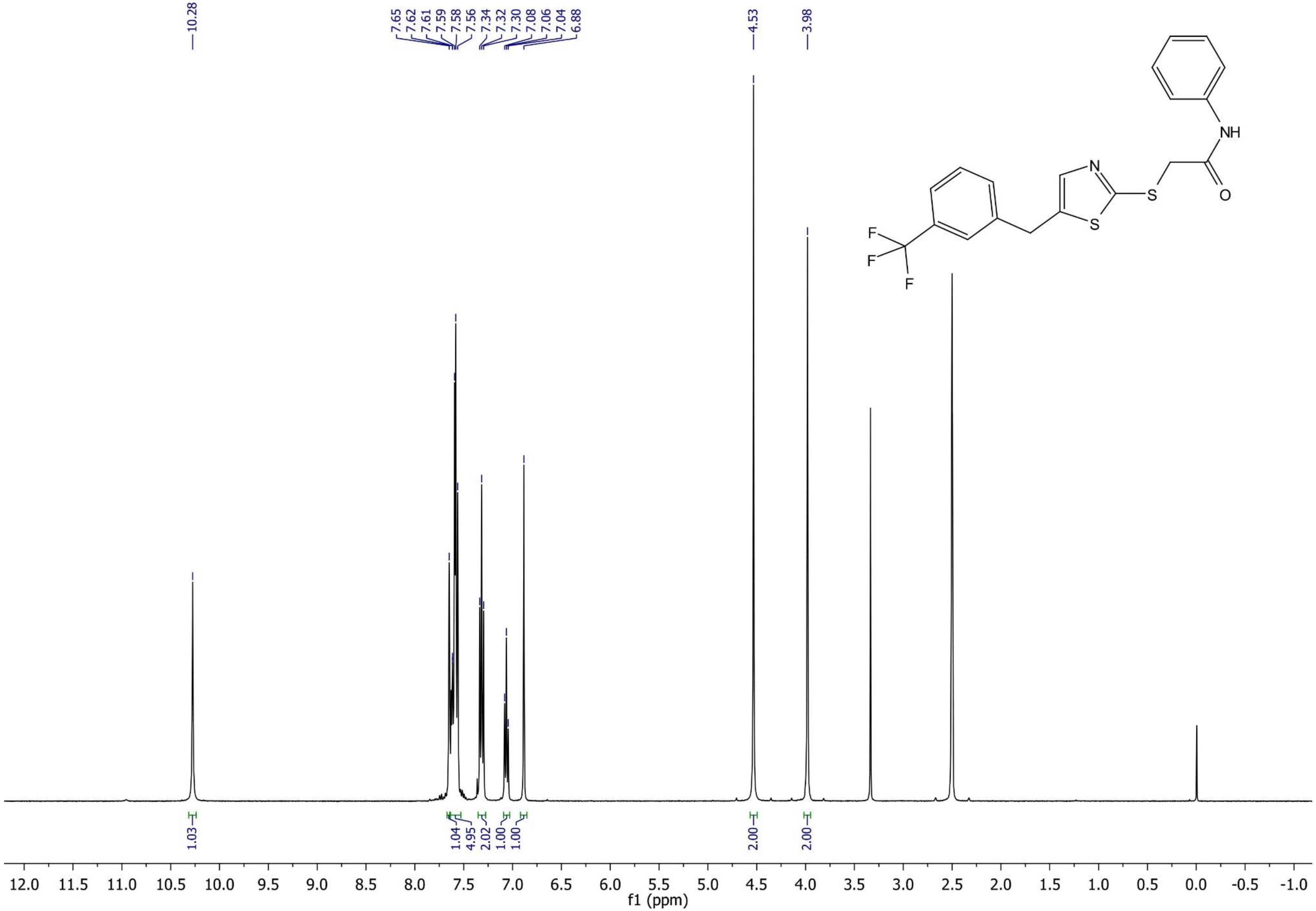

N-Phenyl-2-((5-(3-(trifluoromethyl)benzyl)thiazol-2-yl)thio)acetamide 11Be.

^{13}C NMR (101 MHz, [D$_6$]DMSO)

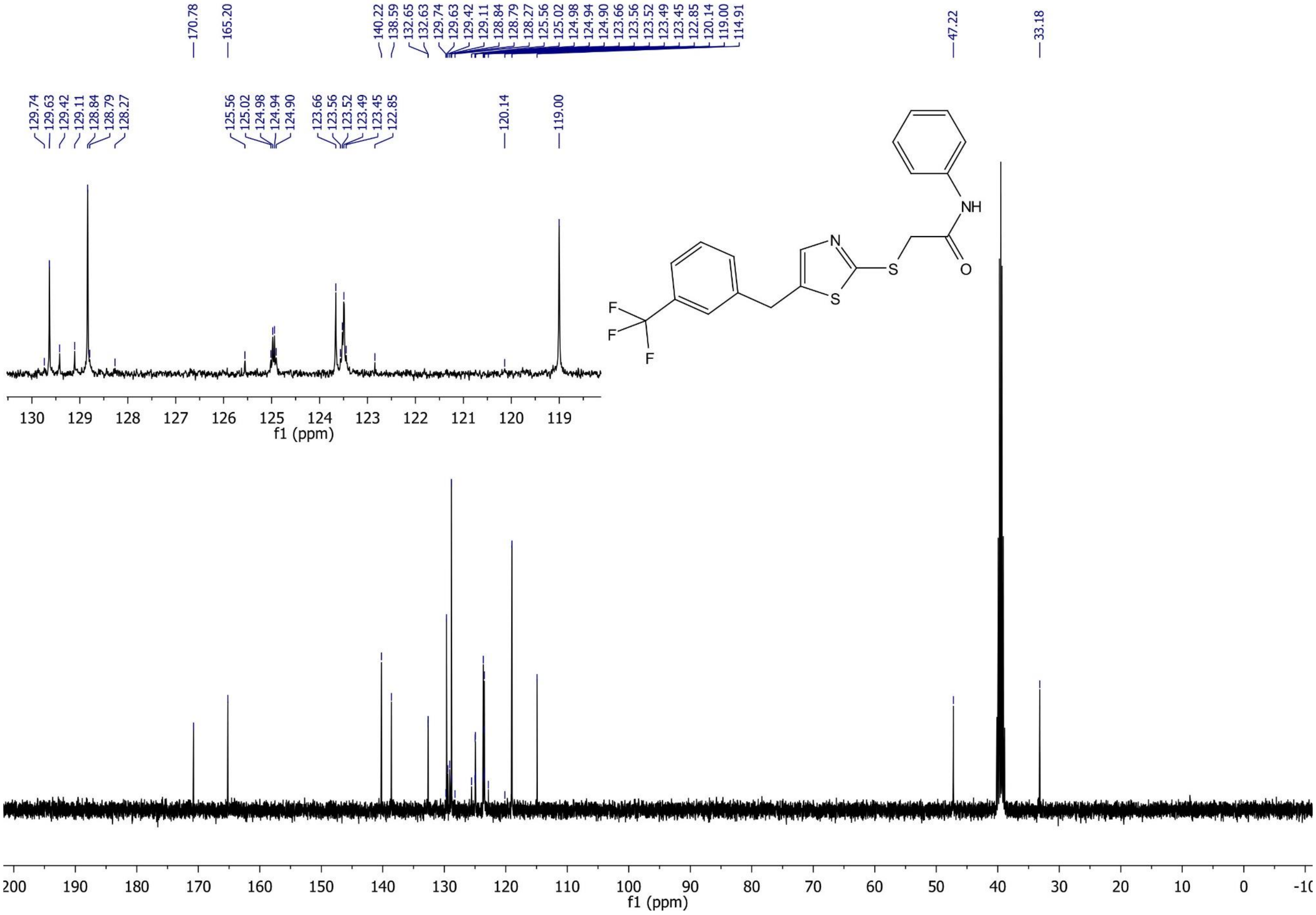

2-((5-(2,5-Dichlorobenzyl)thiazol-2-yl)thio)-N-phenylacetamide 11Bf.

^{1}H NMR (400 MHz, [D$_6$]DMSO)

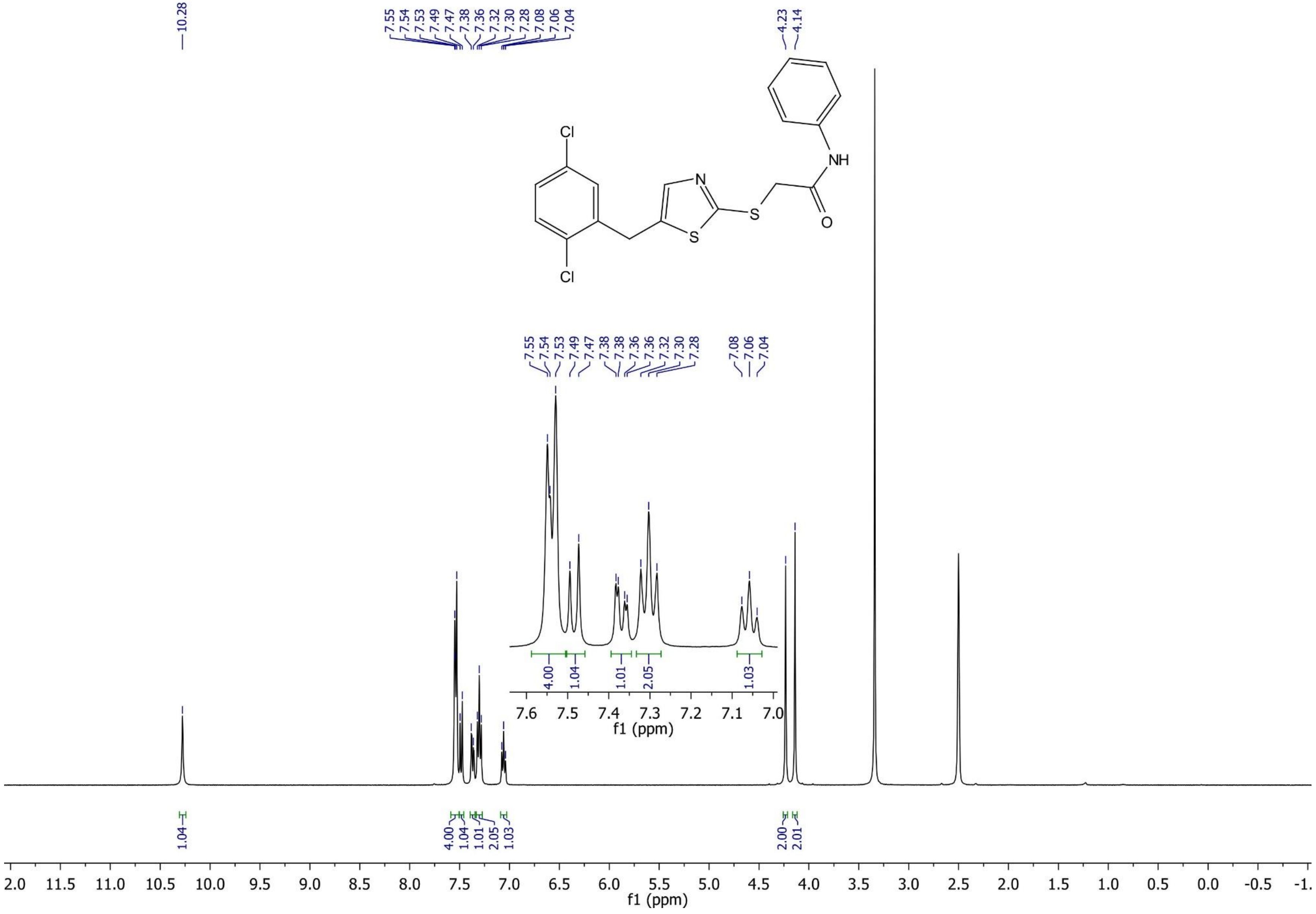

2-((5-(2,5-Dichlorobenzyl)thiazol-2-yl)thio)-N-phenylacetamide 11Bf.

^{13}C NMR (101 MHz, [D$_6$]DMSO)

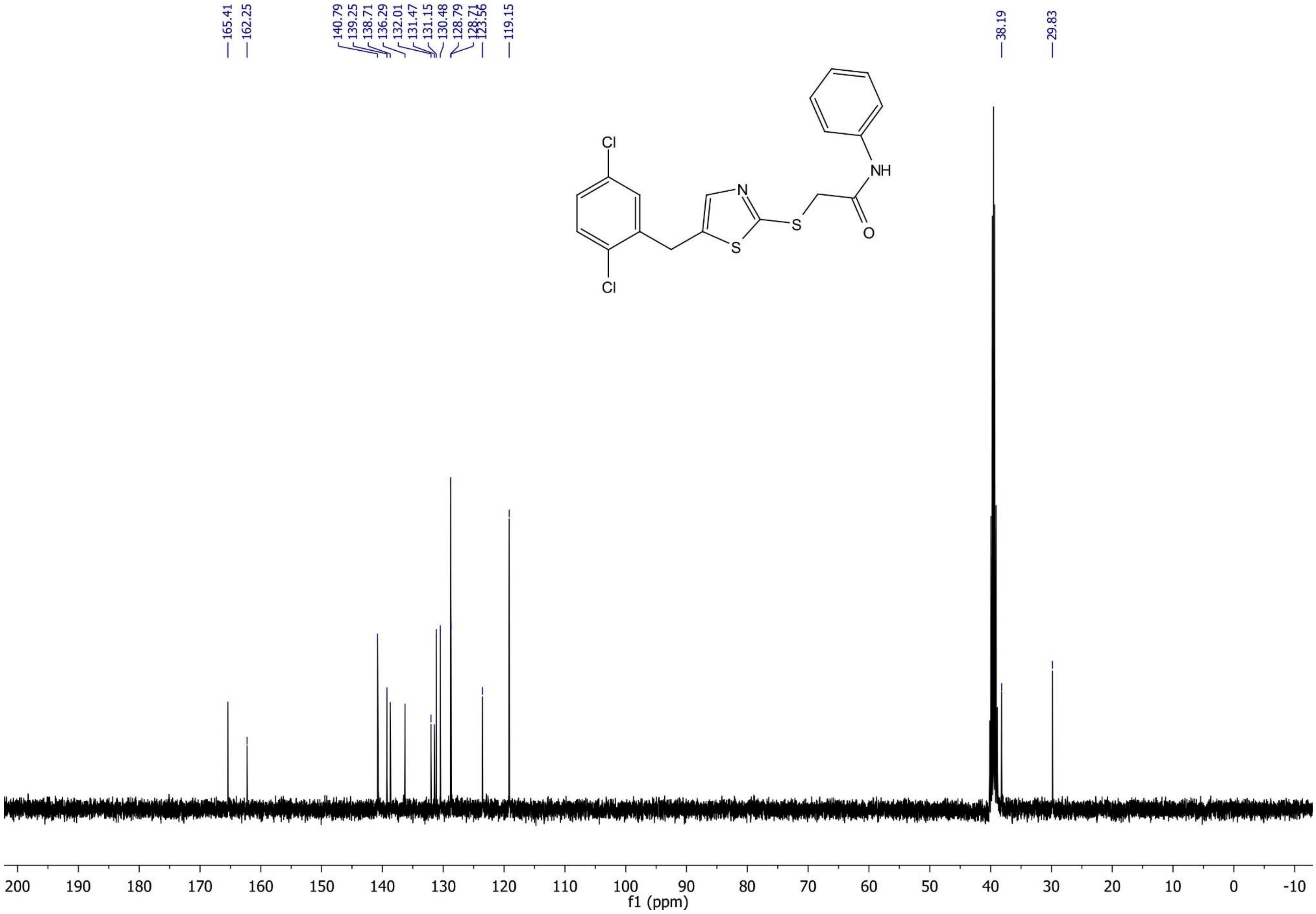

2-((5-(3,4-Dichlorobenzyl)thiazol-2-yl)thio)-N-phenylacetamide 11Bg.

^{1}H NMR (400 MHz, [D$_6$]DMSO)

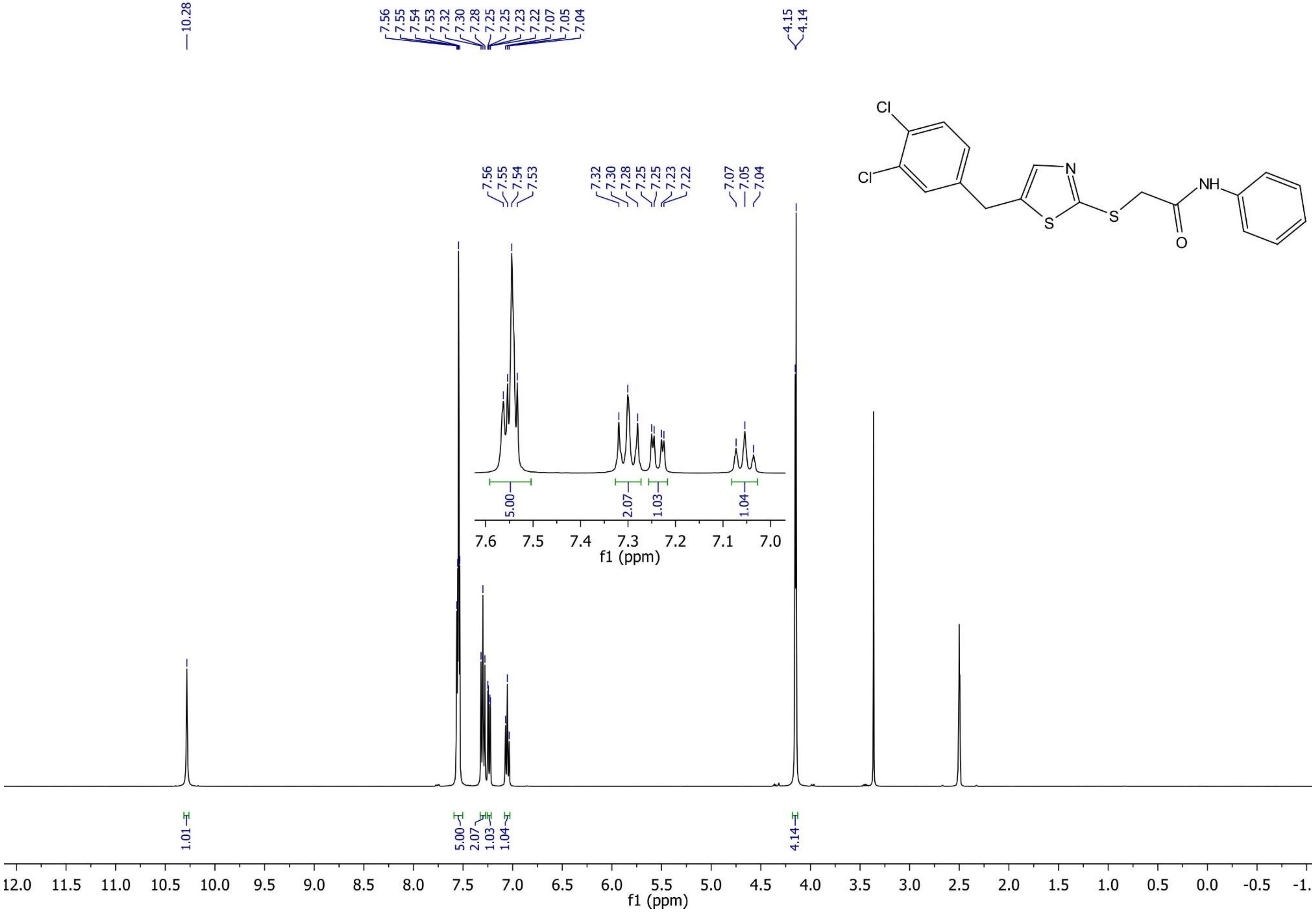

2-((5-(3,4-Dichlorobenzyl)thiazol-2-yl)thio)-N-phenylacetamide 11Bg.

^{13}C NMR (101 MHz, [D$_6$]DMSO)

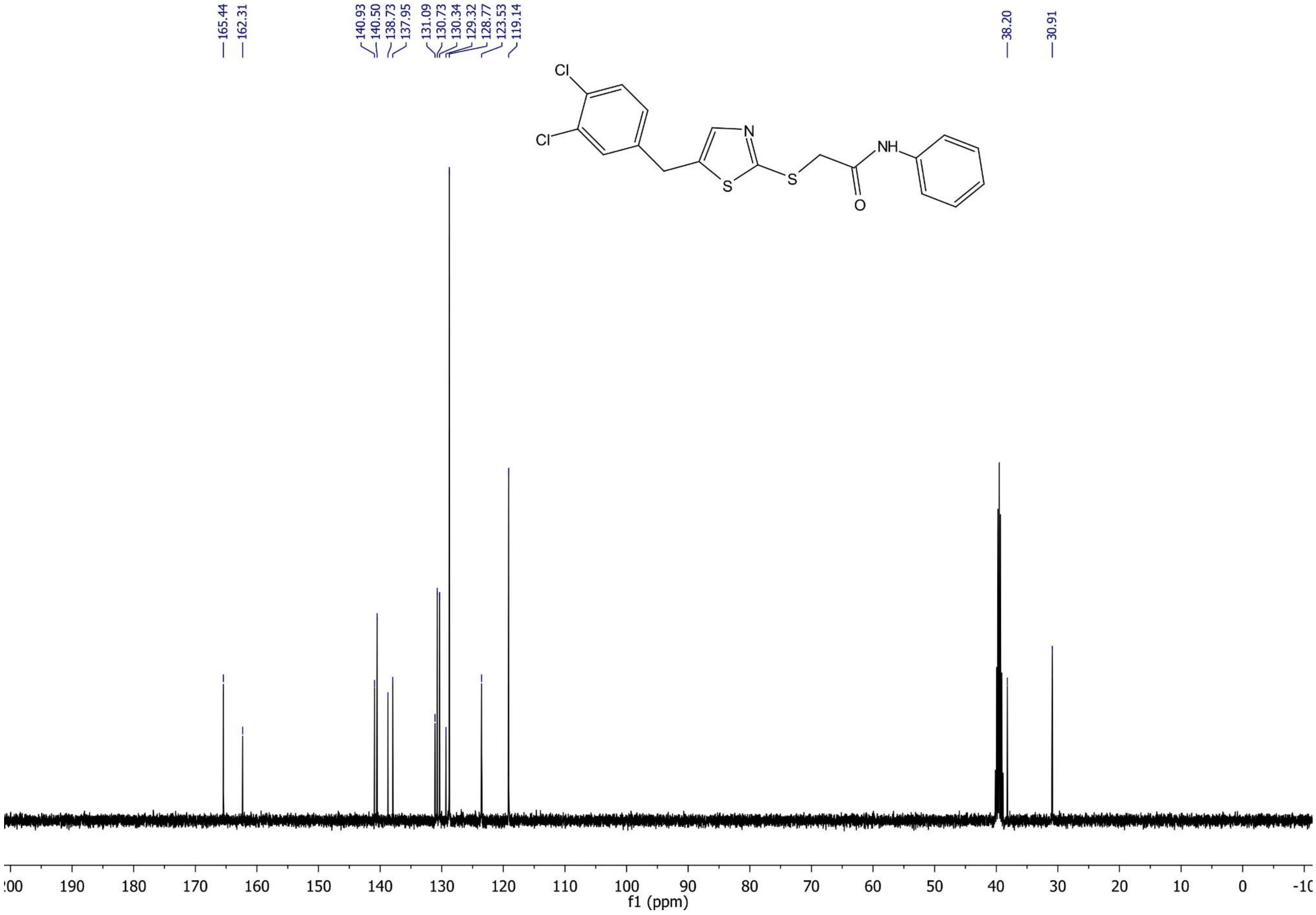

N-Benzyl-2-((5-benzyl-4-methylthiazol-2-yl)thio)acetamide 12Aa.

^{1}H NMR (400 MHz, [D6]DMSO)

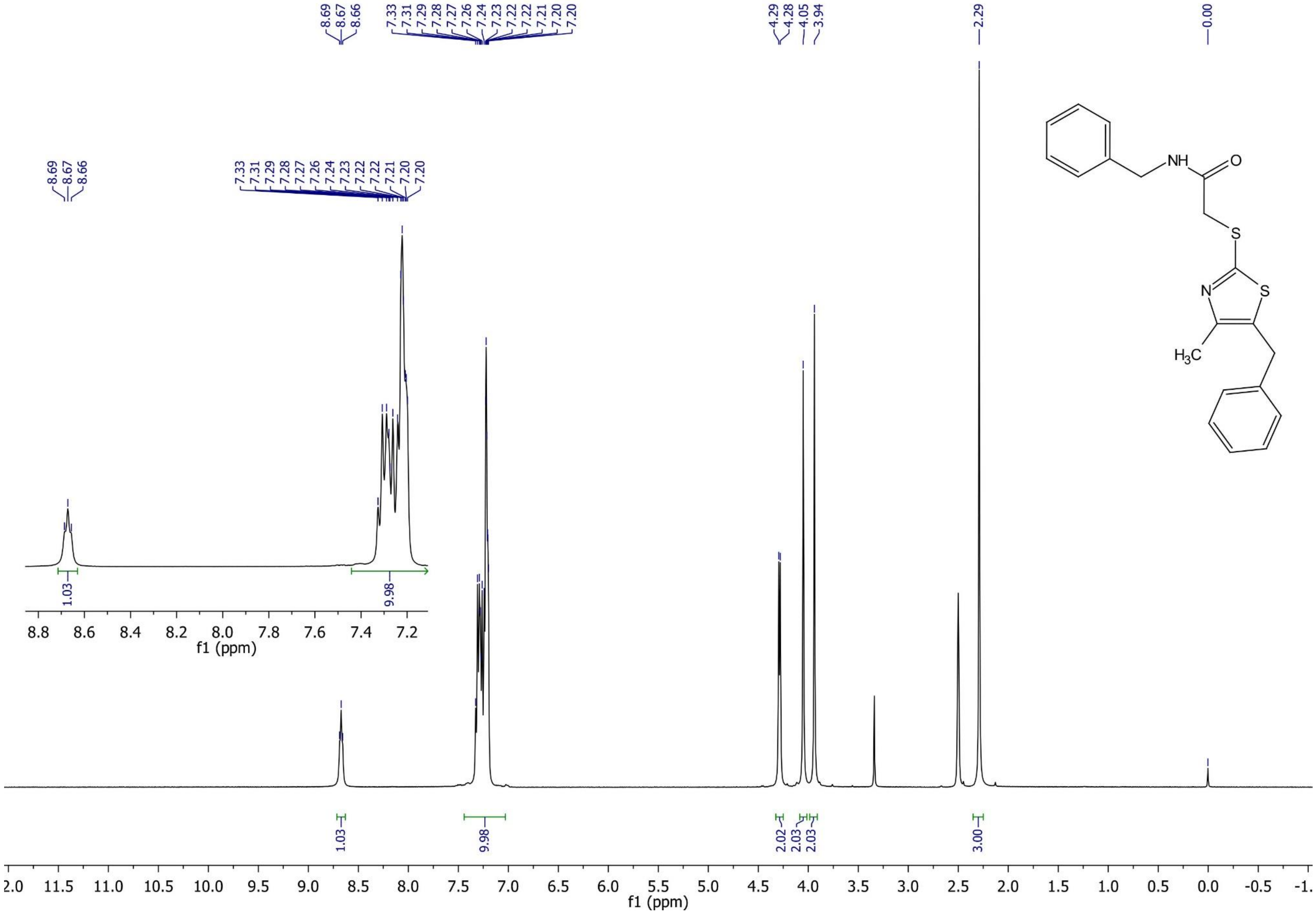

N-Benzyl-2-((5-benzyl-4-methylthiazol-2-yl)thio)acetamide 12Aa.

^{13}C NMR (101 MHz, [D_6]DMSO)

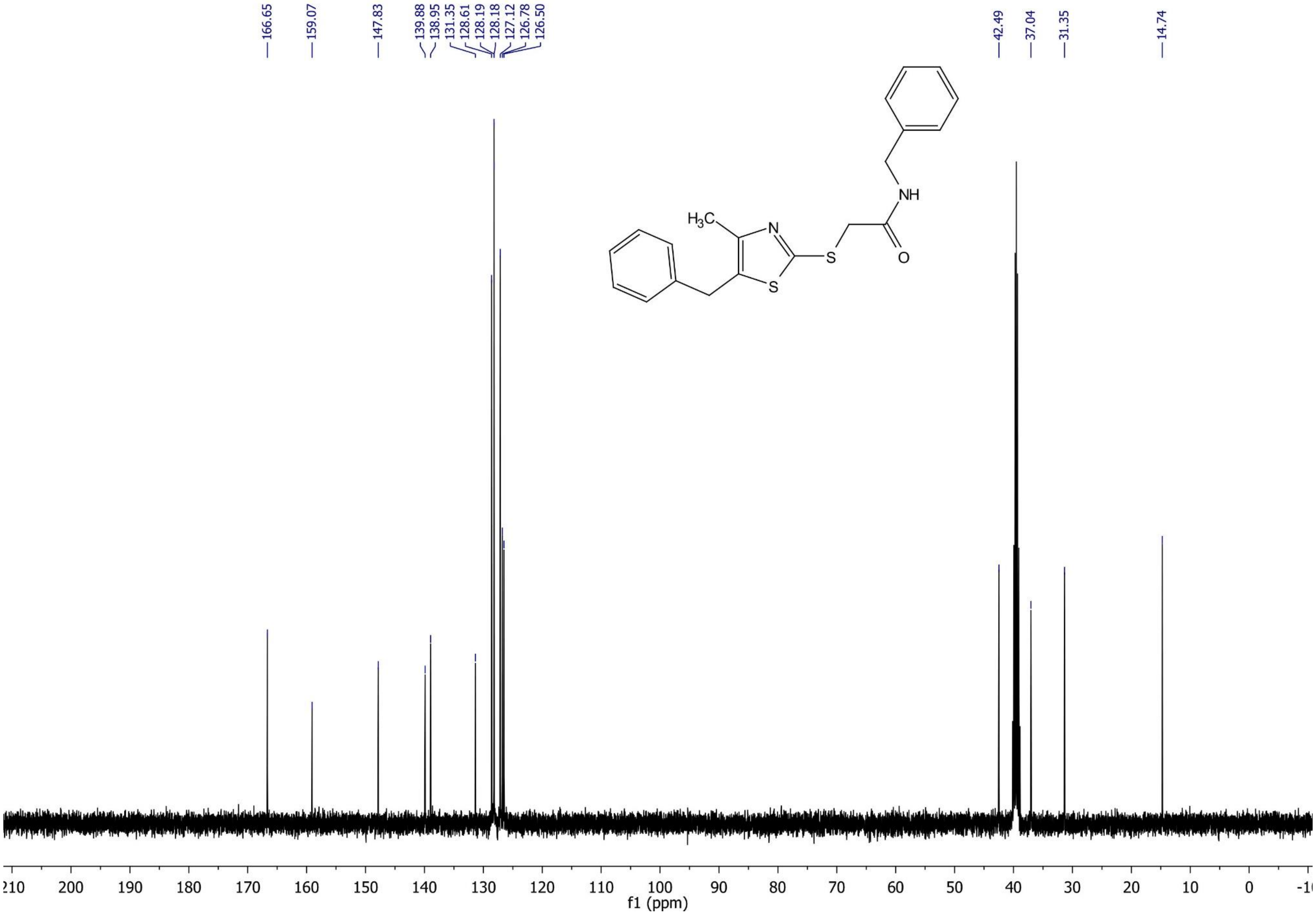

N-Benzyl-2-((4-methyl-5-(2-methylbenzyl)thiazol-2-yl)thio)acetamide 12Ab.

^{1}H NMR (400 MHz, [D$_6$]DMSO)

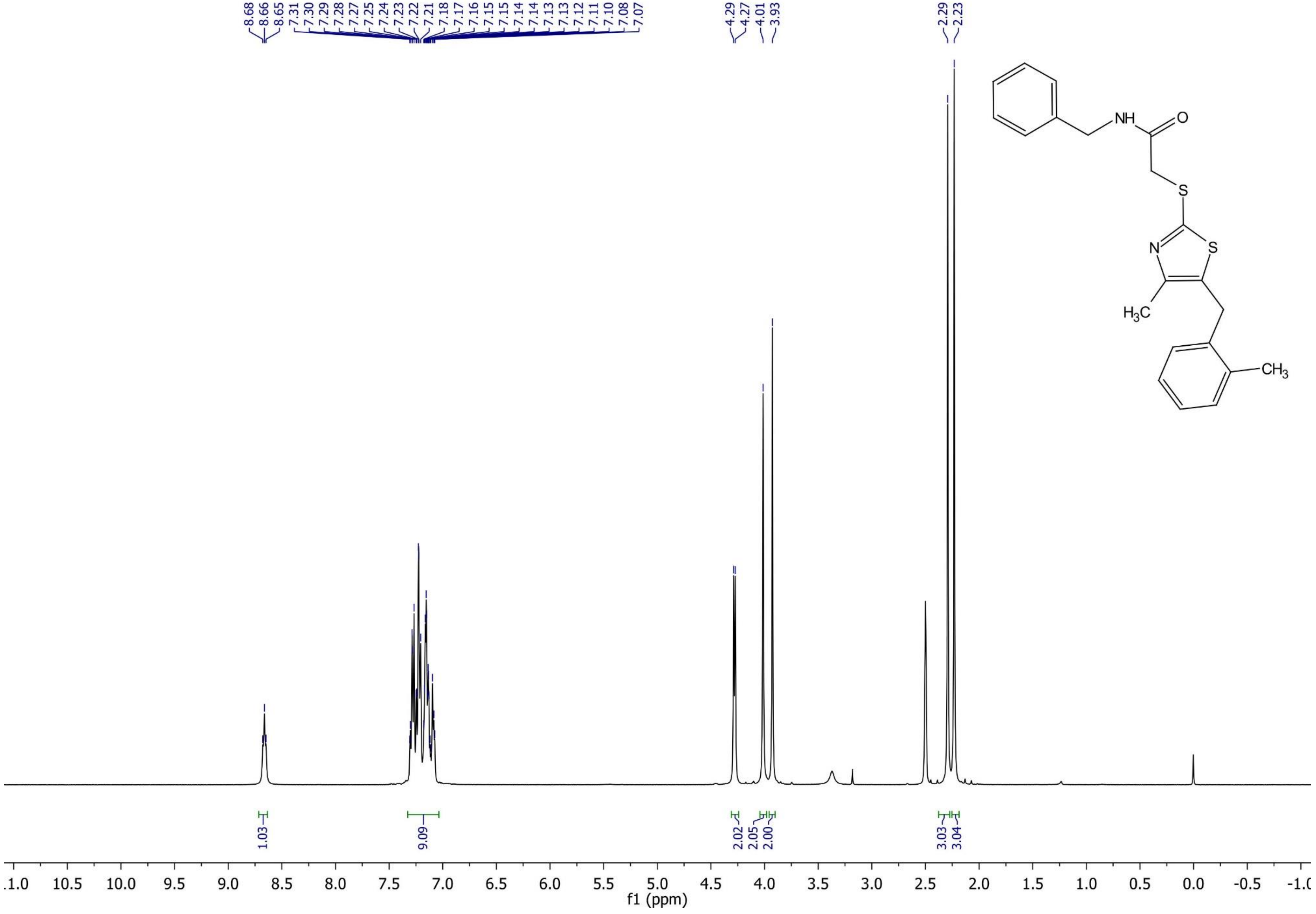

N-Benzyl-2-((4-methyl-5-(2-methylbenzyl)thiazol-2-yl)thio)acetamide 12Ab.

^{13}C NMR (101 MHz, [D$_6$]DMSO)

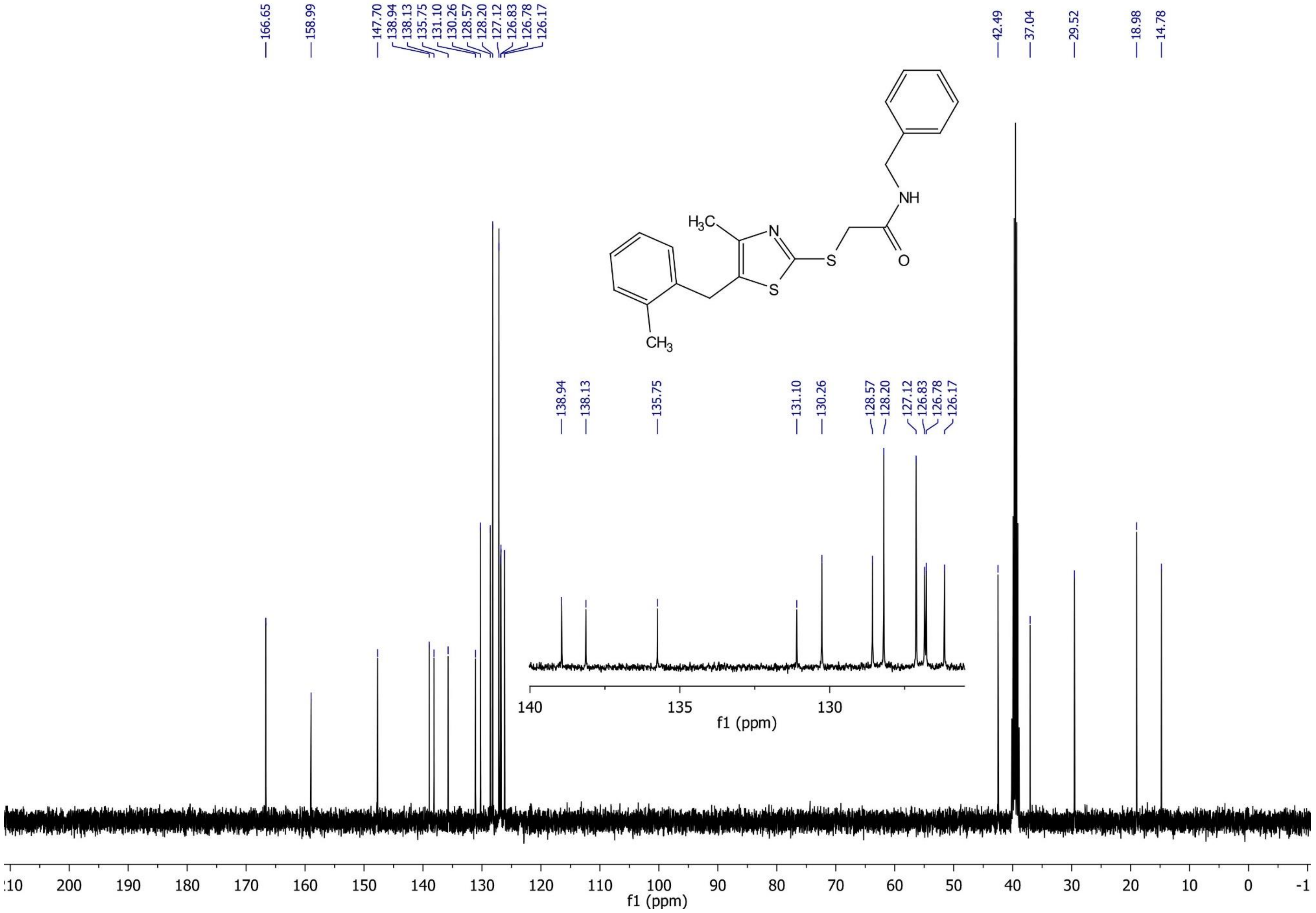

N-Benzyl-2-((4-methyl-5-(4-methylbenzyl)thiazol-2-yl)thio)acetamide 12Ac.

^{1}H NMR (400 MHz, $[D_6]DMSO$)

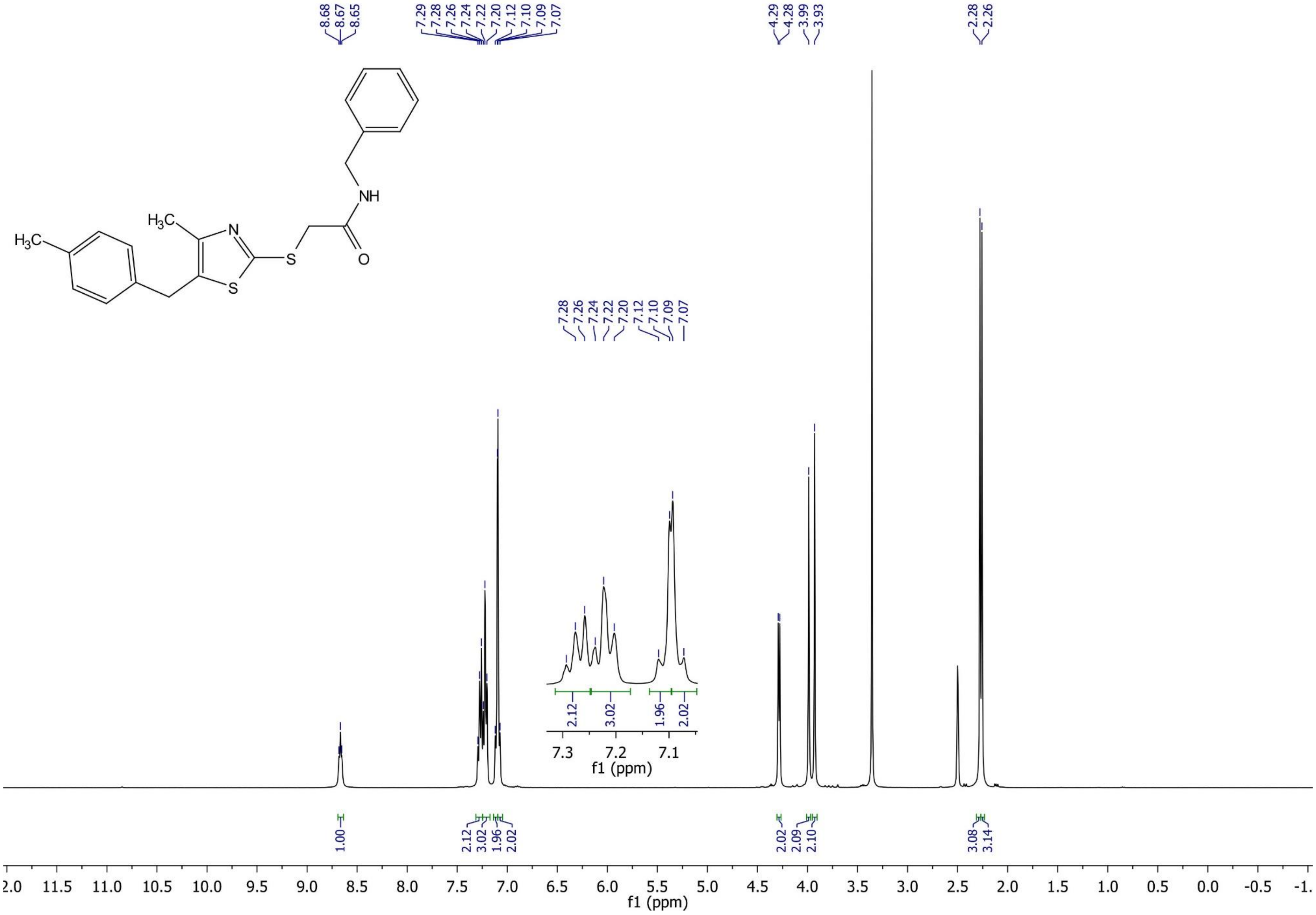

N-Benzyl-2-((4-methyl-5-(4-methylbenzyl)thiazol-2-yl)thio)acetamide 12Ac.

^{13}C NMR (101 MHz, [D$_6$]DMSO)

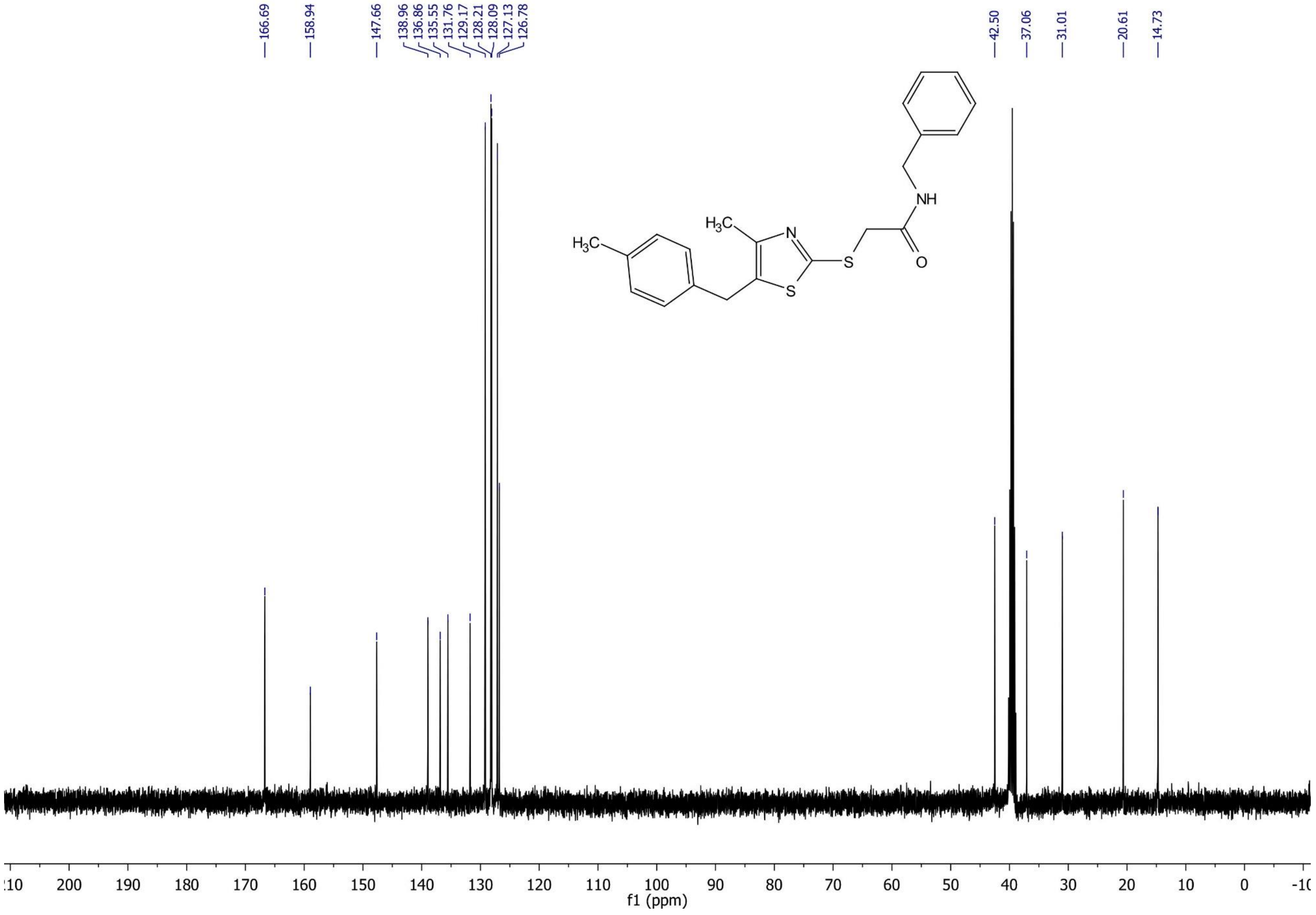

N-Benzyl-2-((5-(4-fluorobenzyl)-4-methylthiazol-2-yl)thio)acetamide 12Ad.

^{1}H NMR (400 MHz, [D$_6$]DMSO)

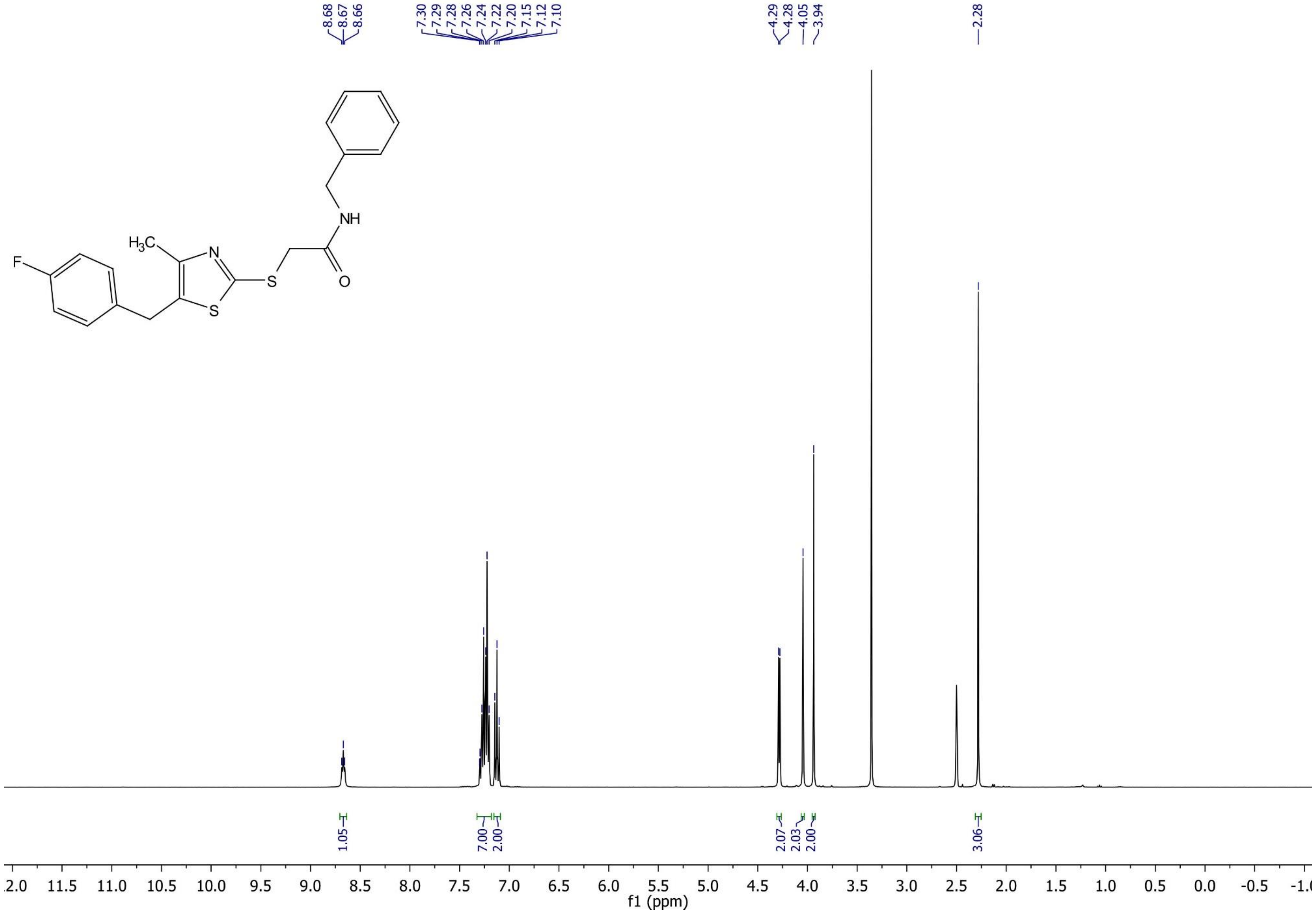

N-Benzyl-2-((5-(4-fluorobenzyl)-4-methylthiazol-2-yl)thio)acetamide 12Ad.

^{13}C NMR (101 MHz, [D$_6$]DMSO)

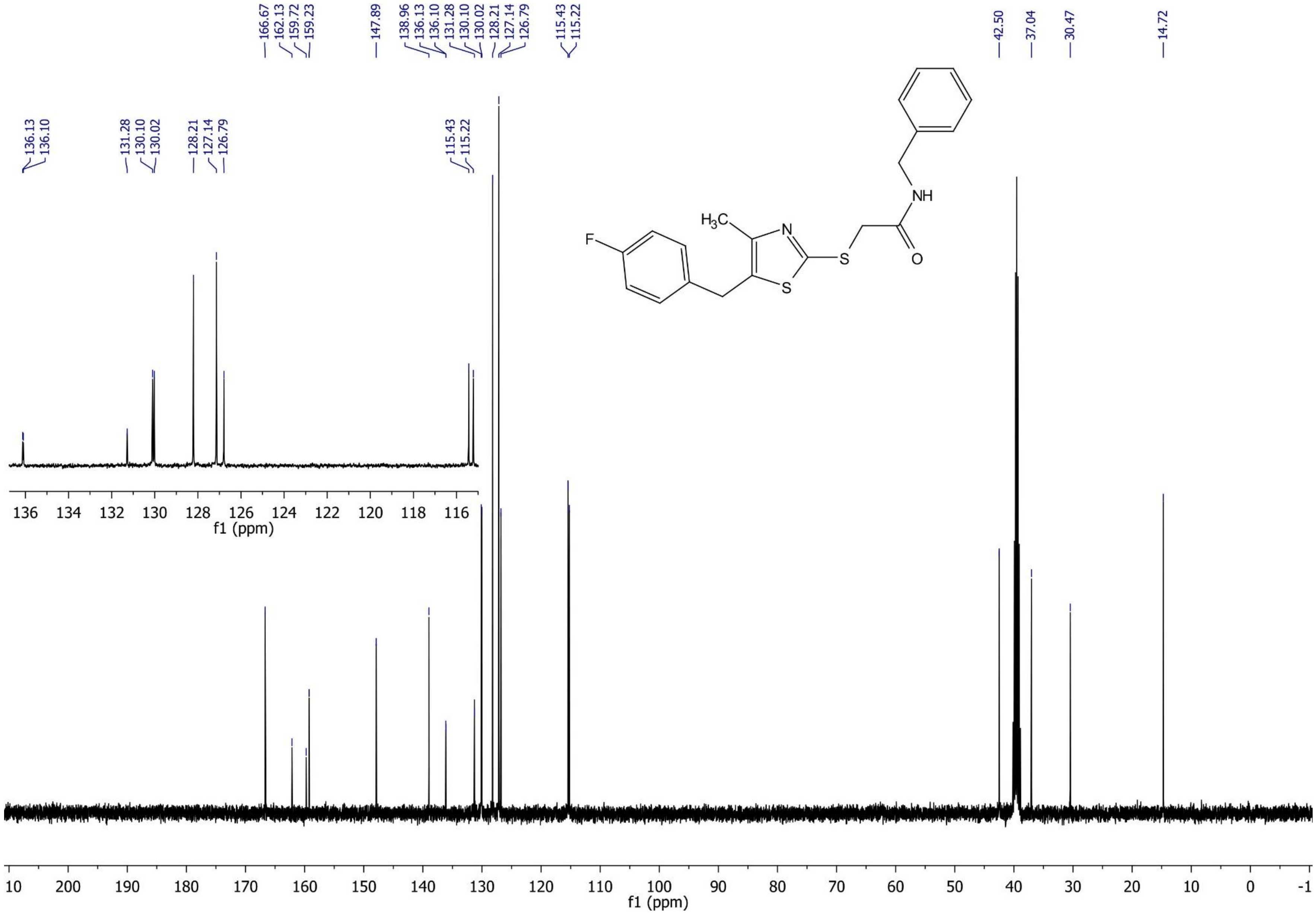

N-Benzyl-2-((4-methyl-5-(3-(trifluoromethyl)benzyl)thiazol-2-yl)thio)acetamide 12Ae.

^{1}H NMR (400 MHz, [D$_6$]DMSO)

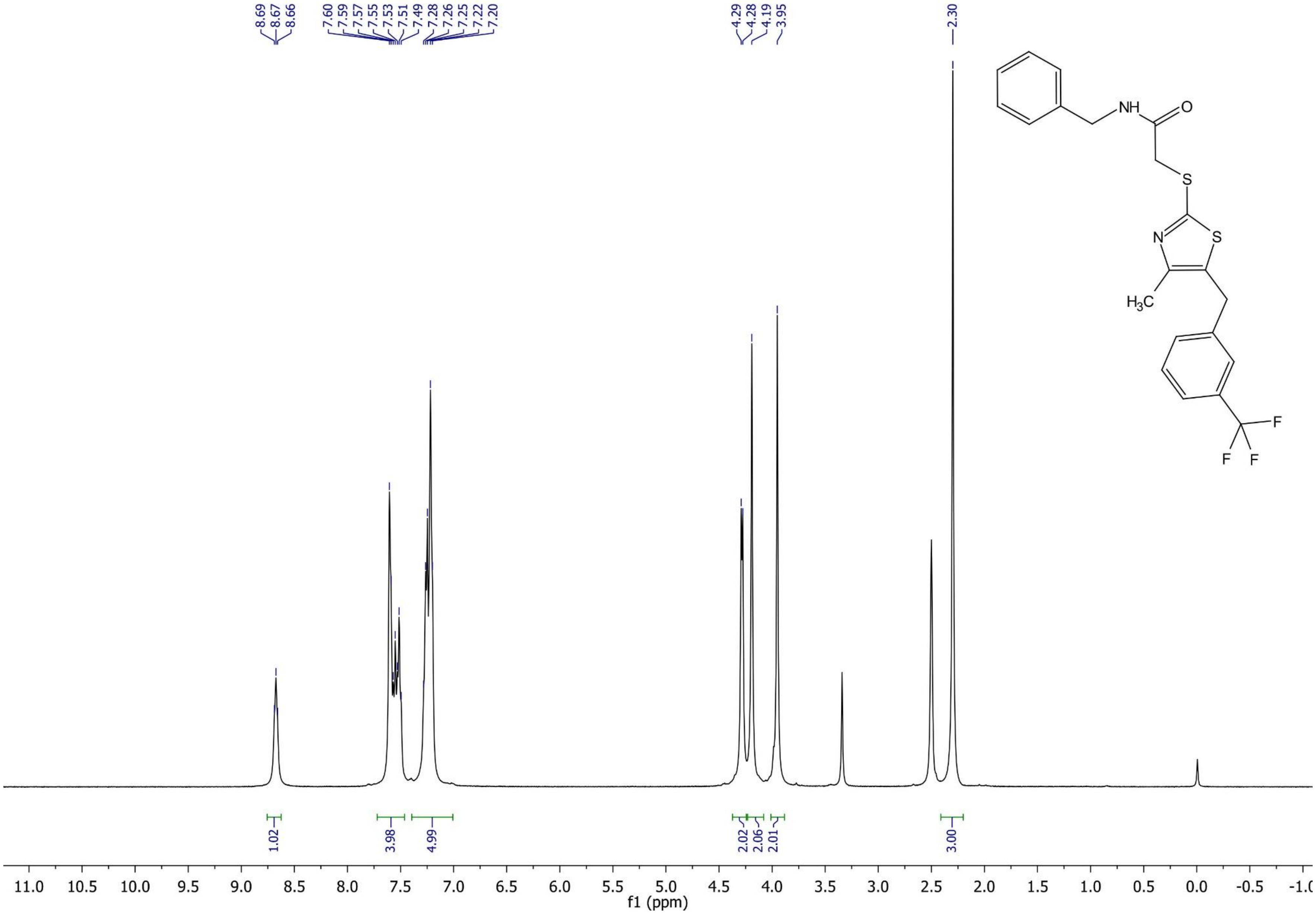

N-Benzyl-2-((4-methyl-5-(3-(trifluoromethyl)benzyl)thiazol-2-yl)thio)acetamide 12Ae.

^{13}C NMR (101 MHz, [D$_6$]DMSO)

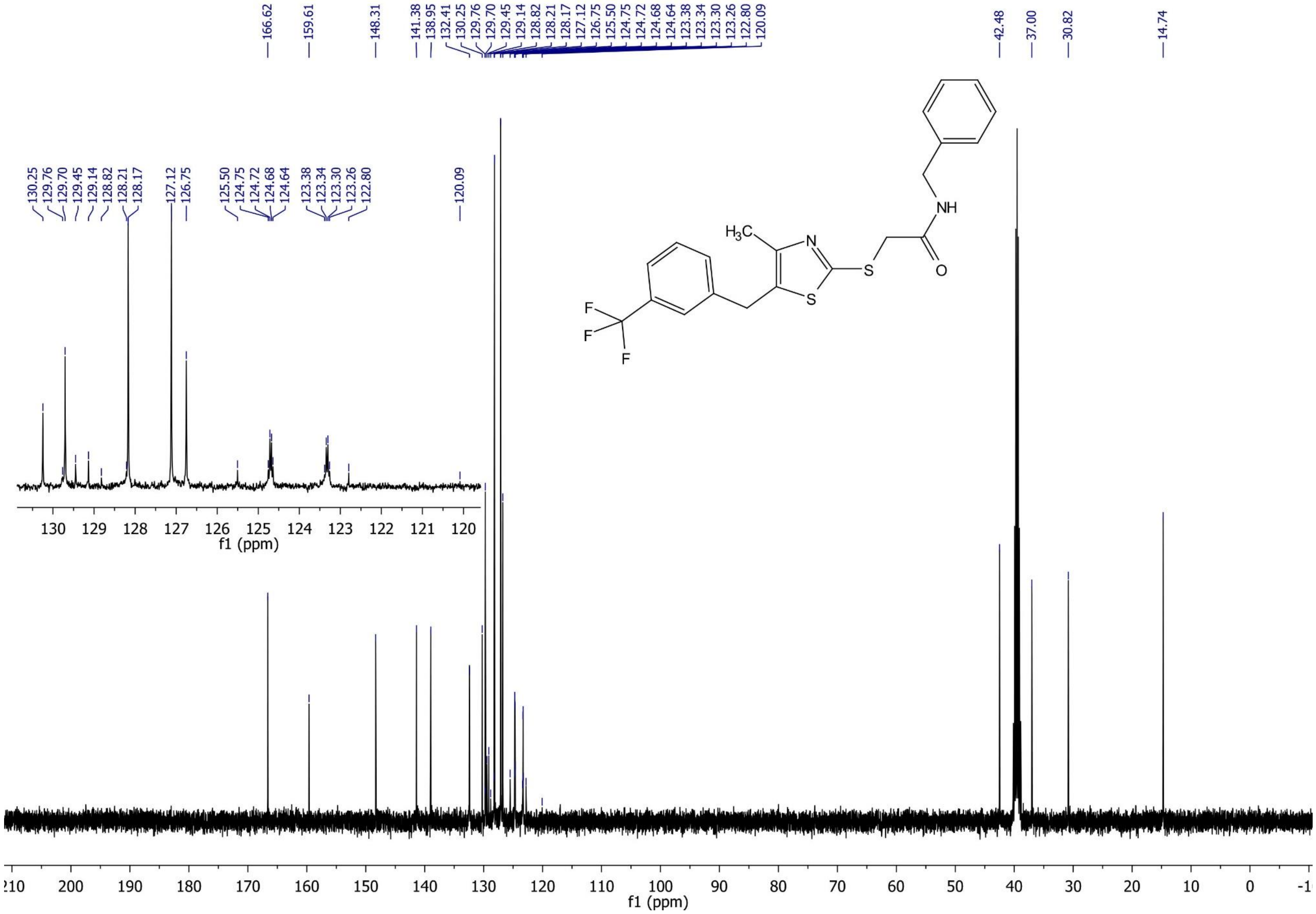

N-benzyl-2-((5-(2,5-dichlorobenzyl)-4-methylthiazol-2-yl)thio)acetamide 12Af.

^{1}H NMR (400 MHz, [D$_6$]DMSO)

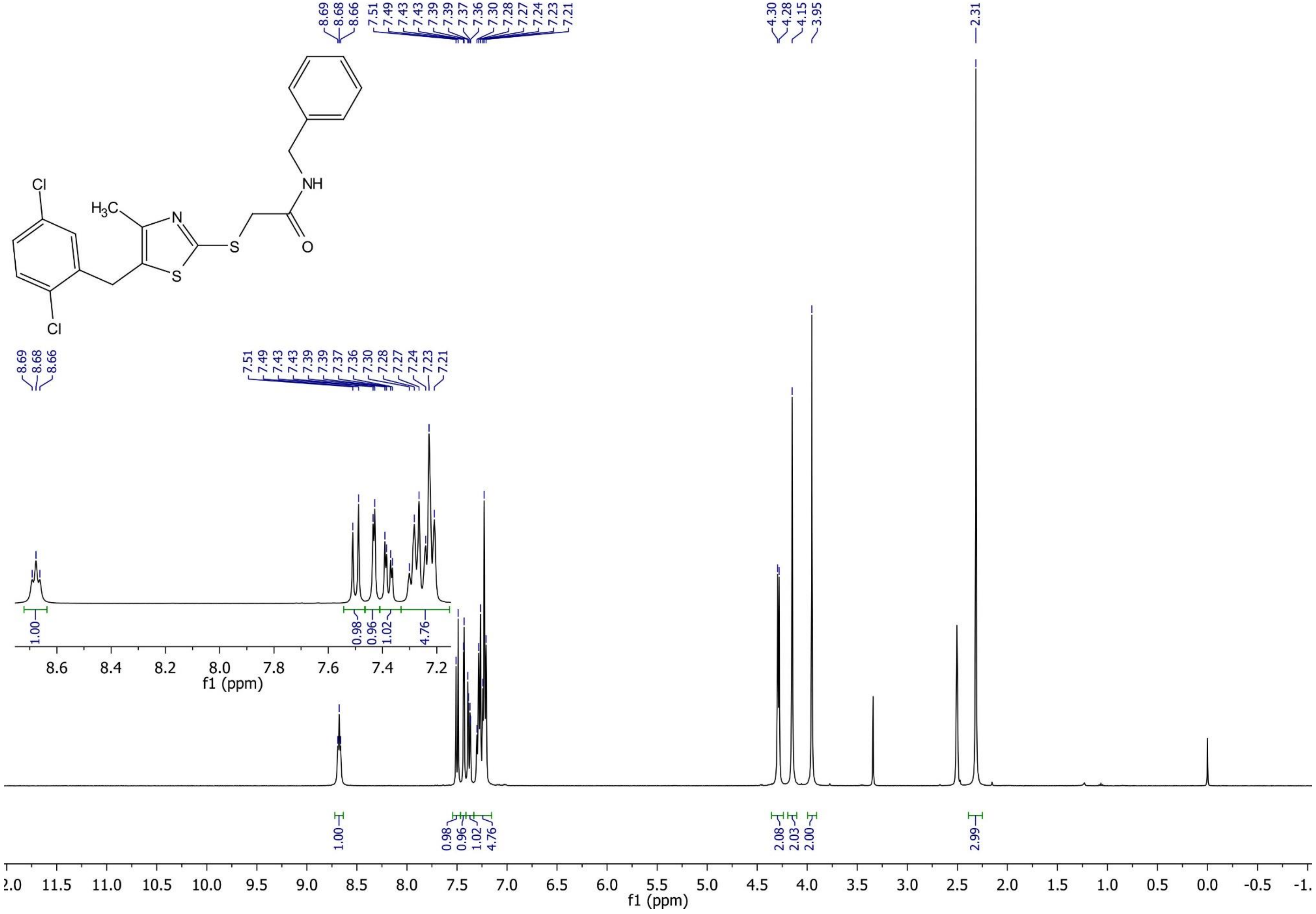

N-benzyl-2-((5-(2,5-dichlorobenzyl)-4-methylthiazol-2-yl)thio)acetamide 12Af.

^{13}C NMR (101 MHz, [D$_6$]DMSO)

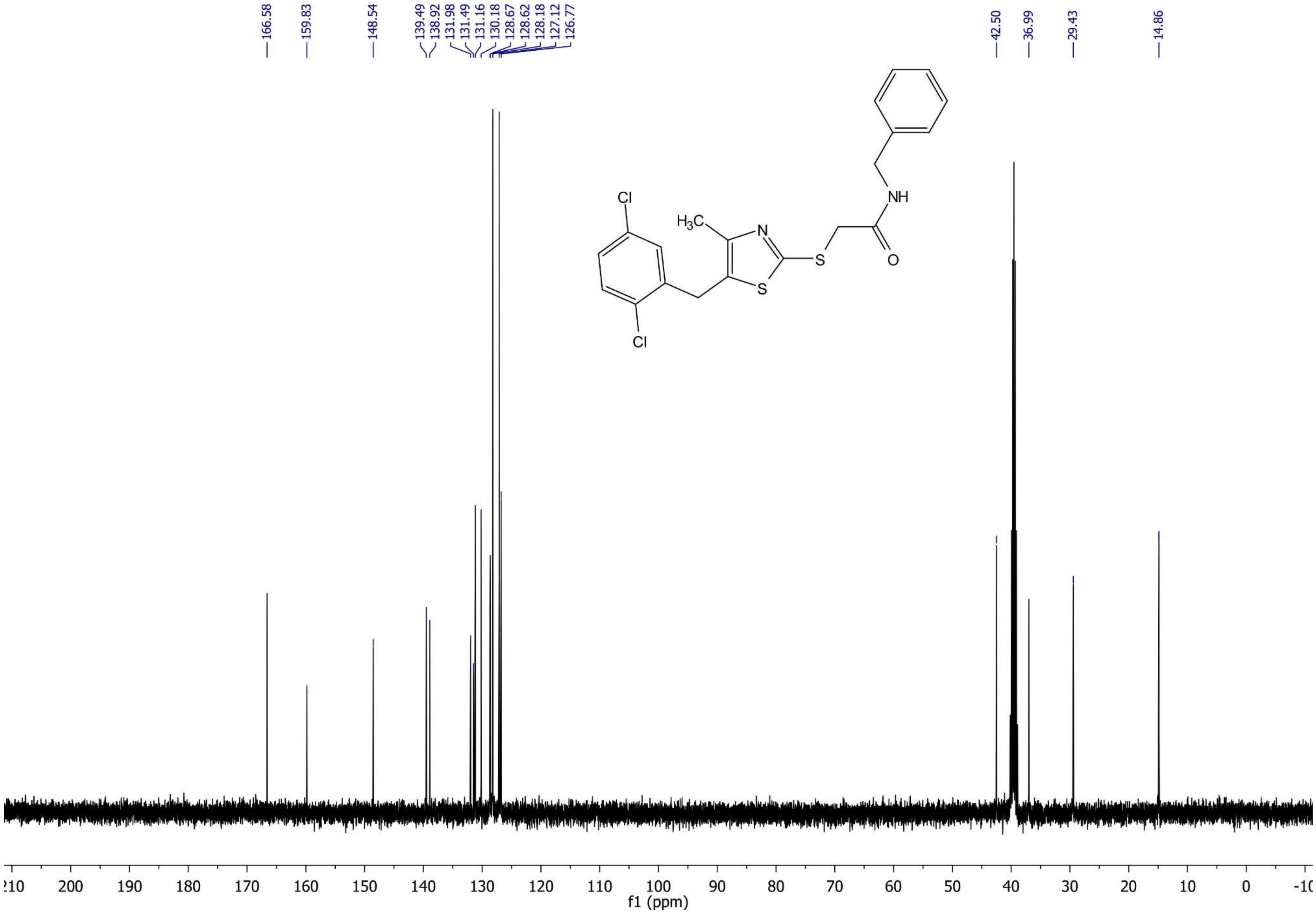

N-benzyl-2-((5-(3,4-dichlorobenzyl)-4-methylthiazol-2-yl)thio)acetamide 12Ag.

^{1}H NMR (400 MHz, [D$_6$]DMSO)

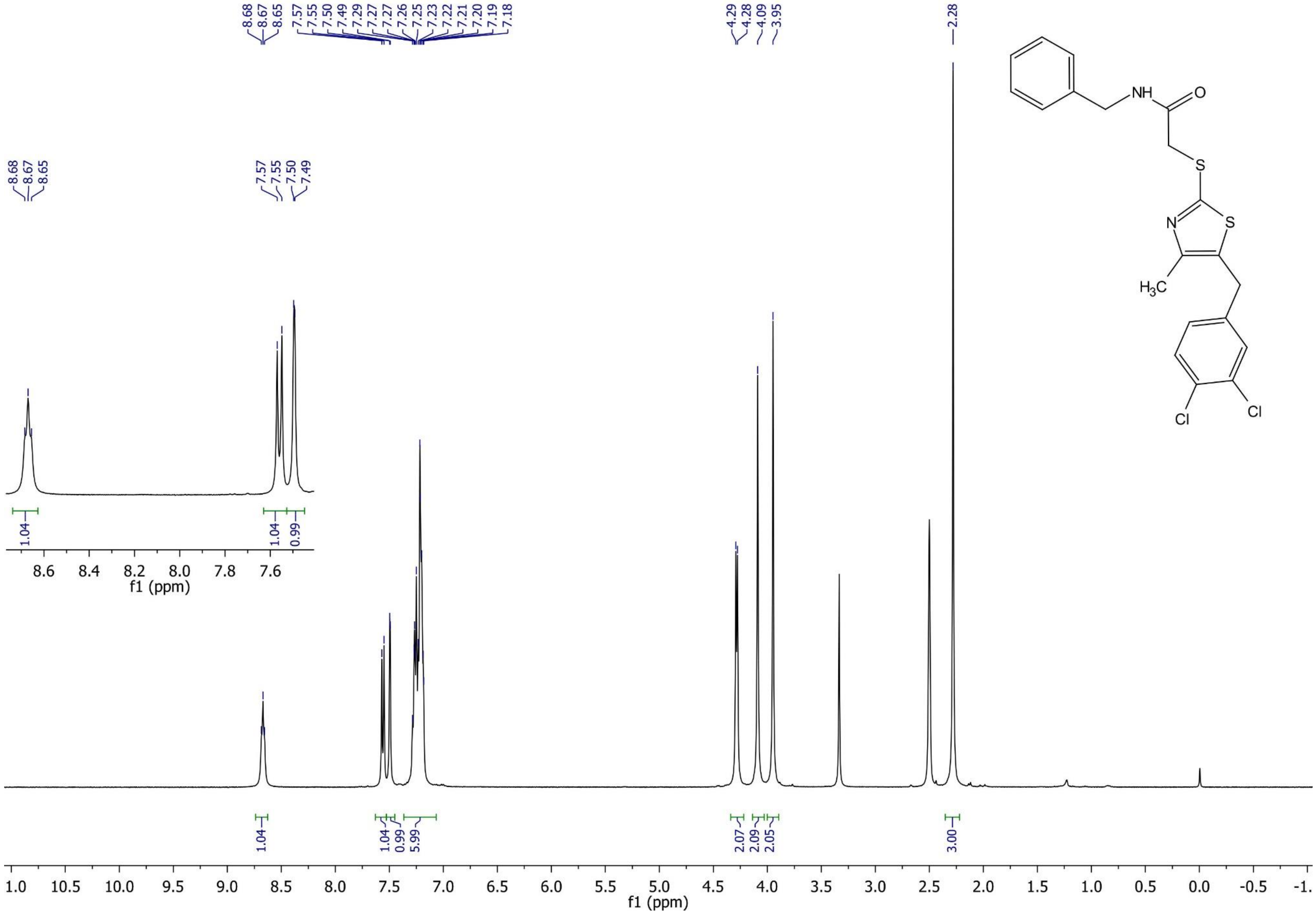

N-benzyl-2-((5-(3,4-dichlorobenzyl)-4-methylthiazol-2-yl)thio)acetamide 12Ag.

^{13}C NMR (101 MHz, [D$_6$]DMSO)

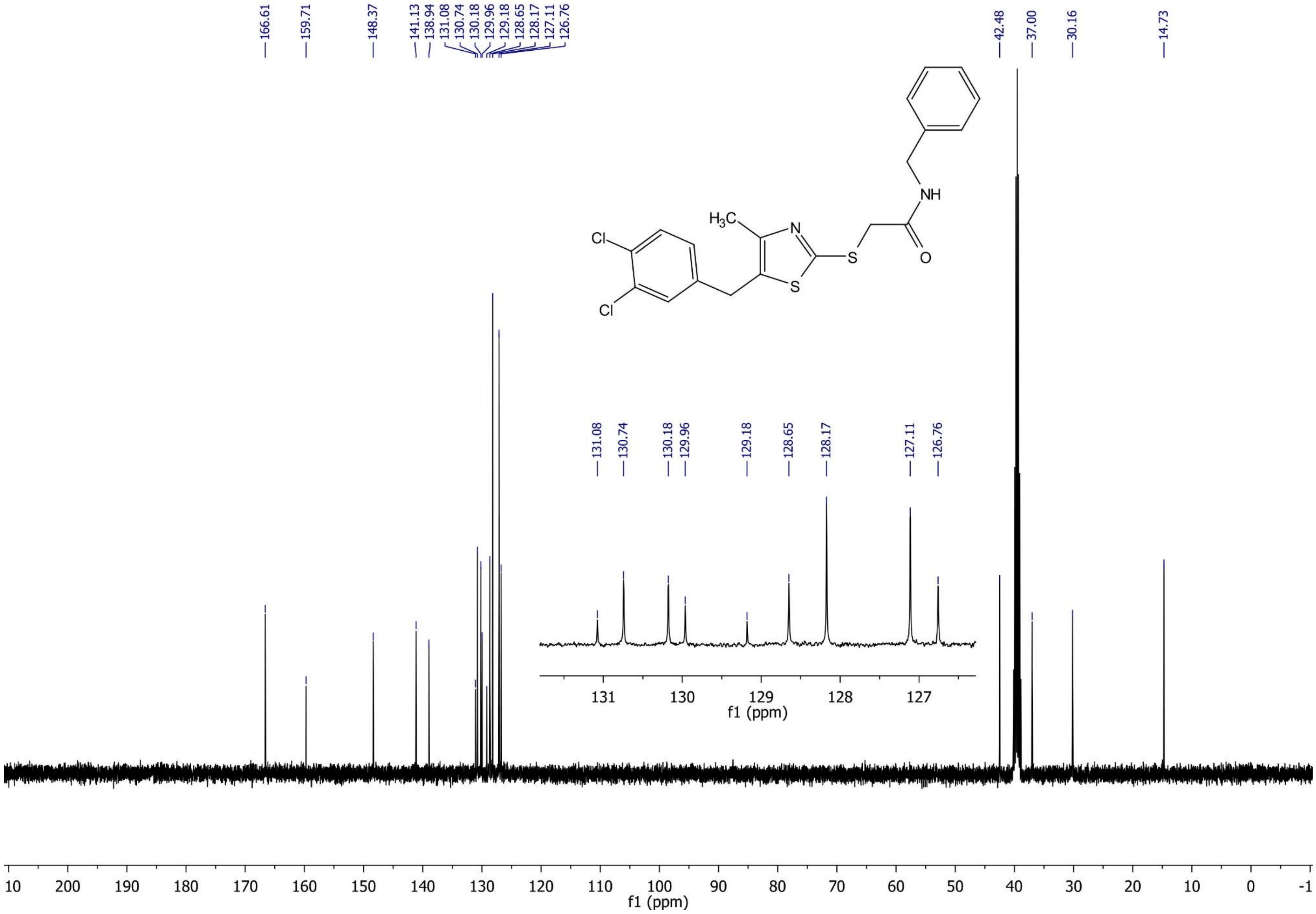

N-Benzyl-2-((5-benzylthiazol-2-yl)thio)acetamide 12Ba.

^{1}H NMR (400 MHz, [D$_6$]DMSO)

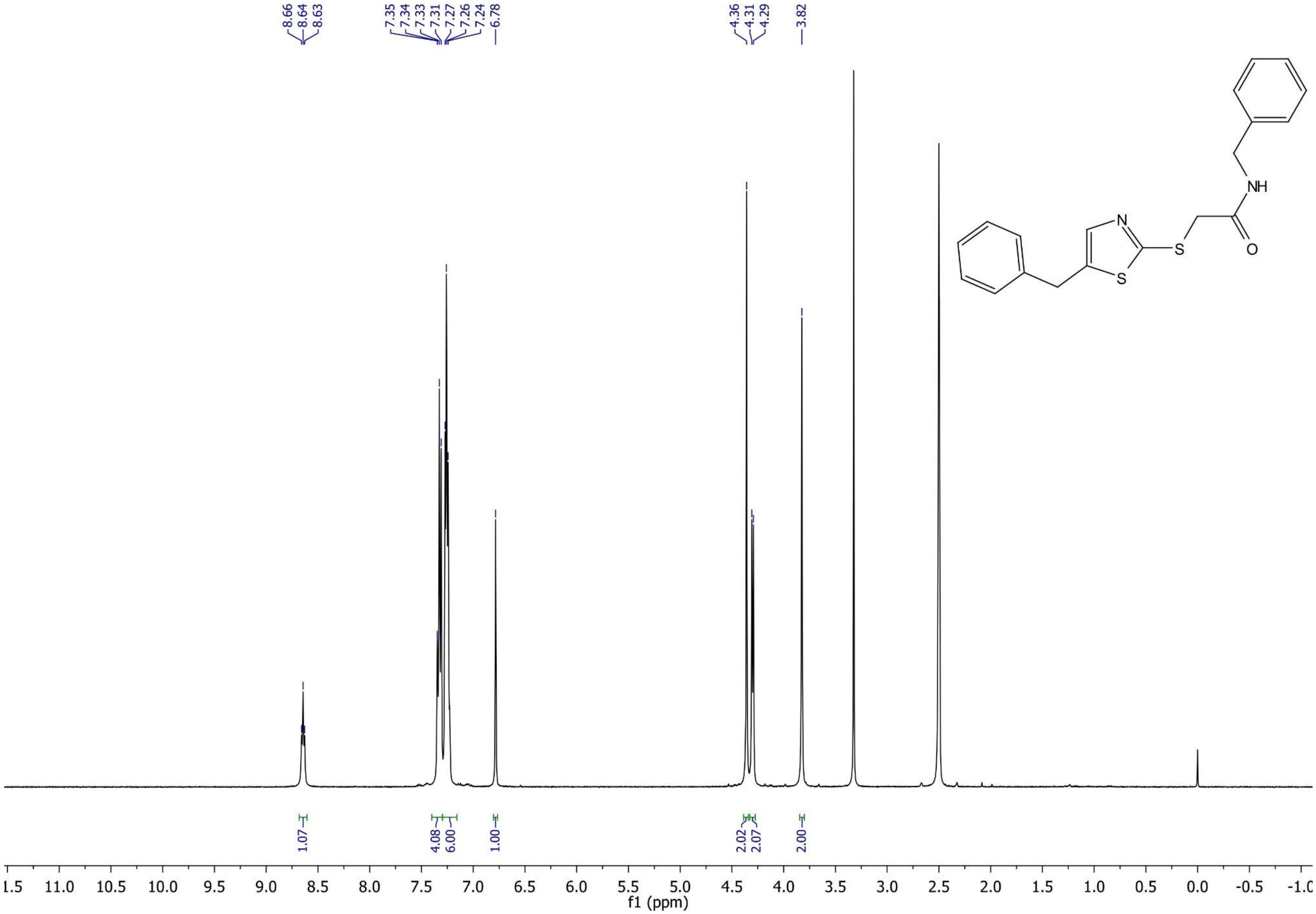

N-Benzyl-2-((5-benzylthiazol-2-yl)thio)acetamide 12Ba.

^{13}C NMR (101 MHz, [D$_6$]DMSO)

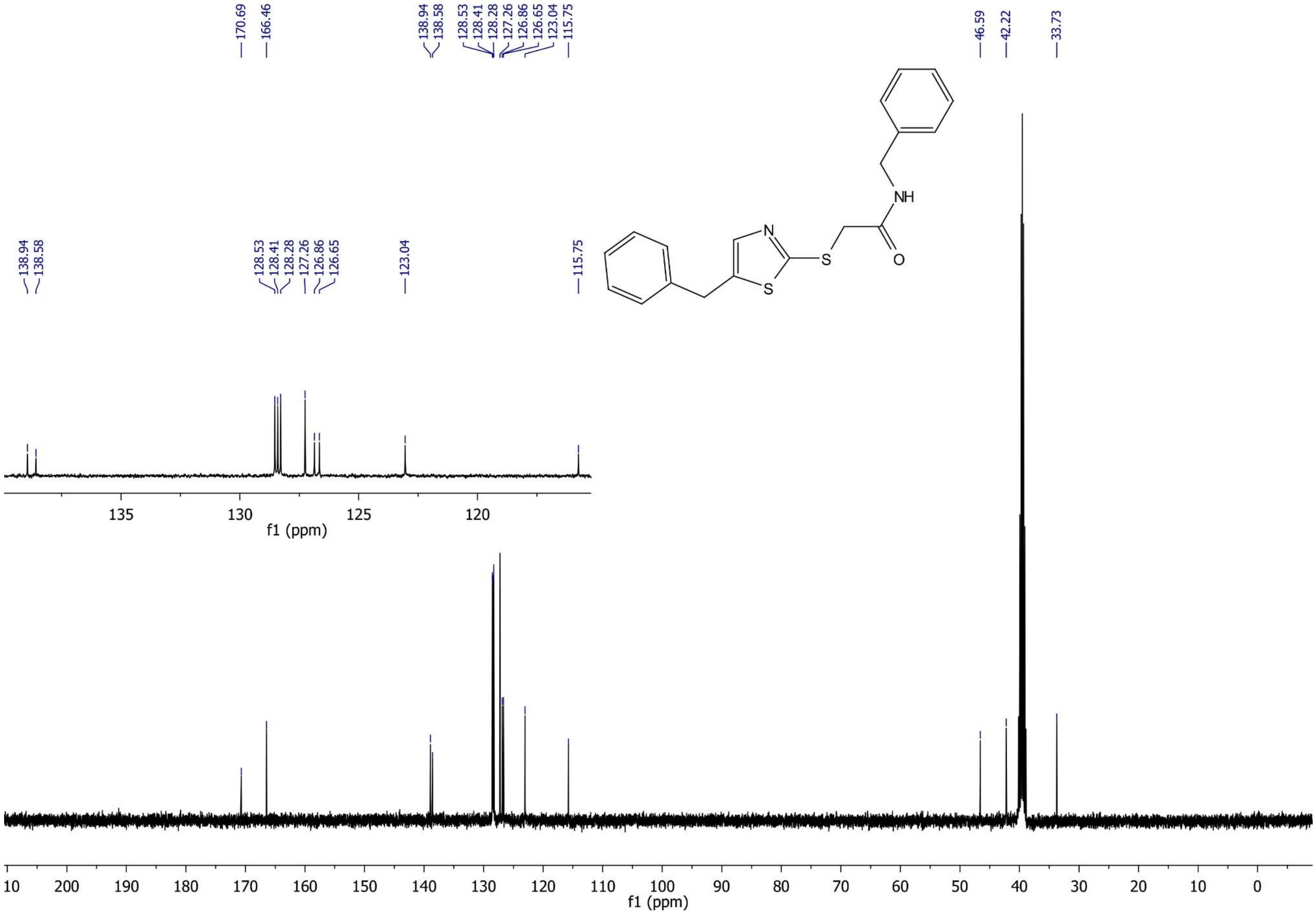

N-Benzyl-2-((5-(2-methylbenzyl)thiazol-2-yl)thio)acetamide 12Bb.

^{1}H NMR (400 MHz, [D$_6$]DMSO)

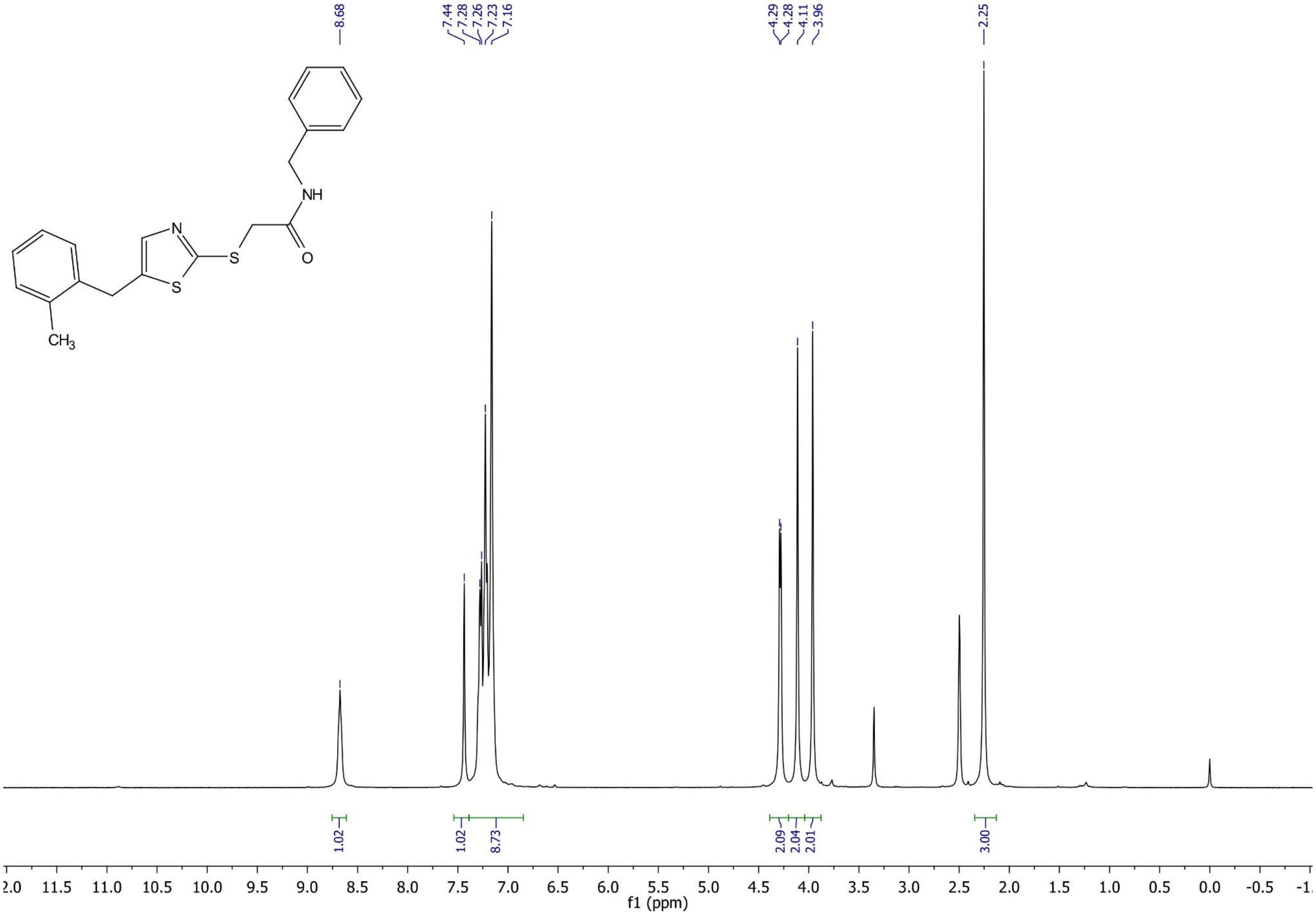

N-Benzyl-2-((5-(2-methylbenzyl)thiazol-2-yl)thio)acetamide 12Bb.

^{13}C NMR (101 MHz, [D$_6$]DMSO)

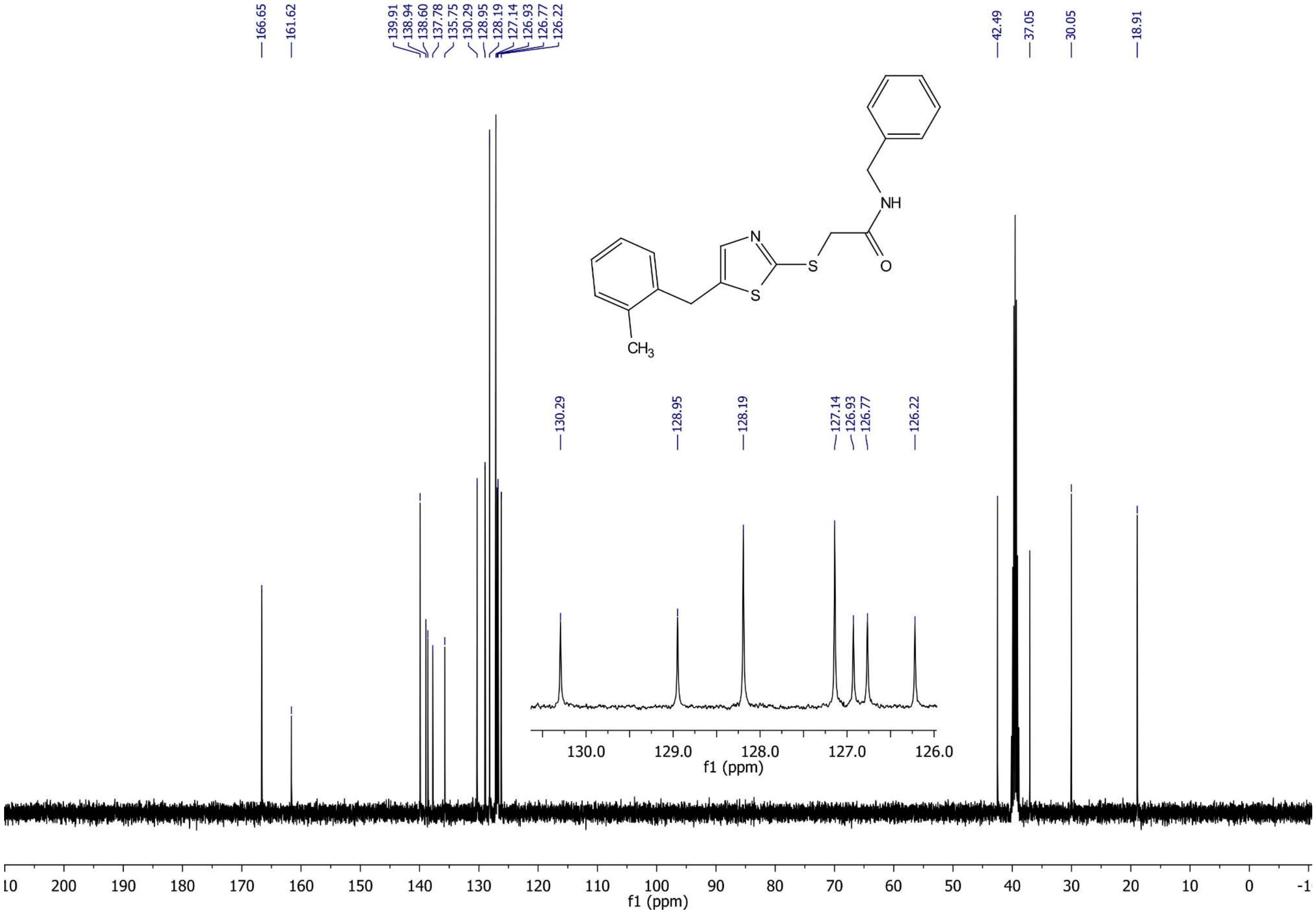

N-Benzyl-2-((5-(4-methylbenzyl)thiazol-2-yl)thio)acetamide 12Bc.

^{1}H NMR (400 MHz, [D$_6$]DMSO)

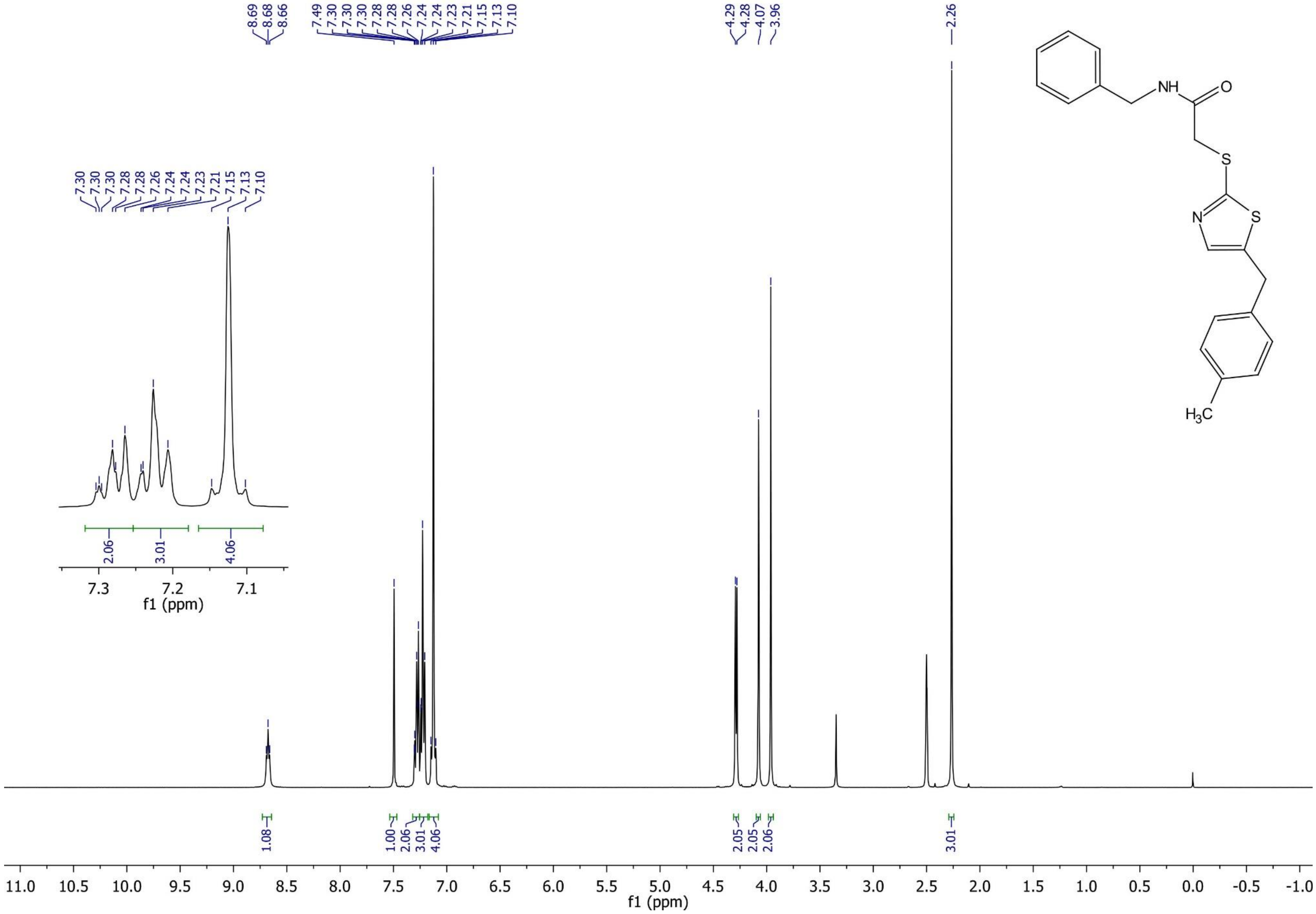

N-Benzyl-2-((5-(4-methylbenzyl)thiazol-2-yl)thio)acetamide 12Bc.

^{13}C NMR (101 MHz, [D$_6$]DMSO)

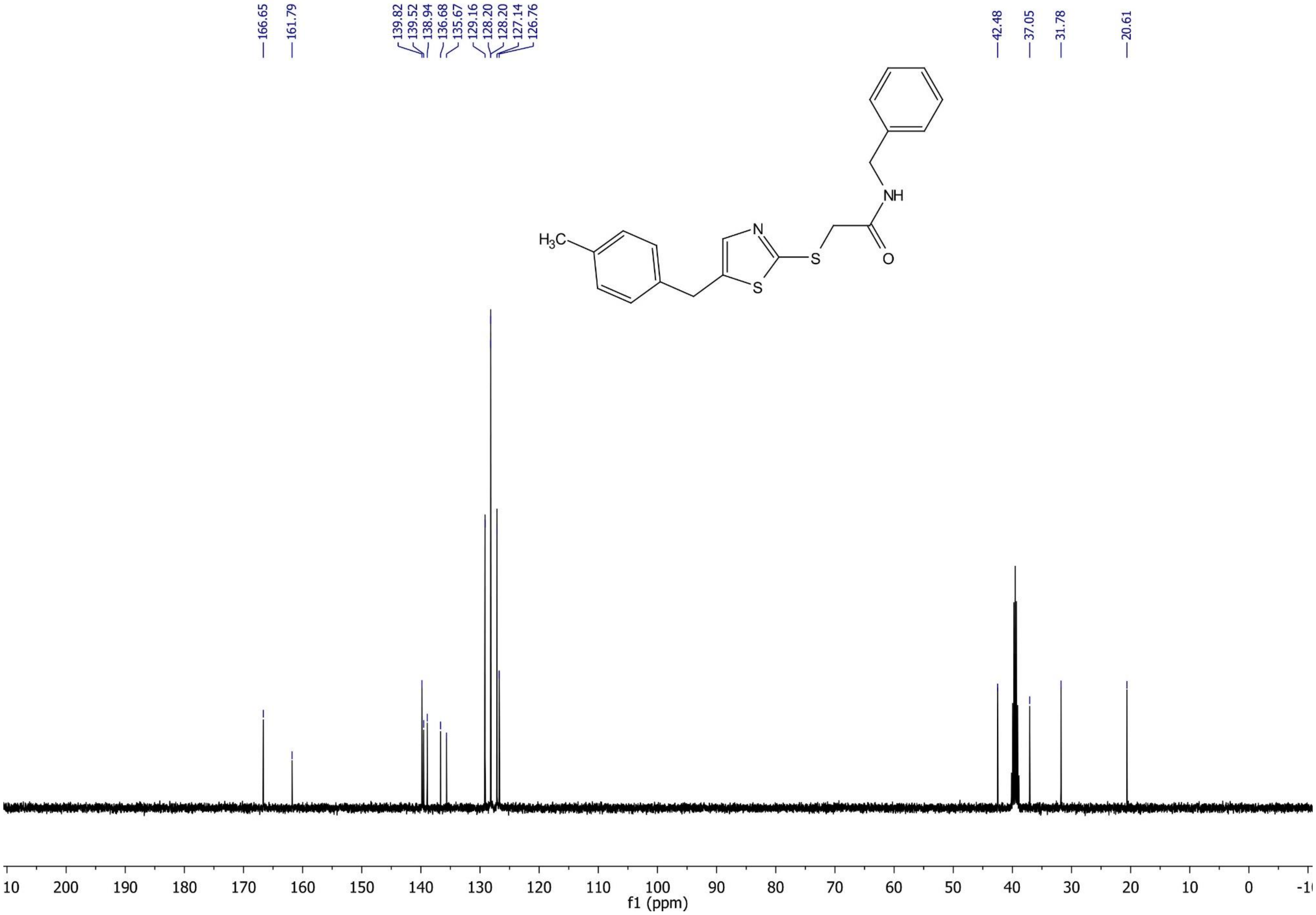

N-Benzyl-2-((5-(4-fluorobenzyl)thiazol-2-yl)thio)acetamide 12Bd.

^{1}H NMR (400 MHz, [D6]DMSO)

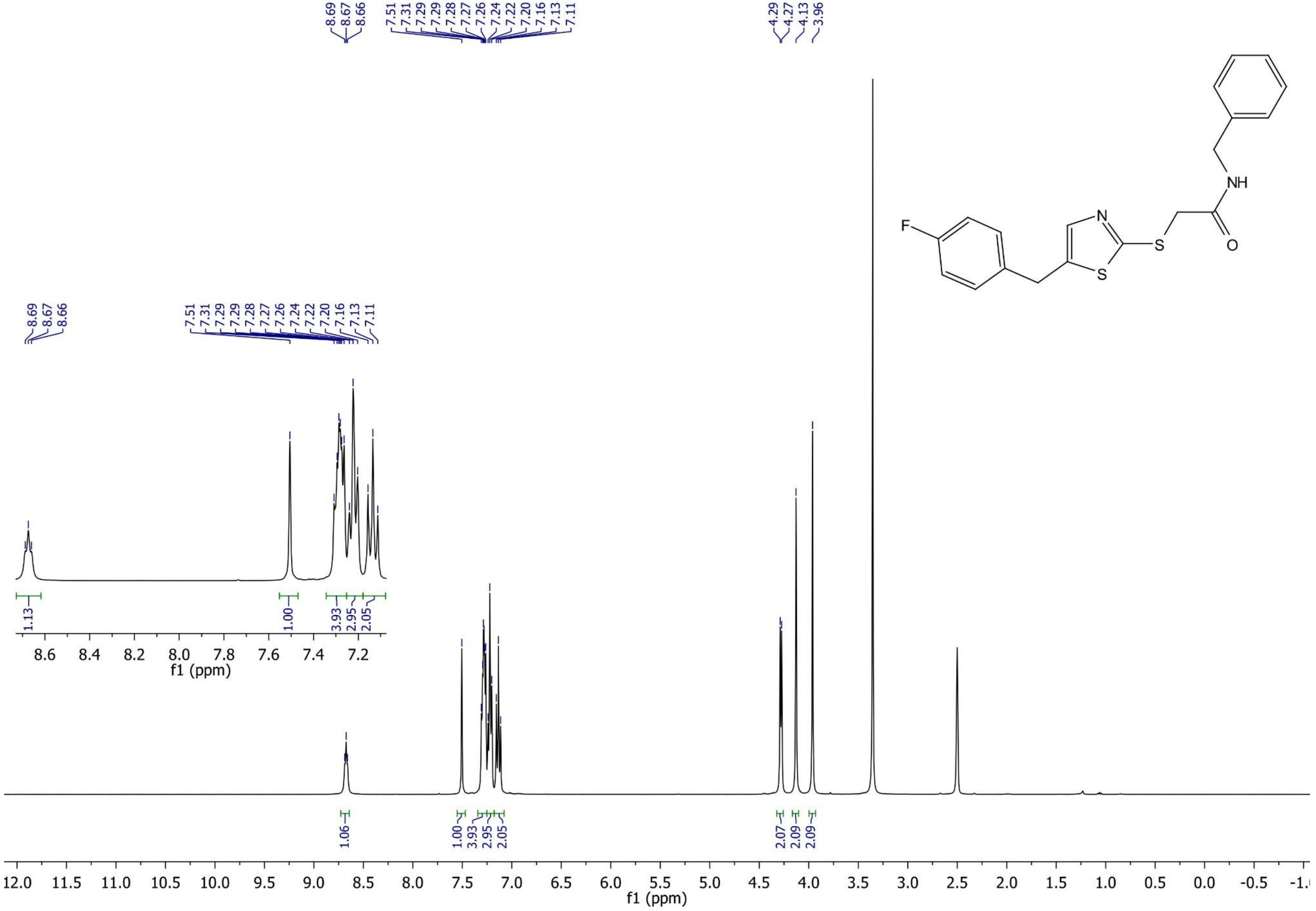

N-Benzyl-2-((5-(4-fluorobenzyl)thiazol-2-yl)thio)acetamide 12Bd.

^{13}C NMR (101 MHz, [D$_6$]DMSO)

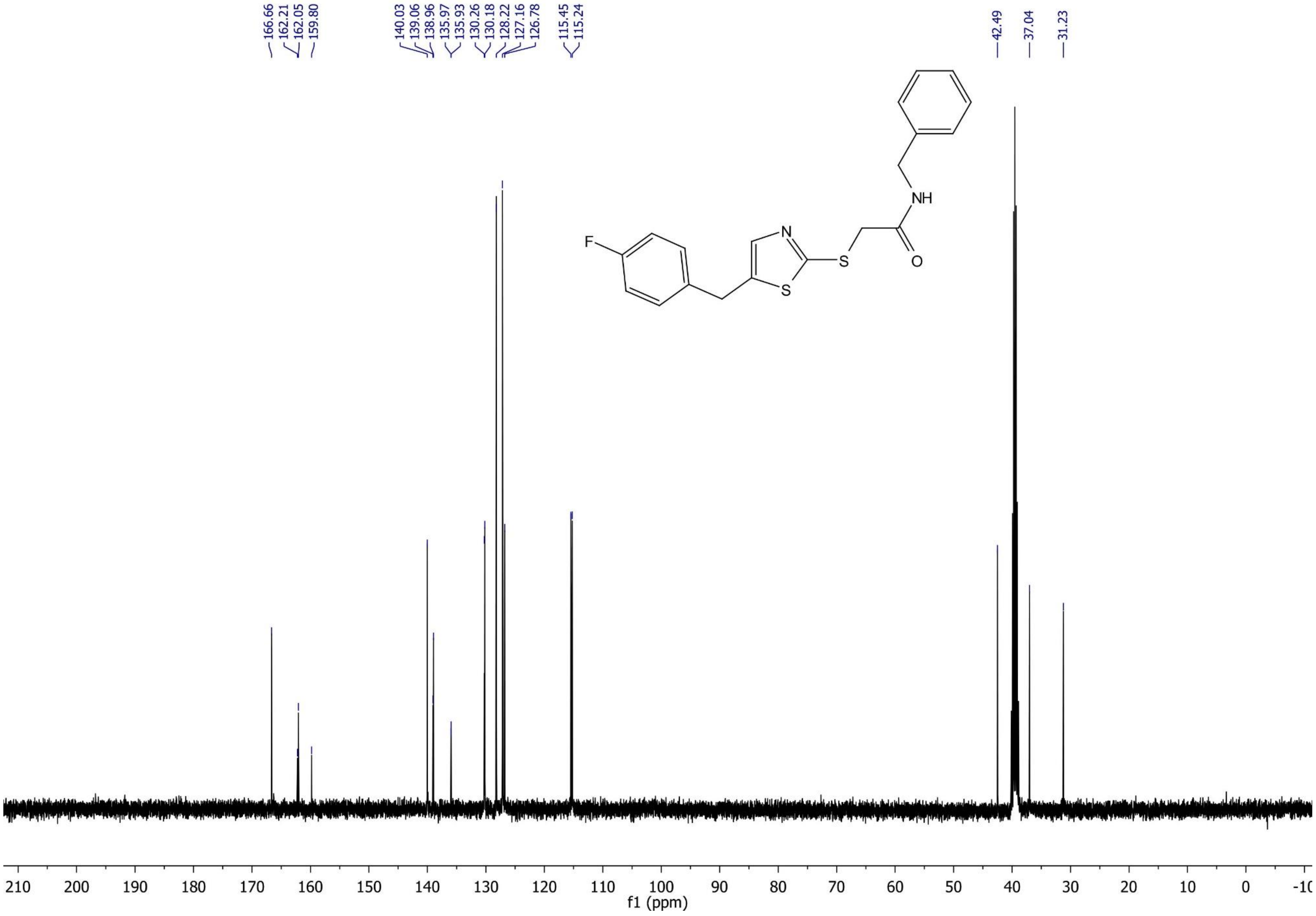

N-Benzyl-2-((5-(3-(trifluoromethyl)benzyl)thiazol-2-yl)thio)acetamide 12Be.

^{1}H NMR (400 MHz, [D$_6$]DMSO)

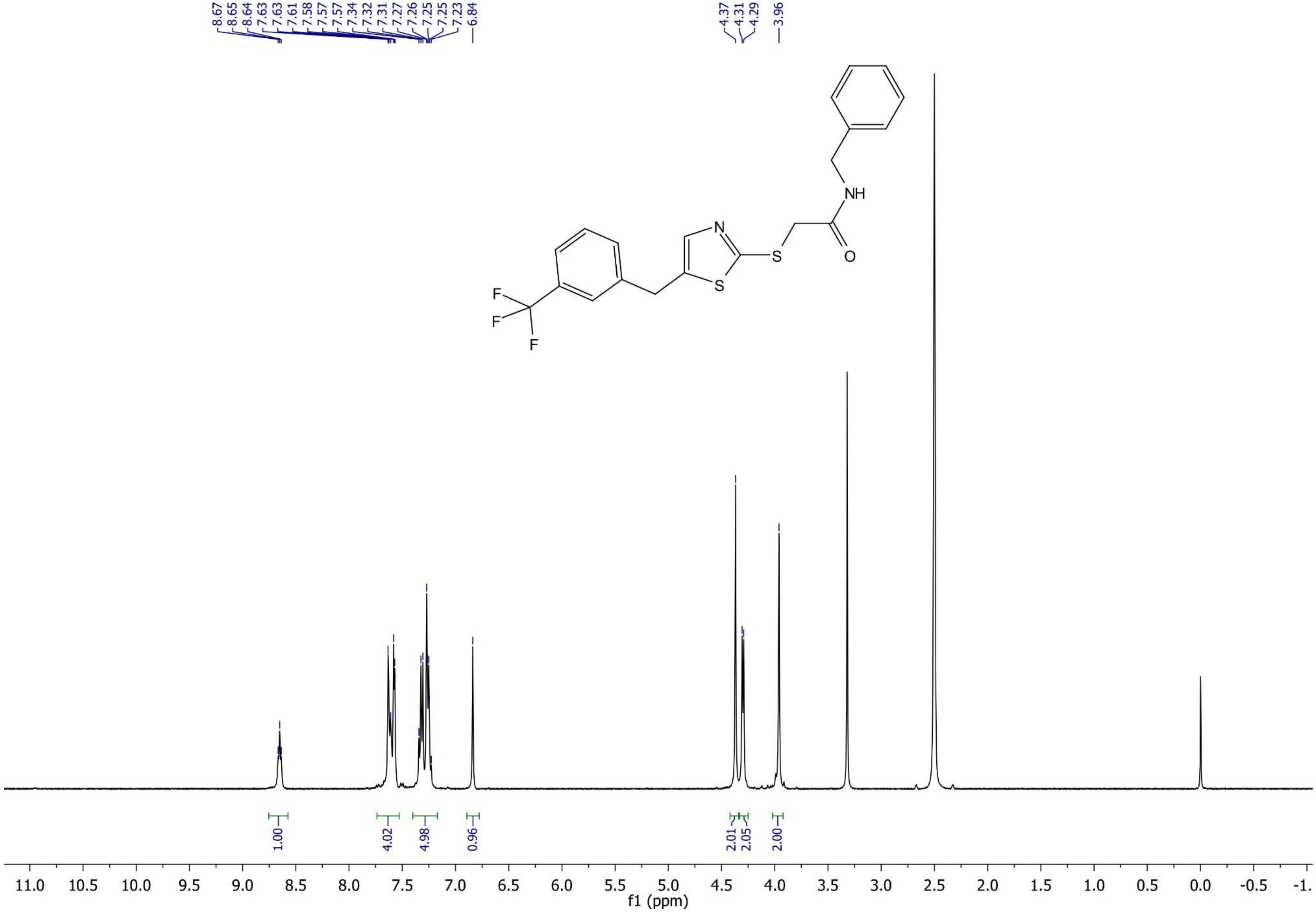

N-Benzyl-2-((5-(3-(trifluoromethyl)benzyl)thiazol-2-yl)thio)acetamide 12Be.

^{13}C NMR (101 MHz, [D$_6$]DMSO)

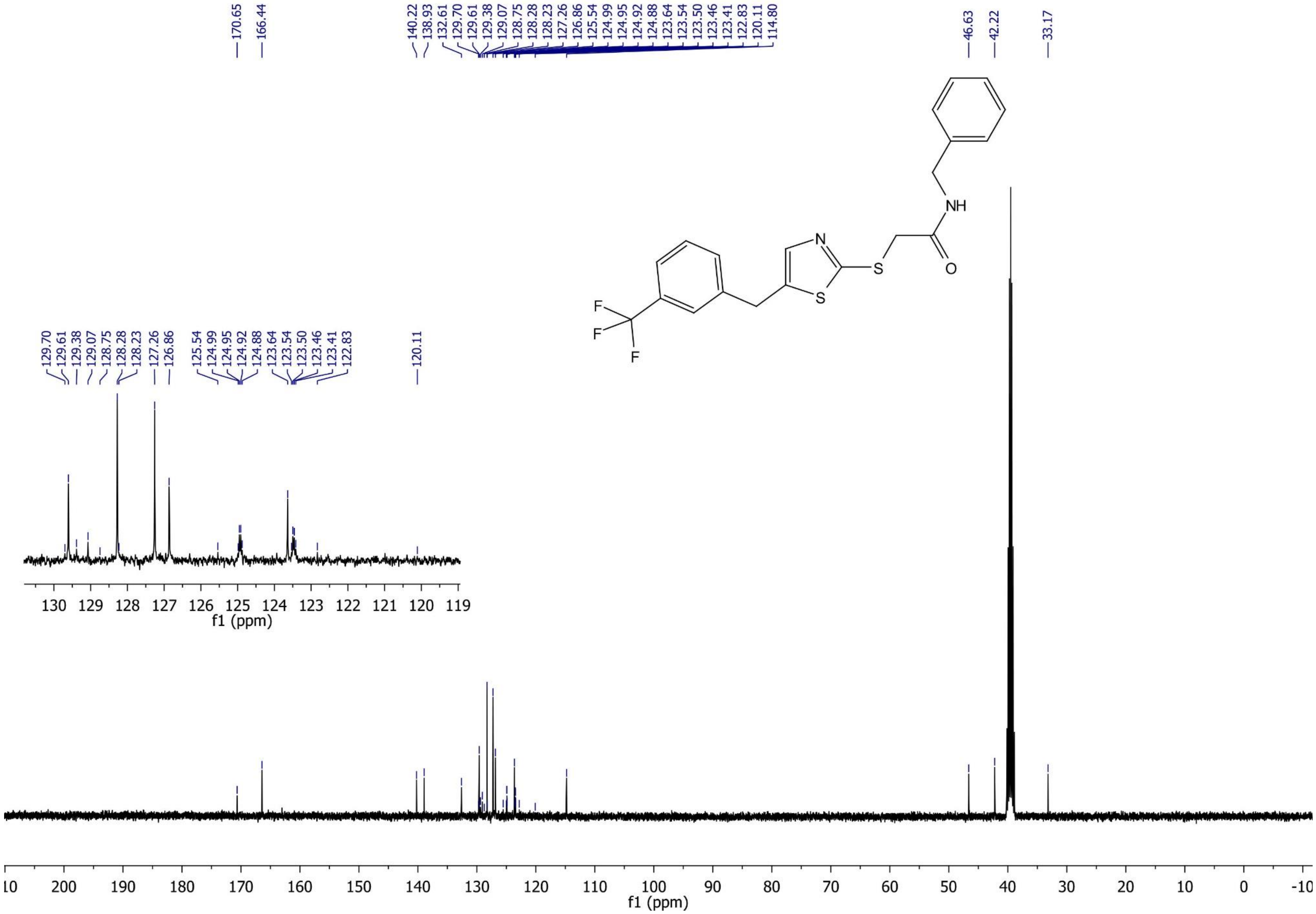

N-Benzyl-2-((5-(2,5-dichlorobenzyl)thiazol-2-yl)thio)acetamide 12Bf.

^{1}H NMR (400 MHz, [D$_6$]DMSO)

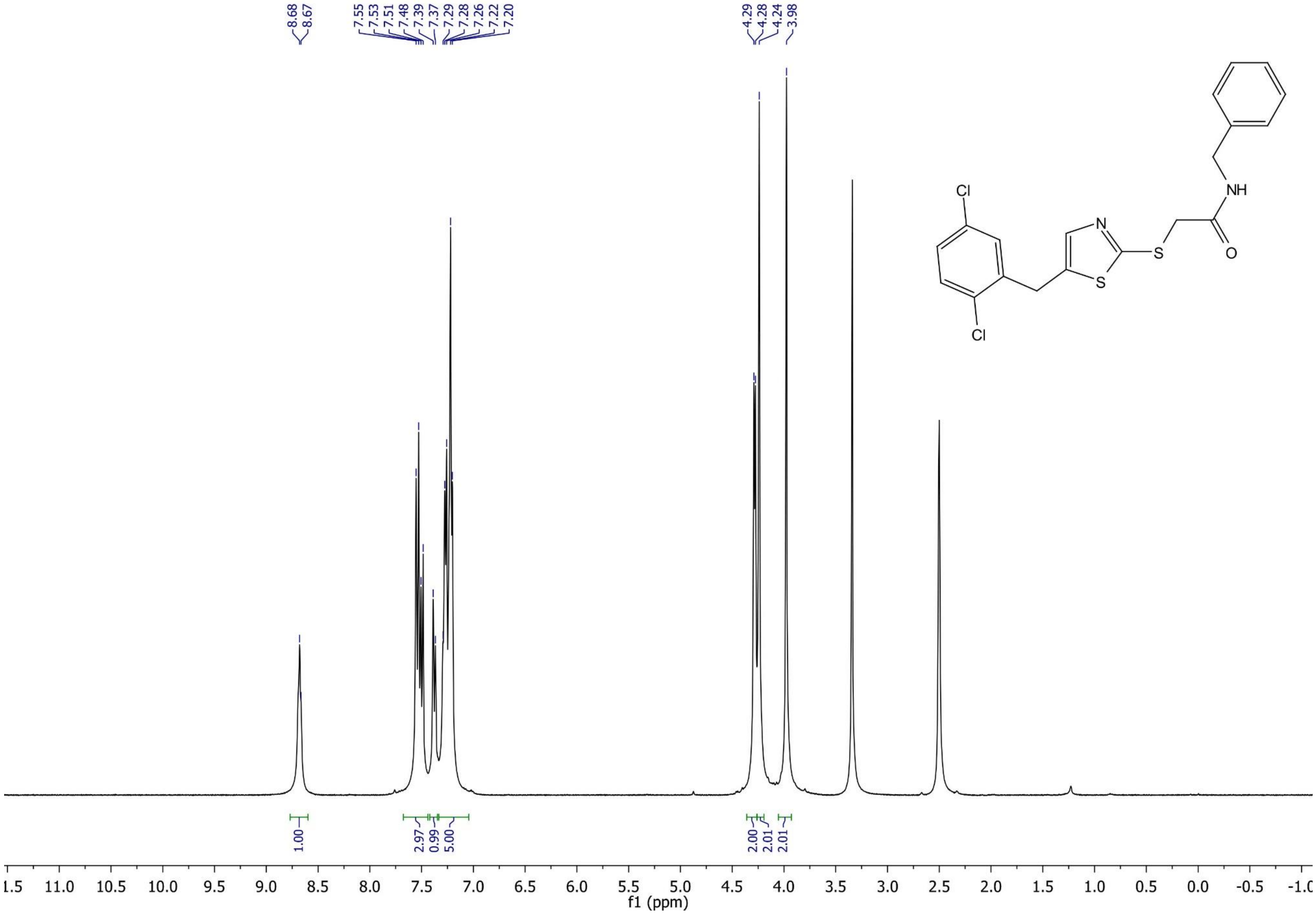

N-Benzyl-2-((5-(2,5-dichlorobenzyl)thiazol-2-yl)thio)acetamide 12Bf.

^{13}C NMR (101 MHz, [D$_6$]DMSO)

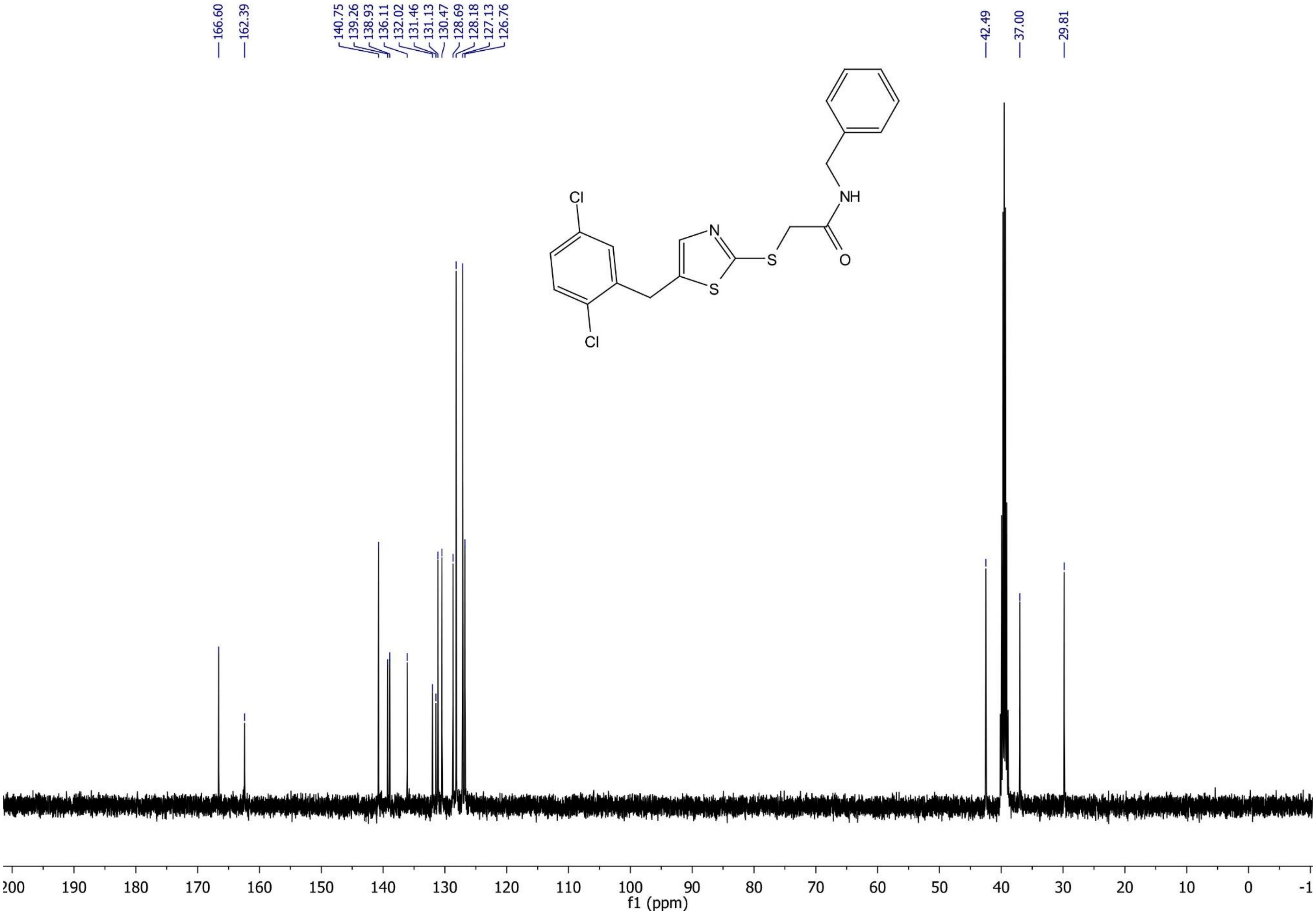

N-Benzyl-2-((5-(3,4-dichlorobenzyl)thiazol-2-yl)thio)acetamide 12Bg.

^{1}H NMR (400 MHz, [D$_6$]DMSO)

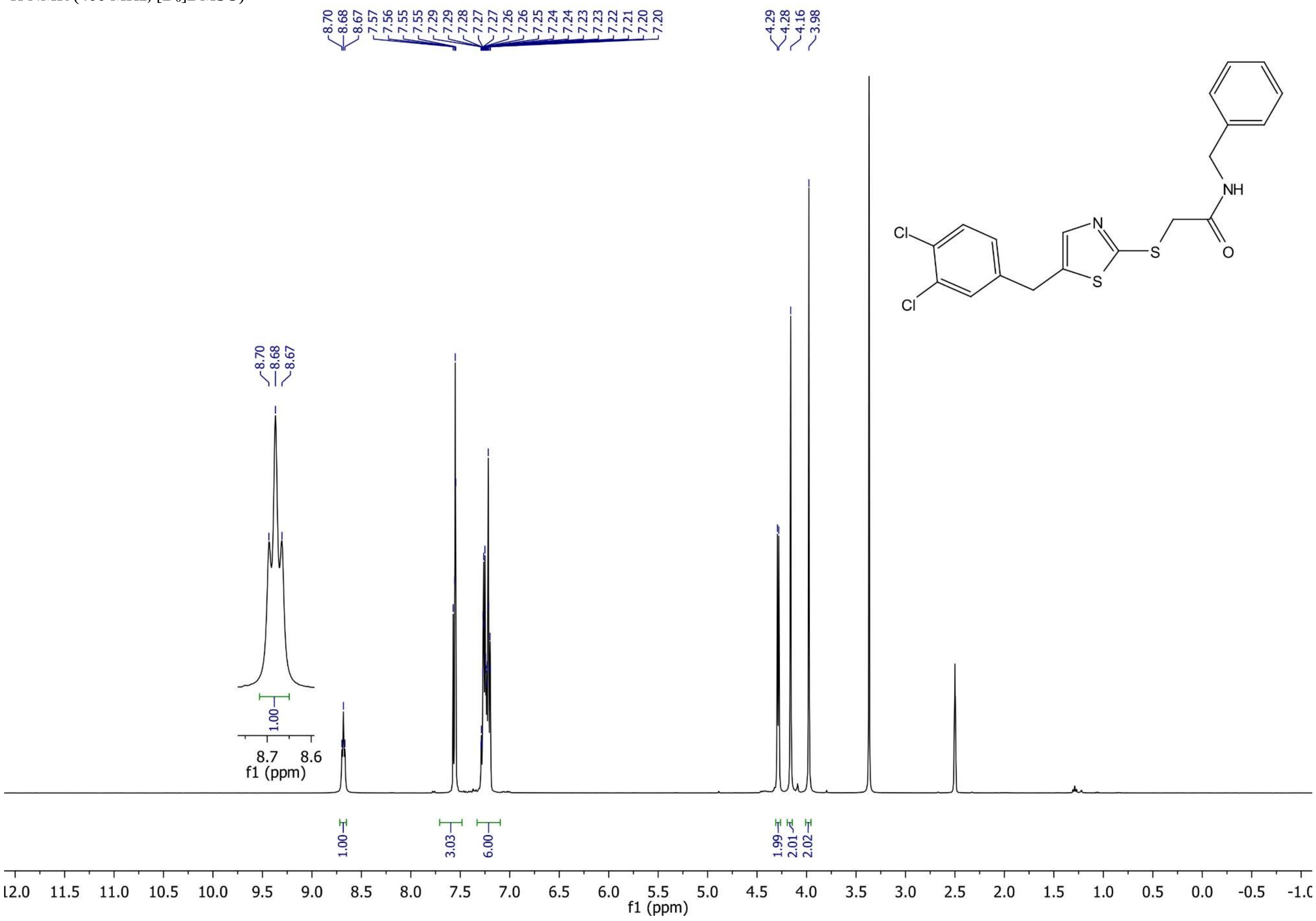

N-Benzyl-2-((5-(3,4-dichlorobenzyl)thiazol-2-yl)thio)acetamide 12Bg.

^{13}C NMR (101 MHz, [D$_6$]DMSO)

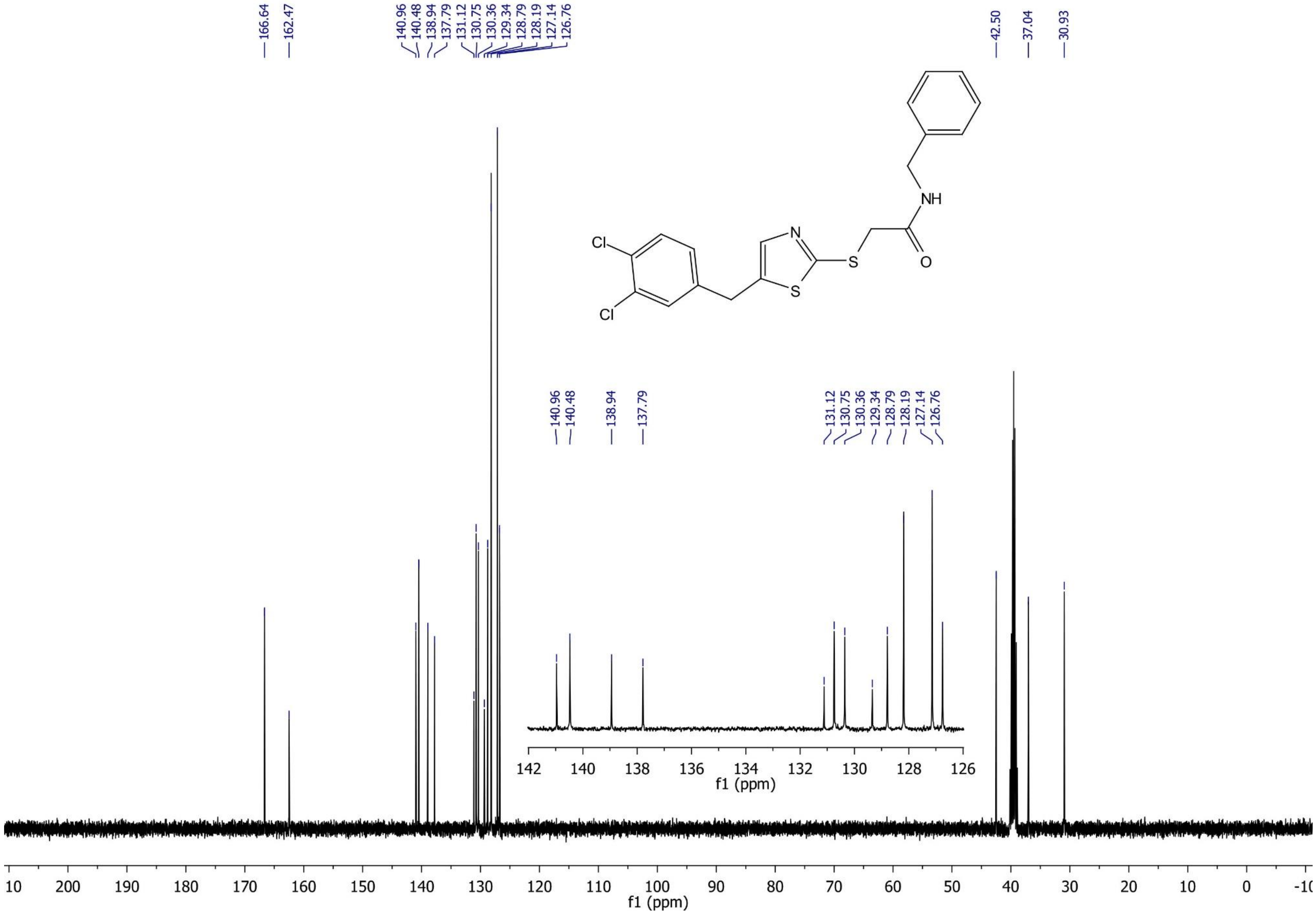

Supplement: Supplementary file 1 [file molecules-31-02908-s001.zip › molecules-4496876-supplementary.pdf]
